# Phylogenetic relationships and biogeographical patterns in Circum-Mediterranean subfamily Leuciscinae (Teleostei, Cyprinidae) inferred from both mitochondrial and nuclear data

**DOI:** 10.1186/1471-2148-10-265

**Published:** 2010-08-31

**Authors:** Silvia Perea, Madelaine Böhme, Primož Zupančič, Jörg Freyhof, Radek Šanda, Müfit Özuluğ, Asghar Abdoli, Ignacio Doadrio

**Affiliations:** 1 Museo Nacional de Ciencias Naturales-CSIC. Department of Biodiversity and Evolutionary Biology. José Gutiérrez Abascal 2, 28006 Madrid. Spain; 2 Senckenberg Centre for Human Evolution and Paleoecology (HEP) and Institute for Geoscience, University Tübingen, Sigwartstr. 10, D-72076 Tübingen, Germany; 3 AZV Agency. Dolsko 14, S1-1262. Slovenia; 4 Leibniz-Institute of Freshwater Ecology and Inland Fisheries. Müggelseedamm 310, 12587 Berlin. Germany; 5 National Museum. Václavské náměstí 68, 115 79 Prague 1. Czech Republic; 6 Istanbul University. Faculty of Science. Department of Biology, 34134 Vezneciler, İstanbul. Turkey; 7 Department of Biodiversity and Ecosystem Management, Environmental Sciences Research Institute. Shahid Beheshti University G. C., Tehran, Iran

## Abstract

**Background:**

Leuciscinae is a subfamily belonging to the Cyprinidae fish family that is widely distributed in Circum-Mediterranean region. Many efforts have been carried out to deciphering the evolutionary history of this group. Thus, different biogeographical scenarios have tried to explain the colonization of Europe and Mediterranean area by cyprinids, such as the "north dispersal" or the "Lago Mare dispersal" models. Most recently, Pleistocene glaciations influenced the distribution of leuciscins, especially in North and Central Europe. Weighing up these biogeographical scenarios, this paper constitutes not only the first attempt at deciphering the mitochondrial and nuclear relationships of Mediterranean leuciscins but also a test of biogeographical hypotheses that could have determined the current distribution of Circum-Mediterranean leuciscins.

**Results:**

A total of 4439 characters (mitochondrial + nuclear) from 321 individuals of 176 leuciscine species rendered a well-supported phylogeny, showing fourteen main lineages. Analyses of independent mitochondrial and nuclear markers supported the same main lineages, but basal relationships were not concordant. Moreover, some incongruence was found among independent mitochondrial and nuclear phylogenies. The monophyly of some poorly known genera such as *Pseudophoxinus *and *Petroleuciscus *was rejected. Representatives of both genera belong to different evolutionary lineages. Timing of cladogenetic events among the main leuciscine lineages was gained using mitochondrial and all genes data set.

**Conclusions:**

Adaptations to a predatory lifestyle or miniaturization have superimposed the morphology of some species. These species have been separated into different genera, which are not supported by a phylogenetic framework. Such is the case of the genera *Pseudophoxinus *and *Petroleuciscus*, which real taxonomy is not well known. The diversification of leuciscine lineages has been determined by intense vicariant events following the paleoclimatological and hydrogeological history of Mediterranean region. We propose different colonization models of Mediterranean region during the early Oligocene. Later vicariance events promoted Leuciscinae diversification during Oligocene and Miocene periods. Our data corroborate the presence of leuciscins in North Africa before the Messinian salinity crisis. Indeed, Messinian period appears as a stage of gradually Leuciscinae diversification. The rise of humidity at the beginning of the Pliocene promoted the colonization and posterior isolation of newly established freshwater populations. Finally, Pleistocene glaciations determined the current European distribution of some leuciscine species.

## Background

Mediterranean freshwater fish fauna is characterized by a relatively low number of fish families, with most of the species belonging to Cyprinidae [1,2]. In effect, this family is among the most speciose families of freshwater fishes and likely to be one of the largest vertebrate families [3]. The family Cyprinidae also features a relatively high number of endemic species on the Mediterranean slope [2] and its wide biological and ecological plasticity has bestowed this group an important role in biogeographical models [4-7]. As dispersion mechanisms are highly restricted in primary freshwater fishes [8], their distributions are directly related to paleogeography and relationships among different areas [9-11]. Thus, phylogenetic relationships among evolutionary lineages reflect the history of cyprinids within the Mediterranean region.

The classification of cyprinids has always been a matter of controversy and from 2 to 12 subfamilies have been recognized depending on author and the morphological traits considered [12-15] and even have been recently elevated to family level, being assigned European and North American leuciscins and phoxinins to the new called family Leuciscidae [16,17]. Traditional morphology, however, sometimes conflicts with the more recent molecular studies [5,18-23] because some morphological characters are prone to homoplasy [5] and usually there is unclear homology of morphological traits [24,25]. This determines that recognized monophyletic groups are clearly misinterpreted. In this paper, we followed the classification scheme of Saitoh et al. [22] based on complete mitochondrial genomes. These authors consider the subfamily Leuciscinae exclusively formed by the Eurasian and North African leuciscins (including the North American genus *Notemigonus*, as the only known representative in this region) and promote phoxinins [15] to their own subfamily, Phoxininae, as the sister group of the subfamily Leuciscinae. To date, however, the nuclear phylogenetic relationships of cyprinids, and particularly leuciscins, have been little explored [26,27].

The fossil record of Cyprinidae family in Eurasia [28-30] suggests its origin in East and Southeast Asia, where greatest generic and species diversity is found [1,3,31]. This group then colonized Europe for the first time during the Oligocene period when the Ob Sea disappeared because of the uplifting of the Urals, and reached the Iberian Peninsula (the westernmost part of Europe) in the late Oligocene-early Miocene [32]. The colonization of North Africa has been explained by connections with Iberian and Asian fish faunas during Cenozoic period [33]. Within the family Cyprinidae, the subfamily Leuciscinae may be used to test biogeographical hypotheses for the Mediterranean basin. In addition, owing to its long-standing distribution range in the Circum-Mediterranean area it should be possible to unravel the evolutionary history of this group.

Traditional hypotheses have proposed the origin of the subfamily Leuciscinae in the Mediterranean area and its subsequent diversification through river captures from central Europe as several waves stretched across a long time period (from the Oligocene until late Pliocene, 35-1.7 mya) [34,35], following the hydrogeographical and geological history of the European area [36]. This model has been designated as the "north dispersal model" [37]. Hypotheses opposing this model have argued that the colonization of Circum-Mediterranean rivers cannot be explained by a northern route. The alternative model proposed is based on leuciscine dispersion during the lacustrine stage of the Mediterranean basin [38], when this sea became refilled with fresh water from Paratethys Sea during the Messinian salinity crisis [39]. This would have allowed Paratethyan freshwater icthyofauna to colonize the Mediterranean margins. This hypothesis is described as the "Lago Mare" dispersal model [38]. The later opening of the Straits of Gibraltar [40] filled the Mediterranean area with marine water, with the subsequent isolation of the new-formed freshwater populations along with intense vicariant events [41,42]. However, the Lago Mare and north dispersal hypotheses are not mutually exclusive and together could have played an important role on dispersal of cyprinids across Europe [1,11,43]. On the other hand, the Middle East has been considered an important interchange area for freshwater ichthyofauna during the gradual closing of the Tethys Sea [6,44,45]. It is in fact held by some authors that the Middle Eastern freshwater fauna is made up of species that came from Asia and more recently from Euromediterranean ancestors [6,45,46]. The basis for this latter proposal is the close affinity found between Middle Eastern and Euromediterranean cyprinids [6,47]. This region has been also recognized as a center of origin for some Leuciscinae species [6,11,48].

Most recently, Pleistocene glaciations influenced the distribution of Leuciscinae representatives, especially in North and Central Europe, where some species became locally extinct when the region was covered by ice [35,49]. Later recolonization from eastern refuges such as Circum-Black Sea drainages has been suggested to explain the wide distribution of some extant cyprinid species [11,50,51]. Although the Mediterranean Peninsulas and Caspian/Caucasus region are known to have acted as glacial refuges [52,53], the Iberian, Italian and Balkan Peninsulas were isolated from northern and central Europe because of the Alps uplift during the Pleistocene thus preventing most Mediterranean freshwater species moving northwards. This interpretation explains the low level of shared freshwater species between north-central and southern Europe.

Weighing up all biogeographical scenarios, some models have attempted to explain the center of origin and the main dispersion routes for cyprinids [1,6,11,34-36,47,48,54,1], while others have tried to identify barriers to explain the vicariant patterns observed in cyprinid fishes [4,5,14,25,32,33,37,43,55]. Despite these efforts, the phylogenetic relationships of Circum-Mediterranean leuciscins and their biogeographical patterns in Mediterranean area remain unclear.

To obtain reliable information on the mitochondrial phylogenetic structure of this group, the comprehensive study examines mitochondrial DNA in numerous species of the subfamily Leuciscinae. Indeed, sequences of the cytochrome *b *(cyt *b*) gene have achieved phylogenetic resolution in some fish groups [56,57], including cyprinids [4-7,11,18,20,37,43,48,58]. In turn the cytochrome oxidase I (COxI) gene has also proved to be a useful tool for the identification of fish species [59,60]. Here we complete the mitochondrial phylogeny of the subfamily Leuciscinae using nuclear information by analyzing the Recombination Activating Gene 1 (RAG-1) and the Ribosomal Protein Gene S7 (S7). Only a few previous molecular studies on cyprinids have yielded a nuclear phylogeny of Circum-Mediterranean representatives of the subfamily Leuciscinae. Some leuciscine groups have been examined using nuclear allozymes [10,61,62] or DNA sequences [63-65] approaches and some phylogenetic relationships have been proposed for cyprinids [66]. However, this paper constitutes the first attempt at deciphering the nuclear relationships of the main Mediterranean Leuciscinae lineages based on sequences data.

The aim of this exhaustive study was to investigate phylogenetic relationships among the major Circum-Mediterranean Leuciscinae lineages by analyzing sequence variation of both mitochondrial (cyt *b *and COxI) and nuclear (RAG-1 and S7) genes. Data were obtaining for 321 individuals representing 176 species of the subfamily Leuciscinae and 9 outgroup species. The data were used to test for biogeographical events that could have determined the distribution of leuciscins in Mediterranean area.

## Results

### Leuciscinae phylogenetic performance

Out of a total of 4439 characters obtained, 1786bp corresponded to mitochondrial DNA and 2653bp to nuclear DNA. Table 1 compares the phylogenetic performance of the individual genes under the taxon sampling in phylogenetic analyses. ML parameters estimated using Modeltest v3.7 [67] are provided in Table 2. The χ^2 ^test for base homogeneity indicated that base frequency distributions were always homogenous among taxa. The nuclear genes showed much higher consistency (CI) and retention (RI) indices, while the mitochondrial genes offered more parsimony informative (PI) sites. Indeed, the proportion of informative sites showed by cyt *b *gene was the highest (Table 1).

**Table 1:** Comparison of the phylogenetic performance for each individual gene and the combined data set.

GENE	TOTAL CHARACTERS	PARSIMONY INFORMATIVE CHARACTERS (in %)	Tv/Ts RATE	LENGTH parsimony tree	CI	RI	HI
CYT B	1140	562 (49.29%)	5.45	8995	0.233	0.769	0.882

COxI	646	242 (37.46%)	0.79	2322	0.199	0.797	0.801

NUCLEAR DATA SET (RAG1+S7)	2647	600 (22.66%)	0.87	2531	0.637	0.795	0.363

COMBINED GENES (Cytb+COxI+RAG1+S7)	4339	1258 (28.37%)	1.94	10913	0.371	0.556	0.729

**Table 2:** Parameters of ML analyses estimated with Modeltest under Akaike criterion

	Cytb	COxI	RAG1	S7
**Model selected by Modeltest** **(AIC criteria)**	GTR+I+G	GTR+I+G	K81+G	K81+G

**Rates substitution matrix**	[A-C] = 0.61	[A-C] = 1.00	[A-C] = 1.93	[A-C] = 1.00
	[A-G] = 26.57	[A-G] = 17.05	[A-G] = 5.09	[A-G] = 1.56
	[A-T] = 0.35	[A-T] = 1.00	[A-T] = 1.23	[A-T] = 0.64
	[C-G] = 1.62	[C-G] = 1.00	[C-G] = 0.54	[C-G] = 0.64
	[C-T] = 6.60	[C-T] = 9.43	[C-T] = 5.09	[C-T] = 1.56
	[G-T] = 1.00	[G-T] = 1.00	[G-T] = 1.00	[G-T] = 1.00

**Assumed proportion of invariable sites (I)**	0.36	0.57	0	0

**Alpha (G)**	0.65	0.98	0.46	1.02

### Mitochondrial phylogenetic relationships

All mitochondrial analyses generated almost identical and well-supported topologies. Our discussion therefore focuses on the more resolved Bayesian tree and the results of the Maximum likelihood (ML) and Maximum Parsimony (MP) analyses are summarized. Bayesian analysis [68,69] has been empirically demonstrated to be the most efficient character-based method for accurately reconstructing a phylogeny [70].

**Figure 1:**
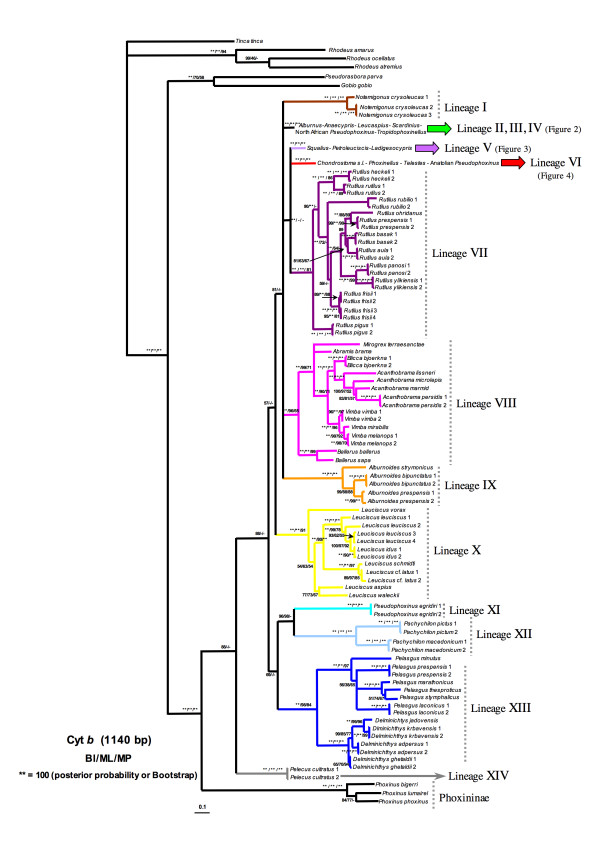
**Phylogenetic tree rendered by Bayesian analysis of the mitochondrial cytochrome *b *data set**. Numbers above branches means posterior probabilities of BI/Bootstrap values of ML/Bootstrap values of MP.

According to our molecular mitochondrial cyt *b *data, the subfamily Leuciscinae comprises fourteen major monophyletic lineages. All lineages were supported by high posterior probability and bootstrap values (Figure 1).

**Figure 2:**
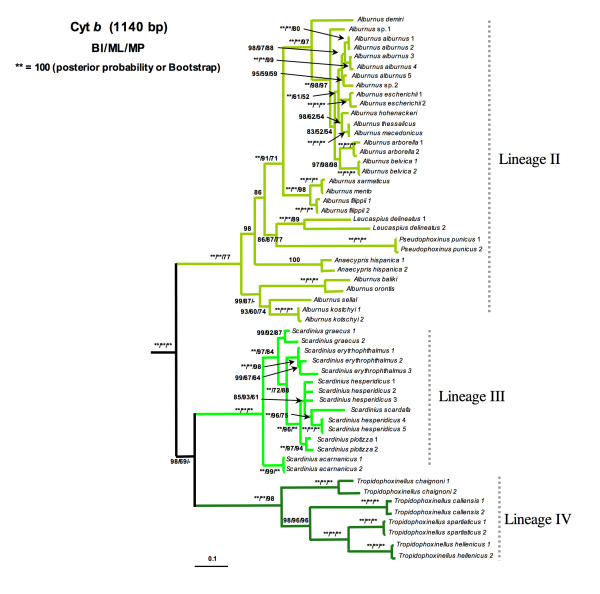
**Phylogenetic tree rendered by Bayesian analysis of the mitochondrial cytochrome *b *gene for *Alburnus*-*Anaecypris*-*Leucaspius*-*Pseudophoxinus punicus *lineage**. Numbers above branches means posterior probabilities of BI/Bootstrap values of ML/Bootstrap values of MP.

**Figure 3:**
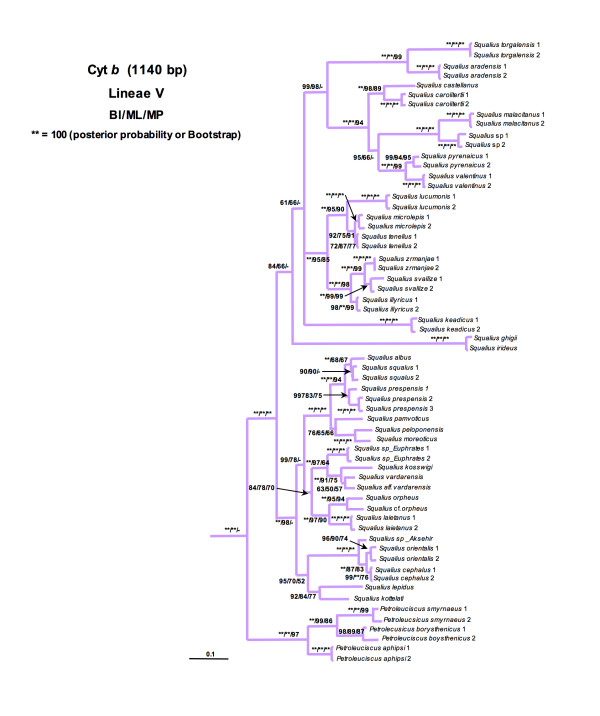
**Phylogenetic tree rendered by Bayesian analysis of the mitochondrial cytochrome *b *gene for *Squalius-Petroleuciscus *lineage**. Numbers above branches means posterior probabilities of BI/Bootstrap values of ML/Bootstrap values of MP.

**Figure 4:**
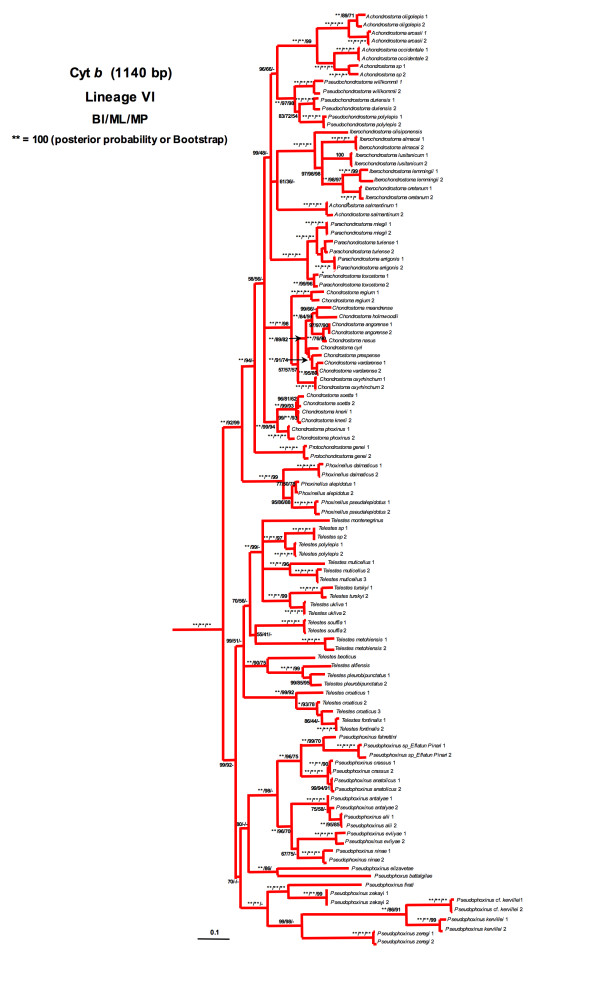
**Phylogenetic tree rendered by Bayesian analysis of the mitochondrial cytochrome *b *gene for *Chondrostoma s.l*.-Phoxinellus-Telestes-Anatolian *Pseudophoxinus *lineage**. Numbers above branches means posterior probabilities of BI/Bootstrap values of ML/Bootstrap values of MP.

Lineage I was formed by the monotypic North American genus *Notemigonus *(*N. crysoleucas*). Lineage II comprised the widespread genus *Alburnus*, the monotypic genera *Leucaspius *and *Anaecypris *and the North African species *Pseudophoxinus punicus*. Although *L. delineatus *appeared as more related with *P. punicus*, this relationship was not highly supported (Figure 2). Lineage III was formed by the genus *Scardinius*. Lineage IV comprised the two species of the Greek genus *Tropidophoxinellus *and the two North African species *Pseudophoxinus chaignoni *and *P. callensis*. Lineage V included the genera *Squalius*, *Ladigesocypris *(*L. ghigii *and *L. irideus*) and two species of *Petroleuciscus *(*P. borysthenicus *and *P. smyrnaeus*) (Figure 3). The genus *Squalius *was subdivided into two well-differentiated clades. The "*S. cephalus *species group" clade, which was in turn structured into two subclades: the first subclade included central European and Mediterranean species while the second subclade consisted of Anatolian and Caucasian species. The second large *Squalius *clade was structured into three subclades: the Iberian subclade, the Adriatic subclade and the Greece subclade formed by the species *S. keadicus*. *Ladigesocypris *were nested into the *Squalius *clade and *Petroleuciscus *were positioned basal to all species of *Squalius*. Lineage VI was comprised of the former genus *Chondrostoma s.l*., which has been recently subdivided into six genera [64]: *Achondrostoma, Chondrostoma, Iberochondrostoma, Parachondrostoma, Protochondrostoma *and *Pseudochondrostoma*. The genera *Phoxinellus*, *Telestes *and the Anatolian representatives of the genus *Pseudophoxinus *also clustered in this lineage (Figure 4). *Phoxinellus *and *Chondrostoma s.l*., together represented the sister group of *Telestes *and the Anatolian *Pseudophoxinus*. The Anatolian *Pseudophoxinus *species were represented by two monophyletic and well-differenciated clades: one of them including the species *P. alii, P. anatolicus*, *P. antalyae*, *P. battalgilae, P. crassus*, *P. elizevetae, P. evliyae, P. fahretinne, P. ninae *and one possibly undescribed species. The second one was formed by *P. firati*, *P. kervillei*, *P. zekayi *and *P. zeregi*. Lineage VII constituted the genus *Rutilus *and lineage VIII contained the genera *Abramis*, *Acanthobrama*, *Acanthalburnus, Ballerus, Blicca*, *Mirogrex *and *Vimba*, together with the Iranian species *Petroleuciscus persidis*. Lineage IX was exclusively represented by the genus *Alburnoides*. Lineage X was composed by the genera *Aspius *and *Leuciscus*. Lineage XI was comprised only by the species *Pseudophoxinus egridiri*. Lineage XII was formed by the genus *Pachychilon*. Lineage XIII comprised the genera *Pelasgus *and *Delminichthys*. Finally lineage XIV was formed by the monotypic genus *Pelecus*, which was the first lineage to split.

**Figure 5:**
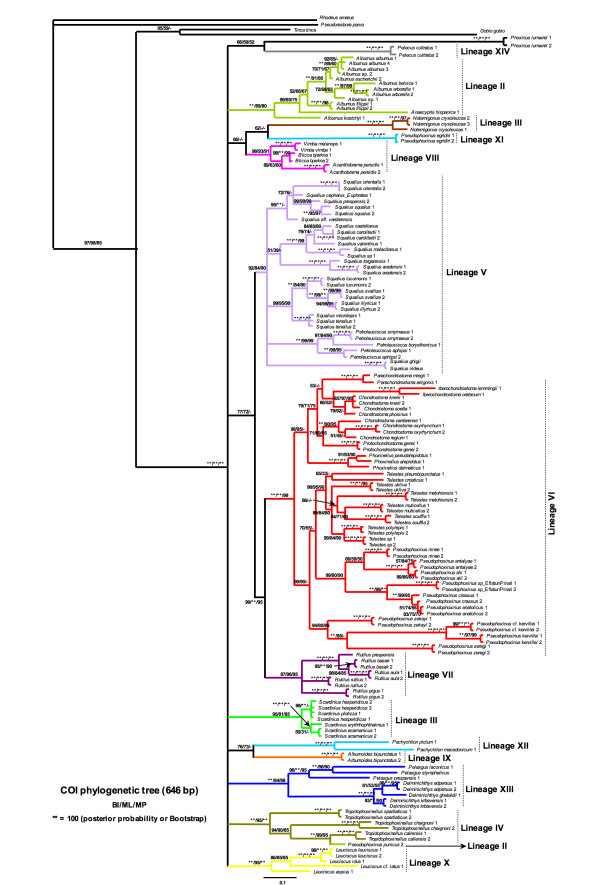
**Phylogenetic tree rendered by Bayesian analysis of the mitochondrial cytochrome oxidase I gene**. Numbers above branches means posterior probabilities of BI/Bootstrap values of ML/Bootstrap values of MP.

The cytochrome oxidase I gene (COxI) rendered the same main lineages as cytochrome *b *but also detected a deep politomy among all lineages (Figure 5). Thus, excluded *Pelecus *basal to the remaining leuciscine lineages with regarding to the phylogenetic relationships between different lineages, deep relationships between them were not well solved with mitochondrial markers.

Moreover, some incongruence was found among cyt *b *and COxI topologies, such as *Pseudophoxinus punicus*, which was more related with *P. callensis *than with the Alburninae group, as well as *Pachychilon*, which was closer to *Alburnoides *lineage than to *Pelasgus-Delminichthys *and *Pseudophoxinus egridiri *lineages. On the other hand, the Lineages II (*Alburnus, Anaecypris, Leucaspius *and *Pseudophoxinus punicus*), III (*Scardinius*) and IV (*Pseudophoxinus callensis, P. chaignoni, Tropidophoxinellus*) were clustered together and highly support with cytochrome *b*, but were unrelated with COxI gene.

### Nuclear phylogenetic relationships

**Figure 6:**
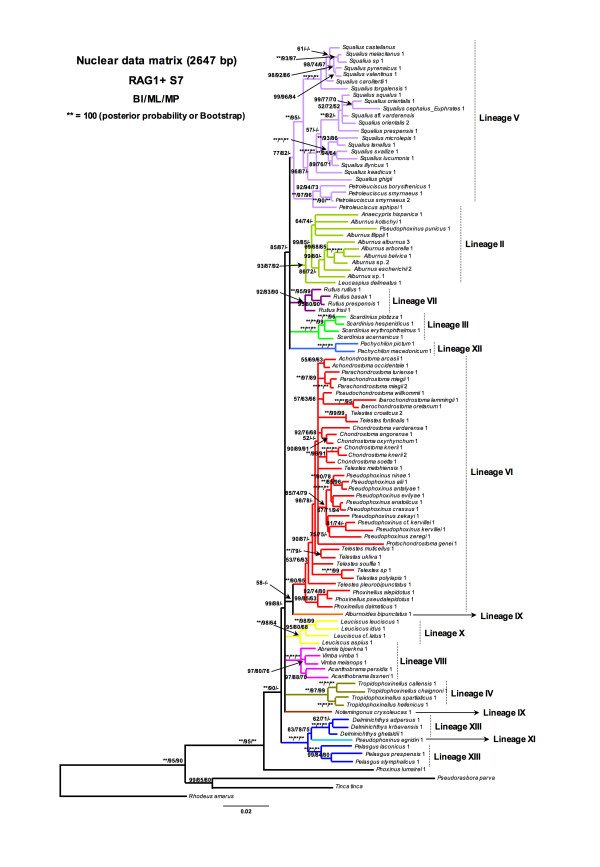
**Phylogenetic tree rendered by Bayesian analysis of the nuclear dataset (RAG1+S7 genes)**. Numbers above branches means posterior probabilities of BI/Bootstrap values of ML/Bootstrap values of MP.

Although the number of species is lesser in nuclear analyses than in mitochondrial one, the topologies of both mitochondrial and combined nuclear genes (RAG1 + S7) support the identical major lineages. However, deep relationships among lineages were not solved with nuclear markers (Figure 6). Even within some lineages as number VI (*Chondrostoma s.l., Telestes *and Anatolian *Pseudophoxinus*) basal relationships were not solved. As occurred in COxI topology, the lineages II, III and IV were unrelated with nuclear genes. Moreover, some incongruence was found between mitochondrial and nuclear data. Such is the case of *Pachychilon*, which was not related with *Pelasgus-Delminichtys-Pseudophoxinus egridiri*. In turn, *P. egridiri *was nested, and highly support, within *Pelasgus-Delminichthys *lineage.

### Phylogenetic relationships with all-genes data matrix

**Figure 7:**
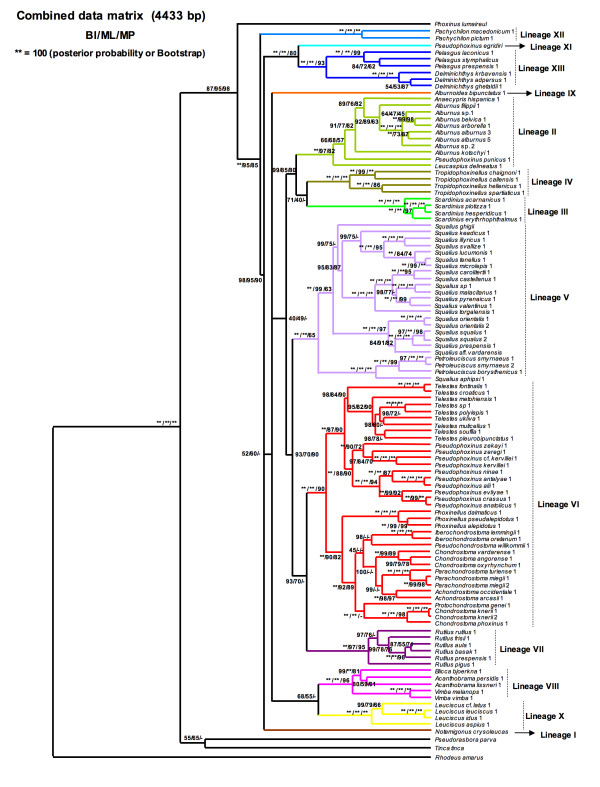
**Phylogenetic tree rendered by Bayesian analysis of the all-genes data set (Cytochrome *b*-COxI-RAG1+S7 genes)**. Numbers above branches means posterior probabilities of BI/Bootstrap values of ML/Bootstrap values of MP.

Combined data analysis recovered the major lineages and increased their support (posterior probabilities and bootstrap values higher than only mitochondrial and nuclear analysis) (Figure 7). Indeed, this effect can be explained because large number of variable characters can improve the accuracy of reconstructed relationships [71]. Nevertheless, the topology of combined data was very similar to that obtained by cytochrome *b*, due to the higher number of informative-parsimony characters of this gene respect to the others (Table 1).

### Divergence time

**Figure 8:**
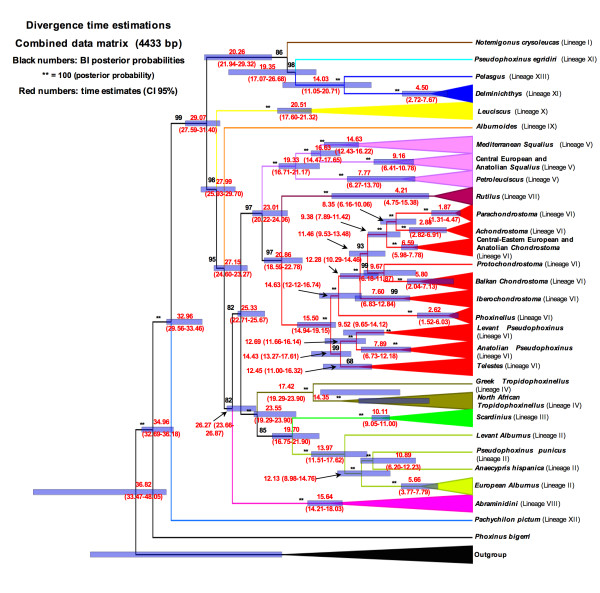
**Timing of the major cladogenetic events in leuciscin lineages based on a relaxed molecular clock and in all-genes data set**. Black numbers represent posterior probability values for BI (** symbol means a value of posterior probability equal to 100). Red numbers represent divergence times and their HPD 95% confidence intervals.

A relaxed molecular clock was performed based on mitochondrial cytochrome *b *gene (not showed) and all-genes data set. Our analysis supports the divergence of the main lineages occurred in Late Oligocene-Early Miocene. The timing of splitting-events of the Circum-Mediterranean leuciscine lineages is reported in Figure 8.

## Discussion

### Phylogenetic relationships of Leuciscinae lineages

Although our study is focused on the Circum-Mediterranean Leuciscinae representatives, the inclusion of the majority of west Paleartic lineages allowed a more accuracy resolution of their phylogenetic framework, and subsequently of their evolutionary history. We have based our phylogenetic discussion in the all-genes topology, which was the most solved and supported tree and summarizes the information of all analyzed genes.

Although some studies have tried to explain the phylogenetic relationships of Mediterranean leuciscins [4,5,18,37,43,63,64], this study is the first molecular approximation to the phylogenetic placement of the three very poorly studied, small size North African *Pseudophoxinus *species: *P. callensis, P. chaignoni *and *P. punicus*. We have demonstrated here that none of this three species belong to the real genus *Pseudophoxinus*. Morphological approaches have already hypothesized a close relationship among *P. callensis *and *P. chaignoni *in relation with *P. punicus *[72]. Our molecular phylogeny corroborate this later affirmation, and whereas *P. callensis *and *P. chaignoni *were closely related, and in turn clustered with the genus *Tropidophoxinellus*, *P. punicus *was nested within the *Alburnus *and *Leucaspius *group. The phylogenetic positions of the North African species *P. punicus *and the genera *Leucaspius *and *Anaecypris *strongly suggest that these species belong to the lineage of *Alburnus*. The inclusion of these genera turns into paraphyletic the genus *Alburnus*. For this reason, this issue opens the question about whether the group formed by *Alburnus baliki, A. orontis *and *A. sellal *from Anatolia and Levant should represent an independent genus or if the monotypic miniature *Anaecypris *and *Leucaspius *should be included into *Alburnus*. The inclusion of *P. punicus *into the *Alburnus *lineage has been already proposed based on morphology [72,73], as well as previous morphological [18,73] and molecular studies [5] have already hypothesized a close relationship of *Anaecypris *and *Leucaspius *with *Alburnus*. Moreover, as other dwarf leuciscine, *Anaecypris *also have been placed in *Pseudophoxinus *or *Phoxinellus *by some authors [74,75]. As in other small sized and specialized cyprinids as the Leuciscinae *Delminichthys *[63] and *Pelasgus *[2] or the Rasborinae *Paedocypris *[23], synapomorphic characters seem to have been overshadowed by morphological changed connected to miniaturization. However, further specific studies are required to solve these questions. In turn, *P. callensis *and *P. chaignoni *are here transferred provisionally to the genus *Tropidophoxinellus *(See Table 3).

**Table 3:** Taxonomy of subfamily Leuciscinae based on the phylogenetic results obtained in this study and lineages that need further review.

Previous Taxonomy	Proposed taxonomy after this study
**Genus *Notemigonus *(Lineage I)**	
*N. crysoleucas *[152]	*N. crysoleucas*

**Genus *Alburnus *(Lineage II)**	
*Al. alburnus *[2]; *Al. arborella *[2]; *Al. belvica *[2]; *Al. demiri *[153]*; Al. escherichii *[154]; *Al. filippii *[154]; *Al. hohenackeri *[2]; *Al. macedonicus *[2]; *Al. mento *[2]; *Al. orontis *[154]; *Al. sarmaticus *[2]; *Al. thessalicus *[2]	Without changes
*Alburnus *populations from Kizilimark R.	*Alburnus *sp. 1
*Alburnus *populations from Stryrmon R.	*Alburnus *sp. 2
	
*Al. sellal *[154]; *Al. baliki *[155]; *Al. kotschyi *[156]; *Al. orontis *[154]	A new genus should be described for these species to avoid the paraphyletic status of *Alburnus *(Type species: *A. alburnus*)

**Genus *Anaecypris *(Lineage II)**	
*An. hispanica *[2]	*An. Hispanica*

**Genus *Leucaspius *(Lineage II)**	
*L. delineatus *[2]	*L. delineatus*

**Genus *Pseudophoxinus *(Lineage II)** *Ps. punicus *[157]	A new genus should be described for this species because real *Pseudophoxinus *(type species *P. zeregi*) belong to a different lineage

**Genus *Scardinius *(Lineage III)**	
*Sc. acarnanicus *[2]; *Sc. erythrophthalmus *[2]; *Sc. graecus *[2]; *Sc. hesperidicus *[2]; *Sc. plotizza *[2]; *Sc. scardafa *[2]	Without changes

**Genus *Tropidophoxinellus *(Lineage IV)**	**Genus *Tropidophoxinellus***
*Tr. hellenicus *[2]; *Tr. spartiaticus *[2]	Without Changes
**Genus *Pseudophoxinellus***	
*Ps. callensis *[77]	*Tr. callensis*
*Ps. chaignoni *[77]	*Tr. chaignoni*

**Genus *Petroleuciscus *(Lineage V)**	Without changes
*Pe. borysthenicus *[2], *Pe. smyrnaeus *[2]	**Genus *Petroleuciscus***
**Genus *Squalius***	
*Sq. aphipsi *[2]	*Pe. aphipsi* Necessary to analyze all species considered as belonging to *Petroleuciscus *(*P. kurui, P. squaliusculus, P. ulanus*)

**Genus *Ladigesocypris *(Lineage V)**	**Genus *Squalius***
*La. ghigii *[2] *La. irideus *[2]	*Sq. ghigii* *Sq. irideus*
**Genus *Squalius *(Lineage V)**	
*Sq. albus *[2]; *Sq. aradensis *[2]; *Sq. carolitertii *[2]; *Sq. castellanus *[158]; *Sq. cephalus *[2]; *Sq. illyricus *[2]; *Sq. keadicus *[2]; *Sq. kosswigi *[159]; *Sq. kottelati *[159]; *Sq. laietanus *[2]; *Sq. lepidus *[2]; *Sq. lucumonis *[2]; *Sq. malacitanus *[2]; *Sq. microlepis *[2]; *Sq. moreoticus *[2]; *Sq. orientalis *[2]; *Sq. orpheus *[2]; *Sq. pamvoticus *[2]; *Sq. peloponensis *[2]; *Sq. prespensis *[2]; *Sq. pyrenaicus *[2]; *Sq. squalus *[2]; *Sq. svallize *[2]; *Sq. tenellus *[2]; *Sq. torgalensis *[2]; *Sq. valentinus *[2]; *Sq. vardarense *[2]; *Sq. zrmanjae *[2]	Without changes
Squalius populations from Southern Spain	*Squalius *sp
Squalius populations from Aksheir L. Turkey	*Squalius *sp_Euphrates
Squalius populations from Euphrates drainage	*Squalius *sp_Aksheir
Squalius populations from Rama L. BiH	*Squalius aff. vardarensis*
Squalius populations from Manikiotico R.	*Squalius *cf. *orpheus*

**Genus *Achondrostoma *(Lineage VI)**	***Achondrostoma***
*Ach. arcasii *[2]; *Ach. occidentale *[2]; *Ach. salmantinum *[158]; *Ach. oligolepis *[2] *Achondrostoma *populations from NE Spain	Without changes *Achondrostoma sp*

**Genus *Chondrostoma *(Lineage VI)**	
*Ch. angorense *[160]; *Ch. cyri *[160]; *Ch. holmwoodii *[161]; *Ch. knerii *[2]; *Ch. meandrense *[160]; *Ch. nasus *[2]; *Ch. oxyrhynchum *[2]; *Ch. phoxinus *[2]; *Ch. prespensis *[2]; *Ch. regium *[160]; *Ch. soetta *[2]; *Ch. vardarense *[2] *Ch. olisiponensis *[162]	Without changes *Iberochondrostoma olisiponensis*

**Genus *Iberochondrostoma *(Lineage VI)**	
*Ib. almacai *[2];; *Ib. lemmingii *[2]; *Ib. lusitanicum *[2]; *Ib. oretanum *[2]	Without changes

**Genus *Parachondrostoma *(Lineage VI)**	
*Pa. arrigonis *[2]; *Pa. miegii *[2]; *Pa. toxostoma *[2]; *Pa. turiense *[2]	Without changes

**Genus *Phoxinellus *(Lineage VI)**	
*Ph. alepidotus *[2]; *Ph. dalmaticus *[2]; *Ph. pseudalepidotus *[2]	Without changes

**Genus *Protochondrostoma *(Lineage VI)**	
*Pr. genei *[2]	Without changes

**Genus *Pseudochondrostoma *(Lineage VI)**	
*Pse. duriense *[2]; *Pse. polylepis *[2]; *Pse. willkommii *[2]	Without changes

**Genus *Pseudophoxinus *(Lineage VI)**	
*Ps. alii *[77]; *Ps. anatolicus *[154]; *Ps. antalyae *[154]; *Ps. battalgilae *[154]; *Ps. crassus *[154]; *Ps. elizavetae *[77]; *Ps. evliyae *[163]; *Ps. fahrettini *[163]; *Ps. firati *[77]; *Ps. kervillei *[164]; *Ps. ninae *[63]; *Ps. zekayi *[77]; *Ps. zeregi *[165]	Without changes
*Pseudophoxinus *populations from Eflatun Pinari spring. Turkey	*Pseudophoxinus *sp_EflatunPinari

**Genus *Telestes *(Lineage VI)**	
*Te. alfiensis *[63]; *Te. beoticus *[2]; *Te. croaticus *[2]; *Te. fontinalis *[2]; *Te. metohiensis *[2]; *Te. montenegrinus *[2]; *Te. muticellus *[2]; *Te. pleurobipunctatus *[2]; *Te. polylepis *[2]; *Te. souffia *[2]; *Te. turskyi *[2]; *Te. ukliva *[2] *Telestes *populations from Dreznica. Croatia	Without changes *Telestes *sp.

**Genus *Rutilus *(Lineage VII)**	
*Ru. aula *[2]; *Ru. basak *[2]; *Ru. frisii *[2]; *Ru. heckeli *[2]; *Ru. ohridanus *[2]; *Ru. panosi *[2]; *Ru. pigus *[2]; *Ru. prespensis *[2]; *Ru. rubilio *[2]; *Ru. rutilus *[2]; *Ru. ylikiensis *[2]	Without changes

**Genus *Abramis *(Lineage VIII)**	
*Ab. brama *[2]	Without changes

**Genus *Acanthobrama *(Lineage VIII)**	**Genus *Acanthobrama***
*Ac. lissneri *[166]; *Ac. marmid *[154]	Without changes
**Genus *Acanthalburnus *(Lineage VIII)**	
*Aca. microlepis *[154]	*Ac. microlepis*
**Genus *Petroleuciscus *(Lineage VIII)**	
*Pe. persidis *[167]	*Ac. Persidis*

**Genus *Ballerus *(Lineage VIII)**	
*Ba. ballerus *[2]; *Ba. sapa *[2]	Without changes

**Genus *Blicca *(Lineage VIII)**	
*Bl. bjoerkna *[2]	Without changes

**Genus *Mirogrex *(Lineage VIII)**	
*Mi. terrasanctae *[164]	Without changes

**Genus *Vimba *(Lineage VIII)**	**Genus *Vimba***
*Vi. melanops *[2]; *Vi. vimba *[2]	Without changes
**Genus Acanthobrama**	
*Ac. mirabilis *[154] (*Vi. Vimba *by synonymy [6])	*V. mirabilis*

**Genus *Alburnoides *(Lineage IX)**	
*Alb. bipunctatus *[2]; *Alb. prespensis *[2]; *Alb. bipunctatus *[2]	Without changes

**Genus *Leuciscus *(Lineage X)**	**Genus *Leuciscus***
*Le. idus *[2]; *Le. latus *[167]; *Le. leuciscus *[2]; *Le. schdmidti *[46]; *Le. walecki *[168]	Without changes
**Genus *Aspius *(Lineage X)**	
*As. aspius *[2];	*Le. aspius*
*As. vorax *[2]	*Le. vorax*

**Genus *Pseudophoxinus *(Lineage XI)** *P. egridiri *[154]	A new genus should be described for this species because real *Pseudophoxinus *(type species *P. zeregi*) belong to a different lineage

**Genus *Pachychilon *(Lineage XII)**	
*Pac. macedonicum *[2]; *Pac. pictum *[2]	Without changes

**Genus *Delminichthys *(Lineage XII)**	
*D. adpersus *[2]; *D. ghetaldii *[2]; *D. jadovensis *[2]; *D. krbavensis *[2]	Without changes

**Genus *Pelasgus *(Lineage XIII)**	
*P. laconicus *[2]; *P. marathonicus *[2]; *P. minutus *[2]; *P. prespensis *[2]; *P. stymphalicus *[2]; *P. thesproticus *[2]	Without changes

**Genus *Pelecus *(Lineage XIV)**	
*P. cultratus *[2]	Without changes

The real genus *Pseudophoxinus *(type species *P. zeregi*) comprises the Anatolian and Levant species, which are clustered in the same lineage that *Telestes *(its sister group), *Phoxinellus *and the six new genera described for the former genus *Chondrostoma s. l*.: *Achondrostoma, Chondrostoma, Iberochondrostoma, Parachondrostoma, Protochondrostoma *and *Pseudochondrostoma *[64]. Within the Anatolian *Pseudophoxinus *two well-differentiated groups are recognized: one of them is the group formed by the complex of species inhabiting Central Anatolia (*P. alii, P. anatolicus, P. antalyae, P. battalgilae, P. crassus, P. elizavetae, P. evliyae, P. fahettini, P. ninae *and one possibly undescribed species) and the other one from the Levant (*P. firati, P. kervillei, P. zeregi *and *P. zekayi*). Relationships of *Pseudophoxinus *were not in agreement with morphological studies [76,77].

We also demonstrate here that *P. egridiri *forms a single group related to the *Delminichtys-Pelasgus *lineage instead of the rest of *Pseudophoxinus *species. The hypothesis that *P. egridiri *is not related to *Pseudophoxinus *was already proposed and suspicions that it might be more related to the genus *Phoxinus *have been formulated [78]. However, our data rejected this hypothesis and demonstrated that *P. egridiri *constitutes an independent phylogenetic lineage. All the species of *Delminichthys-Pelasgus *lineage are dwarf cyprinids mostly characterized by reductive traits but also have some own synapomorphic characters [63,76]. They have been erroneously included in the genus *Pseudophoxinus *or *Phoxinellus *[2,63,79]. Indeed, *Pseudophoxinus *was considered to be a subgenus of *Phoxinellus *for many decades [76].

The genus *Telestes *is closely related to the main group of *Pseudophoxinus *and we also support the position of *Telestes croaticus, T. fontinalis *and *T. metohiensis *into this genus as were previously pointed out [63]. These species were previously placed in the genus *Phoxinellus *[79].

The genus *Squalius *is widely distributed throughout Europe and is highly diversified in the Mediterranean area and three groups have been identified within this genus [32]: A Mediterranean group composed of small species from southern Spain, Central Italy, Southern Greece and the Balkans; an Euroasiatic group that is widely distributed throughout central-east Europe, Asia and the north of Mediterranean area and the Parathethys group, which includes species around the Black sea and Anatolia. Our phylogeny supports these groups, but we have complemented the taxonomical sampling, finding that *S. illyricus, S. microlepis, S. svallize *and *S. tenellus *also are included within the Mediterranean group reported by Sanjur et al. [32], as well as some Anatolian species in the Euroasiatic group. Furthermore, within the Mediterranean group a geographical structure is observed: an Iberian subgroup, a second Italo-Adriatic subgroup, and a third Greek subgroup (Peloponnesus), which is not found in the "*S. cephalus *group". As same as *Chondrostoma s. l*. phylogenetic relationships between all of these subgroups were not solved, showing basal politomies rather than bifurcating relationships. Concerning to "*Squalius cephalus*" complex, an underestimation on the diversity of this group is observed, a further taxonomic review is required, especially in Euphrates drainage. In contrast to the remaining *Squalius *species, "*S. cephalus*" complex did not show a geographical structure; thus, Greek *Squalius *species did not form a monophyletic group due to Iberian *S. laietanus *was closer to Greek *S. orpheus *[5,32,37] and Adriatic *S. squalus *was the sister group of Greek *S. prespensis*. However, phylogenetic relationships within Greek *Squalius *were in agreement with allozymic [10] and mitochondrial [80] previous studies.

The genus *Petroleuciscus *(the *Squalius *Paratethys group recognized by Sanjur et al. [32]) comprises six poorly known species: *P. borysthenicus *(type species), *P. kurui*, *P. smyrnaeus*, *P. persidis*, *P. squaliusculus *and *P. ulanus *[81]. This study represents the first molecular approach that takes into account three species of *Petroleuciscus*. *Petroleuciscus *turn out to represent a group of unrelated taxa. Thus, *Petroleuciscus borysthenicus *and *P. smyrnaeus *are two closely related species from the Aegean and Black sea basins with relationships to the genera *Ladygesocypris *and *Squalius*. Interestingly, *Leuciscus aphipsi *from the northern Caucasus also belongs to this group, being a morphologically very unspecialized species with still many plesiomorphic characters [82,83]. Although this species was transferred from the genus *Leuciscus *into the genus *Squalius *[2,84], our outcome demonstrates that it is more related to the genus *Petroleuciscus *than to *Squalius *and it is therefore transferred to *Petroleuciscus *here (See table 3). Iranian *Petroleuciscus persidis *was initially described as a species of *Pseudophoxinus *[85], later transferred to the genus *Leuciscus *[44] suspected to be related to *P. smyrnaeus *(former considered as *L. smyrnaeus*) [86] and then included in *Petroleuciscus *[81]. These relationships are inconsistent with our molecular data, which strongly support close affinities of *P. persidis *with the genus *Acanthobrama*, also pointing on the difficulties of morphological characterization of miniaturized cyprinids [23,63]. Therefore, this species is transferred to *Acanthobrama *as *A. persidis *(See table 3). Furthermore, our data support the synonymization of the genus *Acanthalburnus *with *Acanthobrama*, both genera just distinguished by the two *vs*. one rows of pharyngeal teeth [46]. Our results demonstrate also that both species of the highly specialized, predatory genus *Aspius *belong to *Leuciscus*. Therefore, these species are transferred to *Leuciscus *as *L. aspius *and *L. vorax *(See table 3).

The position of the genus *Ladygesocypris *(*L. ghigii *and *L. irideus*) nested in or close to the *Squalius *clade was already reported for mitochondrial data [6,32] and here is also supported by nuclear markers. *Ladygesocypris *was recently included into the genus *Pseudophoxinus *[77], a hypothesis that has not molecular support. However, its transfer to *Squalius *is supported by our data (See table 3).

With regards to *Scardinius*, this genus have been considered the sister group of *Tropidophoxinellus *[5,87] but a close phylogenetic relationship among *Scardinius *and *Alburnus *has also been hypothesized [5,18]. Our phylogeny strongly supported the sister relationship among *Scardinius *and *Tropidophoxinellus *more than among *Scardinius *and *Alburnus*, independently of the North African species *Pseudophoxinus chaignoni *and *P. callensis *belong to the genus *Tropidophoxinellus *or not. Our nuclear phylogeny showed a low support for a relationship among *Scardinius *and *Rutilus*, as suspected already by morphological characters [14]. Whereas mitochondrial cyt *b *analysis grouped together the lineages of *Scardinius, Tropidophoxinellus-Pseudophoxinus callensis-P.chaignoni *(transferred here to *Tropidophoxinellus callensis *and *T. chaignoni*) and *Alburnus-Anaecypris-Leucaspius-Pseudophoxinus punicus*, basal relationships among these lineages were not solved with COxI and nuclear markers, probably due to the slower evolutionary rate of these genes respect to cyt *b *one. However, combined data matrix (all genes) analysis recovered this relationship. Within the genus *Scardinius*, our data support some recognized species [2]. *S. erythrophthalmus *is a Central and Eastern European species, *S. scardafa *endemic to Lake Scanno in Central Italy [62,88] is closely related to *S. hesperidicus*, as has been previously established [62,89]. *Scardinius plotizza *from the Dalmatian River Neretva constituted an independent and highly supported clade.

### Biogeography

#### Divergence time estimates and general biogeographical patterns

To decipher evolutionary and biogeographical patterns in the Mediterranean leuciscins, the different authors have calibrated a molecular clock for the cytochrome *b *gene using fossil or geological data [5,7,20,37,43,90]. Based on fossil calibration, we obtained an evolutionary rate of 0.4% divergence per lineage per million years (0.8% per pairwise comparison) for cytochrome *b*, which differs from previous estimates [5,90], and is slightly slower than formerly proposed for North American cyprinids based on fossil data (0.5% per lineage per million year) [91]. The present study is the first to use nuclear markers to estimate the main historical processes that gave rise to the current phylogeny and distribution of the Mediterranean leuciscine lineages. The average evolutionary rate obtained here was 0.2% per lineage per million years for the combined dataset (cytochrome *b*, COxI, RAG1 and S7) (0.4% per pairwise comparison). Notwithstanding, estimated divergence times were congruent to those predicted by the cytochrome *b *gene (not shown) and fell within the same confidence intervals. However, credibility intervals of the combined dataset (i.e. more informative sites in the analysis) were narrower, suggesting that the combination of several markers may improve the results [92].

The complex paleogeography of the Mediterranean region, with migrating island arcs, fragmenting tectonic belts and other plate tectonic events have contributed to the formation of a reticulate biogeographical pattern in which the current leuciscine groups formed as geographical barriers appeared and disappeared through time. This reticulate pattern is, however, not reflected by a congruent biogeographical pattern in leuciscine relationships based on sister groups within each evolutionary lineage.

#### Origin of leuciscins in Mediterranean inland waters: birth of the Paratethys

**Figure 9:**
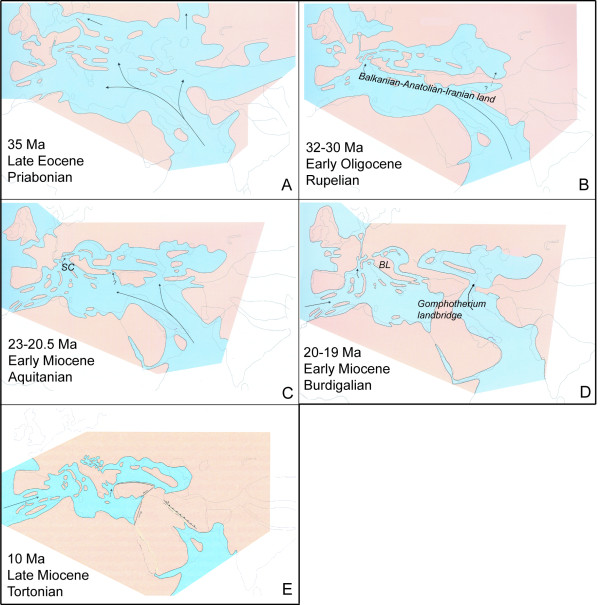
**Map of the main paleogeographic events in Mediterranean region during Late Eocene-Early Miocene periods**. Black arrows point out marine movements. SC: Slovenian corridor. BL: Balkanian landmass.

According to our molecular evolutionary rate based on a relaxed molecular clock and all-gene database, the main Mediterranean leuciscine lineages arose and diversified in the Oligocene period (Figure 8). In the context of the widely accepted origin of cyprinids in Asia, our data do not contradict the north-dispersal model of the first cyprinid colonization of northern Europe across Siberia [34,35], when the Turgai Straits were closed around the Eocene-Oligocene boundary (approximately 34 mya; Figure 9A) and strongly support a gradual colonization of Mediterranean region since Oligocene, as have been recently stated [93]. As the fossil record shows, this first cyprinid colonization event involved phoxinin and gobionin lineages [94,95]. However, according to our data, the presence of old leuciscins in the Balkan/Anatolia area of possible Southwest Asian origin could be explained by the emergence of a huge Balkanian-Anatolian-Iranian landmass in the Early Oligocene (33 mya) [96]. Continental collisions and tectonic movements along the Alpine-Himalayan orogenic belt, which led to the separation of the Paratethys Epicontinental Sea from the Tethys Ocean, drove this event. We therefore propose an initial leuciscine colonization of Europe from southwestern Asia via the Balkanian-Anatolian-Iranian landmass at the beginning of the Early Oligocene (Figure 9B), which precisely matches the initial splitting of the most basal leuciscine lineages (XI, XII and XIII) from the remaining leuciscins in 32.9 mya (CI 95% 29.6-33.5).

Unequivocal fossil proof of this biogeographic hypothesis is hampered by the fact that Oligocene freshwater deposits from the Balkanian-Anatolian-Iranian landmass are scarce and not well studied. The oldest fossils that could be ascribed to the Leuciscinae have been recovered in central Anatolia (Table 4) and have been dated around the Oligocene-Miocene boundary [97,98]. This material consists of isolated pharyngeal teeth and otoliths for which a leuciscine relationship is possible, but not certain.

**Table 4:** First fossil occurrence of Leuciscinae in Europe

FOSSIL TAXON	RECENT RELATIVES	LOCALITY	AGE (Ma)	REFERENCE
Leuciscinae indet.	Leuciscinae	Kilcak (Eskikilcak), central Anatolia	23.5-24.5	[150]

*Palaeoleuciscus dietrichsbergensis*	*Pseudophoxinus*, *Delminichthys*	Dietrichsberg, Rhön mountains, Germany	19	[94,106,107]

*Aspius laubei*	*Aspius aspius*	North Bohemian Browncoal Basin, Czech Republic	18-19	[108,109]

*Alburnus steindachneri*	*Alburnus chalcoides *species group	North Bohemian Browncoal Basin, Czech Republic	18-19	[108,109]

*Leuciscus *sp.	*Leuciscus *s. str.	Sofca, Turkey	11-13	Böhme unpubl., [150]

*Scardinius haueri*	*Scardinius *ssp.	Vösendorf, Austria	10.3	[151]

*Leuciscus *aff. *cephalus*	*Squalius cephalus*	Lava 2, Greece	6.56	Böhme unpubl., [150]

*"Chondrostoma" *sp.	*Achondrostoma, Pseudochondrostoma, Iberochondrostoma, Parachondtrostoma*	Tolosa, Spain	5.33-5.8	Böhme unpubl., [150]

*Scardinius *cf. *erythrophtalmus*	*Scardinius erythrophthalmus*	Ptolemais 1A, Greece	5.31	Böhme unpubl., [150]

*Aspius *sp.	*Aspius aspius*	Ptolemais 1B, Greece	5.29	Böhme unpubl., [150]

*Chondrostoma *sp.	*Chondrostoma *ssp.	Ptolemais 2, Greece	5.21	Böhme unpubl., [150]

*Rutilus *sp.	*Rutilus *ssp.	Tchelopetchene, Sofia Basin, Bulgaria	4-5.3	Böhme unpubl., [150]

#### Vicariant events promoting leuciscine diversification in the Oligocene: Paratethys isolation and reconnection

During most of the Early Oligocene (Solenovian regional stage), the Tethys and Paratethys Seas remained isolated by the Balkanian-Anatolian-Iranian landmass. Marine connections fragmented this landmass around 28 mya into the huge Balkanian and Anatolian islands [96]. This vicariant event was probably responsible for splitting of the ancient Balkan-Anatolian lineages around the Early-Late Oligocene transition (29.1 mya, CI 95% 27.6-31.4). This main Late Oligocene paleogeographic (Figure 9C) setting persisted until the early Burdigalian 20 to 19 mya.

By the end of the Oligocene (23 mya) half of all leuciscin lineages (7 out of 14) (Figure 8) had become established, indicating their rapid diversification during the Late Oligocene. During this time, leuciscine fossils are unknown from the European mainland and we hypothesize that this diversification occurred on the Balkanian-Anatolian-Iranian archipelago. Geodynamic data substantiating vicariant events in the Late Oligocene are rare from this area. For the time being, it may be speculated that karstic landscapes, similar to those found in this region today, could have facilitated the geographical isolation and speciation process.

#### Vicariant events promoting leuciscine diversification in the Early-Middle Miocene: the Alpine Orogeny, Dinarid Lake systems and Gomphotherium landbridge

More recent paleogeographical events support the splitting of some leuciscine groups in the Early-Middle Miocene, from 20 to 12 mya, giving rise to most of the current genera. This period coincides with a major period in the Alpine orogeny, including the closing of the Slovenian corridor, the extrusion and uplift of the Eastern Alps and substantial microplate rotations and basin inversion in the Pannonian-Carpathian-Dinaric realm [99-104].

The Slovenian corridor, a marine connection between the Tethys and Paratethys Seas separated the Balkanian-Anatolian landmass from the rest of Europe during the Oligocene and earliest Miocene. This gateway closed around 20 mya [105] and probably enabled the first colonization of Central Europe by leuciscins from the Balkanian-Anatolian landmass (Figure 9D). This view is supported by fossils like the oldest European leuciscins in sediments from about 19 mya in Germany related to *Pseudophoxinus *and *Delminichthys *[94,106,107] and from 19-18 mya old fossils of *Aspius *and "*Alburnus*" [108,109] in the Czech Republic. These paleontological data fit the start of diversification of leuciscins such as *Aspius *(20.5 mya, CI 95% 17.6-21.3) or *Alburnus *lineages (19.7 mya, CI 95% 16.7-21.9).

The Alpine orogeny similarly affected the leuciscine ichthyofauna of the Mediterranean Peninsulas. Thus, in the Iberian Peninsula, the Pyrenees promoted the isolation of Iberian freshwater fishes during the Miocene period when they took on their current form [110-112]. Indeed, all leuciscins from this region are endemic except *Squalius laietanus*, which also inhabits southern France. Thus, the Iberian *Squalius *must have split from the Balkan and Italian *Squalius *14.6 mya (CI 95% 10.9-14.9). In turn, the endemic genera *Achondrostoma, Iberochondrostoma, Parachondrostoma *and *Pseudochondrostoma *split from the genus *Chondrostoma *9.4 mya (CI 95% 7.9-11.4). The Pyrenees may have also constituted a vicariant barrier between the European and Iberian Alburnine (ancestors of *Anaecypris*, *Alburnus *and/or *Leucaspius*), splitting 12.1 mya (CI 95% 9-14.8). This timing of the origin of Iberian Leuciscinae lineages is congruent with the results of previous studies [43,64].

In the Apennine Peninsula, the Alpine orogeny may have also played an important role in isolating its ichthyofauna. Most of Italy was below sea level during most of the Miocene [113] and it is consequently believed that the Italian ichthyofauna is of a more recent origin than that of the remaining Mediterranean Peninsulas. Although dispersion during the lacustrine phase of the Mediterranean Sea has been suggested as the origin of the Italian ichthyofauna [38], our data point to an older origin, probably related to the lateral tectonic extrusion of the eastern Alps between 17 and 11 mya that isolated Italian taxa from north and central Europe. The role of the eastern Alps as a barrier to Italian and Adriatic freshwater ichthyofauna has been previously established [114,115]. This is the case for *Protochondrostoma *11.5 mya (CI 95% 9.5-13.5), or some *Rutilus*, which according to our mitochondrial data (figure not shown), split 9.7 mya (CI 95% 6.2-10.6) into *R. rubilio *and 13.2 mya (CI 95% 9.4-15.1) into *R. pigus*. This is the only case in which mitochondrial information has been used to disscus date estimation. This can be explained due to we only incorporated a few representatives of each genera to perform all-gene molecular clock analysis and none of these species have been include on all-gene dataset. However, the knowledge of this splitting date for species such as *Protochondrostoma genei *or *Rutilus rubilio and R. pigus *is crucial to show the effect of Alps as a barrier for Italian and Adriatic freshwater icthyofauna.

In the Balkan Peninsula, and especially in the Dalmatian area, huge lake systems existed between 17 and ~15 mya during the late early Miocene and early Middle Miocene (e.g. Dinaric Lake system, Lake Skopje; [116]). The fragmentation or disappearance of these lakes could have enhanced isolation and promoted speciation. Such is the case of *Delminichthys*, which split from *Pelasgus *in approximately 14 mya (CI 95% 11.0- 20.7). This dating is close to previously estimates (13 mya; [63]) and also coincides with the splitting of *Chondrostoma s.l*. from its sister group *Phoxinellus *isolated in Dalmatia 14.6 mya (CI 95% 12.1-16.7). Subsequent to this period of lakes, the region experienced the onset of compressional tectonic stress in the late Serravallian around 12.5 mya, initiating a period of uplift of the Dinaric Alps [117]. This orogenic event could have generated barriers for the Dalmatian *Chondrostoma, Squalius *and *Telestes*, which split approximately 9.7 mya (CI 95% 6.2-11.9), 9.2 mya (CI 95% 5-10.1) and 12.4 mya (CI 95% 11-16.3) respectively.

Another important vicariant event between the Greek and North African *Tropidophoxinellus *took place in the Early Miocene in 17.4 mya (CI 95% 7.9-16.6), indicating colonization of Africa by leuciscins well before the Messinian salinity crisis. This finding rejects the later hypothesis of "Lago Mare dispersal" [38] for the colonization of North Africa by leuciscins, which would explain the colonization of this region due to dispersion during the lacustrine phase of the Mediterranean Sea (Messinian salinity crisis). The most plausible explanation for vicariance between the Peloponessus and Magreb area is the migration of common ancestors of *Tropidophoxinellus *from the Balkanian-Anatolian landmass into North Africa via the *Gomphotherium *landbridge established in West Asia around 19 mya (Figure 9D). This landbridge separated the Tethyan Ocean into the Mediterranean Sea and Indian Ocean and allowed the first continental exchange between Eurasian and African mammals and reptiles [118,119] and the first colonization of Africa by barbin cyprinids ([120]; Böhme unpublished data). Despite fossil leuciscins being as yet unknown from the African Miocene, the data presented here suggest that leuciscins probably took part in this Early Miocene dispersal event.

Of special biogeographic interest is the sister-group relationship between *Notemigonus crysoleucas*, the only North American leuciscine species, and the old Balkanian-Anatolian *Pseudophoxinus egridiri/Pelasgus/Delminichthys *lineages, which separated according to our data around 20.3 mya (Figure 8). However this relationship has to be considered with caution due to its relatively low support (posterior probability equal to 86). As stated above, a possible sister taxon of the *Pseudophoxinus egridiri/Pelasgus/Delminichthys *lineages, the fossil genus *Palaeoleuciscus*, made its oldest fossil appearance in 19 mya old sediments of central Europe, indicating a transatlantic biogeographic connection. Interestingly, an Early Miocene transatlantic colonization of North America by cyprinids was already speculated by Böhme [94] in the context of the paleobiogeography of phoxinins. This author demonstrates a sister-group relationship between the central European Oligocene phoxinin *Palaeorutilus *and the eastern North American Creek Chub clade of phoxinins. The data presented here further support such an idea, and may indicate that eastern North America was colonized during Early Miocene times via a transatlantic route by the ancestors of the leuciscin *Notemigonus *(a species related to *Palaeoleuciscus*) and the Creek Chubs (species related to *Palaeorutilus*). The paleogeographic details of this hypothesis, however, remain obscure.

#### Vicariant events promoting leuciscine diversification in the Upper Miocene

In this period, the opening of the Aegean Sea was an important event for leuciscins (Figure 9E), which is thought to have started in the Late Serravallian about 12 mya [101] and finished during the Tortonian between 10 and 9 mya [121-123]. As a consequence, some groups of Greek and Anatolian leuciscins diversified, such as *Squalius ghigii/irideus *from Rhodos Island and Southwestern Anatolia and *Squalius keadicus *from southern Greece. These taxa split 8.7 mya (CI 95% 7-13.2). In fact, the paleogeographical history of the Balkan Peninsula is strongly linked to the paleogeography of Anatolia since both areas were joined in the same landmass and isolated from the rest of Europe during long periods of the Oligocene and Miocene.

The North African *Pseudophoxinus punicus *splits near the Middle-Late Miocene boundary; also well before the Messinian salinity crisis. Two possible vicariant barriers could explain its distribution: the opening of the Gibraltar Straits (splitting of Iberian *Anaecypris*), which has been recognized as a geographical barrier for some groups [124], or the opening of the Channel of Sicily (splitting of European *Alburnus *or *Leucaspius*). However, both the Gibraltar Straits and the Sicily Channel formed at the Miocene/Pliocene boundary at the end of the Messinian salinity crisis [40,125] and our estimated divergence time for *P. punicus *is earlier than this date (10.9 mya, CI 95% 6.2-12.2). The vicariant event leading to the separation of *Pseudophoxinus punicus *therefore remains unclear.

#### The Messinian

The Messinian has been postulated as the time of diversification of Mediterranean cyprinids. Hence, the Lago Mare stage of the Mediterranean would have enabled the massive dispersion of cyprinids across the basin. The later return to marine conditions could have meant a fast speciation process for cyprinids. This may be reflected in the deep polytomies found in the phylogeny. Thus, some authors have defended the Lago Mare hypothesis [38] based on non-resolved polytomies among the main clades [7,42,54]. However, our phylogeny does not show a critical point of speciation around 5 mya. These processes took place gradually over time, but preclude any massive dispersal and speciation during the Messinian period, as has been proven for *Squalius *and *Chondrostoma s.l*. [37,43,64]. Fossils of the Spanish genera (*Achondrostoma, Iberochondrostoma, Parachondrostoma, Pseudochondrostoma*) are recorded first in the late Messinian (Table 4), which may indicate dispersal of their ancestors during the Messinian Lago Mare stage.

#### Pliocene and Pleistocene

The beginning of the Pliocene is characterized by a rise in humidity in the Mediterranean region [126], which may have promoted the diversification of Leuciscinae genera in different Mediterranean Peninsulas, through the possible colonization of newly established freshwater environments after the Messinian salinity crisis. In the Iberian Peninsula, tectonic disconnections of present-day Iberian basins took place during the Plio-Pleistocene [127] promoting widespread endorrheism and thus enhancing the process of allopatric speciation, which is reflected in the high species-levels of most Iberian genera except the monotypic genus *Anaecypris*. In turn, the divergence of some Italian taxa from their Balkanian sister-taxa occurred in this period, such as the splitting of *Rutilus aula *(4.75 mya, CI 95% 1.91-5.02), and *Squalius lucumonis *(3.53 mya, CI 95% 2.13-5.80).

In the Adriatic-Balkanian region, even more recent events explain the great affinities found between the ichthyofauna of northern Italy and northern rivers of the Balkan Peninsula. These similarities reflect recent contact between North Italian and North Adriatic-Balkanian ichthyofauna due to expansion of the Po River during the Last Glacial Maximum as a result of a drop in sea level [128]. This event may have prompted the exchange of many freshwater taxa among Italian and Balkan river systems, and also explains the current distribution of species such as *Squalius squalus *and similarities found between *Chondrostoma soetta *and the Dalmatian species *Chondrostoma knerii *and *C. phoxinus*. Similar affinities have been described for freshwater fish species from northern Italy and Balkan rivers including *Cobitis *[129] or *Cottus *[130] species.

On the other hand, Pleistocene glaciations also played an important role in the current European distribution of the Leuciscinae, and determined a more recent origin of some leuciscine taxa after colonization from glacial refuges such as the Danube basin. However, other rivers of the Black Sea basin could have also acted as a glacial refuge for freshwater ichthyofauna [51]. Some north-central European representatives of leuciscins share a widespread distribution range: *Alburnoides bipunctatus*, *Alburnus alburnus*, *Chondrostoma nasus, Rutilus rutilus, Scardinius erythrophthalmus *and *Squalius cephalus*. This pattern can be explained by the homogeneity conferred by glacial refuges [35,131-133]. Thus, secondary recolonization of Western Europe from a Danubian refuge has been postulated for *S. cephalus *[131] and *C. nasus *[34,134]. River captures, river confluences and sea level lowering have been incriminated in the dispersion of these species [131]. Thus, glacial periods promoted the expansion and genetic homogenization of these species across Europe [48,131,135]. Since most central European basins were covered by ice during the Pleistocene, colonization is the most likely mechanism to explain the expansion of these species. However, they could not have reached the Mediterranean Peninsulas because of the transverse alignment of Alpine Mountains, which were well formed in the Pleistocene except in the eastern part of the Balkan Peninsula. This area shows the influence of the Danube basin due to the oblique direction of the Dinaric Alps.

## Conclusions

Mitochondrial and nuclear results demonstrated the existence of fourteen major phylogenetic leuciscine lineages. However, some incongruence was found between mitochondrial and nuclear markers, possibly due to the lack of resolution of deep nodes in nuclear phylogeny as well as their slower evolutionary rate. Combined analysis (mitochondrial + nuclear) recovered the major lineages and increased their support.

With regards to phylogenetic relationships, this study is the first molecular approximation to the phylogenetic placement of North African leuciscine species (*P. callensis*, *P. chaignoni*, and *P. punicus*). None of these three species belong to the real genus *Pseudophoxinus*, as well as *P. egridiri*, and constitute independent lineages. The real genus *Pseudophoxinus *includes species from Anatolia and Levant. Our phylogeny also demonstrates that the genus *Petroleuciscus *is polyphyletic and as a result its species involve different leuciscine lineages. It also point out the closer relationship of *Squalius aphipsi *to *Petroleuciscus*. As our phylogeny show, the taxonomy of the genera *Pseudophoxinus *and *Petroleuciscus *is a matter of controversy because of synapomorphic characters are prone to be overshadowed by morphological changes associated to miniaturization. New insights in the phylogenetic relationships of some *Squalius *species are showed too, such as some Dalmatian species, as well as the corroboration by nuclear markers of the phylogenetic position of *Ladigesocypris ghigii/irideus *as closer related to the genus *Squalius*.

In relation to biogeographical history of Leuciscinae, the present study is the first to use nuclear markers to estimate main historical processes that gave rise to the current phylogeny and distribution of Circum-Mediterranean Leuciscinae. A relaxed molecular clock corroborated the arisen and divergence of the main Leuciscinae lineages during Late Oligocene-Early Miocene. Our data do not contradict the north-dispersal model of the first cyprinid colonization of Northern Europe across Siberia. However, most of the divergence events were older than Lago Mare dispersal model. We proposed an initial colonization of Europe from Southwestern Asia via the Balkanian/Anatolian/Iranian landmass at the beginning of the Early Oligocene, which precisely matches the initial splitting of the most basal leuciscins. Later vicariant events as the Paratethys isolation and later reconnection with Tethys during the Oligocene and the Alpine Orogeny, Dinarid lake systems and Gomphoterium landbridges during Miocene promoted Leuciscinae diversification. Our data also corroborate the colonization of North Africa before the Messinian salinity crisis. In Upper Miocene the opening of Aegean Sea was an important vicariant event for Anatolian and Greek leuciscins. Messinian appears as a stage of gradually Leuciscinae diversification more than a critical point of speciation. The rise of humidity at the beginning of the Pliocene may have promoted the diversification of Leuciscinae genera due to the colonization of newly established freshwater environments. Finally, Pleistocene glaciations also played an important role in the current European distribution of some leuciscins.

## Methods

### Sampling, DNA extraction, PCR and sequencing

**Figure 10:**
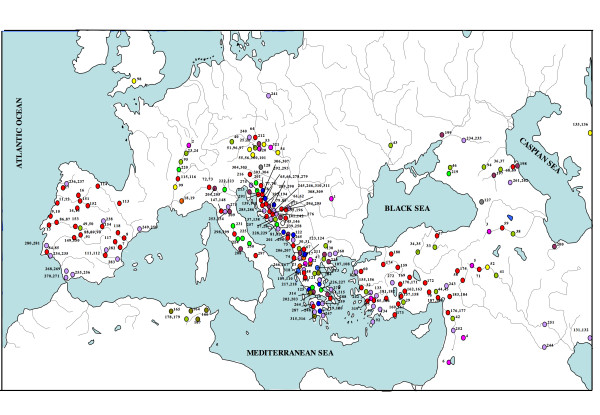
**Map of localities samples of the leuciscine representatives included in the phylogenetic framework**. Numbers correspond to those indicated in the Additional file 1.

The complete mitochondrial cytochrome *b *(total of 1140 bp) from 321 specimens belonging to 176 species of leusciscins was obtained from Europe, Western Asia, North Africa and North America (Figure 10). Of total individuals, 186 were new sequences for cytochrome *b *and the remaining sequences were acquired from Genbank. Similarly, 146 individuals were sequenced to obtain a fragment of the mitochondrial Cytochrome oxidase I gene (646 bp). For the nuclear phylogenetic analysis, a subset of 101 individuals was selected from mtDNA lineages and sequenced for both the nuclear genes RAG1 (1473 bp) and the first intron of the ribosomal protein S7 (1112 bp total alignment including gaps). DNA voucher specimens were deposited at the collection of the Museo Nacional de Ciencias Naturales (Madrid, Spain). List of individuals, localities and GenBank Accession numbers are reported in Additional file 1.

For phylogenetic analyses, we rooted the trees with representatives of several cyprinids subfamilies. For this purpose, species such as *Gobio gobio *(*Gobioninae*) *Rhodeus amarus, R. ocellatus *and *R. atremius *(Acheilognatinae), *Tinca tinca *(Tincinae), and *Pseudorasbora parva *(*Gobioninae*) were used as outgroups in phylogenetic analyses.

Total cellular DNA was extracted from both ethanol preserved or frozen tissue by a standard proteinase K and phenol/chloroform extraction method [136] and ethanol purification [137]. Both mitochondrial (cytochrome *b *and COxI) and nuclear (RAG1 and S7) genes were amplified via polymerase chain reaction (PCR) from each individual DNA sample. Primers, amplification protocols and PCR products lengths for these loci are represented in Additional file 2. In all cases, PCR mixtures were prepared under similar conditions in a final volume of 25 μl containing 1-2 μl DNA, 0.5 μM each primer, 0.2 mM each dNTP, 1.5 mM MgCl_2_, and 1 unit of Taq DNA polymerase (Invitrogen). After checking PCR products on 1.5% agarose gels, the four genetic fragments were purified with the kit ExoSAP-IT™(USB) and directly sequenced. For RAG1 some internal primers were designed to sequencing (See Additional file 2). All samples were sequenced on an Applied Biosystems 3700 DNA sequencer following the manufacturer's instructions. All new are available online [Genbank: HM560056-HM560237 for cyt *b*; HM560238-HM560383 and HM989722-HM989724 for COxI; HM560384-HM560471, HM560573-HM560586 and HM998711-HM998712 for RAG1; HM560472-HM560572 and HM998713-HM998714 for S7].

### Sequences alignment and Phylogenetic Analyses

Homologous regions were aligned manually against previously published cytochrome *b *sequences of leuciscins [4,5]. Chromatograms and alignments were visually checked and verified and there were no gaps in the resulting cyt *b*, COxI and RAG1. In these three coding genes sequences alignment was based on the inferred amino acid sequence. Alignments sequences of all performed analyses are proporcioned in Additional file 3. All codon posititions were included in the analysis. The first intron of S7 gene were aligned with Clustal X [138], using default parameters, to optimize sequence alignment including gaps and aligned sequences were later checked by eye. Despite of indels are often considered as a class of phylogenetic characters to be incorporate in the phylogenetic analysis [139,140], gaps of S7 gene was discarded in phylogenetic reconstructions due to their ambiguity in the alignment.

For all data sets, the transition (ti)/transversion (tv) rate was estimated using a maximun-likelihood approach (Table 1). Furthermore, for each gene, the saturation of transition and transversion changes was checked by plotting the absolute number of changes of each codon position against patristic distances (p). There was no evidence of saturation for any data set of sequences, even in the third position of coding genes.

Analyses were performed independently on each gene (cyt *b*, COxI, RAG1 and S7), in nuclear data sets and on the total number of base pairs sequenced (4339 bp) ("Total evidence", [141]). Nucleotide composition was examined and the χ^2 ^homogeneity test of base frequencies was carried out in Paup *4.0b10 [142] for all genes. The Akaike Information Criterion implemented in ModelTest v. 3.7 [67] was used to determine the evolutionary model that best fits the data set for each data set. The model selected was used for subsequent analyses. Bayesian inference (BI) was performed with MrBayes 3.1.2 [143]. In the combined data set each gene partition was allowed to follow its own model of evolution. In all cases, BI was obtained by simulating two simultaneous Markov chain analyses (MCMC) for 3.000.000 generations each, to estimate the posterior probabilities distribution. Topologies were sampled every 100 generations and a majority-rule consensus tree was estimated after eliminated the first 10^5 ^generations in each analysis. Maximum Likelihood (ML) analysis was performed with PhyML package [144]. To estimate the robustness of the likelihood analyses a nonparametric bootstrap test was conducted with 500 replicates. Maximum Parsimony (MP) [145] analysis was performed with the package PAUP* 4.0b10 [142]. MP analysis was conducted with TBR branch swapping and 10 random stepwise additions using the heuristic search algorithm. Analyses were performed on each independent data set and on the total data matrix after check homogeneity among partitions [146]. Confidences for this analysis were estimated by bootstrapping (500 repetitions) [147].

### Molecular clock and divergence times estimates

As a general clock-like behaviour was rejected, divergence times and their credibility intervals (highest posterior density: HPD) were estimated using a relaxed clock model in BEAST v1.4.7 [148], with branch rates drawn from an uncorrelated lognormal distribution [149] and a Yule speciation prior. Fossil evidence was used to place the constraints of the age of different nodes within the topology. The use of multiple calibration points would provide a more realistic divergence time estimates. The calibration points used to estimate divergence times are represented in Table 5. Tracer v1.4 [148] was used to plot the log-likelihood scores against generation time to evaluate run convergence and the burn-in needed before to reconstruct the 50% majority-rule consensus tree of the post burn-in trees. To obtain a maximum clade credibility tree, trees were summarized with the software TreeAnnotator 1.4.6 [148].

**Table 5:** Fossil calibration points

Clade	Mya
*Squalius *cephalus clade	6.5
*Squalius *lineage	13
*Achondrostoma*/*Pseudochondrostoma *clades	5.3
*Scardinius *clade	10
*Scardinius *erythrophthalmus clade	5.3
*Leuciscus leuciscus*/*leuciscus idus *clade	5.3
Miminun Recent Common Ancestor of all Iberian taxa	25
Abramidini clade	17
Split among *Phoxinus *and leuciscine groups	33

## Authors' contributions

SP conceived the study, collected samples, obtained data and analyses and drafted the manuscript. MB contributed to review and support the biogeographical chapter with paleontological and fossil data. PZ, JF and RS collected samples, reviewed the manuscript and make useful suggestions for it. MO and AA collected valuables samples for the study. ID participated in its design and coordination, helped to draft the manuscript and collected samples. All authors read and approved the final manuscript.

## Supplementary Material

Additional file 1 **Individuals included in the phylogeny and localities samples**. Click here for file

Additional file 2 **Laboratory performance (PCR conditions and primers)**. Click here for file

Additional file 3 **Sequences alignments used in phylogenetic performance and molecular clock analyses**. Click here for file
