# Supplementary material for: Phylogenetic relationships and biogeographical patterns in Circum-Mediterranean subfamily Leuciscinae (Teleostei, Cyprinidae) inferred from both mitochondrial and nuclear data
Source: BMC Evol Biol. 2010 Aug 31;10:265. doi: 10.1186/1471-2148-10-265 (PMC2940817; doi:10.1186/1471-2148-10-265)
Supplement: Additional file 2 — Laboratory performance (PCR conditions and primers). [file 1471-2148-10-265-S2.DOC]

**Additional File 2. PCR conditions and primers for each amplified gene and PCR product length.**

| **Gene** | **Primers**  **(Source)** | **PCR conditions** | **Gene length (bp)** |
| --- | --- | --- | --- |
| **Cytochrome *b* (cytb)** | GluF:  5’AACCACCGTTGTATTCAACTACAA3’  ThrR:  5’ACCTCCGATCTTCGGATTACAAGACCG3’  (Machordom and Doadrio, 2001) | Initial denaturation: (94ºC, 5 min.)  35 cycles:  denaturation (94ºC, 1 min.)  annealing (50ºC, 1.15 min.)  extension (72º, 1.30 min.)  Final extension: (72ºC, 10 min.) | 1140 |
| **Cytochrome Oxidase I (COI)** | FISH1F: 5’TCAACCAACCACAAAGACATTGGCAC3’  FISH1R: 5’TAGACTTCTGGGTGGCCAAAGAATCA3’  (Ward et al., 2005) | Initial denaturation: (95ºC, 2 min.)  30 cycles of denaturation (94ºC, 1 min.), annealing (54ºC, 1 min.) and extension (72º, 1.30 min.)  Final extension: (72ºC, 10 min.) |  |
| **Recombinant activating gene 1 (RAG1)** | RAG1F:  5’AGCTGTAGTCAGTAYCACAARATG3’  RAG9R:  5’GTGTAGAGCCAGTGRTGYTT3’  Internal primers to sequencing:  RAG3F:  5’GGGTGtgtcagygagaagca3’  RAG5R:  5’CGCCACACAGGYTTCATCT3’  (Quenouille et al., 2004) | Touchdown pcr:  Initial denaturation: (95ºC, 3 min.);  10 cycles of denaturation (95ºC, 45 seg.), annealing (58ºC, 1.15 min.) and extension (72º, 1.45 min.), with reduction of 0.5ºC each annealing cycle (58ºC-53ºC);  25 cycles of denaturation (95ºC, 45 seg.), annealing (53ºC, 1.15 min.) and extension (72º, 1.45 min.)  Final extension: (72ºC, 10 min.) | 1473 |
| **Protein Ribosomal S7 (first intron)** | S7RPEX1F:  5’TGGCCTCTTCCTTGGCCGTC3’  S7RPEX2R:  5’AACTCGTCTGGCTTTTCGCC3’  (Chow and Hazama, 1998) | Initial denaturation: (95ºC, 5 min.)  40 cycles of denaturation (94ºC, 1 min.), annealing (54ºC, 1.30 min.) and extension (72º, 2 min.)  Final extension: (72ºC, 10 min.) | 1112 (total alignment including gaps) |

**References:**

1.- Machordom A, Doadrio I: **Evidence of a Cenozoic Betic-Kabilian connection based on freshwater fish phylogeography (*Luciobarbus, Cyprinidae)****. Mol Phylogenet Evol* 2001, **18**(2): 252-263.

2.- Ward DR, Zemlak TS, Innes BH, Last PR, Hebert PDN: **DNA barcoding Australia’s fish species**. *Phil Trans R Soc B* 2005. doi:10.1098/rstb.2005.1716.

3.- Quenouille B, Bermingham E, Planes S: **Molecular systematics of the damselfishes (Teleostei: Pomacentridae): Bayesian phylogenetic analyses of mitochondrial and nuclear DNA sequences.** *Mol phylogenet Evol* 2004, **31**: 66-88.

4.- Chow S, Hazama K: **Universal primer fo S7 ribosomal protein gene intron in fish.** *Mol Ecol* 1998, **7**:1255-1256.
