# Supplementary material for: Phylogenetic relationships and biogeographical patterns in Circum-Mediterranean subfamily Leuciscinae (Teleostei, Cyprinidae) inferred from both mitochondrial and nuclear data
Source: BMC Evol Biol. 2010 Aug 31;10:265. doi: 10.1186/1471-2148-10-265 (PMC2940817; doi:10.1186/1471-2148-10-265)
Supplement: Additional file 3 — Sequences alignments used in phylogenetic performance and molecular clock analyses. [file 1471-2148-10-265-S3.RTF]

1.- Cytochrome b (cytb) alignment used in phylogenetic and mitochondrial molecular clock analyses #NEXUS Begin data;Dimensions ntax=321 nchar=1140;Format datatype=dna gap=-;MatrixEF137863Abramisbrama                    atggcaagcctacgaaaaacccacccactaataaaaatcgctaatgacgcactagtcgacctcccaacaccatctaacatttccacactatgaaacttcggatccctcctaggattatgtttaattacccaaatcctcacgggattatttctagccatacactacacctctgatatctccaccgcattttcatcagtaacccacatctgccgagacgttaactacggctgacttattcgaaacttacatgctaatggagcatcattcttctttatctgcctttatatacatattgcacgaggcctatactacgggtcatatctttacaaagaaacctgaaatattggcgtagtcctatttcttctagttataataacagccttcgtcggctacgtacttccatgaggacaaatgtccttttgaggtgccaccgtaattacaaatctcctctcagcagtcccttatataggagacactcttgtccaatgaatctgaggcggcttttcagtagacaacgcaactctcacacgattcttcgcattccacttcctcctgccattcgttgtagccggcgcaaccctcctacacctactatttctacacgaaacgggatcaaataacccgaccggattaaactccgacgcagataaaatttctttccacccatacttctcatataaagaccttcttggctttgtaatcatgttactagccctcacttcgctagcattattttcacctaatctactaggtgacccagaaaattttaccccagcaaacccactcgtgacacccccgcatattcagccagaatgatacttcttatttgcctacgctattctccgatccattccaaataaattaggaggggttcttgcattattattcagtattttagtgctaatagttgtgccaatcttacatacctcaaagcaacgaggactaactttccgtcctataacacaattcttattctgaaccctagttgcagacatggtcattctaacatgaattggaggcatacccgtagaacacccatatattattattggccaagtcgcatccattctatactttgcactcttcctcattcttattccactagcagggtgaatggaaaacaaagcattgaaatgagccY10441Abramisbrama                      atggcaagcctacgaaaaacccacccactaataaaaatcgctaatgacgcactagtcgacctcccaacaccatctaacatttcaacactatgaaacttcggatccctcctaggattatgtttaattacccaaatcctcacgggattatttctagccatacactacacctctgatatctccaccgcattttcatcagtaacccacatctgccgagacgttaactacggctgacttattcgaaacttacatgctaatggagcatcattcttctttatctgcctttatatacatattgcacgaggcctatactatgggtcatatctttacaaagaaacctgaaatattggcgtagtcctatttcttctagttataataacagccttcgtcggctacgtacttccatgaggacaaatgtccttttgaggtgccaccgtaattacaaatctcctctcagcagtcccttatataggagacactcttgtccaatgaatctgaggcggcttttcagtagacaacgcaactctcacacgattcttcgcattccacttcctcctgccattcgttgtagccggcgcaaccctcctacacctactattcctacacgaaacgggatcaaataacccgaccggattaaactccgacgcagataaaatttctttccacccatacttctcatacaaagaccttcttggctttgtaatcatgttactagccctcacttcgctagcattattttcacctaatctactaggtgacccagaaaaatttaccccagcaaacccactcgtgacacccccacatattcagccagaatgatactttctatttgcctacgctattctccgatccattccaaataaattaggaggggttcttgcattattattcagtattttagtgctaatagttgtgccaatcttacatacctcaaaacaacgaggactaactttccgtcctataacacaattcttattctgaaccctagttgcagacatggtcattctaacatgaattggaggcatacccgtagaacacccatatattattattggccaagtcgcatccattctatactttgcactcttcctcattcttattccactagcagggtgaatggaaaacaaagcattgaaatgagccAY026407Acanthobramamicrolepis          atggcaagcctacgaaaaacccacccactaataaaaatcgctaatgacgcgctagtcgaccttccaacaccatctaacatttcagtaatgtgaaactttgggtccctcctaggattatgtttaattacccaaatcctcacaggattattcctagccatacattacacctctgatatttccaccgcgttttcatcggtaacccacatttgccgagacgttaactatggctgactcattcgaagtttgcacgctaacggagcatcattcttcttcatctgtctttacatacacattgcacgaggcctgtactacggatcatatctttacaaagaaacctgaaatattggcgtagtcctgtttcttctagttataatgacagccttcgtcggctatgtacttccatgggggcagatatctttttgaggtgctaccgtaattacaaacctactttcagcagtcccttatataggcgacacccttgtacaatgaatctgaggcggtttctcagtagataacgcaaccctcacacggtttttcgcgttccacttcctcctaccattcgttgtcgccggcgcaaccctcatacacctgctatttctacacgaaacaggatcaaacaacccggccggcttaaactccgacgcggataaaatttctttccacccgtacttctcgtataaataccttcttggctttgtagtcatgttactagcccttacttctctggcattattttcacctaacctattaggtgacccagagaatttaaccccagcaaacccaatcgtgacacccccacatattcagccagaatgatatttcttatttggctacgccattctccgatccattccaaataaactaggaggggtccttgcattactattcagtattttagtgctaatagttgtgccaatcttacatacctcaaaacaacgaggactaactttccgccctataacacagttcttattttgaaccctggttgcagatataatcattttgacatgaattgggggcatacccgtagagcacccatatattattattggccaaatcgcatccgttttatattttgcactcttcctcatccttgtcccactagcaggatgggtagagaacaaagcactgaaatgagcc  HM560056Acanthobramalissneri            ATGGCAAGCCTACGAAAAACCCACCCACTAATAAAAATCGCTAATAGCGCGCTAGTCGACCTTCCAACACCGTCTAACATTTCAGCAATGTGAAACTTCGGGTCGCTTCTAGGATTATGTTTAATTACCCAAATCCTCACAGGACTATTTCTAGCCATACATTACACCTCCGATATTTCCACCGCATTTTCATCAGTAACCCACATTTGCCGAGACGTGAACTACGGCTGGCTTATCCGGAACCTACACGCTAACGGAGCGTCATTCTTCTTCATCTGTCTTTATATACACATTGCACGAGGCCTTTACTACGGATCATATCTTTACAAGGAGACCTGAAATATTGGCGTAGTCCTATTTCTTCTAGTTATGATAACGGCCTTTGTAGGCTATGTACTTCCATGAGGACAGATATCTTTTTGAGGTGCCACCGTAATTACAAACCTTCTCTCGGCAGTCCCTTACATGGGCGACACCCTTGTACAATGGATCTGAGGGGGCTTCTCAGTAGATAACGCAACTCTCACACGGTTCTTCGCATTCCACTTCCTTCTACCATTCGTTGTCGCCGGCGCAACCCTCCTACACCTGCTATTTCTACACGAAACAGGGTCGAACAACCCAGCCGGCTTAAACTCCGACGCAGACAAAATTTCTTTCCACCCGTACTTCTCGTACAAAGATCTCCTTGGCTTTGTAGTAATACTACTAGCCCTTACTTCCCTAGCATTATTCTCCCCTAACCTATTAGGTGATCCAGAAAATTTCACCCCAGCAAACCCACTCGTGACACCCCCACATATTCAGCCAGAATGATATTTCTTATTTGCTTACGCCATTCTCCGATCCATTCCAAATAAACTAGGAGGGGTTCTTGCATTACTATTCAGTATTTTAGTGCTAATAGTTGTGCCAATTTTACATACCTCAAAACAACGAGGACTAACTTTCCGCCCTATGACACAGTTTTTATTTTGAACCTTAGTTGCAGATATAATCATTTTGACATGAATCGGCGGCATACCCGTAGAGCACCCATATATTATTATTGGCCAAATCGCATCCATTTTATATTTTGCACTCTTCCTCGTTCTTGTCCCGCTAGCAGGATGAGTGGAGAACAAAGCACTGAAATGAGCCAY026411Acanthobramamarmid              atggcaagcctacgaaaaacccacccactaataaaaatcgctaatgacgcgctagtcgaccttccaacaccatctaacatttcagtaatatgaaacttcgggtccctcctaggattatgcttaattacccaaatcctcacgggagtatttctagctatacattacacctctgatatttccaccgcgttttcatcagtaacccatatttgccgagacgttaactatggttgactcattcgaagtttacacgctaacggagcatcgttcttctttatctgcctttatatacacattgcacgaggcctatactacggatcatatctttacaaagagacctgaaatattggcgtagtcctatttcttctagttataatgacagccttcgtcggctatgtacttccatggggacagatatctttttgaggtgccaccgtaattacaaacctcctctcagcagtcccttacatgggcgacaccctcgtacagtgaatctgaggcggcttctcagtagataacgcaactctcacacggttcttcgcgttccacttcctcctaccattcgttgtcaccggcgcaaccctcttacacctactatttctactcgaaacagggtcaaacaacccagccgggttaaactccgacgcggataaaatttctttccacccgtacttctcatataaagaccttcttggctttgtaatcatgttactagcccttacttctctggcattattttcccctaacctattaggtgacccagagaattttaccccagcaaacccactcgtgacacccccacatattcagccagaatgatatttcttgtttgcttacgccattctccgatccattccaaataaactaggaggggtcctcgcattactatttagtattttagtgctaatggttgtgccaatcttacatacctcaaaacaacgaggactaactttccgccccgtaacacaattcttattttgaaccttagttgcagacatagttattttaacatgaatcggaggcatacccgtagaacacccatatattgttattggccaagtcgcatccattttatattttgcgctcttcctcattcttgtcccgctagcaggatgagtagagaacaaagcactgaaatgagccHM560112Acanthobramapersidis            ATGGCAAGCCTACGAAAAACCCACCCACTAATAAAAATCGCTAATGACGCGCTAGTCGACCTTCCAACACCATCTAATATTTCAGTAATATGAAACTTCGGATCCCTCCTAGGGTTATGTTTAATTACTCAAATCCTCACGGGATTATTTCTAGCTATACATTATACCTCTGATATTTCCACCGCGTTTTCATCAGTAACCCACATTTGCCGAGACGTTAATTATGGCTGGCTCATTCGAAGCTTACACGCTAACGGAGCATCGTTCTTCTTCATCTGTCTTTACATACACATTGCACGGGGCCTATACTACGGATCATATCTTTACAAGGAAACCTGAAACATTGGTGTAGTCCTATTCCTTCTAGTTATAATGACAGCCTTCGTCGGCTATGTACTTCCATGAGGGCAGATATCTTTTTGAGGTGCCACCGTAATTACAAATCTCCTCTCAGCAGTTCCTTATATAGGCGACACCCTTGTGCAATGGATCTGAGGCGGCTTCTCAGTAGATAACGCAACTCTCACGCGGTTCTTCGCATTCCACTTCCTTCTACCATTCGTTGTTACCGGCGCAACCTTCTTACACCTACTATTTCTACACGAAACAGGGTCAAACAACCCGGCCGGCTTAAACTCCGACGCAGATAAAATTTCTTTCCATCCATACTTCTCATACAAAGACCTTCTTGGCTTTGTGGTCATGTTACTAGCCCTTACTTCTCTAGCATTATTTTCCCCTAACCTGTTAGGTGACCCAGAGAACTTTACCCCAGCGAACCCACTCGTAACGCCCCCACATATTCAGCCAGAATGATATTTCTTATTTGCTTACGCCATTCTCCGATCCATTCCAAATAAACTAGGAGGGGTCCTCGCACTACTGTTTAGTATTCTAGTGCTAATAGTTGTGCCGATCTTACATACCTCAAAACAACGAGGACTAACTTTCCGCCCTATAACGCAATTCTTATTTTGAACCTTAATTGCAGACATAATCATTTTGACATGAATCGGAGGCATACCCGTGGAACATCCATATATTATTATTGGCCAAGTCGCATCCATTTTATATTTTGCACTCTTCCTCATTCTTGTTCCACTAGCAGGATGAGTAGAGAACAAAGCGCTGAAATGAGCCHM560113Acanthobramapersidis            ATGGCAAGCCTACGAAAAACCCACCCACTAATAAAAATCGCTAATGACGCGCTAGTCGACCTTCCAACACCATCTAATATTTCAGTGATATGAAACTTCGGATCCCTCCTAGGGTTATGTTTAATTACTCAAATCCTCACGGGATTATTTCTAGCTATACATTATACCTCTGATATTTCCACCGCGTTTTCATCAGTAACCCACATTTGCCGAGACGTTAATTATGGCTGGCTCATTCGAAGCTTACACGCTAACGGAGCATCGTTCTTCTTCATCTGTCTTTACATACACATTGCACGGGGCCTATACTACGGATCATATCTCTACAAGGAAACCTGAAACATTGGCGTAGTCCTATTCCTTCTAGTTATAATGACAGCCTTCGTCGGCTATGTACTTCCATGAGGGCAGATATCTTTTTGAGGTGCCACCGTAATTACAAATCTCCTCTCAGCAGTTCCTTATATAGGCGACACCCTTGTGCAATGGATCTGAGGCGGCTTCTCAGTAGATAACGCAACTCTCACGCGGTTCTTCGCATTCCACTTCCTTCTACCATTCGTTGTTACCGGCGCAACCTTCTTGCACCTACTATTTCTACACGAAACAGGATCAAACAACCCGGCCGGCTTAAACTCCGACGCAGATAAAATTTCTTTCCATCCATACTTCTCATACAAAGACCTTCTTGGCTTTGTGGTCATGTTACTAGCCCTTACTTCTCTAGCATTATTTTCCCCTAACCTGTTAGGTGACCCAGAGAACTTTACCCCAGCGAACCCACTCGTAACGCCCCCACATATTCAGCCAGAATGATATTTCTTATTTGCTTACGCCATTCTCCGATCCATTCCAAATAAACTAGGAGGGGTCCTCGCACTACTGTTTAGTATTCTAGTGCTAATAGTTGTGCCGATCTTACATACCTCAAAACAACGAGGACTAACTTTCCGCCCTATAACGCAATTCTTATTTTGAACCTTAGTTGCAGACATAATCATTTTGACATGAATCGGAGGCATACCCGTGGAACATCCATATATTATTATTGGCCAAGTCGCATCCATTTTATATTTTGCACTCTTCCTCATTCTTGTTCCACTAGCAGGATGAGTAGAGAACAAAGCGCTGAAATGAGCCAY254654Achondrostomaarcasii            ATGGCAAGCCTACGAAAAACTCACCCGCTAATAAAAATCGCTAGCGACGCGCTAGTCGACCTCCCAACACCATCTAATATTTCAGTAATGTGAAACTTCGGATCACTCCTAGGATTATGTTTAATTACACAAATTCTAACAGGATTATTCTTAGCCATACATTACACCTCCGATATCTCAACCGCATTCTCATCGGTAACCCACATCTGCCGAGACGTTAACTACGGCTGACTTATCCGAAGCCTACATGCCAATGGAGCATCCTTTTTCTTTATCTGTCTTTACATACATATTGCACGAGGCCTATATTATGGATCATATCTTTATAAAGAAACCTGAACCATTGGCGTAGTCCTATTCCTTTTAGTTATAATAACAGCCTTTGTCGGCTACGTTCTTCCGTGGGGACAAATATCCTTTTGAGGTGCTACTGTGATTACAAACCTTCTATCTGCAGTCCCATACATAGGAGATACCCTCGTTCAATGAATCTGGGGTGGGTTTTCAGTAGACAATGCAACTCTCACCCGATTCTTCGCATTCCACTTCCTACTACCATTTGTCGTTGCCGGCGCAACCATCCTACACTTATTATTTCTACACGAAACCGGATCGAACAACCCGGCCGGATTAAATTCCGACGCAGACAAGATTTCTTTCCACCCATATTTTTCATATAAAGACCTTCTTGGCTTTGTGGCCATGTTACTAGCCCTTACCTCCTTAACGTTATTTTCCCCAAACCTATTAGGTGACCCGGAGAATTTTACCCCAGCAAACCCACTCGTAACACCCCCACATATCCAGCCAGAATGATACTTCTTATTTGCCTACGCCATCCTTCGATCTATCCCGAATAAACTAGGAGGGGTCCTTGCACTACTATTCAGCATTCTAGTGCTATTAGTCGTGCCAATTCTACACACATCCAAACAACGAGGACTAACTTTCCGCCCAATGACCCAATTTCTATTCTGAACCCTGGTTGCAGACATATTCATCCTCACGTGAATCGGGGGTATACCCGTAGAACACCCATATATTGTTATTGGCCAAATCGCATCCATCCTATATTTTGCACTATTCCTCGTCCTTGTCCCACTAGCAGGATGGGTGGAAAATAAAGCATTAAAATGAGCCAY568614Achondrostomaarcasii            atggcaagcctacgaaaaactcacccgctcataaaaatcgctaacgacgcgctagtcgacctcccaacaccatctaatatttcagtaatgtgaaacttcggatcactcctaggattatgtttaattacacaaattctaacaggattattcttagccatacattacacctccgatatctcaaccgcattctcatcggtaacccacatctgccgagacgttaactacggctgacttatccgaagcctacatgccaatggagcatcctttttctttatctgtctttacatacatattgcacgaggcctatattatggatcatatctttataaagaaacctgaaccattggcgtagtcctattccttttagttataataacagcctttgtcggctacgttcttccgtggggacaaatatccttttgaggtgctactgtgattacaaaccttctatctgcagtcccatacataggagataccctcgttcaatgaatctggggtgggttttcagtagacaatgcaactctcacccgattcttcgcattccacttcctactaccatttgtcgttgccggcgcaaccatcctacacttattatttctacacgaaaccggatcgaacaacccggccggattaaattccgacgcagacaagatttctttccacccatatttttcatataaagaccttcttggctttgtggccatgttactagcccttacctccttaacgttattttccccaaacctattaggtgacccggagaattttaccccagcaaacccactcgtaacacccccacatatccagccagaatgatacttcttatttgcctacgccatccttcgatctatcccgaataaactaggaggggtccttgcactactattcagcattctagtgctattagtcgtgccaattctacacacatccaaacaacgaggactaactttccgcccaatgacccaatttctattctgaaccctggttgcagacatattcatcctcacgtgaatcgggggcatacccgtagaacacccatatattgttattggccaaatcgcatccatcctatattttgcactattcctcgtccttgtcccactagcaggatgggtggaaaataaagcattaaaatgagccHM560057Achondrostomaoccidentale        ATGGCAAGCCTACGAAAAACTCATCCGCTAATAAAAATCGCTAATGACGCGCTAGTCGACCTCCCAACACCATCTAATATTTCAGTAATGTGAAACTTCGGATCACTCCTAGGATTGTGTTTATTTACCCAAATTCTGACAGGATTGTTCTTAGCCATACATTACACCTCCGATATCTCAACCGCATTCTCATCAGTGACCCACATCTGCCGAGACGTTAACTACGGCTGACTTATCCGGAGCCTACATGCCAATGGAGCATCCTTTTTCTTCATCTGTCTTTATATGCATATTGCACGGGGCCTATATTATGGATCATATCTTTATAAAGAAACCTGAAACATTGGTGTAGTCCTGTTCCTTCTAGTTATGATAACGGCCTTTGTCGGCTACGTTCTCCCATGAGGGCAAATGTCCTTTTGAGGTGCCACTGTAATTACGAACCTCCTATCTGCGGTCCCTTACATGGGTGATACCCTCGTTCAATGAATCTGAGGCGGATTTTCAGTGGACAATGCAACTCTCACACGATTCTTCGCATTCCACTTCCTGCTACCATTTGTCGTTACCGGCGCAACCATCCTACACTTATTGTTTTTACACGAAACCGGATCAAACAACCCGGCCGGATTAAACTCCGACGCAGACAAAATTTCTTTCCACCCATATTTTTCATATAAAGACCTTCTTGGCTTTGTGGCCATATTATTAGCCCTTACCTCCCTAACATTATTTTCTCCCAACCTATTAGGTGACCCGGAAAACTTTACCCCAGCAAACCCACTCGTTACGCCACCACATATTCAGCCAGAATGATACTTCTTGTTTGCCTACGCCATCCTTCGATCTATTCCCAATAAACTGGGGGGGGTCCTTGCACTACTATTCAGCATTCTAGTGCTATTAGTTGTGCCAATTCTACACACATCCAAACAACGAGGACTAACTTTCCGCCCGATGACCCAATTTCTATTCTGAACCCTAGTTGCGGACATATTTATCCTGACATGAATCGGAGGCATACCCGTAGAACACCCATATATTATTATTGGCCAAGTCGCATCCATCCTATACTTTGCACTATTCCTCATCCTTGTCCCACTAGCAGGATGGGTGGAAAATAAAGCATTAAAATGAGCCHM560058Achondrostomaoccidentale        ATGGCAAGCCTACGAAAAACTCATCCGCTAATAAAAATCGCTAATGACGCGCTAGTCGACCTCCCAACACCATCTAATATTTCAGTAATGTGAAACTTCGGATCACTCCTAGGATTGTGTTTATTTACCCAAATTCTGACAGGATTGTTCTTAGCCATACATTACACCTCCGATATCTCAACCGCATTCTCATCAGTGACCCACATCTGCCGAGACGTTAACTACGGCTGACTTATCCGGAGCCTACATGCCAATGGAGCATCCTTTTTCTTCATCTGTCTTTATATGCATATTGCACGGGGCCTATATTATGGATCATATCTTTATAAAGAAACCTGAAACATTGGTGTAGTCCTGTTCCTTCTAGTTATGATAACGGCCTTTGTCGGCTACGTTCTCCCATGAGGGCAAATGTCCTTTTGAGGTGCCACTGTAATTACGAACCTCCTATCTGCGGTCCCTTACATGGGTGATACCCTCGTTCAATGAATCTGAGGCGGATTTTCAGTGGACAATGCAACTCTCACACGATTCTTCGCATTCCACTTCCTGCTACCATTTGTCGTTACCGGCGCAACCATCCTACACTTATTGTTTTTACACGAAACCGGATCAAACAACCCGGCCGGATTAAACTCCGACGCAGACAAAATTTCTTTCCACCCATATTTTTCATATAAAGACCTTCTTGGCTTTGTGGCCATATTATTAGCCCTTACCTCCCTAACATTATTTTCTCCCAACCTATTAGGTGACCCGGAAAACTTTACCCCAGCAAACCCACTCGTTACGCCACCACATATTCAGCCAGAATGATACTTCTTGTTTGCCTACGCCATCCTTCGATCTATTCCCAATAAACTGGGGGGGGTCCTTGCACTACTATTCAGCATTCTAGTGCTATTAGTTGTGCCAATTCTACACACATCCAAACAACGAGGACTAACTTTCCGCCCGATGACCCAATTTCTATTCTGAACCCTAGTTGCGGACATATTTATCCTGACATGAATCGGAGGCATACCCGTAGAACACCCATATATTATTATTGGCCAAGTCGCATCCATCCTATACTTTGCACTATTCCTCATCCTTGTCCCACTAGCAGGATGGGTGGAAAATAAAGCATTAAAATGAGCCDQ061932Achondrostomaoligolepis         NNNNNNNNNNNNNNNNNNNNNNNNNNNNNNNNNNNNNNNNNNNNNNNNNNNNNNgtcgacctcccaacaccgtctaatatttcagtaatgtgaaacttcggatcactcctaggattatgtttaattacacaaattctaacaggattgttcttagccatacattacacctccgatatctcgaccgcattctcatcagtaacccacatctgccgagacgttaactacggctggcttatccggagcctacatgccaatggagcatcctttttctttatctgtctttatatacatattgcacgaggcctatattatggatcatatctttataaagaaacctgaaccattggtgtagtcctgttccttttagttataataacagcctttgtcggctacgttcttccatggggacaaatatccttttgaggcgctactgtgattacaaacctcctatctgcggtcccatacataggagataccctcgttcaatggatctggggtgggttttcagtagacaatgcaactctcacccgattcttcgcattccacttcctactaccatttgtcgttgccggcgcaaccatcctacacttattatttctacacgaaaccggatcgaacaacccggccggattaaattccgacgcagacaaaatttctttccacccatatttttcatataaagaccttcttggctttgtggccatgttactagctcttacctccttaacattattttccccgaacctactaggtgacccggagaactttaccccagcaaacccactcgtaacgcccccacatatccagccagaatgatacttcttatttgcctacgccatccttcgatctatcccgaataaactaggaggggtccttgcactactattcagcattctagtgctattagtcgtgccaattctacacacgtccaaacaacgaggactaactttccgcccaatgacccaatttctattctgaaccctggttgcagacatatttatcctgacatgaatcggaggcatacccgtagagcacccatatattattattggccaaatcgcatccatcctatattttgcactattcctcgtccttgtcNNNNNNNNNNNNNNNNNNNNNNNNNNNNNNNNNNNNNNNNNNAY254683Achondrostomaoligolepis         atggcaagcctacgaaaaactcacccgctaataaaaatcgctaacgacgcgctagtcgacctcccaacaccatctaatatttcagtaatgtgaaacttcggatcactcctaggattatgtttaattacacaaattctaacaggattattcttagccatacattacacctccgatatctcgaccgcattctcatcagtaacccacatctgccgagacgttaactacggctggcttatccggagcctacatgccaatggagcatcctttttctttatctgtctttatatacatattgcacgaggcctatattatggatcatatctttataaagaaacctgaaccattggtgtagtcctgttccttttagttataataacagcctttgtcggctacgttcttccatggggacaaatatccttttgaggtgctactgtgattacaaacctcctatctgcggtcccatacatgggagataccctcgttcaatgaatctggggtgggttttcagtagacaatgcaactctcacccgattcttcgcattccacttcctactaccatttgtcgttgccggcgcaaccatcctacacttattatttctacacgaaaccgggtcgaacaacccggccggattgaattccgacgcagacaaaatttctttccacccatatttttcatataaagaccttcttggctttgtggccatgttactagctcttacctccttaacattattttccccatacctattaggtgacccggagaactttaccccagcaaacccactcgtaacgcccccacatatccagccagaatgatacttcttatttgcctacgccatccttcgatctatcccgaataaactaggaggggtccttgcactactattcagcattctagtgctattagtcgtgccagttctacacacgtccaaacaacgaggactaactttccgcccaatgacccaatttctattctgaaccctggttgcagacatatttatcctgacgtgaatcgggggcatacccgtagaacacccatatattattattggccaaatcgcatccatcctatattttgcactattcctcgtccttgtcccactagcaggatgggtggagaataaagcattgaaatgagccAY568612Achondrostomasalmantinum        atggcaagcctacgaaaaacccacccactaataaaaatcgctaacgacgcgctagtcgacctcccaacaccatctaatatctcagcaatgtgaaacttcggatctctcctaggactgtgtttaatcacccaaatcctaacaggattattcttagccatacattacacctcggatatctcaaccgcattctcatcagtagcccacatctgccgagatgttaactacggctgacttatccgaagtctacatgccaatggtgcatcctttttcttcatctgtctttatatacatattgcacgaggcctgtattacgggtcatacctttataaagaaacctgaaacattggtgtagtcctgttccttcttgttatgatgacagcctttgtcggctatgttcttccatgagggcaaatatccttttgaggtgctaccgtaattacaaacctcctgtctgcagttccatacataggagataccctcgttcaatgaatctgagggggattctcagtagacaatgcgactctcacacgattcttcgcattccacttcctactgccatttgtcgttgccggcgcaaccatcctacacttgctatttctacacgaaacgggatctaacaacccggccggactaaattccgacgcagacaagatttctttccacccatatttctcatataaagaccttctcggctttgtagccatgctactagccgttacctccctaacattattttcccctaacctactaggtgacccggaaaactttaccccagcaaacccgctcgtaacacccccacatatccaaccagaatgatacttcttatttgcctacgctatccttcgatctatcccaaataaactaggaggggttcttgcactactattcagcattctagtgctaatagtcgtgccaattttacacacatccaaacaacgaggactaactttccgaccagtaacccaattcctattctgaaccctggttgcagatatatttatcttgacatgaatcgggggtatacccgtagagcacccatatattatcattggccaaatcgcatccgtcctatattttgcactattccttattcttgtcccactagcaggatgggtggaaaataaggcattgaaatgagccAY568613Achondrostomasalmantinum        atggcaagcctacgaaaaacccacccactaataaaaatcgctaacgacgcgctagtcgacctcccaacaccatctaatatctcagcaatgtgaaacttcggatctctcctaggactgtgtttaatcacccaaatcctaacaggattattcttagccatacattacacctcggatatctcaaccgcattctcatcagtagcccacatctgccgagatgttaactacggctgacttatccgaaatctacatgccaatggtgcatcctttttcttcatctgtctttatatacatattgcacgaggcctgtattacgggtcatacctttataaagaaacctgaaacattggtgtagtcctgttccttcttgttatgatgacagcctttgtcggctatgttcttccatgagggcaaatatccttttgaggtgctaccgtaattacaaacctcctgtctgcagttccatacataagggataccctcgttcaatgaatctgaaggggattctcagtagacaatgcgactctcacacgattcttcgcattccacttcctactgccatttgtcgttgccggcgcaaccatcctacacttgctatttctacacgaaacgggatctaacaacccggccggactaaattccgacgcagacaagatttctttccacccatatttctcatataaggaccttctcggctttgtagccatgctactagccgttacctccctaacattattttcccataacctactaggtgacccggaaaactttaccccagcaaacccgctcgtaacacccccacatatccaaccagaatgatacttcttatttgcctacgctatccttcgatctatcccaaataaactaggaggggttcttgcactactattcagcattctagtgctaatagtcgtgccaattttacacacatccaaacaacgaggactaactttccgaccagtaacccaattcctattctgaaccctggttgcagatatatttatcttgacatgaatcgggggtatacccgtagagcacccatatattatcattggccaaatcgcatccgtcctatattttgcactattccttattcttgtcccactagcaggatgggtggaaaataaggcattgaaatgagccAY568625Achondrostomasp                 atggcaagcctacgaaaaactcacccactaataaaaatcgctaacgacgcactagtcgacctcccaacaccatctaatatttcagtaatgtgaaacttcggatcactcctagggttgtgtttaattacccaaattctaacaggattgtttttagccatacattacacctccgatatctcaaccgcgttctcatcagtgacccacatctgccgagacgttaactacggctgacttatccggagcctacatgccaatggagcatcctttttcttcatctgtctttatatgcatattgcacggggcctatattatggatcgtatctttataaagaaacctgaaacattggtgtagtcctgttccttctagttatgataacagcctttgtcggctacgttctcccatgaggacaaatgtccttttgaggcgccactgtaattacaaacctcctatctgcggtcccttacataagggatactctcgttcaatgaatctgaggcggattttcagtggataatgcgactcttacacgattcttcgcattccacttcctgctgccatttgtcgttgccggcgcaaccatcctacacttattgtttttacacgaaaccggatcaaacaacccggccggattaaactccgacgcagacaaaatttctttccacccatatttttcatataaagaccttcttggctttgtggccatgctactagcccttacctccctaacattattttcccctaacctattaggtgacccggaaaactttaccccagcaaacccactagttacgccaccacatatccagccagagtggtacttcttgtttgcctacgccatccttcgatctatcccaaataaactaggaggggtccttgcactactgttcagcattctagtgctattagttgtgccaattctacacacatccaaacaacgaggactaactttccgtccaatgacccaatttctattctgaaccctagttgcagacatatttatcctgacatgaattgggggcatacccgtagaacatccatatattattattggccaagtcgcatccatcctatactttgcactatttctcatccttgtcccactagcaggatgggtggaaaataaagcattgaaatgagccAY568626Achondrostomasp                 atggcaagcctacgaaaaactcacccactaataaaaatcgctaacgacgcgctagtcgacctcccaacaccatctaatatttcggtaatgtgaaacttcggatcactcctagggttgtgtttaattacccaaattctaacaggattgtttttagccatacattacacctccgatatctcaaccgcgttctcatcagtgacccacatctgccgagacgttaactacggctgacttatccggagcctacatgccaatggagcatcctttttcttcatctgcctttatatgcatattgcacggggcctatattatggatcgtatctttataaagaaacctgaaacattggtgtagtcctgttccttctagttatgataacagcctttgtcggctacgttctgccatgaggacaaatgtccttttgaggcgccactgtaattacaaacctcctatctgcggtcccttacataggggatactctcgttcaatgaatctgaggcgggttttcagtggataatgcgactcttacacgattcttcgcgttccacttcctgctgccatttgtagttgccggcgcaaccatcctacacttattgtttttacacgaaaccggatcaaacaacccggccggattaaactccgacgcagacaaaatttctttccacccatatttttcatataaagaccttcttggctttgtggccatgttactagcccttacctccctaacattattttcccccaacctattaggtgacccggaaaactttaccccagcaaacccactcgttacgccaccacatatccagccagaatgatacttcttatttgcctacgccatccttcgatctatcccaaataaactaggaggggtccttgcactactattcagcattctagtgctattagttgtgccaattctacacacatccaaacaacgaggactaactttccgcccaataacccaatttctattctgaaccctagttgcagacatatttatcctgacgtgaattgggggcatacccgtagaacatccgtatattattattggccaagtcgcatccatcctatactttgcactatttcttatccttgtcccactagcaggatgggtggaaaataaagcattgaaatgagccY10445Alburnoidesbipunctatus            atggcaagcctacgaaaaactcccccactaataaaaatcgcgaatggtgcactagttgaccttccaaccccatctaatatttcagcactctgaaacttcggctccctgctagggctgtgtttaattacccaaattctaacaggactatttttagccatgcactatacctccgatatctcaaccgcattttcatcagtaacccacatctgtcgagacgtcaactacggctgacttatccgaagccttcacgctaacggagcatcttttttcttcatctgcctttacttgcacattgcacgaggcctatattatggctcctacctttacaaagaaacctgaaacatcggcgtagtcctattccttctagtaataatgacagccttcgtcggctacgtacttccatggggacaaatatcattttggggcgccacngtcatcacgaatctcctttcagcagtaccttacatgggagacatacttgttcaatgaatctggggcggcttctcagtagataacgcaaccctcacacgattcttcgcgttccacttcctcctcccattcgtcgtcgccggcgcaaccattctccacttgctattcctacacgagacaggatcaaacaacccggccggattaaattccgacgcagacaaaatttctttccacccatacttctcatataaagaccttcttggctttgtaatcatgttactagccctcacctccctaacgctattttctcccaacctactaggtgacccagaaaattttacccctgcgaacccccttgtgacacccccacacatccaacctgagtgatacttcctgtttgcctatgccatcctacgatctatcccaaataaacttggaggggtccttgcgctgctatttagcatcttggtgctaatagtcgtgccaattctacacacctcaaaacaacggggactaactttccgcccgctaacacaattcctattctgaaccctagttgcagatataattattttaacatgaattgggggcatacctgtagagcacccgtacattattattggccaaattgcatcaattttatactttgctctttttctcgttcttgccccactagcaggatgagtagaaaataaagcactgaaatgagccHM560059Alburnoidesbipunctatus          ATGGCAAGCCTACGAAAAACTCACCCACTAATAAAAATCGCGAATGGTGCACTAGTTGACCTTCCAACCCCATCTAATATTTCAGCACTCTGAAACTTCGGCTCCCTGCTAGGGCTGTGTTTAATTACCCAAATTCTAACAGGACTATTTTTAGCCATGCACTATACCTCCGATATCTCAACCGCATTTTCATCAGTAACCCACATCTGTCGAGACGTCAACTACGGCTGACTTATCCGAAGCCTTCACGCTAACGGAGCATCTTTTTTCTTCATCTGCCTTTACTTGCACATTGCACGAGGCCTATATTATGGCTCCTACCTTTACAAAGAAACCTGAAACATCGGCGTAGTCCTATTCCTTCTAGTAATAATGACAGCCTTCGTCGGCTACGTACTTCCATGGGGACAAATATCATTTTGGGGCGCCACCGTCATCACGAATCTCCTTTCAGCAGTACCTTACATGGGAGACATACTTGTTCAATGAATCTGGGGCGGCTTCTCAGTAGATAACGCAACCCTCACACGATTCTTCGCGTTCCACTTCCTCCTCCCATTCGTCGTCGCCGGCGCAACCATTCTCCACTTGCTATTCCTACACGAGACAGGATCAAACAACCCGGCCGGATTAAATTCCGACGCAGACAAAATTTCTTTCCACCCATACTTCTCATATAAAGACCTTCTTGGCTTTGTAATCATGTTACTAGCCCTCACCTCCCTAACGCTATTTTCTCCCAACCTACTAGGTGACCCAGAAAATTTTACCCCTGCGAACCCCCTTGTGACACCCCCACACATCCAACCTGAGTGATACTTCCTGTTTGCCTATGCCATCCTACGATCTATCCCAAATAAACTTGGAGGGGTCCTTGCGCTGCTATTTAGCATCTTGGTGCTAATAGTCGTGCCAATTCTACACACCTCAAAACAACGGGGACTAACTTTCCGCCCGCTAACACAATTCCTATTCTGAACCCTAGTTGCAGATATAATTATTTTAACATGAATTGGGGGCATACCTGTAGAGCACCCGTACATTATTATTGGCCAAATTGCATCAATTTTATACTTTGCTCTTTTTCTCGTTCTTGCCCCACTAGCAGGATGAGTAGAAAATAAAGCACTGAAATGAGCCAF090741Alburnoidesprespensis           atggcaagcctacgaaaaacccacccactaataaaaatcgcgaatggtgcactagttgaccttccaaccccatctaatatttccgcactctgaaatttcgggtccctgttagggctatgtttaattacccaaattctaacgggactatttttagccatgcactacacctctgatatctcaaccgcattttcatcagtaacccacatctgtcgagacgtcaactacggctgacttatccgaagccttcacgctaatggggcatcttttttcttcatctgcctttacttacacattgcacgaggcctatattatggctcctacctttataaagaaacctgaaacatcggcgtggtcctattccttctagtaataatgacagccttcgtcggctacgtacttccatggggacaaatatcattttgaggcgccaccgtcatcacaaacctcctttcagcagtaccttacatgggagacatacttgttcaatgaatctggggcggcttctcagtagataacgcaaccctcacacgattcttcgcgttccacttcctcctcccattcgtcgtcgccggcgcaaccatcctccacttgctgttcctacacgagacagggtcaaacaacccggccggattaaactccgatgcggacaaaatttctttccacccatacttctcatataaagacctccttggctttgtaattatattactagccctcacctccctaaccctattttctcccaacctactaggtgacccagaaaattttacccctgcgaacccacttgtaacgcctccacacatccagcctgaatggtacttcctgtttgcctatgccatcctacgatccatcccaaataaacttgggggggtccttgcgctgctatttagcatcttagtactaatagtcgtgccaattttacacacctcaaaacaacgaggactgactttccgcccgctaacacaattcctattctgaaccctggttgcagatataattatcttaacatgaattgggggcatacctgtagagcacccgtacattattattggccaaattgcatcaattttatactttgcgctttttctcgttcttgccccactagcaggatgagtagaaaataaagcactgaaatgagccAF09740Alburnidesprespensis             atggcaagcctacgaaaaacccacccactaataaaaatcgcgaatggtgcactagttgaccttccaaccccatctaatatttccgcactctgaaatttcgggtccctgctaggactatgtttaattacccaaattctaacgggactatttttagccatgcactacacctctgatatctcaaccgcattttcatcagtaacccacatctgtcgagacgtcaactacggctgacttatccgaagccttcacgctaatggggcatcttttttcttcatctgcctttacttacacattgcacgaggcctgtattatggctcctacctttataaagaaacctgaaacatcggcgtggtcctattccttctagtaataatgacagccttcgtcggctacgtacttccgtggggacaaatatcattttgaggcgccaccgttatcacaaacctcctttcagcagtaccttacatgggagacatacttgttcaatgaatctggggcggcttctcagtagataacgcaacccttacacgattcttcgcgttccacttcctcctcccattcgtcgtcgccggcgcaaccatcctccacttgctgttcctacacgagacagggtcaaacaacccggccggattaaactccgatgcagacaaaatttctttccacccatacttctcgtataaagaccttcttggctttgtaattatattactagccctcacctccctaaccctattttctcccaacctactaggtgacccagaaaattttacccctgcgaacccacttgtaacgcccccacacatccagcctgaatggtacttcctgtttgcctatgccatcctacgatccatcccaaataaactggggggggtccttgcgctgctatttagcatcttagtactaatagtcgtgccgattttacacacctcaaaacaacgaggactaactttccgcccgntaacacaattcatattctgaaccctggttgcagatataattatcttaacatgaattgggggcatacctgtagagcacccgtacattattattggccaaattgcatcaattttatactttgcgctttttctcgttcttgccccactagcaggatgagtagaaaataaagcactgaaatgagccAF090742Alburnoidesstrymonicus          atggcaagcctacgaaaaacccacccactaataaaaatcgcgaatggtgcactagttgaccttccaaccccctctaatatttcggcactttgaaacttcggatccctgctgggattatgtttaattacccaaattctaacgggattgtttttagccatacactacacctctgatatctcaaccgcattttcatcagtgacccacatctgtcgagacgttaactacggctgacttatccgaagccttcacgctaacggcgcatcttttttcttcatctgcctttatttacacattgcacgaggcctatattatggctcctacctttacaaagaaacctgaaacatcggcgtagtcctatttcttttagtgataataacagccttcgtcggctacgtgctcccatggggacaaatatcattttgaggtgccaccgtcattacaaatctcctttcagcagtaccctacatgggagacatacttgttcaatgaatctgaggcggcttctcagtagacaacgcaaccctcacacgattcttcgcgttccacttccttctcccatctgtcgtcgccggcgcaaccattctgcacttgctatttctacacgagacggggtcgaacaatccggccggnttaaactccgatgcagacaagatttctttccacccatacttctcatataaagatctccttggctttgtaatcatgttactagccctcacctccctgaccctattttctccgaacctattaggtgacccagaaaattttacccctgcgaacccacttgtaacgcccccacacatccaaccagagtgatacttcttgtttgcctatgctatcctgcgatctattccaaataaacttggaggggtccttgcgctactatttagcatcttggtattaatggtcgtgccagtcttacacacctcaaaacaacgaggactaactttccgccctttgacacaatgcctattctgaaccctcgttgcagatatgattatcttaacatgaattgggggcatacccgtagagcacccgtacattattattggccaaattgcatcaattttatactttgcactttttctcgttcttgccccgctagcaggatgaatagaaaataaagcactgaaatgagccNC_008659Alburnusalburnus               atggcaagcctacgaaaaacccacccactaataaaaatcgctaatgacgcgctagtcgatcttccaacaccatctaacatttcagcaatatgaaatttcggatcccttctagggttatgtttaattacccaaattctaacagggttattcctagccatacactacacctccgatatctcaaccgcattctcatcagtcacccatatttgccgggacgttaactacggctggctcattcgaaacctacatgccaacggagcatccttcttcttcatctgcctatatatgcatatcgcacgaggtctatattacggctcatatctttataaagagacctgaaacattggggtagtactatttcttctggttatgataacagccttcgtgggctatgtactcccatgaggacaaatatccttttgaggtgctaccgtaatcacgaacctcctctcagcagttccctacatgggagatacccttgttcaatgaatttggggcggtttctcagtagataacgcgactcttacgcgattcttcgcgttccacttcctcctgccgttcgtcgttgcaggcgcaaccgtcctccacttactattcctacacgagacaggatcaaacaacccagccgggttaaactctgacgcggataaaatttctttccacccatacttctcctacaaagaccttctcggcttcgtaatcatgctgctggccctcacctcgctggcgctattttcccccaacctcctaggtgatccagagaactttaccccagcaaacccacttgtgacacccccacatatccaaccagagtgatacttcttgtttgcatacgccatcctccggtctattcctaataaactaggcggggttcttgcactattatttagtattctagtgctaatagttgtgccaattctacacacctcaaaacaacgaggactaactttccgccccgtgacacaattcctattttgaaccctagtcgcagatatgattatcttaacatgaattgggggcatgcctgtagagcacccatacattattattggtcaggtcgcatccgtcctatactttgcactcttccttatccttattccactagcagggttaatagagaataaagcattgaaatgagctAB239593Alburnusalburnus                atggcaagcctacgaaaaacccacccactaataaaaatcgctaatgacgcgctagtcgatcttccaacaccatctaacatttcagcaatatgaaatttcggatcccttctagggttatgtttaattacccaaattctaacagggttattcctagccatacactacacctccgatatctcaaccgcattctcatcagtcacccatatttgccgggacgttaactacggctggctcattcgaaacctacatgccaacggagcatccttcttcttcatctgcctatatatgcatatcgcacgaggtctatattacggctcatatctttataaagagacctgaaacattggggtagtactatttcttctggttatgataacagccttcgtgggctatgtactcccatgaggacaaatatccttttgaggtgctaccgtaatcacgaacctcctctcagcagttccctacatgggagatacccttgttcaatgaatttggggcggtttctcagtagataacgcgactcttacgcgattcttcgcgttccacttcctcctgccgttcgtcgttgcaggcgcaaccgtcctccacttactattcctacacgagacaggatcaaacaacccagccgggttaaactctgacgcggataaaatttctttccacccatacttctcctacaaagaccttctcggcttcgtaatcatgctgctggccctcacctcgctggcgctattttcccccaacctcctaggtgatccagagaactttaccccagcaaacccacttgtgacacccccacatatccaaccagagtgatacttcttgtttgcatacgccatcctccggtctattcctaataaactaggcggggttcttgcactattatttagtattctagtgctaatagttgtgccaattctacacacctcaaaacaacgaggactaactttccgccccgtgacacaattcctattttgaaccctagtcgcagatatgattatcttaacatgaattgggggcatgcctgtagagcacccatacattattattggtcaggtcgcatccgtcctatactttgcactcttccttatccttattccactagcagggttaatagagaataaagcattgaaatgagctHM560060Alburnusalburnus                ATGGCAAGCCTACGAAAAACCCACCCACTAATAAAAATCGCTAATGACGCGCTAGTCGATCTTCCAACACCATCTAACATTTCAGCAATATGAAATTTCGGATCCCTTCTAGGGTTATGTTTAATTACCCAAATCCTAACAGGGTTATTCCTAGCCATACACTACACCTCCGATATCTCAACCGCATTCTCATCAGTCACCCATATTTGCCGGGACGTTAACTACGGCTGGCTCATTCGAAACCTACATGCCAACGGAGCATCCTTCTTCTTCATCTGCCTATATATGCATATCGCACGAGGTCTATATTACGGCTCATATCTTTATAAAGAGACCTGAAACATTGGGGTAGTACTATTTCTTCTGGTTATGATAACAGCCTTCGTGGGCTATGTACTCCCATGAGGACAAATATCCTTTTGAGGCGCTACCGTAATCACGAACCTCCTCTCAGCAGTTCCCTACATGGGAGATACCCTTGTTCAATGAATTTGGGGCGGTTTCTCAGTAGATAACGCGACTCTTACGCGATTCTTCGCGTTCCACTTCCTCCTGCCGTTCGTCGTTGCAGGCGCAACCGTCCTCCACTTACTATTCCTACACGAGACAGGATCAAACAACCCAGCCGGGTTAAACTCTGACGCGGATAAAATTTCTTTCCACCCATACTTCTCCTACAAAGACCTTCTCGGCTTCGTACTCATGCTGCTCGCCCTCACCTCGCTGGCGCTATTTTCCCCCAACCTCCTAGGTGATCCAGAGAACTTTACCCCAGCAAACCCACTTGTGACACCCCCACATATCCAACCAGAGTGATACTTCTTGTTTGCATACGCCATCCTCCGGTCTATTCCTAATAAACTAGGCGGGGTTCTTGCACTACTGTTTAGTATTCTAGTGCTAATAGTTGTGCCAATTCTACATACCTCAAAACAACGAGGACTAACTTTCCGCCCCGTGACACAATTCCTATTTTGAACCCTAGTCGCAGATATGATTATCTTAACATGAATTGGGGGCATGCCTGTAGAGCACCCATACATTATTATTGGTCAGGTCGCATCCGTCCTATACTTTGCACTCTCCCTTATCCTTATTCCACTAGCAGGGTTAATAGAGAATAAAGCATTGAAATGAGCTHM560061Alburnusalburnus                ATGGCAAGCCTACGAAAAACCCACCCACTAATAAAAATCGCTAATGACGCGCTAGTCGATCTTCCAACACCATCTAACATTTCAGCAATATGAAATTTCGGATCCCTTCTAGGGTTATGTTTAATTACCCAAATCCTAACAGGGTTATTCCTAGCCATACACTACACCTCCGATATCTCAACCGCATTCTCATCAGTCACCCATATTTGCCGGGACGTTAACTACGGCTGGCTCATTCGAAACCTACATGCCAACGGAGCATCCTTCTTCTTCATCTGCCTATATATGCATATCGCACGAGGTCTATATTACGGCTCATATCTTTATAAAGAGACCTGAAACATTGGGGTAGTACTATTTCTTCTGGTTATGATAACAGCCTTCGTGGGTTATGTACTCCCATGAGGACAAATATCCTTTTGAGGCGCTACCGTAATCACGAACCTCCTCTCAGCAGTTCCCTACATGGGAGATACCCTTGTTCAATGAATTTGGGGCGGTTTCTCAGTAGATAACGCGACTCTTACGCGATTCTTCGCGTTCCACTTCCTCCTGCCGTTCGTCGTTGCAGGCGCAACCGTCCTCCACTTACTATTCCTACACGAGACAGGATCAAACAACCCAGCCGGGTTAAACTCTGACGCGGATAAAATTTCTTTCCACCCATACTCCTCCTACAAAGACCTTCTCGGCTTCGTACTCATGCTGCTCGCCCTCACCTCGCTGGCGCTATTTTCCCCCAACCTCCTAGGTGATCCAGANAACTTTNCCCCAGCAAACCCACTTGTGACACCCCCACATATCCAACCAGAGTGATACTTCTTGTTTGCATACGCCATCCTCCGGTCTATTCCTAATAAACTAGGCGGGGTTCTTGCACTACTGTTTAGTATTCTAGTGCTAATAGTTGTGCCAATTCTACATACCTCAAAACAACGAGGACTAACTTTCCGCCCCGTGACACAATTCCTATTTTGAACCCTAGTCGCAGATATGATTATCTTAACATGAATTGGGGGCATGCCTGTAGAGCACCCATACATTATTATTGGTCAGGTCGCATCCGTCCTATACTTTGCACTCTTCCTTATCCTTATTCCACTAGCAGGGTTAATAGAGAATAAAGCATTGAAATGAGCTHM560062Alburnusalburnus                ATGGCAAGCCTACGAAAAACCCACCCACTAATAAAAATCGCTAATGACGCGCTAGTCGATCTTCCAACACCATCTAACATTTCAGCAATATGAAATTTCGGATCCCTTCTAGGGTTGTGTTTAATTACCCAAATCCTAACAGGGCTATTCCTAGCCATACACTACACCTCCGATATCTCAACCGCATTCTCATCAGTCACCCATATTTGCCGAGACGTTAACTACGGCTGACTCATTCGAAACCTACATGCCAACGGAGCATCCTTCTTCTTCATCTGCCTATATATGCATATCGCACGAGGTCTATATTACGGCTCATATCTTTATAAAGAGACCTGAAACATTGGGGTAGTACTATTTCTTCTGGTTATGATAACAGCCTTCGTGGGCTATGTGCTCCCATGAGGGCAAATATCCTTTTGAGGTGCTACCGTAATCACGAACCTCCTCTCAGCAGTTCCCTACATGGGAGATACCCTTGTTCAATGAATTTGGGGCGGTTTCTCAGTAGATAACGCGACTCTTACGCGATTCTTCGCGTTCCACTTCCTCCTGCCGTTCGTCGTTGCAGGCGCAACCGTCCTCCACTTACTATTCCTACACGAGACAGGATCAAACAACCCAGCCGGGTTAAACTCTGACGCGGATAAAATTTCTTTCCACCCATACTTCTCCTACAAAGACCTTCTCGGCTTCGTAATCATGCTGCTGGCCCTCACCTCGCTGGCGCTATTTTCCCCCAACCTCCTAGGTGATCCAGAGAACTTTACCCCAGCGAACCCACTTGTGACACCCCCACATATCCAACCAGAGTGATACTTCTTGTTTGCATACGCCATCCTCCGGTCTATTCCTAATAAACTAGGCGGGGTTCTTGCACTATTGTTTAGTATTCTAGTGCTAATAGTTGTGCCAATTCTACACACCTCAAAACAACGAGGACTAACTTTCCGCCCCGTGACGCAATTCCTATTTTGAACCCTAGTCGCAGATATGATTATCTTAACATGAATTGGGGGCATGCCCGTAGAGCACCCATACATTATTATTGGTCAGGTCGCATCCGTCCTATACTTTGCACTCTTCCTTATCCTTATTCCACTAGCAGGGTTAATAGAGAATAAAGCATTGAAATGAGCTHM560063Alburnusarborella               ATGGCAAGCCTACGAAAAACCCACCCACTAATAAAAATCGCTAATGACGCGCTAGTCGATCTTCCAACACCATCTAACATTTCAGCAATGTGAAATTTCGGATCCCTTCTAGGGTTGTGTTTAATTACCCAAATCCTAACAGGACTATTCCTAGCCATACACTACACCTCTGATATCTCAACCGCATTCTCATCAGTCACCCATATTTGCCGAGACGTTAACTACGGCTGGCTCATTCGAAACCTACACGCCAACGGAGCATCCTTCTTCTTTATCTGCCTATACATGCATATCGCACGGGGTCTATATTACGGCTCATATCTTTATAAGGAGACCTGAAACATTGGGGTAGTACTATTTCTTCTGGTTATGATAACAGCCTTCGTGGGCTATGTACTCCCATGAGGACAAATATCCTTTTGAGGTGCTACCGTAATCACAAACCTCCTCTCAGCAGTTCCCTACATGGGAGACACCCTTGTTCAATGAATTTGGGGCGGTTTCTCAGTAGATAACGCGACTCTTACGCGGTTCTTCGCGTTCCACTTTCTCCTGCCGTTCGTCGTTGCAGGCGCAACCGTCCTCCACTTACTATTCCTACACGAAACAGGGTCAAACAACCCAGCCGGGCTAAACTCTGACGCGGATAAAATTTCTTTCCACCCATACTTCTCCTACAAAGACCTTCTCGGCTTCGTACTCATGCTGCTGGCCCTCACCTCGCTGGCGCTATTTTCCCCTAACCTCCTAGGTGATCCAGAGAACTTTACCCCAGCAAACCCACTTGTGACACCCCCACATATTCAACCAGAGTGATACTTCTTGTTTGCATACGCCATCCTCCGGTCTATTCCTAATAAACTAGGCGGGGTTCTTGCACTATTATTTAGTATCCTAGTGCTTATAGTTGTGCCGATTCTACACTACTCAAAACAACGAGGACTAACTTTTCGCCCCGTGACGCATTTCCTATTTTGAACCCTAGTCGCAGATATGATTATCTTAACATGAATTGGGGGCATGCCCGTAGAGCACCCATACATTATTATTGGTCAGGTCGCATCCGTCCTATACTTTGCACTCTTCCTCATCCTTATTCCACTAGCAGGACTAATAGAGAATAAAGCATTGAAATGAGCTHM560064Alburnusarborella               ATGGCAAGCCTACGAAAAACCCACCCACTAATAAAAATCGCTAATGACGCGCTAGTCGATCTTCCAACACCATCTAACATTTCAGCAATGTGAAATTTCGGATCCCTTCTAGGGTTGTGTTTAATTACCCAAATCCTAACAGGACTATTCCTAGCCATACACTACACCTCTGATATCTCAACCGCATTCTCATCAGTCACCCATATTTGCCGAGACGTTAACTACGGCTGGCTCATTCGAAACCTACACGCCAACGGAGCATCCTTCTTCTTTATCTGCCTATACATGCATATCGCACGGGGTCTATATTACGGCTCATATCCTTATAAGGAGACCTGAAACATTGGGGTAGTACTATTTCTTCTGGTTATGATAACAGCCTTCGTGGGCTATGTACTCCCATGAGGACAAATATCCTTTTGAGGTGCTACCGTAATCACAAACCTCCTCTCAGCAGTTCCCTACATGGGAGACACCCTTGTTCAATGAATTTGGGGCGGTTTCTCAGTAGATAACGCGACTCTTACGCGGTTCTTCGCGTTCCACTTTCTCCTGCCGTTCGTCGTTGCAGGCGCAACCGTCCTCCACTTACTATTCCTACACGAAACAGGGTCAAACAACCCAGCCGGGCTAAACTCTGACGCGGATAAAATTTCTTTCCACCCATACTTCTCCTACAAAGACCTTCTCGGCTTCGTAATCATGCTGCTGGCCCTCACCTCGCTGGCGCTATTTTCCCCTAACCTCCTAGGTGATCCAGAGAACTTTACCCCAGCAAACCCACTTGTGACACCCCCACATATTCAACCAGAGTGATACTTCTTGTTTGCATACGCCATCCTCCGGTCTATTCCTAATAAACTAGGCGGGGTTCTTGCACTATTATTTAGTATCCTAGTGCTAATAGTTGTGCCGATTCTACACACCTCAAAACAACGAGGACTAACTTTCCGCCCCGTGACGCAATTCCTATTTTGAACCCTAGTCGCAGATATGATTATCTTAACATGAATTGGAGGCATGCCCGTAGAGCACCCATACATTATTGTTGGTCAGGTCGCATCCGTCCTATACTTTGCACTCTTCCTCATCCTTATTCCACTAGCAGGACTAATAGAGAATAAAGCATTGAAATGAGCTAY838936Alburnusbaliki                  atggcaagcctacgaaaaacccacccactaataaaaatcgctaatgacgcactagtcgatcttccaacaccatccaatatttcagcaatatgaaacttcggatcccttctaggattgtgtttaattacccaaatcctaacaggattattcctggctatacattacacctctgacatctcaaccgccttttcatcagtaacccatatctgccgggacgttaactacggctgactcattcgaaacctacatgccaacggagcatccttcttcttcatttgcctgtatatacacatcgcacgaggcctgtactacggctcatatctttataaagaaacctgaaatattggagtagtactatttcttctggttataatgaccgccttcgtgggctacgtacttccgtgagggcagatgtccttctgaggggccactgtcattacaaatcttctctcggcggtcccttatataggggacacccttgttcagtgaatctgaggaggcttctcagtagataatgcaaccctcacacgattcttcgcattccacttcctcttaccattcgtcgttgcaggcgcaaccgtcctacacttgttgttcctacacgaaacgggatcgaacaacccggccgggctaaattccgacgcagacaaaatttctttccacccatacttctcgtacaaagaccttctcggcttcgtgatcatattactggccctcacctccctggcgctattttcgccgaacctattaggtgatccagagaactttaccccagcaaacccactcgtgacaccccctcatatccagccagaatgatatttcttatttgcatacgccattctccgatctattcctaataaactaggcggggttcttgcactattattcagcattctagtgctaatggttgtgccaattctacacacctcaaaacaacgaggactaactttccgtccggtgactcaattcttgttttggaccttagttgcagacataattattctgacatgaattggaggtatacccgtagaacacccatatattattattggtcagatcgcatccctgctgtactttgcacttttccttattcttattccactagcaggattaatggaaaataaagcattgaaatgagccHM560065Alburnusbelvica                 ATGGCAAGCCTACGAAAAACCCACCCACTAATAAAAATCGCTAATGACGCGCTAGTCGATCTTCCAACACCATCTAACATTTCAGCAATGTGAAATTTCGGATCCCTTCTAGGGTTGTGTTTAATTACCCAAATCCTAACAGGATTATTCCTAGCCATACACTACACCTCTGATATCTCGACCGCATTCTCATCAGTCACCCATATTTGCCGAGACGTTAACTACGGCTGGCTCATTCGAAACCTACATGCCAACGGAGCATCCTTCTTCTTTATTTGCCTATATATGCATATCGCACGAGGTCTATATTACGGCTCATATCTTTATAAGGAGACCTGAAACATTGGGGTAGTACTATTTCTTCTGGTTATGATAACAGCCTTTGTGGGCTATGTGCTCCCATGAGGACAAATATCCTTTTGAGGTGCTACCGTAATCACAAATCTCCTCTCAGCAGTTCCCTACATAGGAGATACCCTTGTTCAATGAATTTGGGGCGGTTTCTCAGTAGATAACGCGACTCTTACGCGATTCTTCGCGTTCCACTTCCTGCTGCCGTTCGTCGTTGCAGGCGCAACCGTCCTCCACTTACTATTCCTACACGAGACAGGATCAAACAACCCAGCCGGGCTAAACTCTGACGCGGATAAAATTTCCTTCCACCCATACTTCTCCTACAAAGACCTTCTCGGCTTCGTGATCATGCTGCTGGCCCTCACCTCGCTAGCGCTATTTTCCCCTAACCTCCTAGGTGATCCGGAGAACTTTACCCCAGCAAACCCACTTGTGACACCCCCACATATCCAACCAGAGTGATACTTCCTGTTTGCATACGCCATCCTCCGGTCTATCCCTAATAAACTAGGCGGGGTTCTTGCACTATTATTTAGTATCCTAGTGCTAATGGTTGTGCCAATTCTACACACCTCAAAACAACGAGGACTAACTTTCCGCCCCGTGACGCAATTCCTATTTTGAACCCTAGTTGCAGATATGGTTATCTTAACATGAATTGGGGGCATGCCCGTAGAACACCCGTACATTATTATTGGTCAGATCGCATCCGTCCTATACTTTGCACTCTTCCTTATCCTTATTCCACTAGCAGGATTAATAGAGAATAAAGCATTGAAATGAGCTAF090746Alburnusbelvica                 atggcaagcctacgaaaaacccacccactaataaaaatcgctaatgacgcgctagtcgatcttccaacaccatctaacatttcagcaatgtgaaatttcggatcccttctagggttgtgtttaattacccaaatcctaacaggattattcctagccatacactacacctctgatatctcgaccgcattctcatcagtcacccatatttgccgagacgttaactacggctggctcattcgaaacctacatgccaacggagcatccttcttctttatttgcctatatatgcatatcgcacgaggtctatattacggctcatatctttataaggagacctgaaacattggggtagtactatttcttctggttatgataacagcctttgtgggctatgtgctcccatgaggacaaatatccttttgaggtgctaccgtaatcacaaatctcctctcagcagttccctacataggagatacccttgttcaatgaatttggggcggtttctcagtagataacgcgactcttacgcgattcttcgcgttccacttcctgctgccgttcgtcgttgcaggcgcaaccgtcctccacttactattcctacacgagacaggatcaaacaacccagccgggctaaactctgacgcggataaaatttccttccacccatacttctcctacaaagaccttctcggcttcgtgatcatgctgctggccctcacctcgctagcgctattttcccctaacctcctaggtgatccggagaactttaccccagcaaacccacttgtgacacccccacatatccaaccagagtgatacttcctgtttgcatacgccatcctccggtctatccctaataaactaggcggggttcttgcactattatttagtatcctagtgctaatggttgtgccaattntacacacctcaaaacaacgaggactaactttccgccccgtgacgcaattcctattttgaaccctagttgcagatatggttatcttaacatgaattgggggcatgcccgtagaacacccgtacattattattggtcagatcgcatccgtcctatactttgcactcttccttatctttattccactagcaggattaatagagaataaagcattgaaatgagctAY026390Alburnudemiri                   atggcaagcctacgaaaaacccacccactaataaaaatcgctaatgacgcgctagtcgatcttccaacaccatctaacatttcagcaatgtgaaattttggatcccttctaggattatgtttaattacccaaatcctaacagggttgttcctagccatacactacacctctgatatctcaaccgcattctcatcagtcacccatatttgccgagacgttaactacggctggctcattcgaaacttacatgccaacggagcatccttcttcttcatctgcctatacatgcatatcgcacgaggtctgtattacggctcatatctttataaggaaacctgaaacattggggtagtactatttcttcttgttatgatgacagccttcgtgggctatgtacttccatgaggacaaatatccttttggggtgctaccgtaatcacaaatctcctctcagcagttccctacatgggagacacccttgtccagtgaatctggggcggtttctcggtaaataatgcaactcttacacggttcttcgcattccacttcctcctgccgttcgtcgttgcaggcgcaaccgtcctccacttactattcctacacgagacgggatcaaacaacccggccgggctaaattctgacgcagataaaatttctttccacccctacttctcctacaaagaccttctcggcttcgtgatcatgctgctggccctcacctcgctagcgctgttttcccccaacctcctaggtgatccagaaaattttaccccagcaaacccacttgtgacacccccacatattcaaccagagtgatacttcttatttgcatacgccatcctccggtctatccctaataaactaggcggggttcttgcactattattcagcatcctagtgctaatagttgtgccaattctacacacctcaaaacaacgaggactaactttccgccccgtgacacaattcctattttgaaccctagttgcagacataattatcttaacatgaattggaggcatgcccgtggaacacccatacattattattgggcaaatcgcatccctcttatactttgcgctcttccttattcttatcccactagcaggattaatagagaataaagcattgaaatgagctHM560067Alburnusescherichii             ATGGCAAGCCTACGAAAAACCCACCCACTAATAAAAATCGCTAATGACGCGCTAGTCGATCTTCCAACACCATCTAACATTTCAGCAATGTGAAATTTCGGATCCCTTCTAGGGTTGTGTTTAATTACCCAAATCCTAACAGGGTTATTCCTAGCCATACACTACACCTCCGACATCTCAACCGCATTCTCATCAGTCACTCATATTTGCCGAGACGTTAACTACGGCTGGCTCATTCGAAACCTACATGCCAACGGAGCATCCTTCTTCTTCATCTGCCTATATATGCATATCGCACGAGGTCTATATTACGGCTCATATCTTTATAAAGAAACCTGAAACATTGGCGTGGTACTATTTCTTCTGGTCATGATAACGGCCTTCGTGGGCTATGTACTCCCATGAGGACAAATATCCTTTTGAGGTGCTACCGTGATCACGAACCTCCTCTCAGCAGTTCCCTACATGGGAGATACCCTTGTTCAATGAATTTGGGGTGGTTTCTCAGTAGATAACGCGACTCTTACGCGATTCTTCGCGTTCCACTTCCTCCTGCCGTTCGTCGTTGCAGGCGCAACCGTCCTCCACTTACTATTCCTACACGAGACAGGATCAAACAACCCAGCCGGATTAAATTCTGACGCGGATAAAATTTCTTTCCATCCATACTTCTCCTACAAAGACCTTCTCGGCTTCGTAATCATGCTGCTGGCCCTCACCTCGCTGGCGCTATTTTCCCCCAACCTCCTAGGTGATCCAGAGAACTTTACCCCAGCAAACCCACTTGTGACACCCCCACATATTCAACCAGAGTGATACTTCTTGTTTGCATACGCCATCCTCCGGTCTATTCCTAATAAACTAGGCGGGGTTCTTGCACTATTGTTTAGTATTCTAGTGCTAATAGTTGTGCCAATTCTACGCACCTCAAAACAACGAGGACTAACTTTCCGCCCCGTGACGCAATTCCTATTTTGAACCCTAGTCGCAGATATGATTATCTTAACATGAATTGGGGGCATACCCGTAGAGCACCCATACATTATTATTGGTCAGGTCGCATCCGTCCTATACTTTGCACTCTTCCTTATCCTTATTCCACTAGCAGGATTAATAGAGAATAAAGCATTGAAATGAGCTHM560068Alburnusescherichii             ATGGCAAGCCTACGAAAAACCCACCCACTAATAAAAATCGCTAATGACGCGCTAGTCGATCTTCCAACACCATCTAACATTTCAGCAATGTGAAATTTCGGATCCCTTCTAGGGTNGTGTTTAATTACCCAAATCCTAACAGGGTTATTCCTAGCCATACACTACACCTCCGACATCTCAACCGCATTCTCATCAGTCACTCATATTTGCCGAGACGTTAACTACGGCTGGCTCATTCGAAACCTACATGCCAACGGAGCATCCTTCTTCTTCATCTGCCTATATATGCATATCGCACGAGGCCTATATTACGGNTCATATCTTTATAAAGAAACCTGAAACATTGGCGNGGNACTATTTCTTCTGGTCATGATAACGGCCTTCGNGGGCTATGTACTCCCATGAGGACAAATATCCTTTTGGGGTGCTACCGTAATCACCAACCTCCTCTCAGCAGNTCCCTACATGGGAGATACCCTTGTTCAATGAATTTGGGGCGGTTTCTCAGTAGATAACGCGACTCTTACGCGATTNNNNNNNNNCCACTTCCTCCNGCNGTTCGTCGTTGCAGGCGCAACCGTCCTCCACTTACTATTCCTNCACGAGACAGGATCAAACAACCCAGCCGGATTAAATTCTGACGCGGATAAAATTTCTTTCCATNCATACTTCTCCTACAAAGACCTTCTCGGCTTCGTAATCATGCTGCTGGCCCTCACCTCGCTGGCGCTATTTTCCCCCAACCTCCTAGGTGATCCAGAGAACTTTACCCCAGCAAACCCACTTGTGACACCCCCACATATTCGACCAGAGTGATACTTCTTGTTTGCATACGCCATCCTCCGGTCTATTCCTAATAAACTAGGCGGGGTTCTTGCACTACTGTTTAGTATTCTAGTGCTAATAGTTGTGCCAATTCTACACACCTCAAAACAACGAGGACTAACTTTCCGCCCCGTGACGCAATTCCTATTTTGAACCCTAGTCGCAGATATGATTATCTTAACATGAATTGGGGGCATACCNGTAGAGCACCCATACATTATTATTGGTCAGGTCGCATCCGTCCTATACTATGCACTCTTCCTTATCCTTATTCCACTAGCAGGATTAATAGAGAATAAAGCATTGAAATGAGCTHM560069Alburnusfilippii                ATGGCAAGCCTACGAAAAACCCACCCACTAATAAAAATCGCTAATGACGCACTAGTCGATCTTCCAACACCATCTAACATTTCAGCAATGTGAAATTTCGGATCCCTTCTAGGATTATGTTTAATTACCCAAATCCTAACAGGATTATTCCTGGCTATACACTACACCTCTGATATCTCAACCGCATTCTCATCAGTAACCCATATCTGCCGGGACGTTAACTACGGCTGACTCATTCGAAAACTGCATGCTAACGGAGCGTCCTTCTTCTTCATCTGCTTATATATGCATATCGCACGAGGGCTATATTACGGCTCATATCTTTATAAAGAAACCTGAAATATTGGGGTAGTACTATTTCTTCTGGTTATAATGACAGCCTTCGTGGGCTATGTACTTCCATGAGGACAAATATCCTTTTGAGGCGCTACCGTAATCACAAACCTCCTCTCAGCAGTCCCCTATATAGGAGACACCCTTGTTCAATGAATCTGAGGCGGTTTCTCAGTAGATAACGCAACCCTTACGCGATTCTTCGCATTCCACTTCCTCCTGCCATTCGTCGTTGCAGGCGCGACCATCCTCCACTTACTATTCCTACACGAAACAGGATCAAACAACCCGGCCGGACTGAACTCTGACGCGGACAAAATTTCTTTCCATCCATACTTCTCCTACAAGGACCTTCTCGGCTTCGTAATCATGCTGCTGGCCCTCACCTCCCTGGCGCTATTTTCCCCTAACCCTTTAGGTGATCCAGAAAACTTTACCCCAGCAAACCCACTCGTGACACCCCCACATATCCAGCCAGAATGATACTTCTTATTTGCATACGCCATCCTCCGGTCTATTCCTAATAAACTAGGCGGGGTTCTTGCACTACTATTTAGCATTCTAGTGCTAATGGTTGTGCCAATTCTACACACTTCAAAACAACGAGGACTAACCTTCCGTCCCGTGACACAATTCCTATTTTGAACCCTAGTTGCAGATATGATTATCTTAACATGAATTGGAGGCATACCCGTAGAGCACCCATACATTATTATTGGTCAAATCGCATCCGTCCTATACTTTGCACTCTTCCTTATTCTTATCCCCCTAGCAGGATTAATGGAAAATAAAGCATTGAAATGAGCTHM560070Alburnusfilippii                ATGGCAAGCCTACGAAAAACCCACCCACTAATAAAAATCGCTAATGACGCACTAGTCGATCTTCCAACACCATCTAACATTTCAGCAATGTGAAATTTCGGATCCCTTCTAGGATTATGTTTAATTACCCAAATCCTAACAGGATTATTCCTGGCTATACACTACACCTCTGATATCTCAACCGCATTCTCATCAGTAACCCATATCTGCCGGGACGTTAACTACGGCTGACTCATTCGAAATCTGCATGCTAACGGAGCGTCCTTCTTCTTCATCTGCTTATATATGCATATCGCACGAGGGCTATATTACGGCTCATATCTTTATAAAGAAACCTGAAATATTGGGGTAGTACTATTTCTTCTGGTTATAATGACAGCCTTCGTGGGCTATGTACTTCCATGAGGACAAATATCCTTTTGAGGCGCTACCGTAATCACAAACCTCCTCTCAGCAGTCCCCTATATAGGAGACACCCTTGTTCAATGAATCTGAGGCGGTTTCTCAGTAGATAACGCAACCCTTACGCGATTCTTCGCATTCCACTTCCTCCTGCCATTCGTCGTTGCAGGCGCGACCATCCTCCACTTACTATTCCTACACGAAACAGGATCAAACAACCCGGCCGGACTGAACTCTGACGCGGACAAAATTTCTTTCCATCCATACTTCTCCTACAAGGACCTTCTCGGCTTCGTAATCATGCTGCTGGCCCTCACCTCTCTGGCGCTATTTTCCCCTAACCTTTTAGGTGATCCAGAAAACTTTACCCCAGCAAACCCACTCGTGACACCCCCACATATCCAGCCAGAATGATACTTCTTATTTGCATACGCCATCCTCCGGTCTATTCCTAATAAACTAGGCGGGGTTCTTGCACTACTATTTAGCATTCTAGTGCTAATGGTTGTGCCAATTCTACACACTTCAAAACAACGAGGACTAACCTTCCGTCCCGTGACACAATTCCTATTTTGAACCCTAGTTGCAGATATGATTATCTTAACATGAATTGGAGGCATACCCGTAGAGCACCCATACATTATTATTGGTCAAATCGCATCCGTCCTATACTTTGCACTCTTCCTTATTCTTATCCCCCTAGCAGGATTAATGGAAAATAAAGCATTGAAATGAGCTAY026393Alburnushohenackeri             atggcaagcctacgaaaaacccacccactaataaaaatcgctaatgacgcgctagtcgatcttccaacaccatctaacatttcagcaatgtgaaatttcggatcccttctagggttgtgtttaattacccaaatcctgacagggttattcctagccatacactacacctccgatatctcaaccgcattctcatcagtcacccatatttgccgagacgttaactacggctggctcattcgaaacctacatgccaacggagcatccttcttcttcatctgcctatatatgcatatcgcacgaggtctatattacggctcatatctttataaagagacctgaaacattggggtagtactatttcttctggttatgataacagccttcgtgggctatgtactcccatgaggacaaatatccttttgaggtgctaccgtaatcacgaacctcctctcagcagttccctacatgggagatacccttgttcaatgaatttggggcggtttctcagtagataacgcgactcttacacgattcttcgcgttccacttcctcctgccgttcgtcgttgcaggcgcaaccgtcctccacttactattcctacacgagacaggatcaaacaacccagccgggttaaactctgacgcggataaaatttctttccacccatacttctcctacaaagaccttctcggcttcgtaatcatgctgctggccctcacctcgctggcgctattttcccccaacctcttaggtgatccagagaactttaccccagcaaacccacttgtgacacccccacatattcaaccagagtggtacttcctgtttgcatacgccatcctccggtctatccctaataaactaggcggggttcttgcactattatttagtattctagtgctaatagttgtgccaattctacacacctcaaaacaacgaggactaactttccgccccgtgacgcaattcctattttgaaccctagtcgcagatatgattatcttaacatgaattgggggcatgcccgtagagcacccatacattattattggtcagatcgcatccgtcctatactttgcactcttcctcatccttattccactagcaggattaatagagaataaagcattgaaatgagctHM560071Alburnuskostchyi                ATGGCAAGCCTACGAAAAACCCATCCACTAATAAAAATCGCTAATGACGCACTAGTCGATCTTCCAACACCATCTAACATCTCAGCAATATGAAACTTCGGGTCCCTTCTAGGATTATGTTTAATTACCCAAATCCTGACAGGATTATTCCTGGCCATACATTACACCTCAGATATCTCAACCGCATTCTCATCAGTAACCCACATCTGCCGAGACGTTAACTACGGCTGACTCATCCGAAACCTGCATGCTAACGGAGCATCCTTCTTCTTCATCTGCCTCTATATGCATATCGCACGAGGCCTATACTACGGCTCATATCTTTACAAAGAAACCTGAAATATTGGAGTAGTACTATTTCTTCTGGTCATGATGACAGCCTTCGTGGGCTACGTACTTCCATGAGGACAAATGTCCTTCTGAGGCGCCACCGTAATCACAAATCTCCTCTCAGCAGTCCCCTACATGGGAGACACCCTTGTTCAATGAATCTGGGGCGGCTTCTCAGTAGATAATGCAACCCTTACACGATTCTTCGCATTCCATTTCCTCTTACCATTCGTCGTTGCAGGCGCAACCGTCCTCCACTTATTATTCCTACACGAGACGGGGTCAAACAACCCGGCCGGACTGAACTCTGACGCGGACAAAATTTCTTTCCATCCGTACTTCTCATACAAAGACCTTCTCGGCTTCGTAATCATGTTGCTGGCCCTCACCTCTCTGGCGCTGTTTTCCCCCAACCTGTTAGGTGATCCGGAAAACTTTACCCCAGCAAACCCACTCGTGACACCCCCACATATCCAGCCAGAATGATACTTCTTATTTGCATATGCCATCCTCCGATCTATTCCTAATAAACTAGGCGGGGTTCTTGCACTATTATTCAGCATCCTAGTGCTAATGGTTGTGCCAATTCTACACACCTCGAAACAACGAGGACTAACTTTCCGTCCGGTGACCCAATTCCTATTCTGAACCCTAGTTGCAGACATGATTATTCTAACGTGAATTGGAGGCATACCCGTAGAACACCCATATATTATCATTGGTCAAGTCGCATCCGTCCTATACTTTGCACTCTTCCTTATTCTTATTCCACTAGCAGGATTAATGGAGAATAAAGCACTGAAATGAGCCHM560072Alburnuskostchyi                ATGGCAAGCCTACGAAAAACCCATCCACTAATAAAAATCGCTAATGACGCACCAGTCGATCTTCCAACACCATCTAACATCTCAGCAATATGAAACTTCGGGTCCCTTCTAGGATTATGTTTAATTACCCAAATCCTGACAGGATTATTCCTGGCCATACATTACACCTCAGATATCTCAACCGCATTCTCATCAGTAACCCACATCTGCCGAGACGTTAACTACGGCTGACTCATCCGAAACCTGCATGCTAACGGAGCATCCTTCTTCTTCATCTGCCTCTATATGCATATCGCACGAGGCCTATACTACGGCTCATATCTTTACAAGGAAACCTGAAATATTGGAGTAGTACTATTTCTTCTGGTCATGATGACAGCCTTCGTGGGCTACGTACTTCCATGAGGACAAATGTCCTTCTGAGGCGCCACCGTAATCACAAATCTCCTCTCAGCAGTCCCCTACATGGGAGACACCCTTGTTCAATGAATCTGGGGCGGCTTCTCAGTAGATAATGCAACCCTTACACGATTCTTCGCATTCCATTTCCTCTTACCATTCGTCGTTGCAGGCGCAACCGTCCTCCACTTATTATTCCTACACGAGACGGGGTCAAACAACCCGGCCGGACTGAACTCTGACGCGGACAAAATTTCTTTCCATCCGTACTTCTCATACAAAGACCTTCTCGGCTTCGTAATCATGTTGCTGGCCCTCACCTCTCTGGCGCTGTTTTCCCCCAACCTGTTAGGTGATCCGGAAAACTTTACCCCAGCAAACCCACTCGTGACACCCCCACATATCCAGCCAGAATGATACTTCTTATTTGCATATGCCATCCTCCGATCTATTCCTAATAAACTAGGCGGGGTTCTTGCACTATTATTCAGCATCCTAGTGCTAATGGTTGTGCCAATCCTACACACCTCGAAACAACGAGGACTAACTTTCCGTCCGGTGACCCAATTCCTATTCTGAACCCTAGTTGCAGACATGATTATTCTAACGTGAATTGGAGGCATACCCGTAGAACACCCATATATTATCATTGGTCAAGTCGCATCCGTCCTATACTTTGCACTCTTCCTTATTCTTATTCCACTAGCAGGATTAATGGAGAATAAAGCACTGAAATGAGCCAF090743Alburnusmacedonicus             atggcaagcctacgaaaaacccacccactaataaaaatcgctaatgacgcgctagtcgatcttccaacaccatctaacatttcagcaatgtgaaatttcggatcccttctagggttgtgtttaattacccaaatcctaacagggttattcctagccatacactacacctccgatatctcaaccgcattctcatcagtcacccatatttgccgagacgttaactacggctggctcattcgaaacctacatgccaacggagcatccttcttcttcatctgcctatatatgcatatcgcacgaggtctatattacggctcatatctttataaagagacctgaaacattggggtagtactatttcttctggttatgataacagccttcgtgggctatgtactcccatgaggacaaatatccttttgaggcgctaccgtgatcacgaacctcctctcagcagttccctacatgggagatacccttgttcaatgaatttggggcggtttctcagtagataacgcgactcttacacgattcttcgcgttccacttcctcctgccgttcgtcgttgcaggcgcaaccgtcctccacttactattcctacacgagacaggatcaaacaacccagccgggttaaactctgacgcggataaaatttctttccacccgtacttctcctacaaagaccttctcggcttcgtaatcatactgctggccctcacctcgctggcgctattttcccccaacctcttaggtgatccagagaactttaccccagcgaacccacttgtgacacccccacatatccaaccagagtggtacttcttgtttgcatacgccatcctccggtctattcctaataaactaggcggggttcttgcactattgtttagtattctagtgctaatagttgtgccaattctacacacctcaaaacaacgaggactaactttccgccccgtgacgcaattcctattttgaaccctagtcgcagatatgattatcttaacatgaattgggggcatgcccgtagagcacccatatattattattggtcaggtcgcatccgtcctatactttgcactcttccttatcctaattccactagcaggattaatagagaataaagcattgaaatgagctAY026394Alburnusmento                   atggcaagcctacgaaaaacccacccactaataaaaatcgctaatgacgcactagtcgatcttccaacaccatctaatatttcagcaatgtgaaatttcggatcccttctaggattatgtttaattacccaaatcctaacaggattattcctggctatacactacacctctgatatctcaaccgcattctcatcagtgacccatatctgccgagacgttaactacggctgactcattcgaaatctacatgctaacggagcatccttcttcttcatctgcttatatatgcatatcgcacgaggtctatattacggctcatatctttataaagaaacctgaaatattggggtagtactatttcttctggttataataacagccttcgtgggctatgtacttccatgaggacaaatatccttttgaggtgctaccgtaatcacaaacctcctctcagcagtcccctatataggagacacccttgttcaatgaatttgaggcggtttctcagtagataacgcaacccttacgcgattcttcgcattccacttcctcctgccgttcgtcgttgcaggcgcgactatcctccacttactattcctacacgaaacaggatcaaacaacccagccggactgaactctgacgcggacaaaatttctttccacccatatttctcctacaaggaccttctcggcttcgtaatcatgctgctggccctcacctctctggcgctattttcccctaaccttttaggtgatccagaaaactttaccccagcaaacccactcgtgacacccccacatatccagccagaatgatacttcttatttgcatacgccatcctccgatctattcctaataaactaggcggggttcttgcactactatttagcattctagtactaatagttgtgccaattctacacacttcaaaacaacgaggactaaccttccgtcccgtgacacaattcctattttgaaccctagtcgcagacatgattatcttaacatgaattggaggcatacccgtagagcacccatacattattattggtcaaatcgcatccatcctatactttgcactcttccttattcttatccccctagcaggattaatggaaaataaagcattgaaatgagctAY026391Alburnusorontis                 atggcaagcctacgaaaaacccacccactaataaaaattgctaatgacgcactagtcgatcttccgacaccatccaatatttcagcaatgtgaaacttcggatcccttctaggattatgtttaattacccaaatcctaacaggactattcctggctatacattacacctctgacatctcaaccgccttttcatcggtaacccacatctgccgagacgttaactacggctgactcattcgaaacctacatgccaacggagcatccttcttcttcatttgcctctacatacacatcgcacgaggcctatactacggctcatatctttataaagaaacctgaaatattggagtagtactatttcttctagttataatgaccgccttcgtgggctacgtacttccatgagggcaaatgtccttctggggcgccactgtcattacaaatcttctctcagcggtcccttatataggggacacccttgttcagtgaatctgagggggcttctcagtagataatgcaaccctcacacgattcttcgcattccacttcctcttaccattcgtcgttgcgggcgcaaccgtcctccatttattgttcctacacgaaacaggatcaaacaacccggccgggctaaattccgacgcggacaaaatttctttccacccatacttctcgtacaaagaccttctcggcttcgtaatcatattgctggccctcacctccctggcgctattttcgccaaacctattaggtgatccggagaactttaccccagcaaacccactcgtgacaccccctcatatccagccagaatgatatttcttgtttgcatacgccattctccgatctattcctaataaactaggcggggttcttgcactattattcagcattctagtgctaatggttgtgccgattctacacacctcaaaacaacgaggactaactttccgtccggtgactcaattcctgttttgaaccctggttgcagacataattattctgacatgaattggaggtatacccgtagaacacccatatatcattattggtcagatcgcatccctgctgtattttgcactcttccttgttcttatcccactagcaggattagtggaaaacaaagcattgaaatgagccAY026392Alburnussellal                  atggcaagcctacgaaaaacccacccactgataaaaatcgctaatggcgcactagtcgatcttccaacaccatctaatatctcagcaatgtgaaacttcgggtcccttctgggattatgtttaattacccaaatcctgacaggattattcctggccatgcattacacctcagatatctcaaccgcattctcatcagtaactcacatctgccgagacgttaactacggctgactcattcgaaacctacatgctaacggagcatccttcttcttcatctgcctctatatgcatatcgcacgaggtctatactacggctcatatctttacaaagaaacctgaaatattggagtagtactatttcttctggttatgatgacggccttcgtgggctacgtacttccgtgaggccaaatgtccttctggggcgccaccgtcatcacgaacctcctctcggcagtcccctacatgggggacacccttgtgcaatgaatctgaggtggcttctcagtagataatgcaaccctcacacggttcttcgcattccacttccttttaccattcgtcgttgcgggcgcaaccatcctccacttattgttcctacacgagacaggatcaaacaacccggccgggctaaactctgacgcggacaaaatttctttccacccatacttctcatacaaagaccttctcggcttcgtaatcatgttgctggccctcacctcgctggcgctattttcccccaacctattaggtgatccagaaaactttaccccagcaaacccactcgtgacacccccacatattcagccagaatgatacttcttgtttgcatacgccattctccgatctattcctaataaattaggcggggttcttgcactattattcagcatcctggtgctaatagttgtgccaattctacacacctcaaaacaacgaggactaaccttccgtccggtgacccaattcctattctgaaccctagttgcagacataattattctaacatgaattgggggcatacccgtagaacatccatatattatcattggtcaagtcgcatccgtcctatattttgcactctttctcattcttatcccactagcaggattaatggaaaataaagcattaaaatgagccAY83893Alburnussarmaticus               atggcaagcctacgaaaaacccacccactaataaaaatcgctaatgacgcactagtcgatcttccaacaccatctaatatttcagcaatatgaaatttcggatcccttctaggattatgtttaattacccaaatcctaacaggattattcctggctatacactacacctctgatatctcaaccgcattctcatcagtgacccatatctgccgagacgttaactacggctgactcattcgaaatctacatgctaacggagcatccttcttcttcatctgcttatatatgcanatcgcacgaggtctatattacggctcatatctttataaagaaacctgaaatattggggtngtactatttcttctggttataataacagccttcgtgggctatgtacttccatgaggacaaatatccttttgaggtgctaccgtaatcacaaacctcctctcagcagtcccctatataggagacacccttgttcaatgaatttgaggcggtttctcagtagataacgcaacccttacgcgattcttcgcattccacttcctcctgccgttcgtcgttgcaggcgcgactatcctccacttactattcctacacgaaacaggatcaaacaacccagccggactgaactctgacgcggacaaaatttctttccacccatatttctcctacaaggaccttctcggcttcgtaatcatgctgctggccctcacctctctggcgctattttcccctaaccttttaggtgatccagaaaactttaccccagcaaacccactcgtgacacccccacatatccagccagaatgatacttcttatttgcatacgccatcctccggtctattcctaataaactaggcggggttcttgcactactatttagcattctagtactaatagttgtgccaattctacacacttcaaaacaacgaggactaaccttccgtcccgtgacacaattcctattttgaaccctagtcgcagacatgattatcttaacatgaattggaggcatacctgtagagcacccatacattattattggtcaaatcgcatccatcctatactttgcactcttccttattcttatccccctagcaggattaatggaaaataaagcattgaaatgagctHM560066Alburnussp1                     ATGGCAAGCCTACGAAAAACCCGCCCACTAATAAAAATCGCTAATGACGCGCTAGTCGATCTTCCAACACCATCTAACATTTCAGCAATGTGAAATTTTGGATCCCTTCTAGGATTGTGTTTAATTACCCAAATCCTGACAGGGTTATTCCTGGCCATACACTACACCTCTGACATCTCAACCGCATTCTCATCAGTCACCCATATTTGCCGAGACGTTAACTACGGCTGGCTCATTCGAAACCTACATGCTAACGGAGCATCCTTCTTCTTCATCTGCCTATATATGCATATCGCACGAGGTCTATATTACGGCTCATATCTTTATAAAGAAACCTGAAACATTGGGGTCGTACTATTTCTTCTAGTTATGATAACAGCCTTCGTGGGCTATGTACTCCCATGAGGACAAATATCCTTTTGAGGTGCTACCGTAATCACAAACCTCCTCTCAGCAGTTCCCTACATAGGAGATACCCTTGTTCAATGAATTTGGGGCGGTTTCTCAGTAGATAACGCGACTCTTACACGATTCTTCGCGTTCCACTTCCTCCTGCCTTTCGTCGTTGCAGACGCAACCGTCCTCCACTTACTATTCCTACACGAGACAGGATCAAACAACCCAGCCGGGCTAAACTCTGACGCCGATAAAATTTCTTTCCACCCATACTTCTCCTACAAAGACCTTCTCGGCTTCGTGATCATGCTGCTGGCCCTCACCTCGCTGGCGCTATTTTCCCCCAACCTCCTAGGTGATCCAGAGAACTTTACCCCAGCAAACCCACTTGTGACACCCCCACATATCCAACCAGAGTGATACTTCTTGTTTGCATACGCCATCCTCCGGTCTATTCCTAATAAACTAGGCGGGGTTCTTGCACTATTGTTTAGTATTCTAGTGCTAATAGTTGTGCCAATTCTACACACCTCAAAACAACGAGGACTAACTTTCCGCCCCGTGACACAATTCCTATTTTGAACCCTAGTCGCAGATATGATTATCTTAACATGAATTGGGGGCATGCCCGTAGAGCACCCATACATTATTATTGGTCAGGTCGCATCCGTCCTATACTTTGCACTCTTCCTTATCCTTATCCCAATAGCAGGATTAATAGAGAATAAAGCATTGAAATGAGCTAF090745Alburnussp2                     atggcaagcctacgaaaaacccacccactaataaaaatcgctaatgacgcgctagtcgatcttccaacaccatctaacatttcagcaatatgaaatttcggatctcttctagggttatgtttaattacccaaatcctaacagggctattcctagccatacactacacctccgatatctcaaccgcattctcatcagtcacccatatttgccgagacgttaactacggctggctcattcgaaacctacatgccaacggagcatccttcttcttcatctgcctgtatatgcatatcgcacggggtctatattacggctcatatctttataaagagacctgaaacattggggtagtactatttcttctggttatgataacagccttcgtgggctatgtgctcccatgaggacaaatatccttttgaggtgctaccgtaatcacgaatctcctctcagcagttccctacatgggagatacccttgttcaatgaatttggggcggtttctcagtagataacgcaactcttacacgattcttcgcgttccacttcctcctgccgttcgtcgttgcaggcgcaaccgtcctccacttactattcctacacgagacaggatcaaacaacccagccgggttaaactctgacgcggataaaatttctttccacccatacttctcctacaaagaccttctcggcttcgtaatcatgctgctggccctcacctcgctggcgctattttcccccaacctcctaggtgatccagagaactttaccccagcaaacccccttgtgacgcccccacatatccaaccagagtgatacttcttgtttgcatacgccatcctccggtctattcctaataaactaggcggggttcttgcactattgttcagtattctagtgctaatagttgtgccaattctacacacctcaaaacaacgaggactaactttccgccccgtgacgcaattcctattttgaaccctagtcgcagatatgattatcttaacatgaattgggggcatgcccgtagagcacccatacattattattggtcaggtcgcatccgtcctatactttgcactcttccttatccttattccactagcagggttaatagagaataaagcattgaaatgagctAF090744Alburnusthessalicus             atggcaagcctacgaaaaacccacccactaataaaaatcgctaatgacgcgctagtcgatcttccaacaccatctaacatttcagcaatgtgaaatttcggatcccttctagggttgtgtttaattacccaaatcctaacagggttattcctagccatacactacacctccgatatctcaaccgcattctcatcagtcacccatatttgccgagacgttaactacggctggctcattcgaaacctacatgccaacggagcatccttcttcttcatctgcctatatatgcatatcgcacgaggtctatattacggctcatatctttataaagagacctgaaacattggggtagtactatttcttctggttatgataacagccttcgtgggctatgtactcccatgaggacaaatatccttttgaggcgctaccgtgatcacgaacctcctctcagcagttccctacatgggagatacccttgttcaatgaatttggggcggtttctcagtagataacgcgactcttacacgattcttcgcgttccacttcctcctgccgttcgtcgttgcaggcgcaaccgtcctccacttactattcctacacgagacaggatcaaacaacccagccgggttaaactctgacgcggataaaatttctttccacccgtacttctcctacaaagaccttctcggcttcgtaatcatactgctggccctcacctcgctggcgctattttcccccaacctcttaggtgatccagagaactttaccccagcgaacccacttgtgacacccccacatatccaaccagagtggtacttcttgtttgcatacgccatcctccggtctattcctaataaactaggcggggttcttgcactattgtttagtattctagtgctaatagttgtgccaattctacacacctcaaaacaacgaggactaactttccgccccgtgacgcaattcctattttgaaccctagtcgcagatatgattatcttaacatgaattgggggcatgcccgtagagcacccatatattattattggtcaggtcgcatccgtcctatactttgcactcttccttatcctaattccactagcaggattaatagagaataaagcattgaaatgagctHM560073Anaecyprishispanica             ATGGCAAGCCTACGAAAAACCCACCCACTGATAAAAATCGCTAATGACGCACTAGTCGACCTTCCAACACCATCTAACATCTCCGCAATGTGAAACTTTGGATCCCTTCTAGGACTATGTTTGATTACCCAAATCCTGACGGGGTTATTTCTAGCCATACATTATACCTCTGATATCTCAACCGCATTCTCATCAGTAACCCATATCTGCCGAGACGTTAACTACGGTTGGCTCATTCGAAATCTACATGCCAACGGAGCATCCTTCTTCTTCATCTGCCTCTATATGCATATCGCACGAGGCCTGTATTACGGCTCATATCTTTACAAGGAAACCTGAAATATTGGGGTAGTACTATTTCTTCTGGTTATAATGACCGCCTTCGTGGGCTATGTACTTCCATGAGGACAAATATCCTTTTGAGGTGCCACCGTAATTACAAACCTCCTCTCAGCAGTCCCATACATAGGAGATACTCTTGTTCAATGAATCTGAGGCGGCTTCTCGGTAGATAATGCAACCCTAACACGATTCTTCGCATTCCATTTCCTCTTACCATTCGTGGTTGCAGGCGCAACCGTCCTCCACTTACTATTCCTGCACGAAACCGGATCAAACAACCCGGCCGGATTAAACTCCGACGCGGACAAAATTTCCTTCCACCCGTACTTCTCCTACAAAGACCTTCTCGGCTTCGTAATCATATTACTGGCCCTCACCTCCCTGGCGCTATTTTCCCCCAACCTCTTAGGTGATCCGGAAAACTTTACCCCAGCGAACCCACTTGTAACACCTCCACATATTCAGCCAGAGTGGTACTTCTTATTTGCATACGCCATCCTCCGATCTATTCCTAATAAACTAGGCGGAGTTCTTGCACTATTGTTCAGCATTCTAGTGCTAATAGTTGTGCCCACTTTACATACCTCAAAACAACGAGGACTAACTTTCCGGCCCGTAACTCAATTTCTGTTCTGAACCCTGGTTGCAGATATGATTATCTTAACATGAATTGGAGGCATGCCCGTAGAGCACCCGTACATTATTATTGGTCAAATCGCATCCGTCCTATACTTTGCACTCTTCCTTATTCTTATCCCCATAGCAGGATTAATGGAAAATAAAGCATTGAAATGAGCCHM560074Anaecyprishispanica             ATGGCAAGCCTACGAAAAACCCATCCACTGATAAAAATCGCTAATGACGCACTAGTCGATCTTCCAACACCATCTAACATCTCCGCAATATGAAACTTTGGATCCCTTCTAGGACTATGTTTGATTACCCAAATCCTGACGGGGTTATTTCTGGCCATACATTATACCTCTGATATCTCAACCGCATTCTCATCAGTAACCCATATCTGCCGAGACGTTAACTACGGCTGGCTCATTCGAAATCTACATGCCGACGGAGCATCCTTCTTCTTCATTTGCCTCTATATGCATATCGCACGAGGCCTGTATTACGGCTCATATCTTTATAAGGAAACCTGAAATATTGGGGTAGTACTATTTCTTCTGGTTATAATGACCGCCTTCGTGGGCTATGTACTTCCATGAGGACAAATATCCTCTTGAGGTGCCACCGTAATTACAAACCTCCTCTCAGCAGTCCCATACATAGGAGATACTCTTGTTCAATGAATCTGAGGCGGCTTCTCGGTAGATAATGCAACCCTAACACGATTCTTCGCATTCCACTTCCTCTTACCATTCGTAGTTGTAGGCGCAACTGTCCTCCACTTACTATTCCTGCACGAAACCGGATCAAACAACCCGGCCGGATTAAACTCCGACGCGGACAAAATTTCCTTCCACCCGTACTTCTCCTACAAAGACCTTCTCGGCTTCGTAATCATATTACTGGCCCTCACCTCCCTGGCACTATTTTCCCCCAACCTCTTAGGTGATCCGGAAAACTTTACCCCAGCAAACCCACTTGTAACACCTCCACATATCCAGCCAGAGTGGTACTTCTTATTCGCATACGCCATCCTCCGATCCATTCCTAATAAACTAGGCGGAGTTCTTGCACTATTATTCAGCATTCTAGTGCTAATAGTTGTGCCCATTTTACATACCTCAAAACAACGAGGACTAACTTTCCGACCCGTAACTCAATTTCTGTTCTGAACCCTAGTTGCAGATATGATTATCTTAACATGAATTGGAGGCATACCCGTAGAGCACCCGTACATTATTATTGGTCAAATCGCATCCGTCCTATACTTTGCACTCTTCCTTATTCTTATCCCCATAGCAGGATTAATGGAAAATAAAGCATTGAAATGAGCCHM560075Leuciscusaspius                 ATGGCAAGCCTACGAAAAACCCACCCGCTAATAAAAATCGCTAATGATGCACTAGTCGACCTTCCAACCCCATCTAACATTTCAGCGCTTTGAAACTTCGGATCTCTCCTAGGATTATGTTTGATTACTCAAATCCTGACGGGATTATTCTTAGCCATGCACTACACCTCCGATATTTCAACTGCATTCTCATCGGTAACTCACATCTGCCGGGACGTTAACTACGGCTGACTTATCCGGAATCTACACGCTAACGGAGCGTCATTTTTCTTCATCCGTATTTATATGCATATCGCACGGGGCCTATATTACGGGTCCTATCTCTATAAGGAGACCTGAGACATTGGTGTAGTACTACTTCTCCTAGTTATAATGACCGCCTTCGTGGGTTATGTACTTCCATGAGGTCAAATGTCTTTTTGAGGCGCTACCGTTATTACAAACCTCCTATCAGCAGTCCCTTATATGGGCGACACCCTCGTTCAATGAATTTGAGGCGGCTTCTCTGTAGACAACGCGACCCTCACCCGGTTCTTCGCGTTCCACTTCCTACTACCGTTCGTCATCGCCGGCGCGACCGTCCTCCATTTACTATTTTTACACGAAACAGGGTCAAACAACCCAGCCGGACTAAATTCCGACGCGGACAAAATTTCCTTCCACCCATACTTCTCGTATAAGGACCTTCTTGGCTTTGTGATCATGCTACTAGCCCTCACCTCTCTGGCGCTATTTTCCCCTAACCTGTTAGGTGATCCAGAAAATTTTACCCCAGCAAACCCACTCGTAACGCCCCCACATATTCAGCCAGAGTGGTACTTCCTGTTTGCCTACGCCATCCTCCGGTCTATCCCAAACAAGCTAGGCGGGGTTCTTGCACTACTATTTAGCATTCTTGTGCTAATAGTTGTGCCAATCTTACACACCTCAAAACAACGAGGATTAACTTTCCGACCTGTAACCCAATTCCTATTCTGAACCCTAGTTGCAGATATGATTATCTTAACATGAATTGGGGGCATGCCCGTAGAACACCCATATATCATTATTGGCCAAATCGCATCCATTCTATACTTTGCACTCTTCCTCGTTCTTGTCCCACTAGCAGGGTGAGTAGAAAACAAAGCATTAAAATGAGCCAY026399Leuciscusvoraxvorax             atggcaagcctacgaaaaacccatccactaataaaaatcgctaatgatgcactagttgaccttccaaccccatccaatatttcggcactctgaaacttcggatccctcctaggattatgtttaattacccagatcctcacgggactattcttagccatgcactacacctccgatatttcaaccgcattctcatcagtaactcacatctgccgggacgttaactacggctggcttatccgaaacctacacgctaacggagcatcctttttcttcatttgtatttatatacatattgcacgaggcctatattacgggtcctatctttacaaggagacctgaaacattggtgtcgtactactcctcctagttataataaccgcgtttgtaggttatgtactcccgtgaggtcaaatgtctttttgaggtgccaccgttatcacgaacctcctatcagcagtcccttacataggcgacacccttgttcaatgaatttgaggcggcttctcggtcgacaacgcaaccctcacccgattcttcgcgtttcacttcctcctgccattcgtcattgccggcgcaaccatcctacatttactatttctacacgagacaggatcgaacaacccagccgggttgaactctgacgcagataaaatttcattccacccatacttctcgtacaaagaccttcttggcttcgtaatcatactactagctctcacctcgctagcgctgttctcccctaacctactaggtgacccagaaaactttactccagcaaacccactcgtgacacccccacacattcagccagaatgatacttcttatttgcctacgccattctccgatctatcccaaacaaactgggaggggttcttgcactaatatttagcattctagtactaatggttgtgccaatcttacacacatctaaacaacgaggactaactttccgacctgtaacccaattcctattctgaaccctcgttgcagatatgattatcctaacatgaatcggaggcatacccgtagaacatccatatatcattattggccaaatcgcatccattctatattttgcactattccttattcttatcccactagcgggatgagttgagaataaagcattgaaatgagccAY026409Ballerusballerus                atggcaagcctacgaaaaacccacccactaataaaaatcgctaatgacgcactagtcgacctcccaacaccatctaatatttcagcattatgaaacttcgggtccctcctaggattatgtctaattacccaaatccttacaggactatttctagccatacactacacctctgatatctcaaccgcattttcatcagtaaaccatatttgtcgagacgtcaactacggctggcttatccgaagcctgcatgctaacggagcatcattctttttcatctgtctttatatacatatcgcacgaggcctatactacggatcatatctttataaagaaacctgaaacattggtgtagtcctatttcttctagttataataacagcctttgttggctatgtactcccatgaggacaaatatccttttgaggtgctaccgtaattacaaacctcctatcagcagtcccttatataggagatactcttgttcaatgaatctgaggtggtttctcagtagacaacgcaactctcacacgattcttcgcattccacttcctcctgccatttgtcgtcgccggcgcaactatcctacacctattatttctacacgaaacaggatcgaacaacccagccggactaaactccgacgcagacaaaatctccttccacccgtacttttcatataaagatctttttggttttgtaatcatactactagcccttacttctctggcattattttcacccaacctactaggtgacccagaaaatttcaccccagcaaacccgctcgtgacacccccacatatccagccagaatgatacttcttatttgcttacgccattctccgatctattccaaataaactgggaggggttcttgcactattattcagcatcctggtgctaatagttgtgccaattttacacacctcaaaacaacgaggactaactttccgtcccctaacccaattcttattctgaaccctggttgcagatataattattttaacatgaattgggggcatacccgtagaacacccatacattattattggccaagccgcatccattctatactttgcactcttcctcattcttattccactagcaggatgagtagaaaacaaagcactgaaatgagccAY026408Ballerussapa                    atggcaagcctacgaaaaacccacccactaataaaaatcgctaatgacgcactagtcgacctcccaacaccatctaatatttcaacattatgaaacttcggatccctcctaggactatgtttaattacccaaatccttacgggactatttctagccatacactacacctctgatatctcaaccgcattttcatcagtaaaccatatttgccgagacgtcaactacggctggctcatccgaagcctacatgctaatggagcatcattcttcttcatctgtctttatatacatattgcacgaggcctgtactacggatcatatctttacaaagaaacctgaaacattggtgtagtcctatttcttctagttataatgacagcctttgttggctatgtactcccatgaggacaaatatccttttgaggtgctaccgtgattacaaatctcctctcagcagtcccttatataggagatactcttgttcaatgaatctgaggcggtttctcagtagacaacgcaactctcacacgattcttcgcattccacttcctcctgccattcgtcgtcgccggcgcaaccatcctacacttactattcctacacgaaacaggatcaaacaacccaaccggattaaactccgacgcagacaaaatttccttccacccgtatttctcctataaggaccttcttggctttgtaattatgttactagccctcacttctctagcattattttcacccaacctattaggtgacccagaaaatttcaccccagcaaacccactcgtgacacccccacatattcagccagaatgatacttcttatttgcttacgccattctccgatcaattccaaataaactaggaggggttcttgcactattattcagcattctagtgctaatagtcgtgccaattttacatacctcaaaacaacgaggactaactttccgtcccctaacccaatttttattttgaaccctagttgcagatatactcattctaacatggattggaggcatacccgtagaacatccatatatcattattggccaagccgcatccattctatactttgcactcttcctcattcttatcccactggcaggatgaatagaaaacaaagcactgaaatgagccHM560076Bliccabjoerkna                  ATGGCAAGCCTACGAAAAACCCACCCACTAATAAAAATCGCTAATGACGCACTAGTCGACCTTCCAACACCATCTAACATTTCAGTATTATGAAACTTCGGGTCCCTCCTAGGATTGTGTTTAATTACCCAAATCCTCACGGGATTATTTCTAGCCATACATTACACCTCTGATATTTCCACCGCATTTTCATCAGTAACCCACATCTGCCGAGACGTTAACTACGGCTGGCTCATTCGAAATTTACACGCTAACGGAGCATCATTCTTCTTCATCTGTCTTTATATACATATTGCACGAGGCCTATACTACGGATCATATCTTTACAAAGAAACCTGAAATATTGGTGTAGTCCTATTTCTTCTAGTTATAATGACAGCCTTCGTCGGCTATGTACTTCCATGAGGGCAGATATCTTTTTGAGGTGCCACCGTAATTACAAACCTCCTCTCAGCAGTCCCTTATATAGGCGACACCCTCGTCCAATGAATCTGAGGCGGTTTCTCAGTAGATAACGCAACTCTCACACGATTCTTCGCATTCCACTTCCTTCTACCATTCGCTGTCGCCGGCGCAACCCTCTTACACCTACTATTCCTACACGAAACAGGGTCGAACAACCCAGCCGGCTTAAACTCCGACGCAGATAAAATCTCCTTCCACCCGTACTTCTCATATAAAGACCTTCTTGGCTTTGTAATCATATTACTGGCCCTTACTTCCCTGGCATTATTTTCCCCCAACCTACTAGGTGACCCAGAAAATTTTACCCCAGCAAACCCACTCGTGACACCCCCACATATTCAGCCAGAGTGATATTTCCTATTTGCCTACGCCATTCTCCGATCCATTCCAAATAAACTAGGAGGGGTCCTTGCATTATTATTCAGTATTTTAGTGCTAATAGTTGTGCCAATCTTACATACCTCAAAACAACGAGGACTAACTTTCCGCCCTATAACGCAATTCTTATTTTGAACCTTAGTTGCAGACATAATCATCCTGACATGAATCGGAGGCATACCCGTAGAACACCCATATATTATTATTGGCCAAGTCGCATCCATTTTATATTTTGCACTCTTCCTCATTCTTATCCCGCTAGCAGGATGAATAGAGAACAAAGCACTGAAATGAGCCHM560077Bliccabjoerkna                  ATGGCAAGCCTACGAAAAACCCACCCACTAATAAAAATCGCTAATGACGCACTAGTCGACCTTCCAACACCATCTAACATTTCAGTATTATGAAACTTCGGGTCCCTCCTAGGATTGTGTTTAATTACCCAAATCCTCACGGGATTATTTCTAGCCATACATTACACCTCTGATATTTCCACCGCATTTTCATCAGTAACCCACATCTGCCGAGACGTTAACTACGGCTGGCTCATTCGAAATTTACACGCTAACGGAGCATCATTCTTCTTCATCTGTCTTTATATACATATTGCACGAGGCCTATACTACGGATCATATCTTTACAAAGAAACCTGAAATATTGGTGTAGTCCTATTTCTTCTAGTTATAATGACAGCCTTCGTCGGCTATGTACTTCCATGAGGGCAGATATCTTTTTGAGGTGCCACCGTAATTACAAACCTCCTCTCAGCAGTCCCTTATATAGGCGACACCCTCGTNCAANGAATCTGAGGCGGTTTCTCAGTAGATAACGCAACTCTCACACGATTCTTCGCATTCCACTTCCTTCTACCATTCGTTGTCGCCGGCGCAACCCTCTTACACCTACTATTCCTACACGAAACAGGGTCGAACAACCCAGCCGGCTTAAACTCCGACGCAGATAAAATTTCCTTCCACCCGTACTTCTCATATAAAGACCTTCTTGGCTTTGTAATCATATTACTGGCCCTTACTTCCCTGGCATTATTTTCCCCCAACCTACTAGGTGACCCAGAAAATTTTACCCCAGCAAACCCACTCGTGACACCCCCACATATTCAGCCAGAGTGATATTTCCTATTTGCCTACGCCATTCTCCGATCCATTCCAAATAAACTNGGAGGGGACCTTGCATTATTATTCAGTATTTTAGTGCTAATAGTTGTGCCAATCTTACATACCTCAAAACAACGAGGACTAACTTTCCGCCCTATAACGCANTTCTTATTTTGAACCTTAGTTGCAGACATAATNATCCTGACATGAATCGGANGNNTNCCCGTAGAACNCCCATATATTATGATTGGCCANNNNGNCTNNATTNNATATTTTGCNCTCTTCCTCATNCTTATNCNNNNNNNNNNNNNNNNNNNNNNNNNNNNNNNNNNNNNNNNNHM560078Chondrostomaangorense           ATGGCAAGCCTACGAAAAACCCACCCACTAATAAAAATCGCTAACGACGCGCTAGTCGACCTCCCAACACCATCTAATATCTCAGTAATGTGAAACTTCGGATCTCTCCTAGGATTGTGTTTAATTACCCAAATCCTGACAGGATTATTCTTAGCCATACATTACACCTCTGATATCTCAACCGCATTCTCATCAGTGACCCACATCTGCCGAGATGTCAACTACGGCTGACTTATCCGAAGCCTGCATGCCAATGGAGCATCCTTTTTCTTCATCTGTCTTTACATACATATCGCACGGGGCCTATATTATGGGTCATACCTTTATAAAGAAACCTGAAACATCGGTGTAGTCCTATTCCTTCTGGTTATGATAACGGCCTTTGTAGGCTACGTTCTTCCGTGGGGACAAATATCCTTTTGAGGTGCTACCGTAATTACAAACCTCCTCTCTGCCGTCCCCTACATGGGAGATACCCTTGTTCAATGAATCTGAGGCGGCTTCTCAGTAGACAATGCGACTCTCACACGATTCTTCGCATTTCACTTCCTACTACCCTTTGTAGTTGCCGGCGCAACCATCCTGCACTTGTTGTTTTTACACGAAACGGGGTCGAACAACCCGGCCGGACTAAATTCAGACGCCGACAAAATTTCTTTCCACCCGTACTTCTCATATAAAGATCTCCTTGGTTTTGTGGCAATATTACTAGCCCTTACCTCTCTAACATTATTTTCCCCTAACCTATTAGGTGACCCGGAAAACTTTACCCCAGCAAACCCGCTCGTAACACCACCGCATATCCAGCCAGAATGATACTTCTTATTTGCCTACGCCATCCTTCGATCCATCCCAAATAAACTAGGAGGGGTTCTTGCACTACTATTCAGCATCCTGGTGCTATTAGTCGTGCCAATTTTACACACATCCAAACAACGAGGACTAACTTTCCGCCCAGTGACCCAATTCCTATTCTGAACCCTGGTTGCAGATATATTTATTTTGACATGAATCGGGGGTATACCCGTAGAACACCCATACATTGTTATTGGCCAAGTCGCATCCATCCTATACTTTGCACTATTCCTCATTCTTGTCCCACTAGCAGGATGAGTGGAAAATAAAGCATTGAAATGAGCCAF533760Chondrostomaangorense           ATGGCAAGCCTACGAAAAACCCACCCACTAATAAAAATCGCTAACGACGCGCTAGTCGACCTCCCAACACCATCTAATATCTCAGTAATGTGAAACTTCGGATCTCTCCTAGGATTATGTTTAATTACCCAAATTCTGACAGGATTATTCTTAGCCATACATTACACCTCTGATATCTCAACCGCATTCTCATCAGTGACCCACATCTGCCGAGATGTCAACTACGGCTGACTTATCCGAAGCCTGCATGCCAATGGAGCATCCTTTTTCTTCATCTGTCTTTACATGCATATCGCACGGGGCCTATATTATGGATCATACCTTTATAAAGAAACCTGAAACATCGGTGTAGTCCTATTTCTTCTGGTTATGATAACGGCCTTTGTAGGCTACGTTCTTCCGTGGGGACAAATATCCTTTTGAGGTGCTACCGTGATTACAAACCTCCTCTCTGCCGTCCCCTACATGGGGGATACCCTTGTTCAATGAATCTGAGGCGGCTTCTCAGTAGACAATGCAACTCTCACACGGTTCTTCGCATTCCACTTCCTACTACCCTTTGTAGTTGCCGGCGCAACCATCCTACACTTGTTGTTTTTACACGAAACGGGGTCGAACAACCCGGCCGGACTAAATTCAGACGCCGACAAAATTTCTTTCCACCCGTACTTCTCATATAAAGATCTCCTTGGTTTTGTGGCAATATTACTAGCCCTTACCTCTCTGACATTATTTTCCCCTAACCTATTAGGTGACCCGGAAAACTTTACCCCAGCAAACCCGCTCGTAACACCACCGCATATCCAGCCAGAATGATACTTCTTATTTGCCTACGCCATCCTTCGGTCTATCCCAAATAAACTAGGAGGGGTTCTTGCACTACTATTCAGCATCCTAGTGCTATTAGTCGTGCCAATTTTACACACATCCAAACAACGAGGACTAACTTTCCGCCCAGTGACCCAATTCCTATTCTGAACCCTGGTTGCAGATATATTTATTTTGACATGAATCGGGGGTATACCCGTAGAACACCCATACATTGTTATTGGCCAAGTCGCATCCATCCTATACTTTGCACTATTCCTCATTCTTGTCCCACTAGCAGGATGGGTGGAAAATAAAGCATTGAAATGAGCCAY026401Condrostomahcyri                atggcaagcctacgaaaaacccacccactaataaaaatcgctaacgacgcgctagtcgacctcccaacaccatctaatatctcagtgatgtgaaacttcggatctctcctaggattatgtttgattacccaaatcctgacaggattattcttagccatacattacacctctgatatctcaaccgcattctcatcagtgacccacatctgccgagatgtcaactacggctgacttatccgaagcctgcatgccaatggagcatcctttttcttcatctgtctttacatacatatcgcacggggcctatattatggatcatacctttataaagagacctgaaacatcggtgtagtcctattccttctggttatgataacggcctttgtgggctacgttcttccatggggacaaatatccttttgaggtgctaccgtaattacaaacctcctctctgccgtcccatacataggggatacccttgttcaatgaatctgaggcggcttctcagtagacaatgcgactctcacacggttcttcgcattccacttcctactaccctttgtagttgccggcgcaaccatcctacacttgttgtttttacacgaaacgggatcgaacaacccggccggactaaattcagacgccgacaaaatttctttccacccgtacttctcatacaaagatctccttggttttgtggcaatattactagcccttacctccctaacattattttcccctaacctattaggtgacccagaaaactttaccccagcaaacccgctcgtaacaccgccacatattcagccagagtgatacttcttatttgcctacgccatccttcgatccatcccaaataaactaggaggggttcttgcactactattcagcatcctagtgctattagtcgtgccaattttacacacatccaaacaacgaggactaactttccgcccggtgacccaattcctattctgaaccctggttgcagatatatttattttgacatgaatcgggggtatacccgtagaacacccatacattgttattggccaagtcgcatccatcctatactttgcactatttctcattcttgtcccactagcaggatgagtggaaaataaagcattgaaatgagccAF533765Chondrostomaholmwoodii          atggcaagcctacgaaaaacccacccgctaataaaaatcgctaacgacgcgctagtcgacctcccaacaccatctaacatctcagtgatatgaaacttcggctctctcctaggattgtgtttaattacccaaatcctgacaggattattcttagccatacattacacctctgatatctcaaccgcattctcatcagtgacccacatctgccgagatgtcaactacggctgacttatccgaagcctgcatgccaatggagcatcctttttcttcatctgtctttacatacacatcgcacggggcctatattatgggtcatacctttataaagagacctgaaatatcggtgtagtcctattccttctggtcatgataacggcctttgtaggctacgttcttccgtggggacaaatatccttttgaggtgctaccgtaattacaaacctcctttctgccgtcccttacataggggatacccttgttcaatgaatctgaggcggcttctcagtagacaatgtgactctcacacggttcttcgcattccacttcctgctgccctttgtagttgccggcgcaaccatcctacacttgttgtttttacacgaaacgggatctaacaacccggccggactaaattcagacgccgacaaaatttctttccacccgtacttctcatataaagatctccttggttttgtggcaatattactagcccttacctctttaacattattttcccctaacctattaggtgacccggaaaactttacgccagcaaacccgctcgtaacaccaccgcatattcagccagaatgatacttcttattcgcctacgccatccttcgatccatcccaaataaactaggaggggttcttgcactactattcagcatcctagtgctattagtcgtgccaattttacacacatccaaacaacgaggactaactttccgcccagtgacccaattcctattctgaaccctggttgcagatatatttattttgacatgaatcgggggtatacccgtagaacacccatacattgttattggccaagtcgcatccatcctatactttgcactgttcctcgttcttgtcccactagcaggatgaatggaaaataaagcattgaaatgagccHM560079Chondrostomaknerii              ATGGCAAGCCTACGAAAAACCCACCCACTAATAAAAATCGCTAACGACGCGCTAGTCGACCTCCCAACACCATCTAATATCTCAGTAATGTGAAACTTCGGGTCTCTCCTGGGATTGTGTTTGATTACCCAAATCCTAACAGGATTATTCCTAGCCATGCATTACACCTCTGATATCTCAACCGCATTCTCATCAGTAACCCACATCTGCCGTGATGTTAACTACGGCTGACTTATCCGAAGCCTACATGCCAATGGAGCATCCTTTTTCTTCATCTGTCTTTATATGCATATCGCACGGGGCCTGTATTATGGATCATACCTCTATAAAGAGACCTGAAACATTGGTGTAGTCCTATTCCTCCTGGTTATAATAACAGCCTTTGTAGGCTACGTTCTTCCATGAGGACAAATATCCTTTTGAGGTGCTACCGTGATTACAAACCTCCTCTCTGCAGTCCCATACATAGGGGATACCCTTGTGCAATGAATCTGAGGCGGCTTCTCAGTAGACAATGCGACTCTTACACGATTCTTCGCATTCCACTTCCTACTGCCATTCGTCGTTGCCGGCGCAACCATCCTGCACTTATTATTTTTACACGAAACGGGATCTAACAACCCGGCCGGATTAAATTCCGACGCAGACAAAATTTCTTTCCACCCATATTTCTCATATAAAGACCTTCTTGGTTTTGTGGCCATGTTACTAGCCCTTACCTCCCTAACATTATTTTCTCCTAACCTACTAGGTGACCCGGAAAACTTTACCCCAGCAAACCCACTAGTAACGCCCCCACATATCCAGCCAGAATGATACTTCTTATTTGCCTACGCCATCCTTCGATCTATCCCAAATAAACTAGGAGGGGTTCTTGCACTACTATTTAGCATTCTAGTGCTATTAGTCGTGCCAATTTTACACACATCCAAACAACGAGGACTAACTTTCCGCCCGGTGACCCAATTCTTATTCTGAACCCTGGTTGCAGATATATTTATTCTGACATGAATCGGGGGCATACCCGTAGAGCACCCATATATTATTATTGGCCAAGTCGCATCCATCCTATACTTTGCATTATTCCTCATTCTCGTCCCACTAGCAGGATGGGTAGAAAATAAAGCATTGAAATGAGCCHM560080Chondrostomaknerii              ATGGCAAGCCTACGAAAAACCCACCCACTAATAAAAATCGCTAACGACGCGCTAGTCGACCTCCCAACACCATCTAATATCTCAGTAATGTGAAACTTCGGGTCTCTCCTGGGATTGTGTTTGATTACCCAAATCCTAACAGGATTATTCCTAGCCATGCATTACACCTCTGATATCTCAACCGCATTCTCATCAGTAACCCACATCTGCCGTGATGTTAACTACGGCTGACTTATCCGAGGCCTACATGCCAATGGAGCATCCTTTTTCTTCATCTGTCTTTATATGCATATCGCACGGGGCCTGTATTATGGATCATACCTCTATAAAGAGACCTGAAACATTGGTGTAGTCCTATTCCTCCTGGTTATAATAACAGCCTTTGTAGGCTACGTTCTTCCATGAGGACAAATATCCTTTTGAGGTGCTACCGTGATTACAAACCTCCTCTCTGCAGTCCCATACATAGGGGATACCCTTGTGCAATGAATCTGAGGCGGCTTCTCAGTAGACAATGCGACTCTTACACGATTCTTCGCATTCCACTTCCTACTGCCATTCGTCGTTGCCGGCGCAACCATCCTGCACTTATTATTTTTACACGAAACGGGATCTAACAACCCGGCCGGATTAAATTCCGACGCAGACAAAATTTCTTTCCACCCATATTTCTCATATAAAGACCTTCTTGGTTTTGTGGCCATGTTACTAGCCCTTACTTCCCTAACATTATTTTCTCCTAACCTACTAGGTGACCCGGAAAACTTTACCCCAGCAAACCCACTAGTAACGCCCCCACATATCCAGCCAGAATGATACTTCTTATTTGCCTACGCCATCCTTCGATCTATCCCAAATAAACTAGGAGGGGTTCTTGCACTACTATTTAGCATTCTAGTGCTATTAGTCGTGCCAATTTTACACACATCCAAACAACGAGGACTAACTTTCCGCCCGGTGACCCAATTCTTATTCTGAACCCTGGTTGCAGATATATTTATTCTGACATGAATCGGGGGCATACCCGTAGAGCACCCATATATTATTATTGGCCAAGTCGCATCCATCCTATACTTTGCATTATTCCTCATTCTCGTCCCACTAGCAGGATGGGTAGAAAATAAAGCATTGAAATGAGCCAF533763Chondrostomameandrense          ATGGCAAGCCTACGAAAAACCCACCCACTAATAAAAATCGCTAACGACGCGCTAGTCGACCTCCCAACACCATCTAATATCTCAGTAATGTGAAACTTCGGATCTCTCCTAGGATTATGTTTAATTACTCAAATCCTGACAGGATTATTCTTAGCCATACATTACACCTCTGATATCTCAACCGCATTCTCATCAGTGACCCACATCTGCCGAGATGTCAACTACGGCTGACTTATCCGAAGCTTACATGCCAATGGAGCATCCTTTTTCTTCATCTGTCTTTACATGCATATCGCGCGGGGCCTGTATTATGGATCATACCTTTATAAGGAAACCTGAAATATCGGTGTAGTCCTATTCCTTCTGGTTATGATAACGGCCTTTGTAGGCTACGTTCTTCCGTGGGGACAAATATCCTTTTGAGGTGCTACCGTAATTACAAACCTCCTCTCCGCCGTCCCCTACATAGGGGATACCCTTGTTCAATGAATCTGAGGCGGCTTCTCAGTAGACAATGCGACTCTCACACGGTTCTTCGCATTCCACTTCCTACTACCCTTTGTAGTTGCCGGCGCAACCATCCTACACTTGTTGTTTTTACACGAAACGGGGTCGAACAACCCGGCCGGACTAAATTCAGACGCCGACAAAATTTCTTTCCACCCGTACTTCTCATATAAAGATCTCCTTGGTTTTGTGGCAATGTTACTAGCCCTTACCTCTCTAACATTATTTTCCCCTAACCTATTAGGTGACCCGGAAAACTTTACCCCAGCAAACCCGCTTGTAACACCACCGCATATTCAGCCAGAATGATACTTCTTATTCGCCTACGCCATCCTTCGATCCATCCCAAATAAACTAGGAGGGGTTCTTGCACTACTATTCAGCATCCTAGTGCTATTAGTCGTGCCAATTTTACACACATCCAAACAACGAGGACTAACTTTCCGCCCAGTGACCCAATTCCTATTCTGAACCTTGGTTGCAGATATATTTATTTTGACATGAATCGGGGGTATACCCGTAGAACACCCGTACATTGTTATTGGCCAAGTCGCATCCATCCTATACTTTGCACTATTCCTCGTTCTTGTCCCATTAGCAGGATGAGTGGAAAATAAAGCATTGAAATGAGCCAY026402Chondrostomanasus               atggcaagcctacgaaaaacccacccactaataaaaatcgctaacgacgcgctagtcgacctcccaacaccatctaatatctcagtaatgtgaaacttcggatctctcctaggattatgtttaattacccaaattctgacaggattattcttagccatacattacacctctgatatctcaaccgcattctcatcagtgacccacatctgccgagatgtcaactacggctgacttatccgaagcctgcatgccaatggagcatcctttttcttcatctgtctttacatgcatatcgcacggggcctatattatgggtcatacctttataaagaaacctgaaacatcggtgtagtcctattccttctggttatgataacggcctttgtaggctacgttcttccgtggggacaaatatccttttgaggtgctaccgtaattacaaacctcctctctgccgtcccctacatgggggatacccttgttcaatgaatctgaggcggcttctcagtagacaatgcgactctcacacggttcttcgcattccacttcctactaccctttgtagttgccggcgcaaccatcctgcacttgttgtttttacacgaaacggggtcgaacaacccggccggactaaattcagacgccgacaaaatttctttccacccgtacttctcatataaagatctccttggttttgtggcaatgttactagcccttacctctctgacattattttcccctaacctattaggtgacccggaaaactttaccccagcaaacccgctcgtgacaccaccgcatatccagccagaatgatacttcttatttgcctacgccatccttcggtctatcccaaataaactaggaggggttcttgcactactattcagcatcctagtgctattagtcgtgccaattttacacacatccaaacaacgaggactaactttccgcccagtgacccaattcctattctgaaccctggttgcagatatatttattttgacatgaatcgggggtatacccgtagaacacccatacattgttattggccaagtcgcatccatcctatactttgcactattcctcattcttgtcccactagcaggatgggtggaaaataaagcattgaaatgagccHM560083Chondrostomaoxyrhynchum         ATGGCAAGCCTACGAAAAACCCACCCACTAATAAAAATCGCTAACGACGCGCTAGTCGACCTCCCAACACCGTCTAATATCTCAGTAATGTGAAACTTCGGATCTCTCCTAGGATTATGTTTAATTACCCAAATCCTGACGGGATTATTCTTAGCCATACATTACACCTCTGATATCTCAACCGCCTTCTCATCAGTGACCCACATCTGCCGAGATGTTAACTACGGCTGACTTATCCGAAGCCTACATGCCAACGGAGCATCCTTTTTCTTCATCTGTCTTTACATGCATATCGCACGGGGCCTGTATTATGGGTCATACCTTTATAAAGAGACCTGAAACATCGGTGTAGTCCTATTCCTTCTGGTTATGATGACGGCCTTTGTGGGCTACGTTCTTCCATGAGGACAAATGTCCTTTTGAGGTGCTACCGTAATTACAAACCTCCTCTCTGCCGTCCCCTACATAGGGGATACCCTTGTTCAATGAATCTGGGGCGGCTTCTCAGTAGACAATGCGACTCTCACACGGTTCTTCGCATTCCACTTCCTACTGCCCTTTGTAGTTGCCGGCGCAACCATCCTACACTTGTTGTTTTTACACGAAACGGGGTCGAACAACCCGGCCGGACTAAATTCAGACGCCGACAAGATTTCTTTCCACCCATACTTCTCATATAAAGATCTCCTTGGTTTTGTGGCCATGTTACTAGCCCTTACCTCCCTAACATTATTTTCCCCTAACCTATTAGGTGACCCGGAAAACTTTACCCCAGCAAACCCACTTGTAACACCTCCACATATTCAGCCAGAATGATACTTCTTATTTGCCTACGCCATCCTTCGATCCATCCCAAATAAGCTAGGAGGGGTTCTTGCACTACTATTCAGCATCCTGGTGCTATTAGTCGTGCCAATTTTACACACATCTAAACAACGAGGACTAACTTTCCGCCCAGTGACCCAATTCCTATTCTGAACCCTGGTTGCAGATATATTTATTTTGACATGAATCGGGGGTATACCCGTAGAACACCCATACATTATTATTGGCCAAGTCGCATCCATCCTATACTTTGCACTGTTCCTCATTCTTGTCCCACTAGCAGGATGGGTGGAAAATAAAGCATTGAAATGAGCCHM560084Chondrostomaoxyrhynchum         ATGGCAAGCCTACGAAAAACCCACCCACTAATAAAAATCGCTAACGACGCGCTAGTCGACCTCCCAACACCGTCTAATATCTCAGTAATGTGAAACTTCGGATCTCTCCTAGGATTATGTTTAATTACCCAAATCCTGACGGGATTATTCTTAGCCATACATTACACCTCTGATATCTCAACCGCCTTCTCATCAGTGACCCACATCTGCCGAGATGTTAACTACGGCTGACTTATCCGAAGCCTACATGCCAACGGAGCATCCTTTTTCTTCATCTGTCTTTACATGCATATCGCACGGGGCCTGTATTATGGGTCATACCTTTATAAAGAGACCTGAAACATCGGTGTAGTCCTATTCCTTCTGGTTATGATGACGGCCTTTGTGGGCTACGTTCTTCCATGAGGACAAATGTCCTTTTGAGGTGCTACCGTAATTACAAACCTCCTCTCTGCCGTCCCCTACATAGGGGATACCCTTGTTCAATGAATCTGAGGCGGCTTCTCAGTAGACAATGCGACTCTCACACGGTTCTTCGCATTCCACTTCCTACTGCCCTTTGTAGTTGCCGGCGCAACCATCCTACACTTGTTGTTTTTACACGAAACGGGGTCGAACAACCCGGCCGGACTAAATTCAGACGCCGACAAGATTTCTTTCCACCCATACTTCTCATATAAAGATCTCCTTGGTTTTGTGGCCATGTTACTAGCCCTTACCTCCCTAACATTATTTTCCCCTAACCTATTAGGTGACCCGGAAAACTTTACCCCAGCAAACCCACTTGTAACACCTCCACATATTCAGCCAGAATGATACTTCTTATTTGCCTACGCCATCCTTCGATCCATCCCAAATAAGCTAGGAGGGGTTCTTGCACTACTATTCAGCATCCTGGTGCTATTAGTCGTGCCAATTTTACACACATCTAAACAACGAGGACTAACTTTCCGCCCAGTGACCCAATTCCTATTCTGAACCCTGGTTGCAGATATATTTATTTTGACATGAATCGGGGGTATACCCGTAGAACACCCATACATTATTATTGGCCAAGTCGCATCCATCCTATACTTTGCACTGTTCCTCATTCTTGTCCCACTAGCAGGATGGGTGGAAAATAAAGCATTGAAATGAGCCHM560081Chondrostomaphoxinus            ATGGCAAGCCTACGAAAAACCCACCCACTAATAAAAATCGCTAACGACGCGCTAGTCGACCTCCCAACACCATCTAATATCTCAGTAATGTGAAACTTCGGATCTCTCCTGGGATTGTGTTTGATTACCCAAATCCTAACAGGATTATTCTTAGCCATGCATTACACCTCTGATATCTCAACCGCATTCTCATCAGTAACCCACATCTGCCGTGATGTTAACTACGGCTGACTTATCCGAAGCCTACATGCCAATGGGGCATCCTTTTTCTTCATCTGTCTTTATATGCATATCGCACGAGGCCTGTATTATGGGTCGTACCTCTATAAAGAGACCTGAAACATTGGTGTAGTCCTATTCCTCCTAGTTATAATAACAGCCTTTGTAGGCTACGTTCTTCCATGAGGACAAATATCCTTTTGAGGTGCTACCGTGATTACAAACCTCCTCTCTGCGGTCCCGTACATAGGAGATACCCTTGTTCAATGAATCTGAGGCGGCTTCTCAGTAGACAATGCGACTCTTACACGATTCTTCGCATTCCACTTCCTACTGCCATTCGTCGTTGCCGGCGCAACCATCCTACACTTATTGTTTTTACACGAAACGGGGTCGAACAACCCGGCCGGATTAAATTCCGACGCAGACAAAATTTCTTTCCATCCATACTTCTCCTATAAAGACCTTCTTGGTTTTGTGGCCATGCTACTAGCCCTTACCTCCCTAACATTATTTTCCCCTAACCTACTAGGTGACCCGGAAAACTTTACCCCAGCAAACCCACTAGTAACACCCCCACATATCCAGCCAGAATGATACTTCTTATTTGCCTACGCCATCCTTCGATCTATTCCAAATAAACTAGGAGGGGTTCTTGCACTACTATTCAGCATTCTAGTGCTAATAGTCGTGCCAATTTTACACACATCCAAACAACGAGGACTAACTTTCCGCCCAGTGACCCAATTCCTATTCTGAACCCTGGTTGCAGATATATTTATTTTGACATGAATCGGGGGTATACCCGTAGAACACCCATATATTATTATTGGCCAAATCGCATCCATCCTATATTTTGCATTACTCCTCATTCTTGTCCCACTAGCAGGATGGGTGGAAAATAAAGCATTGAAATGAGCCHM560082Chondrostomaphoxinus            ATGGCAAGCCTACGAAAAACCCACCCACTAATAAAAATCGCTAACGACGCGCTAGTCGACCTCCCAACACCATCTAATATCTCAGTAATGTGAAACTTCGGCTCTCTCCTGGGATTGTGTTTGATTACCCAAATCCTAACAGGATTATTCTTAGCCATGCATTACACCTCTGATATCTCAACCGCATTCTCATCAGTAACCCACATCTGCCGTGATGTTAACTACGGCTGACTTATCCGAAGCCTACATGCCAATGGGGCATCCTTTTTCTTCATCTGTCTTTATATGCATATCGCACGAGGCCTGTATTATGGGTCGTACCTCTATAAAGAGACCTGAAACATTGGTGTAGTCCTATTCCTCCTAGTTATAATAACAGCCTTTGTAGGCTACGTTCTTCCATGAGGACAAATATCCTTTTGAGGTGCTACCGTGATTACAAACCTCCTCTCTGCGGTCCCGTACATAGGAGATACCCTTGTTCAATGAATCTGAGGCGGCTTCTCAGTAGACAATGCGACTCTTACACGATTCTTCGCATTCCACTTCCTACTGCCGTTCGTCGTTGCCGGCGCAACCATCCTACACTTATTGTTTTTACACGAAACGGGGTCAAACAACCCGGCCGGATTAAATTCCGACGCAGACAAAATTTCTTTCCATCCATACTTCTCCTATAAAGACCTTCTCGGTTTTGTGGCCATGCTACTAGCCCTTACCTCCCTAACATTATTTTCCCCTAACCTACTAGGTGACCCGGAAAACTTTACCCCAGCAAACCCACTAGTAACACCCCCACATATCCAGCCAGAATGATACTTCTTATTTGCCCGCGCCATCCTTCGATCTATTCCAAATAAACTAGGAGGGGTTCTTGCACTACTATTCAGCATTCTAGTGCTAATAGTCGTGCCAATTTTACACACATCCAAACAACGAGGACTAACTTTCCGCCCAGTGACCCAATTCCTATTCTGAACCCTGGTTGCAGATATATTTATTTTGACATGAATCGGGGGTATACCCGTAGAACACCCATATATTATTATTGGCCAAATCGCATCCATCCTATATTTTGCATTATTCCTCATTCTTGTCCCACTAGCAGGATGGGTGGAAAATAAAGCATTGAAATGAGCCAF090747Chondrostomaprespensis          atggcaagcctacgaaaaacccacccactaataaaaatcgctaacgacgcgctagtcgacctcccaacaccatctaatatctcagtaatgtgaaacttcggatctctcctaggattatgtttgattacccaaatcctgacaggattattcttagccatacattacacctctgatatctcgaccgcattctcatcagtgacccacatctgccgagatgtcaactacggctgacttatccgaagcctgcatgccaacggagcatcctttttcttcatctgtctttacatacatatcgcacggggcctatattatgggtcatacctttataaagagacctgaagcatcggtgtagtcctattccttctggttatgataacggcctttgtgggctacgttcttccatggggacaaatatccttttgaggtgctaccgtaattacaaacctcctctctgccgtcccctacatgggggatacccttgttcaatgaatctgaggcggcttctcagtagataatgcgactctcacacggttcttcgcgttccacttcctactacccttcgtagttgccggcgcaaccatcctacacttgttgttcttacacgaaacggggtcgaacaacccggccggactaaattcagacgccgacaaaatttctttccacccgtacttctcatataaagatctccttggttttgtggcaatattactagcccttacctccctaacattattttcccctaacctattaggtgacccggaaaactttaccccagcaaacccgctcgtaacaccgccacatatccaaccagaatgatacttcttatttgcctacgccatccttcgatccatcccaaataagctaggaggggttcttgcactactattcagtatcctagtgctattagtcgtgccaattttacacacatccaaacaacgaggactaactttccgccccgtaacccaattcctattctgaaccctggttgcagatatatttattttgacatgaatcgggggtatacccgtagaacacccatacattgttattggccaagtcgcatccatcctatactttgcactattcctcattcttgtcccactagcaggatgagtggaaaataaagcattgaaatgagccAF533757Chondrostomaregium              ATGGCAAGCCTACGAAAAACCCACCCACTAATAAAAATCGCTAACGACGCACTAGTCGACCTCCCAACACCATCTAATATCTCAGTAATGTGAAACTTCGGATCTCTCCTGGGATTGTGTTTGATTACCCAAATCCTAACAGGATTATTCTTAGCCATACACTACACCTCTGATATCTCAACCGCATTCTCATCAGTGACCCACATCTGCCGAGATGTCAACTACGGCTGACTTATCCGAAGCCTGCATGCCAACGGAGCATCCTTTTTCTTCATCTGTCTTTACATGCATATCGCACGGGGCCTATATTATGGATCATACCTTTATAAAGAGACCTGAAACATCGGTGTGGTCCTATTCCTTCTGGTTATAATAACAGCCTTTGTTGGCTACGTTCTTCCATGAGGACAAATGTCCTTTTGAGGTGCTACCGTGATTACAAACCTCCTCTCTGCCGTCCCCTACATGGGGGATACCCTTGTTCAATGAATCTGAGGCGGCTTCTCAGTAGACAATGCAACTCTCACACGGTTCTTCGCATTCCACTTCCTACTGCCCTTTGTAGTTGCCGGCGCAACTATCCTACACTTGTTGTTTTTACACGAAACGGGGTCGAACAACCCAGCCGGACTAAATTCAGACGCCGACAAAATTTCTTTCCACCCATATTTCTCATATAAAGATCTCCTTGGTTTTGTGGCCATATTACTAGCCCTTACCTCCCTAACATTATTTTCCCCTAACCTATTGGGTGACCCGGAAAACTTTACCCCAGCGAACCCACTCGTAACACCACCACATATCCAGCCAGAATGATACTTCTTATTTGCCTACGCCATCCTTCGATCCATCCCAAATAAACTAGGAGGGGTCCTTGCACTACTATTCAGCATTCTAGTGCTATTAGTCGTGCCAATTTTACACACGTCTAAACAGCGAGGACTAACTTTCCGCCCAGTGACCCAATTCCTATTCTGAACCCTAGTTGCAGATATATTTATTTTGACATGAATCGGGGGTATACCCGTAGAACACCCATACATTGTTATTGGCCAAGTCGCATCCATCCTATATTTTGCACTATTCCTCATTCTTGTCCCACTAGCAGGATGAGTGGAAAATAAAGCATTGAAATGAGCCAY026400Chondrostomaregium              ATGGCAAGCCTACGAAAAACCCACCCACTAATAAAAATCGCTAACGACGCGCTAGTCGACCTCCCAACGCCATCTAATATCTCAGTAATATGAAACTTCGGATCTCTCCTGGGATTGTGTTTGATTACCCAAATCCTGACAGGATTATTCTTAGCCATACACTACACCTCTGATATCTCAACCGCATTCTCATCAGTGACCCACATCTGCCGAGATGTCAACTACGGCTGACTTATCCGAAGTCTACATGCCAACGGAGCATCCTTTTTCTTCATCTGTCTTTACATGCATATCGCACGAGGCCTATATTATGGGTCATACCTTTATAAAGAGACCTGAAACATCGGTGTAGTCCTATTCCTTCTGGTCATAATAACAGCCTTTGTTGGCTACGTTCTTCCATGAGGACAAATATCCTTTTGAGGTGCTACCGTGATTACAAACCTCCTCTCTGCCGTCCCCTACATAGGGGATACCCTTGTTCAATGAATCTGAGGCGGCTTCTCAGTAGACAATGCAACTCTCACACGGTTCTTCGCATTCCACTTCCTACTACCCTTTGTAGTTGCCGGCGCAACTATCCTACACTTGTTGTTTTTACACGAAACGGGGTCGAACAACCCAGCCGGACTAAATTCAGACGCCGACAAAATTTCTTTCCACCCATACTTCTCATATAAAGATCTCCTTGGTTTTGTGGCCATATTACTAGCCCTTACCTCCCTAACATTATTTTCCCCTAACCTATTGGGTGACCCGGAGAACTTCACCCCAGCAAACCCACTCGTAACACCACCACATATCCAGCCAGAGTGATACTTCTTATTTGCCTACGCCATCCTTCGATCCATCCCAAATAAACTAGGAGGGGTCCTTGCACTACTATTCAGCATTCTAGTGCTATTAGTCGTGCCAATTTTACACACGTCTAAACAACGAGGACTAACTTTCCGCCCAGTGACCCAATTCCTATTCTGAACCCTAGTTGCGGATATATTTATTTTGACATGAATCGGGGGTATACCCGTAGAACACCCATACATTGTTATTGGCCAAGTCGCATCCATCCTATATTTTGCGCTATTCCTCATTCTTGTCCCACTAGCGGGATGAGTGGAAAATAAAGCATTGAAATGAGCCAY568623Chondrostomasoetta              atggcaagcctacgaaaaacccacccactaataaaaatcgctaacgacgcgctagtcgacctcccaacaccatctaatatctcagtaatgtgaaacttcgggtctctcctgggattgtgtttgattacccaaatcctaacaggattattcctagccatgcattacacctctgatatttcaaccgcattctcatcagtaacccacatctgccgtgatgttaactacggctgacttatccgaagcctacatgccaatggagcatcctttttcttcatctgtctttatatgcatatcgcacggggcctgtattatggatcatacctctataaagagacctgaaacattggtgtagtcctattcctcctggttataataacagcctttgtaggctacgttcttccatgaggacaaatatccttttgaggtgctaccgtgattacaaacctcctctctgcagtcccgtacataggggatacccttgtgcaatgaatctgaggcggcttctcagtaaacaatgcgactcttacacgattcttcgcattccacttcctactgccattcgtcgttgccggcgcaaccatcctgcacttattatttttacacgaaacgggatctaacaacccggccggattaaattccgacgcagataaaatttctttccacccatatttttcatataaagaccttcttggttttgtggccatgttactagcccttacctccctaacattattttctcctaacctactaggtgacccggaaaactttaccccagcaaacccactagtaacgcctccacatatccagccagagtgatacttcttatttgcctacgccatccttcgatctatcccaaataaactaggaggggttcttgcactactatttagcattctagtgctattagtcgtgccaattttacacacatccaaacaacgaggactaactttccgcccggtgacccaattcttattctgaaccctggttgcagatatatttattctgacatgaatcgggggcatacccgtagaacacccatatattattattggccaagtcgcatccatcctatactttgcattattcctcattcttgtcccactagcaggatgggtagaaaataaagcattgaaatgagccAY568624Chondrostomasoetta              atggcaagcctacgaaaaacccacccactaataaaaatcgctaacgacgcgctagtcgacctcccaacaccatctaatatctcagtaatgtgaaacttcgggtctctcctgggattgtgtttgattacccaaatcctaacaggattattcctagccatgcattacacctctgatatctcaaccgcattctcatcggtaacccacatctgccgtgatgttaactacggctgacttatccgaagcctacatgccaatggagcatcctttttcttcatctgtctttatatgcatatcgcacggggcctgtattatggatcatacctctataaagagacctgaaacattggtgtagtcctattcctcctggttataataacagcctttgtaggctacgttcttccatgaggacaaatatccttttgaggtgctaccgtgattacaaacctcctctctgcagtcccgtacataggggatacccttgtgcaatgaatctgaggcggcttctcagtagacaatgcgactcttacacgattcttcgcattccacttcctgctgccattcgtcgttgccggcgcaaccatcctgcacttattatttttacacgaaacgggatctaacaacccggccggattaaattccgacgcagacaaaatttctttccacccatatttttcatataaagaccttcttggttttgtggccatgttactagcccttacctccctaacattattttctcctaacctactaggtgacccggaaaactttaccccagcaaacccactagtaacgcccccacatatccagccagagtgatacttcttatttgcctacgccatccttcgatctatcccaaataaactaggaggggttcttgcactactatttagcattctagtgctattagtcgtgccaattttacacacatccaaacaacgaggactaactttccgcccggtgacccaattcttattctgaaccctggttgcagatatatttattctgacatgaatcgggggcatacccgtagagcacccatatattattattggccaagtcgcatccatcctatactttgcattattcctcattcttgtcccactagcaggatgggtagaaaataaagcattgaaatgagccAF090748Chondrostomavardarense          atggcaagcctacgaaaaacccacccactaataaaaatcgctaacgacgcgctagtcgacctcccaacaccatctaatatctcagtaatgtgaaacttcggatctctcctaggattatgtttgattacccaaatcctgacaggattattcttagccatacattacacctctgatatctcaaccgcattctcatcagtgacccacatctgccgagatgtcaactacggctgacttatccgaagcctgcatgccaatggagcatcctttttcttcatctgtctttacatacatatcgcacggggcctatattatgggtcatacctttataaagagacctgaaacatcggtgtagtcctattccttctggttatgataacggcctttgtgggctacgttcttccatggggacaaatatccttttgaggtgctaccgtaattacaaacctcctctctgccgtcccctacataggggatacccttgttcaatgaatctggggcggcttctcagtagacaatgcgactctcacacggttcttcgcgttccacttcctactacccttcgtagttgccggcgcaaccatcctacacttgttgtttttacacgaaacgggatcgaacaacccggccggattaaattcagacgccgacaaaatttctttccacccgtacttctcatataaagatctccttggttttgtggcaatattactagcccttacctccctaacattattttcccctaacctattaggtgacccggaaaactttaccccagcaaacccgctcgtaacacccccacatatccagccagaatgatacttcttatttgcctacgccatccttcgatccatcccaaacaagctaggaggggttcttgcactactattcagcatcctagtgctattagtcgtgccaattttacacacatccaaacaacgaggactaactttccgcccagtaacccaattcctattctgaaccctggttgcagatatatttattttgacatgaatcgggggtatacccgtagaacacccatacattgttattggccaagtcgcatccatcctatactttgcactattcctcattcttgtcccactagcaggatgagtggaaaataaagcattgaaatgagccHM560085Chondrostomavardarense          ATGGCAAGCCTACGAAAAACCCACCCACTAATAAAAATCGCTAACGACGCGCTAGTCGACCTCCCAACACCATCTAATATCTCAGTAATATGAAACTTCGGATCTCTCCTAGGATTATGTTTGATTACCCAAATCCTGACAGGATTATTCTTAGCCATACATTACACCTCTGATATCTCAACCGCATTCTCATCAGTGACCCACATCTGCCGAGATGTCAACTACGGCTGACTTATCCGAAGCCTGCATGCCAATGGAGCATCCTTTTTCTTCATCTGTCTTTACATACATATCGCACGGGGCCTATATTATGGGTCATACCTTTATAAAGAGACCTGAAACATCGGTGTAGTCCTATTCCTTCTGGTTATGATAACGGCCTTTGTGGGCTACGTTCTTCCATGGGGACAAATATCCTTTTGAGGTGCTACCGTAATTACAAACCTCCTCTCTGCCGTCCCCTACATAGGGGATACCCTTGTTCAATGAATCTGGGGCGGCTTCTCAGTAGACAATGCGACTCTCACACGGTTCTTCGCGTTCCACTTCCTACTACCCTTCGTAGTTGCCGGCGCAACCATCCTACACTTGTTGTTTTTACACGAAACGGGATCGAACAACCCGGCCGGACTAAATTCAGACGCCGACAAAATTTCTTTCCACCCATACTTCTCATATAAAGATCTCCTTGGTTTTGTGGCAATATTACTAGCCCTTACCTCTCTAACATTATTTTCCCCTAACCTATTAGGTGACCCGGAAAACTTTACCCCAGCAAACCCACTCGTAACACCCCCACATATCCAGCCAGAATGATACTTCTTATTTGCCTACGCCATCCTTCGATCCATCCCGAACAAGCTAGGAGGGGTTCTTGCACTACTATTCAGCATCCTAGTGCTATTAGTCGTGCCAATTTTACACACATCCAAACAACGAGGACTAACTTTCCGCCCAGTAACCCAATTCCTATTCTGAACCCTGGTTGCAGATATATTTATTTTGACATGAATCGGGGGTATACCCGTAGAACACCCATACATTGTTATTGGCCAAGTCGCATCCATCCTATACTTTGCACTATTCCTCATTCTTGTCCCACTAGCAGGATAAGTGGAAAATAAAGCATTGAAATGAGCCHM560088Delminichthysadpersus           ATGGCAAGCCTACGAAAGACCCACCCCCTAATAAAAATCGCTAATCACGCCCTGGTTGACCTCCCAACGCCGTCCAATATTTCAGCACTATGAAACTTCGGGTCCCTACTAGGATTATGTTTAATTACCCAAATCCTAACAGGACTATTTCTAGCTATACATTACACCTCTGACATCTCGACTGCATTTTCATCAGTAGTACACATCTGCCGAGACGTTAATTACGGCTGACTTATCCGGAACCTGCATGCTAATGGAGCGTCCTTCTTTTTCATCTGTATCTACATACACATCGCACGCGGTCTATATTACGGATCATACCTTTATAAAGAGACCTGAAACATTGGGGTAGTGCTACTTCTCCTAGTTATAATGACAGCCTTCGTCGGTTATGTACTTCCCTGAGGACAAATATCTTTTTGAGGCGCCACCGTAATTACAAACCTTCTCTCAGCGGTCCCCTACATAGGAGACACCCTTGTCCAATGGATCTGAGGCGGCTTCTCAGTAGATAACGCAACCCTCACACGATTCTTCGCATTCCACTTCCTCTTTCCATTTGTTATTGCCGGCGCAACCCTCCTGCATTTACTATTTCTACACGAAACAGGCTCTAACAACCCAGCCGGGTTAAACTCCGACGCGGACAAAATTTCTTTTCATCCGTACTTCTCATATAAAGACCTTCTTGGATTTGTAATTATATTGCTAGCTCTCACCTCCCTGGCATTATTTTCTCCCAATCTACTAGGGGACCCAGAAAACTTTACCCCAGCGAACCCCCTTGTAACGCCACCACATATTCAGCCTGAGTGATATTTCCTGTTTGCCTACGCCATCCTCCGATCTATCCCAAATAAACTAGGAGGAGTTCTTGCACTATTATTCAGTATTCTAGTGCTAATAGTCGTACCAATTCTCCACACCTCTAAACAACGGGGACTAACTTTCCGCCCTCTAACCCAATTCCTATTCTGAGCCCTCGTTGCAGACATGATTATTCTAACATGAATTGGAGGCATACCTGTTGAACACCCATATATTATTATTGGCCAAATCGCATCCATTTTATACTTTGCACTCTTCCTCGTTCTTGTACCACTAGCAGGATGAGTAGAAAACAAAGCACTGAAATGAGCCHM560089Delminichthysadpersus           ATGGCAAGCCTACGAAAAACCCACCCCCTAATAAAAATCGCTAATCACGCCCTGGTTGACCTCCCAACGCCGTCCAATATTTCAGCACTATGAAACTTCGGGTCCCTACTAGGATTATGTTTAATTACCCAAATCCTAACAGGACTATTTCTAGCTATACATTACACCTCTGACATCTCGACTGCATTTTCATCAGTAGTACACATCTGCCGAGACGTTAATTACGGCTGACTTATCCGGAACCTGCATGCTAATGGGGCGTCCTTCTTTTTCATCTGTATCTACATACACATCGCACGCGGTCTATATTACGGATCATACCTTTATAAAGAGACCTGAAACATTGGGGTAGTGCTACTTCTCCTAGTTATAATGACAGCCTTCGTCGGTTATGTACTTCCCTGAGGACAAATATCTTTTTGAGGCGCCACCGTAATTACAAACCTTCTCTCAGCGGTCCCCTACATAGGAGACACCCTTGTCCAATGGATCTGAGGCGGCTTCTCAGTAGATAACGCAACCCTCACACGATTCTTCGCATTCCACTTCCTCTTTCCATTTGTTATTGCCGGCGCAACCCTCCTGCATTTACTATTTCTACACGAAACAGGCTCTAACAACCCAGCCGGGTTAAACTCCGACGCGGACAAAATTTCTTTTCATCCGTACTTCTCATATAAAGACCTTCTTGGATTTGTAATTATATTGCTAGCTCTCACCTCCCTGGCATTATTTTCTCCCAATCTACTAGGGGACCCAGAAAACTTTACCCCAGCGAACCCCCTTGTAACGCCACCACATATTCAGCCTGAGTGATATTTCCTGTTTGCCTACGCCATCCTCCGATCTATCCCAAATAAACTAGGAGGAGTTCTTGCACTATTATTCAGTATTCTAGTGCTAATAGTCGTACCAATTCTCCACACCTCTAAACAACGGGGACTAACTTTCCGCCCTCTAACCCAATTCCTATTCTGAGCCCTCGTTGCAGACATGATTATTCTAACATGAATTGGAGGCATACCTGTTGAACACCCATATATTATTATTGGCCAAATCGCATCCATTTTATACTTTGCACTCTTCCTCGTTCTTGTACCACTAGCAGGATGAGTAGAAAACAAAGCACTGAAATGAGCCHM560090Delminichthysghetaldii          ATGGCAAGCCTACGAAAAACCCACCCCCTAATAAAAATCGCTAATCACGCCCTAGTTGACCTCCCAACGCCATCCAATATTTCAGCACTATGAAACTTCGGGTCCCTCCTAGGATTATGTTTAATTACCCAAATCCTAACAGGACTATTTCTAGCCATACATTACACCTCTGACATCTCAACTGCGTTTTCATCAGTAGTACACATCTGCCGAGACGTTAATTACGGCTGACTTATCCGGAACCTGCATGCTAATGGAGCGTCCTTCTTTTTCATCTGTATCTACATACACATCGCACGCGGTCTATATTACGGATCATACCTTTATAAAGAAACCTGAAACATTGGGGTAGTCCTACTTCTCCTAGTTATAATGACGGCCTTCGTCGGTTATGTACTTCCCTGAGGACAAATATCTTTTTGAGGCGCCACCGTAATTACAAACCTTCTCTCAGCGGTCCCCTACATAGGAGACACCCTTGTCCAATGGATCTGGGGCGGCTTCTCAGTAGATAACGCAACCCTCACACGATTCTTCGCATTCCACTTCCTCTTTCCATTTGTTATTGCCGGCGCAACCCTCCTGCATTTACTATTTCTACACGAAACAGGCTCTAACAACCCAGCCGGGCTAAACTCCGACGCGGACAAAATTTCTTTTCATCCGTACTTCTCATACAAAGACCTTCTTGGATTTGTAATTATATTGCTAGCTCTCACCTCCCTGGCATTATTTTCTCCCAATCTGCTAGGAGACCCAGAAAACTTTACCCCAGCAAACCCACTTGTAACACCACCACATATTCAGCCTGAGTGATATTTCCTGTTTGCCTACGCCATCCTCCGATCTATCCCAAATAAACTAGGAGGAGTTCTTGCACTATTATTCAGTATTCTAGTGCTAATAGTCGTGCCAATTCTCCACACCTCTAAACAACGAGGACTAACTTTCCGCCCTCTAACCCAATTCCTATTCTGAGCCCTCGTTGCAGACATGATTATTCTAACATGAATTGGAGGCATACCTGTTGAACACCCATATATTATTATTGGCCAAATCGCATCCATTTTATACTTTGCACTCTTCCTTGTTCTTGTACCACTAGCAGGATGAGTAGAAAACAAAGCACTGAAATGAGCCHM560091Delminichthysghetaldii          ATGGCAAGCCTACGAAAAACCCACCCCCTAATAAAAATCGCTAATCACGCCCTAGTTGACCTCCCAACGCCATCCAATATTTCAGCACTATGAAACTTCGGGTCCCTCCTAGGATTATGTTTAATTACCCAAATCCTAACAGGACTATTTCTAGCCATACATTACACCTCTGACATCTCAACTGCGTTTTCATCAGTAGTACACATCTGCCGAGACGTTAATTACGGCTGACTTATCCGGAACCTGCATGCTAATGGAGCGTCCTTCTTTTTCATCTGTATCTACATACACATCGCACGCGGTCTATATTACGGATCATACCTTTATAAAGAAACCTGAAACATTGGGGTAGTCCTACTTCTCCTAGTTATAATGACGGCCTTCGTCGGTTATGTACTTCCCTGAGGACAAATATCTTTTTGAGGCGCCACCGTAATTACAAACCTTCTCTCAGCGGTCCCCTACATAGGAGACACCCTTGTCCAATGGATCTGGGGCGGCTTCTCAGTAGATAACGCAACCCTCACACGATTCTTCGCATTCCACTTCCTCTTTCCATTTGTTATTGCCGGCGCAACCCTCCTGCATTTACTATTTCTACACGAAACAGGCTCTAACAACCCAGCCGGGCTAAACTCCGACGCGGACAAAATTTCTTTTCATCCGTACTTCTCATACAAAGACCTTCTTGGATTTGTAATTATATTGCTAGCTCTCACCTCCCTGGCATTATTTTCTCCCAATCTGCTAGGAGACCCAGAAAACTTTACCCCAGCGAACCCACTTGTAACACCACCACATATTCAGCCTGAGTGATATTTCCTGTTTGCCTACGCCATCCTCCGATCTATCCCAAATAAACTAGGAGGAGTTCTTGCACTACTATTCAGTATTCTAGTGCTAATAGTCGTGCCAATTCTCCACACCTCTAAACAACGAGGACTAACTTTCCGCCCTCTAACCCAATTCCTATTCTGAGCCCTCGTTGCAGACATGATTATTCTAACATGAATTGGAGGCATACCTGTTGAACACCCATATATTATTATTGGCCAAATCGCATCCATTTTATACTTTGCACTCTTCCTTGTTCTTGTACCACTAGCAGGATGAGTAGAAAACAAAGCACTGAAATGAGCCAY838924Delminichthysjadovensis         atggcaagcctacgaaagacccaccccttaataaaaatcgctaatcacgccctagttgacctcccaacgccgtccaatatttcagcactatgaaacttcgggtccctcctaggattatgtttaattacccaaatcctaacaggactatttctagctatacattacacctctgacatctccactgcattttcatcagtggtacacatctgccgagacgttaattacggctgacttatccggaacctgcatgctaatggggcatccttctgtttcatctgtatctacatgcacatcgcacgcggtctatattacggatcatacctttataaagagacctgaaacattggagtggttctacttctcctagttataataacagccttcgttggttatgtacttccctgaggacaaatatctttttggggcgccaccgtaattacaaaccttctctcggcggtcccctacataggagacacccttgtccaatggatctgggggggcttctcagtagataacgcaaccctcacacgattcttcgcattccacttcctctttccatttgttattgccggcgcaaccctcctacatttactatttctacacgaaacaggctctaacaacccagccggactaaactccgacgcggacaaaatttcttttcatccgtacttctcatataaagacctccttggattcgtaattatattgctagctctcacctccctagcattattttcccccaatttgctaggagacccagaaaactttaccccagcaaacccccttgtaacaccaccacatatccagcctgagtgatacttcctgtttgcctacgctatcctccgatctatcccaaataaactaggaggagttcttgcactattattcagNattctagtgctaatagtcgtaccaattctccacacctctaaacaacgaggactaactttccgccctctaacccagttcctattctgagcccttgttgcagacatgattattctaacatgaatcggaggcatacctgttgaacatccgtatattattattggccaagtcgcatccatcctatactttgcactcttccttgttctcgtaccactagcaggatgagtagaaaacaaagcactgaaatgagccHM560086Delminichthyskrbavensis         ATGGCAAGCCTACGAAAAACCCACCCCTTAATAAAAATCGCTAATCACGCCCTAGTTGACCTCCCAACGCCATCCAATATTTCAGCACTATGAAACTTCGGGTCCCTCCTAGGATTATGTCTAATTACCCAAATCCTAACAGGACTATTTCTAGCTATACATTACACCTCTGACATCTCCACTGCATTTTCATCAGTGGTACACATCTGCCGAGACGTTAATTACGGCTGACTTATCCGGAACCTGCATGCTAATGGAGCATCCTTCTTTTTCATCTGTATCTACATGCACATCGCACGCGGTCTATATTACGGATCATACCTTTATAAAGAGACCTGAAACATTGGAGTAGTTCTACTTCTCCTAGTTATAATAACAGCCTTCGTCGGCTATGTACTTCCCTGAGGACAAATATCTTTTTGAGGCGCCACCGTAATTACAAACCTTCTCTCGGCGGTCCCCTACATAGGAGACACCCTTGTCCAATGGATCTGGGGCGGCTTCTCAGTAGATAACGCAACCCTCACACGATTCTTCGCATTCCACTTCCTCTTTCCATTTGTTATTGCCGGCGCAACCCTCCTACATTTACTATTTCTACACGAAACAGGCTCTAACAACCCAGCCGGACTAAACTCCGACGCGGACAAAATTTCTTTTCATCCGTACTTCTCATATAAAGACCTCCTTGGATTCGTAATTATATTACTAGCTCTCACCTCCCTGGCATTATTTTCTCCCAATCTGCTAGGAGACCCAGAAAACTTTACCCCAGCGAACCCCCTTGTAACACCACCACATATCCAGCCTGAATGATACTTCCTGTTTGCCTACGCTATCCTCCGATCCATCCCAAATAAACTAGGAGGAGTTCTTGCACTATTATTCAGTATTCTAGTGCTAGTAGTCGTGCCAATTCTCCACACCTCTAAACAACGGGGACTAACTTTCCGCCCTCTAACCCAATTCCTATTCTGAGCCCTTGTTGCAGACATGATTATTTTAACATGAATCGGGGGCATACCTGTTGAACATCCATATATTATTATTGGCCAAGTCGCATCCATCCTATACTTTGCACTCTTCCTTGTTCTTGTACCACTAGCAGGATGAGTAGAAAACAAAGCACTGAAATGAGCCHM560086Delminichthyskrbavensis         ATGGCAAGCCTACGAAAAACCCACCCCTTAATAAAAATCGCTAATCACGCCCTAGTTGACCTCCCAACGCCATCCAATATTTCAGCACTATGAAACTTCGGGTCCCTCCTAGGATTATGTCTAATTACCCAAATCCTAACAGGACTATTTCTAGCTATACATTACACCTCTGACATCTCCACTGCATTTTCATCAGTGGTACACATCTGCCGAGACGTTAATTACGGCTGACTTATCCGGAACCTGCATGCTAATGGAGCATCCTTCTTTTTCATCTGTATCTACATGCACATCGCACGCGGTCTATATTACGGATCATACCTTTATAAAGAGACCTGAAACATTGGAGTAGTTCTACTTCTCCTAGTTATAATAACAGCCTTCGTCGGCTATGTACTTCCCTGAGGACAAATATCTTTTTGAGGCGCCACCGTAATTACAAACCTTCTCTCGGCGGTCCCCTACATAGGAGACACCCTTGTCCAATGGATCTGGGGCGGCTTCTCAGTAGATAACGCAACCCTCACACGATTCTTCGCATTCCACTTCCTCTTTCCATTTGTTATTGCCGGCGCAACCCTCCTACATTTACTATTTCTACACGAAACAGGCTCTAACAACCCAGCCGGACTAAACTCCGACGCGGACAAAATTTCTTTTCATCCGTACTTCTCATATAAAGACCTCCTTGGATTCGTAATTATATTACTAGCTCTCACCTCCCTGGCATTATTTTCTCCCAATCTGCTAGGAGACCCAGAAAACTTTACCCCAGCGAACCCCCTTGTAACACCACCACATATCCAGCCTGAATGATACTTCCTGTTTGCCTACGCTATCCTCCGATCCATCCCAAATAAACTAGGAGGAGTTCTTGCACTATTATTCAGTATTCTAGTGCTAGTAGTCGTGCCAATTCTCCACACCTCTAAACAACGGGGACTAACTTTCCGCCCTCTAACCCAATTCCTATTCTGAGCCCTTGTTGCAGACATGATTATTTTAACATGAATCGGGGGCATACCTGTTGAACATCCATATATTATTATTGGCCAAGTCGCATCCATCCTATACTTTGCACTCTTCCTTGTTCTTGTACCACTAGCAGGATGAGTAGAAAACAAAGCACTGAAATGAGCCHM560092Gobiogobio                      ATGGCAAGCCTACGAAAAACCCACCCCCTAATAAAAATCGCTAATGACGCGCTAGTTGACCTACCAACACCATCTAATATTTCAGTCTGATGAAACTTTGGGTCCCTCCTCGGACTCTGCCTAATTACGCAAATCTTAACGGGATTGTTCCTGGCTATACACTACACCTCAGACATTTCAACTGCATTCTCATCAGTTGCCCACATCTGCCGGGACGTAAACTACGGCTGGTTTATTCGTAACGTGCACGCCAACGGAGCATCATTCTTTTTCATCTGCATTTATATGCACGTAGCCCGAGGCCTCTATTACGGATCCTACCTCTACAAAGAAACCTGAAACATTGGAGTAGTTCTGCTGCTGCTAGTTATAATAACGGCCTTCGTTGGCTATGTTCTCCCATGAGGTCAAATATCCTTCTGAGGTGCCACAGTGATCACCAACCTTTTATCAGCAGTCCCTTATATGGGGGACACCCTTGTACAATGAATTTGAGGCGGGTTCTCAGTTGATAACGCAACACTAACACGGTTCTTCGCTTTTCACTTTCTCCTCCCATTTGTTATCGCCGGCGCAACCGTCCTCCACCTACTATTTTTACACGAGACAGGATCAAATAACCCAGCCGGACTAAATTCTGACGCAGACAAAATCTCCTTCCACCCATACTTCTCTTACAAGGACCTTCTTGGTTTCGTCCTAATGCTTCTAGCTCTTACATCCTTGGCACTGTTCTCCCCCAACTTGCTGGGAGACCCAGACAACTTCACCCCCGCAAACCCAATAGTTACCCCTCCCCACATTAAGCCCGAGTGATACTTCCTATTTGCCTACGCCATTCTGCGATCTATCCCTAACAAACTTGGAGGCGTCCTTGCACTATTATTTTCTATCCTCATCCTCATAGTAGTCCCAATCTTACATACCTCAAAACAACGAGGACTAACTTTCCGACCCCTAACACAATTCTTATTCTGAACCCTCGTAGCAGACATACTTATCCTTACATGAATTGGGGGCATACCCGTAGAACATCCATATGTTGTCATCGGCCAAGTCGCCTCAATTCTGTATTTTACACTTTTCCTTGTCCTTGCCCCAATAGCAGGCTGACTGGAAAATAAAGCATTAAAATGAGCTAJ698708Iberochondrostomaalmacai        atggcaagcctacgaaaaacccacccactaataaagatcgctaacgacgcgctagtcgacctcccaacaccatctaatatctcggtaatatgaaacttcggatcgcttctgggattatgtttaattacccaaatcctgacaggattattcttagccatgcattacacctctgatatctcaaccgcattctcatcagtaacccacatctgccgggacgttaactacggctgacttatccgaagcctgcatgccaatggagcatcatttttcttcatctgtctttacatgcacatcgcacgaggcctgtactatggatcatatctttataaagagacctgaaacattggtgtagtcctgttccttctagttatgataacagcctttgttggatacgttcttccatgaggacaaatatccttttgaggtgctaccgtgattacaaatcttctatctgcagtcccctacatgggagataccctagttcagtgaatctgaggggggttctcagtagacaacgcaacgctcacacgattcttcgcattccacttcctattaccatttgtcgtcaccggcgcaaccatcctgcacttgttatttctacacgaaacaggctcgaacaacccggccgggctaaattccgacgcagacaaaatttctttccacccgtatttctcatacaaagatcttcttggctttgtggccatgttactagcccttacttccctaacattattttcccccaacctgttaggtgacccggagaactttaccccagcaaacccactcgtaacacccccacatattcagccagagtgatacttcttatttgcctacgccatcctccgatccatcccaaataaactaggaggggttcttgcactactattcagcattctagtgctaatagtcgtgccagtcttacacacgtccaaacaacgaggactaactttccgcccggtaacccaattcctattctgaaccctggttgcagatatattcattttgacatgaatcgggggcatacccgtagaacacccatatattgttattggccaaatcgcatccattctatactttgcactattcctcattcttgtcccactagcaggatgggtggaaaataaagcattgaaatgagccAJ698707Iberochondrosotmaalmacai        atggcaagcctacgaaaaacccacccactaataaagatcgctaacgacgcgctagtcgacctcccaacaccatctaatatctcggtaatatgaaacttcggatcgcttctgggattatgtttaattacccaaatcctgacaggattattcttagccatgcattacacctctgatatctcaaccgcattctcatcagtaacccacatctgccgggacgttaactacggctgacttatccgaagcctgcatgccaatggagcatcatttttcttcatctgtctttacatgcacatcgcacgaggcctgtactatggatcatatctttataaagagacctgaaacattggtgtagtcctgttccttctagttatgataacagcctttgttggatacgttcttccatgaggacaaatatccttttgaggtgctaccgtgattacaaatcttctatctgcagtcccctacatgggagataccctagttcagtgaatctgaggggggttctcagtagacaacgcaacgctcacacgattcttcgcattccacttcctattaccatttgtcgtcaccggcgcaaccatcctgcacttgttatttctacacgaaacaggctcgaacaacccggccgggctaaattccgacgcagacaaaatttctttccacccgtatttctcatacaaagatcttcttggctttgtggccatgttactagcccttacttccctaacattattttcccccaacctgttaggtgacccggagaactttaccccagcaaacccactcgtaacacccccacatattcagccagagtgatacttcttatttgcctacgccatcctccgatccatcccaaataaactaggaggggttcttgcactactattcagcattctagtgctaatagtcgtgccagtcttacacacgtccaaacaacgaggactaactttccgcccggtaacccaattcctattctgaaccctggttgcagatatattcattttgacatgaatcgggggcatacccgtagaacacccatatattgttattggccaaatcgcatccattctatactttgcactattcctcattcttgtcccactagcaggatgggtggaaaataaagcattgaaatgagccHM560093Iberochondrostomalemmingii      ATGGCAAGCCTACGAAAAACCCACCCGCTAATGAAAATCGCTAACGACGCGCTAGTCGACCTACCAACACCATCTAATATCTCAGTAATATGAAACTTCGGATCTCTTCTAGGATTATGTTTGATTACCCAAATCCTAACAGGATTATTCTTAGCCATGCACTACACCTCTGATATCTCAACCGCATTCTCATCAGTAACCCATATCTGCCGGGACGTCAACTACGGCTGACTTATCCGGAGCCTACATGCCAATGGAGCATCGTTTTTCTTCATCTGTCTTTACATACACATCGCACGAGGCCTGTATTACGGATCATATCTTTATAAAGAAACCTGAAACGTTGGTGTAGTCCTGTTTCTTCTGGTTATGATGACAGCCTTTGTTGGATACGTTCTTCCATGAGGACAAATATCTTTTTGAGGTGCTACCGTAATTACAAATCTCCTGTCTGCAGTCCCCTACATAGGGGACACCCTAGTTCAATGAATCTGAGGAGGATTCTCAGTAGACAATGCAACACTCACACGATTCTTCGCGTTCCACTTCCTGCTGCCATTTGTCATTGCCGGGGCAACCATCCTGCACTTGTTATTTCTACACGAAACAGGCTCAAACAACCCGGCCGGACTAAATTCCGACGCGGACAAAATTTCTTTCCACCCATATTTCTCATATAAAGATCTTCTTGGCTTTGTGGCCATGTTACTAGCCCTTACTTCCCTAACATTATTTTCACCTAACCTATTGGGTGACCCGGAAAACTTTACTCCAGCAAACCCACTCGTAACACCTCCACATATTCAGCCAGAGTGATACTTTTTATTTGCCTACGCCATCCTCCGATCCATCCCAAATAAACTAGGGGGGGTTCTTGCACTACTATTCAGCATTCTAGTGCTAATGGTCGTGCCAATTTTACACACGTCCAAACAACGAGGACTAACTTTCCGCCCTATAACCCAATTCCTATTCTGAACCCTGGTTGCAGATATATTTATTTTGACATGAATCGGAGGCATACCCGTAGAACACCCATACATTATTATTGGCCAGGTTGCATCAATTCTATACTTTGCACTGTTCCTCATTCTTGTCCCACTAGCAGGATGAGTGGAAAATAAAGCGTTGAAATGAGCTAY568608Iberochondrostomalemmingii      atggcaagcctacgaaaaactcacccgctaatgaaaatcgctaacgacgcgctagtcgacctaccaacaccatctaatatctcagtaatatgaaacttcggatctcttctaggattatgtttgattacccaaatcctaacaggattattcttggccatgcactacacctctgatatctcaaccgcattctcatcagtaacccatatctgccgggacgtcaactacggctgacttatccgtagcctacatgccaatggagcatcgtttttcttcatctgtctttacatacacatcgcacgaggcctgtattacggatcatatctttataaagaaacctgaaacattggtgtagtcctgtttcttctggttatgatgacagcctttgttggatacgttcttccatgaggacaaatatccttttgaggtgctaccgtaattacaaatctcctgtctgcagtcccctacataggagacaccctagttcaatgaatctgaagggggttctcagtagacaatgcaacactcacacgattcttcgcgttccacttcctgctgccatttctcattgccggggtaaccatcctgcacttgttatttctacacgaaacaggctcaaacaacccggccggactaaattccgacgcagacaaaatttctttccacccatatttctcatataaagatcttcttggctttgtggccatgttactagcccttacttccctaacattattttcacctaacctattgggtgacccggaaaactttactccagcaaacccactcgtaacacccccacatattcagccggaatgatactttttatttgcttacgccatcctccgatccatcccaaataaactaggaggggttcttgcactactattcagcattctagtgctaatggtcgtgccaattttacacacgtccaaacaacgaggactaactttccgccctataacccaattcctattctgaaccctagttgcagatatatttattttgacatgaatcgggggcatacccgtagaacacccatacattattattggccaagttgcatcaattctatactttgcactgttcctcattcttgtcccactagcaggatgagtagaaaataaagcgttgaaatgagctAJ698703Iberochondrostomalusitanicum    nnnnnnnnnnnnnnnnnnnnnnnnnnnnnnnnnnnnnnnnnnnnnnnnnnnnnnnncgacctcccgacaccatctaatatttcagcaatgtgaaacttcggatctcttctgggattatgtttgattacccaaatcctaacaggattattcttagccatgcattacacctctgacatctcaaccgcgttctcatcagtgacccacatctgccgagacgtcaactacggctgacttatccgaagcctacatgccaatggagcatcatttttcttcatctgtctttacatacacatcgcacgaggcctgtattatggatcatacctttataaagagacctgaaacattggtgtaatcctattccttctggttataataacagcctttgtcggatatgttcttccatgaggacaaatatccttttggggtgctaccgtaattacaaatctcctatctgcagtcccctacataggagataccctagttcaatgaatctgagggggattctcagtagacaatgcaacactcacacgattcttcgcattccacttccttctaccatttgtcgttgccggcgcaaccatcctgcacttgttatttctacacgaaacaggctcaaacaacccagccggactaaattccgacgcagacaaaatttctttccacccatatttctcatataaagatcttcttggctttgtggccatgttactagcccttacttccctaacattattttcccctaacctattgggtgacccggaaaactttaccccagcaaccccactcgtaacacccccacatattcagccagagtgatacttcttatttgcctacgccatcctccgatccatcccaaataaactaggaggggttcttgcactactattcagcattctagtgttaatagtcgtgccaattttacacacgtccaaacaacgaggactaactttccgcccactaacccaattcctattctgaaccctggttgcagatatatttattttgacatgaatcgggggcatacctgtagagcacccatatattattattggccaagtcgcatccattctatattttgcactatncctcattcttgtcccactagcaggatgaatggaaancnnngcatnnaaatgagnnAJ698702Iberochondrostomalusitanicum    nnnnnnnnnnnnnnnnnnnnnnnnnnnnnnnnnnnnnnnnnnnnnnnnnnnnnnnncgacctcccgacaccatctaatatttcagcaatgtgaaacttcggatctcttctgggattatgtttgattacccaaatcctaacaggattattcttagccatgcattacacctctgacatctcaaccgcgttctcatcagtgacccacatctgccgagacgtcaactacggctgacttatccgaagcctacatgccaatggagcatcatttttcttcatctgtctttacatacacatcgcacgaggcctgtattatggatcatacctttataaagagacctgaaacattggtgtaatcctattccttctggttataataacagcctttgtcggatatgttcttccatgaggacaaatatccttttggggtgctaccgtaattacaaatctcctatctgcagtcccctacataggagataccctagttcaatgaatctgagggggattctcagtagacaatgcaacactcacacgattcttcgcattccacttccttctaccatttgtcgttgccggcgcaaccatcctgcacttgttatttctacacgaaacaggctcaaacaacccagccggactaaattccgacgcagacaaaatttctttccacccatatttctcatataaagatcttcttggctttgtggccatgttactagcccttacttccctaacattattttcccctaacctattgggtgacccggaaaactttaccccagcaaacccactcgtaacacccccacatattcagccagagtgatacttcttatttgcctacgccatcctccgatccatcccaaataaactaggaggggttcttgcactactattcagcattctagtgttaatagtcgtgccaattttacacacgtccaaacaacgaggactaactttccgcccactaacccaattcctattctgaaccctggttgcagatatatttattttgacatgaatcgggggcatacctgtagagcacccatatattattattggccaagtcgcatccattctatattttgcactatcccnnnnnnnnnnnnnnnnnnnnnnnnnnnnnnnnnnnnnnnnnnnnnnnnnnnnnAM886164Chondrostomaolisiponensis      ATGGCAAGCCTACGAAAAACCCACCCGCTAATAAAAATCGCTAACGATGCGCTAGTCGACCTCCCAACACCGTCTAATATCTCAGCAATATGAAACTTCGGATCTCTTCTGGGATTATGTTTGATTACCCAAATCCTAACAGGATTATTCCTAGCCATGCATTACACCTCTGACATCTCAACTGCATTCTCATCAGTAACCCACATTTGCCGAGATGTTAACTACGGCTGACTTATCCGAAGCCTGCATGCCAATGGAGCATCATTTTTCTTCATCTGCCTTTACATACACATCGCACGGGGCCTGTATTACGGATCATACCTTTATAAAGAAACCTGAAACATTGGTGTAATCCTGTTCCTTCTAGTTATAATGACAGCCTTTGTTGGATACGTCCTTCCGTGGGGACAAATGTCCTTTTGAGGTGCTACCGTAATTACAAATCTTCTGTCTGCGGTGCCGTACATAGGGGACACCCTCGTTCAATGAATCTGAGGCGGATTCTCAGTAGACAATGCAACACTCACACGATTCTTCGCATTCCACTTCCTGCTACCATTTGTCGTTGCCGGCGCAACCATCCTGCACTTATTGTTCTTACACGAAACGGGGTCAAACAACCCGGCCGGACTAAACTCCGACGCAGACAAAATTTCTTTCCACCCATATTTCTCATATAAAGATCTTCTTGGCTTTGTAGCCATATTATTAGCCCTTACTTCCCTAACATTATTTTCTCCAAACCTATTAGGTGACCCAGAAAACTTTACTCCAGCAAACCCACTCGTAACACCCCCACATATCCAGCCAGAGTGATACTTCTTATTTGCCTACGCCATCCTTCGATCCGTCCCAAATAAACTAGGAGGGGTACTTGCGCTACTATTCAGCATTCTAGTGCTAATAATTGTGCCAATTTTACATACATCCAAACAACGAGGACTAACTTTCCGACCAGTGACCCAATTCCTATTCTGAACCCTAGTTGCAGATATATTTATTTTGACATGAATCGGGGGCATACCCGTAGAACACCCATATATTATTATTGGCCAAATCGCATCCATTCTATACTTCGCGCTATTCCTCATTCTTGTCCCGCTAGCAGGATGGATAGAAAATAAAGCATTGAAATGAGCCDQ447737Iberochondrostomaoretanum       nnnnNNNNNNNNNNNNNNNNNNNNNNNNNNNNNNNNNNNNNNNNNNNNNNNNNNNNCGACCTACCAACACCATCTAATATCTCAGTAATGTGAAACTTCGGATCTCTTCTGGGATTATGTTTGATTACCCAAATCCTGACAGGATTATTCTTAGCCATGCACTACACCTCTGATATCTCAACCGCATTCTCATCAGTGACCCACATCTGCCGGGACGTTAACTACGGCTGACTTATCCGGAGCCTACATGCCAATGGGGCATCATTTTTCTTCATCTGTCTTTACATACACATCGCACGAGGCCTGTATTATGGATCATATCTTTATAAAGAAACCTGAAACATTGGTGTAATCCTGTTTCTTCTGGTTATAATGACAGCCTTTGTTGGATACGTTCTTCCATGAGGACAAATATCTTTTTGAGGTGCTACCGTAATTACAAATCTCCTGTCTGCAGTCCCCTACATAGGAGACACCCTAGTTCAGTGAATCTGAGGGGGCTTCTCAGTAGACAATGCAACACTCACACGATTCTTCGCATTCCACTTCCTGCTGCCATTTGTCATTGCCGGGGCAACCATCCTACACTTGTTATTTCTACACGAAACAGGCTCAAACAACCCGGCCGGACTAAATTCCGACGCAGACAAGATTTCTTTCCACCCATATTTCTCATATAAAGATCTTCTTGGCTTTGTGGCCATATTACTAGCCCTTACTTCCCTAACATTATTTTCCCCTAACCTATTGGGTGACCCGGAAAACTTTACCCCAGCAAACCCACTCGTAACACCCCCACATATTCAACCAGAGTGATACTTTTTATTTGCCTACGCCATCCTCCGATCCATCCCAAATAAACTAGGAGGGGTTCTTGCACTACTATTCAGCATTCTAGTGCTAATGATCGTGCCAATTTTACACACGTCCAAACAGCGAGGACTAACTTTCCGGCCAATAACCCAATTCCTATTCTGAACCCTGGTTGCAGATATATTTATTTTGACATGAATCGGGGGTATACCCGTAGAACACCCATATATTATCATTGGCCAAATNGCATCCATTCTATANNNNNNNNNNNNNNNNNNNNNNNNNNNNNNNNNNNNNNNNNNNNNNNNNNNNNNNNNNNNNNNNNNNHM560094Iberochondrostomaoretanum       NNNNNNNNNNNNNNNNNNNNNNNNNNNNNNNNNNNNNNNNNNNNNNNNNNNNNNNNCGACCTACCAACACCATCTAATATCTCAGTAATGTGAAACTTCGGATCTCTTCTGGGATTATGTTTGATTACCCAAATCCTGACAGGATTATTCTTAGCCATGCACTACACCTCTGATATCTCAACCGCATTCTCATCAGTGACCCACATCTGCCGGGACGTTAACTACGGCTGACTTATCCGGAGCCTACATGCTAATGGAGCATCATTTTTCTTCATCTGTCTTTACATACACATCGCACGAGGCCTGTATTATGGATCATATCTTTATAAAGAAACCTGAAACATTGGTGTAATCCTGTTTCTTCTGGTTATAATGACAGCCTTTGTTGGATACGTTCTTCCATGAGGACAAATATCTTTTTGAGGTGCTACCGTAATTACAAATCTCCTGTCTGCAGTCCCCTACATAGGAGACACCCTAGTTCAGTGAATCTGAGGGGGCTTCTCAGTAGACAATGCAACACTCACACGATTCTTCGCATTCCACTTCCTGCTGCCATTTGTCATTGCCGGGGCAACCATCCTACACTTGTTATTTCTACACGAAACAGGCTCAAACAACCCGGCCGGACTAAATTCCGACGCAGACAAGATTTCTTTCCACCCATATTTCTCATATAAAGATCTTCTTGGCTTTGTGGGCATATTACTAGCCCTTACTTCCCTAACATTATTTTCCCCTAACCTATTGGGTGACCCGGAAAACTTTACCCCAGCAAACCCACTCGTAACACCCCCACATATTCAACCAGAGTGATACTTTTTATTTGCCTACGCCATCCTCCGATCCATCCCAAATAAACTAGGAGGGGTTCTTGCACTACTATTCAGCATTCTAGTGCTAATGATCGTGCCAATTTTACACACGTCCAAACAGCGAGGACTAACTTTCCGGCCAATAACCCAATTCCTATTCTGAACCCTGGTTGCAGATATATTTATTTTGACATGAATCGGGGGTATACCCGTAGAGCACCCNNATATTATCATTNNNNNNNNNNNNNNNNNNNNNNNNNNNNNNNNNNNNNNNNNNNNNNNNNNNNNNNNNNNNNNNNNNNNNNNNNNNNNNNNNNNNNNNNNNHM560095Ladigesocyprisghigii            ATGGCAAGCCTACGAAAAACCCACCCACTCATAAAAATCGCCAACGACGCACTAGTTGACCTTCCAACACCATCTAATATCTCAGCACTATGAAACTTCGGATCCCTCTTAGGATTATGTCTAATTACCCAAATCCTAACGGGATTATTTTTAGCAATGCACTACACCTCCGACATCTCAACCGCATTCTCATCGGTAACCCATATCTGCCGGGATGTTAACTACGGCTGACTTATCCGAAATCTACATGCCAATGGGGCCTCTTTCTTCTTTATTTGCCTCTACATACATATTGCACGAGGCCTGTATTATGGCTCGTATTTATATAAAGAGACCTGAAACATTGGTGTGATTCTATTTCTCCTAGTTATAATAACCGCCTTCGTGGGCTACGTACTTCCATGAGGGCAGATATCATTCTGAGGTGCCACGGTAATTACAAACCTTCTCTCAGCAGTTCCCTACATAGGAGATATCCTTGTTCAGTGGATCTGGGGTGGATTTTCCGAANNNNNNNNNNNNNNNNNNNNNNNNNNNNNNNNNNNNNNNNNNNNNNNNNNNNNNNNNNNNNNNNNNNNNNNNNNNNNNNNNNNNNNNNNNNNNNNNNNNNNNNNNNNNNNNNNNNNNNNNNNNNNNNNNNNNNNNNNNNNNNNNNNNNNNNNNNNNNNNNNNNNNNNNNNNNNNNNNNNNNNNNNNNNNNNNNNNNNNNNNNNNNNNNNNNNNNNNNNNNNNNNNNNNNNNNNNNNNNNNNNNNNNNNNNNNNNNNNNNNNNNNNNNNNNNNNNNNNNNNNNNNNNNNNNNNNNNNNNNNNNNNNNNNNNNNNNNNNNNNNNNNNNNNNNNNNNNNNNNNNNNNNNNNNNNNNNNNNNNNNNNNNNNNNNNNNNNNNNNNNNNNNNNNNNNNNNNNNNNNNNNNNNNNNNNNNNNNNNNNNNNNNNNNNNNNNNNNNNNNNNNNNNNNNNNNNNNNNNNNNNNNNNNNNNNNNNNNNNNNNNNNNNNNNNNNNNNNNNNNNNNNNNNNNNNNNNNNNNNNNNNNNNNNNNNNNNNNNNNNNNNNNNNNNNNNNNNNNNNNNNNNNNNNNNNNNNNNNNNNNNNNNNNNNNNNNNNNNNNNNNNNNNNNNNNNNNNNNNNNNNNNNNNNNNHM560096Ladigesocyprisirideus           ATGGCAAGCCTACGAAAAACCCACCCACTCATAAAAATCGCCAACGACGCACTAGTTGACCTTCCAACACCATCTAATATCTCAGCACTATGAAACTTCGGATCCCTCTTAGGATTATGTCTAATTACCCAAATCCTAACGGGATTATTTTTAGCAATGCACTACACCTCCGACATCTCAACCGCATTCTCATCGGTAACCCATATCTGCCGGGATGTTAACTACGGCTGACTTATCCGAAATCTACATGCCAATGGGGCCTCTTTCTTCTTTATTTGCCTCTACATACATATTGCACGAGGCCTGTATTATGGCTCGTATTTATATAAAGAGACCTGAAACATTGGTGTGATTCTATTTCTCCTAGTTATAATAACCGCCTTCGTGGGCTACGTACTTCCATGAGGGCAGATATCATTCTGAGGTGCCACGGTAATTACAAACCTTCTCTCAGCAGTTCCCTACATAGGAGATATCCTTGTTCAGTGGATCTGGGGTGGATTTTCCGTAGACAACGCTACACTCACCCGATTCTTCGCATTCCACTTCCTCCTGCCCTTTGTCGTCGCAGGCGCAACCATCCTCCACCTATTATTTCTACACGAAACAGGATCAAACAACCCAGCTGGGCTAAATTCCGACGCCGACAAGATTTCTTTCCACCCGTATTTCTCATATAAAGACCTTCTTGGCTTTGTTGTTATATTACTAGCCCTTACCTCCTTAACATTATTTTCTCCTAACCTATTAGGTGACCCAGAAAACTTTACCCCAGCAAACCCACTGGTAACACCTCCACATATTCAGCCCGAATGGTACTTCTTATTTGCCTACGCTATCCTACGATCCATTCCGAATAAACTAGGAGGGGTACTTGCACTACTGTTCAGCATTCTCGTACTAATGGTTGTGCCACTATTACACACCTCGAAACAACGAGGACTAACTTTCCGCCCTGTAACACAATTTTTATTCTGGGCCCTCGTTGCAGATATGTTTATTCTGACATGAATCGGGGGCATACCTGTAGAACATCCATATATTATTATTGGCCAAGTCGCATCCGTTCTATATTTTTCACTCTTTCTAGTTCTTGCGCCACTAGCAGGATGACTGGAAAATAAGGCATTAAAATGAGCCHM560097Leucaspiusdelineatus            ATGGCAAGCCTACGAAAAACCCACCCACTAGTAAAAATCGCTAATGACGCTCTAGTCGACCTTCCAACACCGTCTAATATTTCAGCAATATGAAACTTTGGATCCCTTTTAGGATTATGTTTAATTACCCAAATCCTAACAGGATTATTTCTGGCCATGCATTACACCTCTGACATCTCAACCGCATTCTCATCAGTTACTCACATCTGCCGAGACGTCAACTACGGCTGGCTCATTCGAAATCTACATGCTAATGGAGCATCCTTCTTCTTCATCTGCCTGTACATGCACATCGCACGAGGTTTATACTACGGCTCATATCTTTATAAGGAGACCTGAAATATTGGGGTAGTACTATTTCTTCTAGTTATGATGACAGCCTTCGTGGGCTATGTACTTCCATGAGGACAAATGTCCTTCTGGGGCGCTACCGTAATCACAAACCTGCTCTCAGCAGTCCCCTACATAGGGGATACCCTTGTTCAATGAATCTGGGGNGGTNNCNCAGTNGANNNNNNNNNNNNNNNNNNNNNNNNNNNNNNNNNNNNNNNNNNNNNNNNNNNNNNNNNNNNNNNNNNNNNNNNNNNNNNNNNNNNNNNNNNNNNNNNNNNNNNNNNNNNNNNNNNNNNNNNNNNNNNNNNNNNNNNNNNNNNNNNNNNNNNNNNNNNNNNNNNNNNNNNNNNNNNNNNNNNNNNNNNNNNNNNNNNNNNNNNNNNNNNNNNNNNNNNNNNNNNNNNNNNNNNNNNNNNNNNNNNNNNNNNNNNNNNNNNNNNNNNNNNNNNNNNNNNNNNNNNNNNNNNNNNNNNNNNNNNNNNNNNNNNNNNNNNNNNNNNNNNNNNNNNNNNNNNNNNNNNNNNNNNNNNNNNNNNNNNNNNNNNNNNNNNNNNNNNNNNNNNNNNNNNNNNNNNNNNNNNNNNNNNNNNNNNNNNNNNNNNNNNNNNNNNNNNNNNNNNNNNNNNNNNNNNNNNNNNNNNNNNNNNNNNNNNNNNNNNNNNNNNNNNNNNNNNNNNNNNNNNNNNNNNNNNNNNNNNNNNNNNNNNNNNNNNNNNNNNNNNNNNNNNNNNNNNNNNNNNNNNNNNNNNNNNNNNNNNNNNNNNNNNNNNNNNNNNNNNNNNNNNNNNNNNNNNNNNNNNNNNNNNNY10447Leucaspiusdelineatus              atggcaagcctacgaaaaacccacccactaataaaaatcgctaatgacgctctagttgaccttccaacaccatctaatatttcagcaatatgaaactttggatcccttttaggattatgcttaattgcccaaatcctaacgggattattcctggccatgcactacacctctgacatctcgaccgcattctcatcagttactcacatctgccgagacgtcaactacggttggctcattcgaaatctacatgctaatggggcatccttcttcttcatctgtctgtatatacacatcgcacgaggtttatactacggctcatatctttataaagagacctgaaatattggagtagtactatttcttctggttatgatgacagccttcgtgggctatgtattaccatgaggacaaatgtccttctggggcgctaccgtgattacaaacctcctctcagcggtcccctacataggagacacccttgttcaatgaatctggggtggtttctcagtagataatgcaaccctcacacgattcttcgcattccacttcctcttacctttcgttgttgcgggcgcgaccgtcctccaccttctattcctacacgagacagggtcgaataacccggccggactaaattctgacgcggacaaaatttctttccacccctacttctcttacaaagaccttctcggcttcgtaattatattgctagccctcacctcgctggcgctgttctcgcccaaccttttaggtgatccagaaaactttaccccagcaaaccctctcgtgacacccccacatatccagccagaatgatacttcttatttgcgtatgccatcctccggtctattcctaataaactaggcggggttcttgctctattattcagtattctggtgctaatagttgtgccgattatacatacctcaaaacaacgaggactaaccttccgtcccgtgacccaattcctattctgaaccctaggtgcagatatgatcatcttgacatgaattggaggcatacccgtagagcacccgtatgttattatcggtcaaggcgcatccatcctatactttgcactcttcctgattcttatcccactagcaggattaatggaaaataaagcattgaaatgagct  HM560115Leuciscuscflatus                ATGGCAAGCCTACGAAAAACCCACCCACTGATAAAAATCGCTAATGGAGCACTAGTCGACCTTCCAACCCCATCCAACATTTCAGCAATGTGAAACTTCGGATCCCTCCTGGGGCTATGTTTAATTACCCAAATCCTAACGGGACTATTCTTAGCTATGCATTACACCTCTGATATTTCAACCGCATTCTCATCGGTAACCCACATCTGCCGGGACGTTAACTACGGCTGGCTTATCCGAAATCTGCACGCCAACGGAGCATCGTTCTTCTTCATCTGTATTTATATACACATCGCGCGAGGCCTATATTACGGGTCCTATCTTTACAAGGAGACCTGAAATATTGGTGTAGTACTACTTCTCTTGGTTATGGCGACCGCCTTCGTGGGCTATGTACTTCCATGGGGTCAAATGTCTTTCTGAGGCGCTACCGTTATTACAAACCTACTATCAGCAGTCCCTTATATAGGCGACACCCTTGTTCAATGAATTTGAGGCGGCTTNNNNNNNNNNNNNNNNNNNNNNNNNNNNNNNNNNNNNNNNNNNNNNNNNNNNNNNNNNNNNNNNNNNNNNNNNNNNNNNNNNNNNNNNNNNNNNNNNNNNNNNNNNNNNNNNNNNNNNNNNNNNNNNNNNNNNNNNNNNNNNNNNNNNNNNNNNNNNNNNNNNNNNNNNNNNNNNNNNNNNNNNNNNNNNNNNNNNNNNNNNNNNNNNNNNNNNNNNNNNNNNNNNNNNNNNNNNNNNNNNNNNNNNNNNNNNNNNNNNNNNNNNNNNNNNNNNNNNNNNNNNNNNNNNNNNNNNNNNNNNNNNNNNNNNNNNNNNNNNNNNNNNNNNNNNNNNNNNNNNNNNNNNNNNNNNNNNNNNNNNNNNNNNNNNNNNNNNNNNNNNNNNNNNNNNNNNNNNNNNNNNNNNNNNNNNNNNNNNNNNNNNNNNNNNNNNNNNNNNNNNNNNNNNNNNNNNNNNNNNNNNNNNNNNNNNNNNNNNNNNNNNNNNNNNNNNNNNNNNNNNNNNNNNNNNNNNNNNNNNNNNNNNNNNNNNNNNNNNNNNNNNNNNNNNNNNNNNNNNNNNNNNNNNNNNNNNNNNNNNNNNNNNNNNNNNNNNNNNNNNNNNNNNNNNNNNNNNNNNNNNNNNNNNNNNNNNNNNHM560116Leuciscuscflatus                ATGGCAAGCCTACGAAAAACCCACCCACTGATAAAAATCGCTAATGGAGCACTAGTCGACCTTCCAACCCCATCCAACATCTCAGCAATGTGAAACTTCGGATCCCTCCTGGGGCAATGTTTAATTACCCAAATCCTAACGGGACTATTCTTAGCTATGCATTACACCTCTGATATTTCAACCGCATTCTCATCGGTAACCCACATCTGCCGGGACGTTAACTACGGCTGGCTTATCCGAAATCTGCACGCCAACGGAGCATCGTTCTTCTTCATCTGTATTTATATACACATCGCGCGAGGCCTATATTACGGGTCCTATCTTTACAAGGAGACCTGAAANATTGGTGTAGTACTACTTCTCTTGGTTATGGCGACCGCCTTCGTGGGCTATGTACTTCCATGGGGTCAAATGTCTTTCTGAGGCGCTACCGTTATTACAAACCTACTATCAGCAGTCCCTTANATAGGCGACACCCTTGTTCAATGAATCTGAGGCGGCTTCTCCNNNNNNNNNNNNNNNNNNNNNNNNNNNNNNNNNNNNNNNNNNNNNNNNNNNNNNNNNNNNNNNNNNNNNNNNNNNNNNNNNNNNNNNNNNNNNNNNNNNNNNNNNNNNNNNNNNNNNNNNNNNNNNNNNNNNNNNNNNNNNNNNNNNNNNNNNNNNNNNNNNNNNNNNNNNNNNNNNNNNNNNNNNNNNNNNNNNNNNNNNNNNNNNNNNNNNNNNNNNNNNNNNNNNNNNNNNNNNNNNNNNNNNNNNNNNNNNNNNNNNNNNNNNNNNNNNNNNNNNNNNNNNNNNNNNNNNNNNNNNNNNNNNNNNNNNNNNNNNNNNNNNNNNNNNNNNNNNNNNNNNNNNNNNNNNNNNNNNNNNNNNNNNNNNNNNNNNNNNNNNNNNNNNNNNNNNNNNNNNNNNNNNNNNNNNNNNNNNNNNNNNNNNNNNNNNNNNNNNNNNNNNNNNNNNNNNNNNNNNNNNNNNNNNNNNNNNNNNNNNNNNNNNNNNNNNNNNNNNNNNNNNNNNNNNNNNNNNNNNNNNNNNNNNNNNNNNNNNNNNNNNNNNNNNNNNNNNNNNNNNNNNNNNNNNNNNNNNNNNNNNNNNNNNNNNNNNNNNNNNNNNNNNNNNNNNNNNNNNNNNNNNNNNNNNNNNHM560098Leuciscusidus                   ATGGCAAGCCTACGAAAAACCCATCCGTTAATAAAAATCGCTAATGACGCACTAGTCGACCTTCCAACCCCATCCAACATTTCGGCCCTCTGAAACTTCGGGTCCCTCCTAGGATTATGTTTAATTACCCAAATCCTAACAGGATTATTTTTAGCCATGCACTACACCTCTGATATTTCAACCGCATTTTCATCAGTAACTCACATTTGTCGGGACGTTAACTACGGCTGGCTTATCCGAAACCTGCACGCCAACGGGGCGTCATTCTTCTTCATCTGTATTTATATACATATCGCGCGAGGCCTGTATTACGGGTCCTATCTTTATAAGGAGACCTGAAATATTGGTGTAGTACTACTTCTTCTAGTCATAGCAACCGCCTTCGTGGGCTATGTGCTTCCGTGGGGCCAAATATCTTTTTGAGGTGCTACTGTTATTACAAATCTCCTATCAGCAGTCCCTTATATGGGCGACACCCTTGTTCAATGAATTTGAGGCGGCTTCTCAGTAGACAACGCGACCCTCACCCGGTTCTTCGCATTCCACTTCCTCTTACCGTTCGTCGTCGCCGGCGCGACCGTCCTACACTTACTATTTCTACACGAAACAGGGTCAAATAACCCGGCCGGACTAAATTCTGACGCGGACAAAATTTCCTTCCACCCTTACTTCTCATACAAAGACCTTCTTGGCTTTGTAATCATATTACTAGCCCTCACCTCTCTGACACTATTTTCCCCTAATCTTCTAGGTGACCCAGAGAATTTTACCCCAGCAAACCCACTCGTGACTCCCCCACATATTCAGCCAGAATGATATTTCCTATTTGCCTACGCCATTCTCCGGTCCATCCCAAACAAACTAGGAGGGGTTCTTGCGCTACTATTTAGCATTCTTGTGCTAATAGTTGTGCCAATTTTACACACCTCAAAACAACGAGGACTAACTTTTCGACCTGTAACCCAATTCCTATTCTGAACCTTAATTGCAGATATAATTATCTTAACATGAATTGGAGGCATACCCGTAGAACACCCATATGTCATTATTGGCCAAGTCGCATCCGTTCTATACTTTGCACTCTTCCTCGTTCTTGTCCCACTAGCAGGATGAGTCGAAAATAAAGCATTGAAATGAGCCHM560099Leuciscusidus                   ATGGCAAGCCTACGAAAAACCCATCCGTTAATAAAAATCGCTAATGACGCACTAGTCGACCTTCCAACCCCATCCAACATTTCGGCCCTCTGAAACTTCGGGTCCCTCCTAGGATTATGTTTAATTACCCAAATCCTAACAGGATTATTTTTAGCCATGCACTACACCTCTGATATTTCAACCGCATTTTCATCAGTAACTCACATTTGTCGGGACGTTAACTACGGCTGGCTTATCCGAAACCTGCACGCCAACGGGGCGTCATTCTTCTTCATCTGTATTTATATACACATCGCGCGAGGCCTGTATTACGGGTCCTATCTTTATAAGGAGACCTGAAATATTGGTGTAGTACTACTTCTTCTAGTCATAGCAACCGCCTTCGTGGGCTATGTGCTTCCGTGGGGCCAAATATCTTTTTGAGGTGCTACTGTTATTACAAATCTCCTATCAGCAGTCCCTTATATGGGCGACACCCTCGTTCAATGAATTTGAGGCGGCTTCTCAGTAGACAACGCGACCCTCACCCGGTTCTTCGCATTCCACTTCCTCTTACCATTCGTCGTCGCCGGCGCGACCGTCCTACACTTACTATTTCTACACGAAACAGGGTCAAATAACCCGGCCGGACTAAATTCTGACGCGGACAAAATTTCCTTCCACCCTTACTTCTCATACAAAGACCTTCTTGGCTTTGTAATCATATTACTAGCCCTCACCTCTCTGACACTATTTTCCCCTAATCTTCTAGGTGACCCAGAGAATTTTACCCCAGCAAACCCACTCGTGACTCCCCCACATATTCAGCCAGAATGATATTTCCTATTTGCCTACGCCATTCTCCGGTCCATCCCAAACAAACTAGGAGGGGTTCTTGCACTACTATTTAGCATTCTTGTGCTAATAGTTGTGCCAATTTTACACACCTCAAAACAACGAGGACTAACTTTTCGACCTGTGACCCAATTCCTATTCTGAACCTTAATTGCAGATATAATTATCTTAACATGAATCGGAGGCATACCCGTAGAACACCCATATGTCATTATTGGCCAAGTCGCATCCGTTCTATACTTTGCACTCTTCCTCGTTCTTGTCCCACTAGCAGGATGAGTCGAAAATAAAGCATTGAAATGAGCCAY509823Leuciscusleuciscus              atggcaagcctacgaaaaacccatccattaataaaaatcgccaatggcgcactagtcgaccttccaaccccatccaacatttcagccctctgaaacttcgggtccctcctaggattatgtttaattacccaaatcctaacgggattatttttagccatgcactacacctctgatatttcgaccgcattttcatcagtaactcacatttgtcgggacgttaactacggctggcttatccggaacctacacgccaacggagcgtcattcttcttcatctgtatttatatacatatcgcgcgaggcctatattacgggtcctatctttataaggagacctgaaatatcggtgtggtactacttcttctagtcatagcaaccgccttcgtgggctatgtacttccgtggggtcaaatatctttttgaggtgccactgttattacaaatctcctntcagcggtcccttatataggcgacacccttgttcaatgaatttggggcggcttctcggtggacaacgcgaccctcacccggttcttcgcattccacttcctcctgccattcgtcgtcgccggcgcgaccgtcctacacttattatttctacacgaaacagggtcaaataacccagccggactaaattctgacgcggacaaaatttccttccacccgtacttctcatacaaagaccttcttggctttgtgatcatactactagctctcacctctctaacgctattttcccctaatcttctnggtgacccagagaattttaccccagcaaacccactcgtgactcccccacatattcagccagaatgatacttcctatttgcctacgccattctccggtccatcccaaacaagctaggaggggttcttgcactactatttagcattcttgtgctaatagttgtgccaattttacacacctcaaaacaacgaggactaacttttcgacctgtaacccaattcctattctgaaccttagttgcagatataattattctaacatgaattggaggcatacccgtagaacacccatatgtcattattggccaaatcgcatccggtttatactttgcactcttcctcgttcttgtcccactagcaggatgggtggaaaataaagcattggaatgagcc Y10449Leuciscusleuciscus                atggcaagcctacgaaaaacccatccattaataaaaatcgctaatgacgcactagtcgaccttccaaccccatccaacatttcagcactctgaaacttcggatccctcctaggattatgtttaattacccaaatcctaacgggattatttttagccatacactacacctctgatatttcgaccgcattttcatcagtaactcacattcgtcgggacgttaactacggctggctcatccgaaacctgcacgccaacggagcgtcattcttcttcatctgtatttatatgcatatcgcgcgaggcctatattacgggtcctatctttataaggagacctgaaatattggtgtggtactacttcttctagtcatagcaaccgccttcgtgggctatgtacttccctggggccaaatatctttttgaggtgctactgttattacaaatctcctatcagcagtcccttatatgggtgacacccttgttcaatgaatttggggcggcttctcggtagacaacgcgaccctcacccggttcttcgcattccacttcctcttaccattcgtcgtcgccggcgcaaccgtcctacacttactatttctgcacgagacaggatcaaataacccggccggactaaattctgacgcggacaaaatttccttccacccatacttctcatataaagaccttcttggcttggtgatcatactgctagccctcacctcgctaacgctattttcccctaatcttctaggtgacccagagaattttaccccagcaaacccactcgtgactcccccacatattcagccagagtgatacttcctatttgcctacgccattctccgatctatcccaaacaaactaggaggggttcttgcactactatttagcatccttgtgctaatagtcgtgccaattttacacacctcaaagcaacgaggactaacttttcgacctgtgacccaattcctattctgaaccttaattgcagatataattatcttaacatgaattggaggcatacccgtagagcacccatatgtcattattggccaaatcgcattcgttctatactttgcactcttcctcgttcttgttccactagcggggtgagtcgagaataaagcattgaaatgagccHM560100Leuciscusleuciscus              ATGGCAAGCCTACGAAAAACCCATCCGTTAATAAAAATCGCTAATGACGCACTAGTCGACCTTCCAACCCCATCCAACATTTCAGCCCTCTGAAACTTCGGGTCCCTCCTAGGGTTATGTTTAATTACCCAAATCCTAACAGGATTATTTTTAGCCATGCACTACACCTCTGATATTTCGACCGCATTTTCATCAGTAACTCACATTTGTCGGGACGTTAACTACGGCTGGCTTATCCGAAACCTGCACGCCAACGGGGCATCATTCTTCTTCATCTGTATTTATATACATATCGCGCGAGGCCTGTATTACGGGTCCTATCTTTATAAGGAGACCTGAAATATCGGTGTGGTACTACTTCTTCTAGTCATAGCAACCGCCTTCGTGGGCTATGTGCTTCCGTGGGGCCAAATATCTTTTTGAGGCGCTACTGTTATTACAAATCTCCTATCAGCAGTCCCTTATATGGGCGACACCCTTGTTCAATGAATTTGAGGCGGCTTCTCAGTAGACAACGCGACCCTCACCCGGTTCTTCGCATTCCACTTCCTCTTACCATTCGTCGTCGCCGGCGCGACCGTCCTACACTTACTATTTCTACACGAAACAGGATCAAATAACCCGGCCGGACTAAATTCTGACACGGACAAAATTTCCTTCCACCCTTACTTCTCATACAAAGACCTTCTTGGCTTTGTGATCATACTACTAGCCCTCACCTCTTTGACACTATTTTCCCCTAATCTTCTAGGTGACCCAGAgAATTTTACCCCAGCAAACCCACTCGTGACTCCCCCACaTATTCAGCCaGAATGATACTTCCTATTTGCCTACGCCATTCTCCGGTCCATCCCAAACAAACTAGGAGGGGTTCTTGCACTACTATTTAGCATTCTTGTGCTAATAGTTGTGCCAATTTTACACACCTCAAAACAACGAGGACTAACTTTTCGACCTGTAACCCAATTCCTATTCTGAACCTTAATTGCAGACATAATTATCTTGACATGAATTGGGGGCATACCCGTAGAACACCCATATGTCATTATTGGCCAAATCGCATCCGTTCTATACTTTGCACTCTTCCTCGTTCTTGTCCCACTAGCAGGATGGGTCGAAAATAAAGCATTGAAATGAGCCHM560101Leuciscusleuciscus              ATGGCAAGCCTACGAAAAACCCATCCGTTAATAAAAATCGCTAATGACGCACTAGTCGACCTTCCAACCCCATCCAACATTTCAGCCCTATGAAACTTCGGGTCCCTCCTAGGGTTATGTTTAATTACCCAAATCCTAACAGGATTATTTTTAGCCATGCACTACACCTCTGATATTTCGACCGCATTTTCATCAGTAACTCACATTTGTCGGGACGTTAACTACGGCTGGCTTATCCGAAACCTGCACGCCAACGGGGCATCATTCTTCTTCATCTGTATTTATATACATATCGCGCGAGGCCTATATTACGGGTCCTATCTTTATAAGGAGACCTGAAATATCGGTGTAGTACTACTTCTTCTAGTCATAGCAACCGCCTTCGTGGGCTATGTGCTTCCGTGGGGCCAAATATCTTTTTGAGGTGCTACTGTTATTACAAATCTCCTATCAGCAGTCCCTTATATGGGCGACACCCTTGTTCAATGAATTTGAGGCGGCTTCTCAGTAGACAACGCGACCCTCACCCGGTTCTTCGCATTCCACTTCCTCTTACCATTCGTCGTCGCCGGCGCGACCGTCCTACACTTACTATTTCTACACGAAACAGGATCAAATAACCCGGCCGGACTAAATTCTGACGCGGACAAAATTTCCTTCCACCCTTACTTCTCATACAAAGACCTTCTTGGCTTTGTGATCATACTGCTAGCCCTCACCTCTCTGACACTATTTTCCCCTAATCTTCTAGGTGACCCAGAGAATTTTACCCCAGCAAACCCACTCGTGACTCCCCCACATATTCAGCCAGAATGATACTTCCTGTTTGCCTACGCCATTCTCCGGTCCATCCCAAACAAACTAGGAGGGGTTCTTGCACTACTATTTAGCATTCTTGTGCTAATAGTTGTGCCAATTTTACACACCTCAAAACAACGAGGACTAACTTTTCGACCTGTAACCCAATTCCTATTCTGAACCTTAATTGCAGATATAATTATCTTGACATGAATTGGGGGCATACCCGTAGAACACCCATATGTCATTATTGGCCAAGTCGCATCCGTTCTATACTTTGCACTCTTCCTCGTTCTTGTCCCACTAGCAGGATGGGTCGAAAATAAAGCATTGAAATGAGCCAY026396Leuciscusschmidti               atggcaagcctacgaaaaacccacccactgataaaaatcgctaatggagcactagtcgaccttccaaccccatccaacatttcagcaatgtgaaacttcggatccctcctggggctatgtttaattacccaaatcctaacgggactattcttagctatgcattacacctctgatatttcaaccgcattctcatcggtaacccacatctgccgggacgttaactacggctggcttatccgaaatctgcacgccaacggagcatcgttcttcttcatctgtatttatatacacatcgcgcgaggcctatattacggttcctatctttacaaggagacctgaaatattggtgtagtactacttctcctggttatagcgaccgccttcgtgggctatgtacttccatggggtcaaatgtctttctgaggcgctaccgttattacaaacctactatcagcagtcccttatataggcgacacccttgttcagtgaatttgaggcggcttctcagtagacaacgcgaccctcacccggttcttcgcgttccacttcctcctgcccttcgtcgtcgccggcgcaaccgtcctacacttactatttttacacgaaacaggatcaaacaacccagccgggttaaattccgatgcggacaagatttccttccacccatacttctcgtacaaagatcttcttggctttgtagtcatactgctagccctcacctcgctaacgctattttcccctaatctcctaggtgatccagagaattttaccccagcaaacccactcgtgacccccccacatatccagcctgagtgatacttcttatttgcctacgccatcctccgatccatcccgaacaaactgggaggggttcttgcactactttttagcattctagtgttaatagttgtgcctatcttacacacctcgaaacaacgaggactaactttccgacctgtgacccagttcctattctgaaccctaattgcagacatggttatcttaacatggattggaggcatacccgtagaacatccatacgttattatcggtcagatcgcatccgtcctatactttgcactcttcctcgttcttgtcccactagcaggctgagtagaaaataaagcattgaaatgagccAB162651Leuciscuswaleckii               atggcaagcctacgaaaaacccacccattgataaaaatcgctaatgacgcactagtcgaccttccaaccccatccaacatttcagcgctctgaaacttcggatctctcctaggattatgtctaattacccaaatcctaacagggctattcctagccatgcactacacctccgatatttcaaccgcattttcgtcagtaactcatatctgccgggacgttaactacggctgacttatccgaaatctacacgccaacggagcctcattcttcttcatctgtatttatatgcatatcgcccgaggcctatattacgggtcctatctctacaaggagacctgaaatattggtgtagtactacttctcctggtcatgatgaccgccttcgtaggctatgtgcttccgtggggtcaaatatctttctgaggtgctaccgttattacgaacctcctatcagcagtcccttatatgggcgacacccttgttcaatgaatttggggcggcttctcagtagacaacgcaaccctcacccggttcttcgcattccacttcctcctgccgttcgtcatcgccggcgcgaccgtgctccatttactattcttacacgaaacaggatcaaacaacccagccgggctaaattccgacgcggacaaaatttccttccacccgtacttttcatataaagaccttcttggcttcgtgatcatactactagccctcacctctctggcgctattctcccccaacctgctaggtgacccggagaacttcaccccagcaaacccactcgtgacacctccacatattcaaccagagtggtacttcttgtttgcctacgccatcctccgctctatcccaaacaagctaggaggggttcttgcactactgtttagcattctagtgttaatagttgtgccgatcttacacacctcaaaacaacgaggactaaccttccgacctgtgacccaattcctattctgaaccctagtcgcagacataattatcctgacatgaattgggggtatacccgtagaacacccatatatcattatcggccaaatcgcatccgtcctatactttgcactcttcctcgttcttgtcccactggcaggatgagtggaaaataaagcgttgaaatgagccAY026406Mirogrexterraesanctae           atggcaagcctacgaaagacccacccactaataaaaatcgctaatggcgcactagtcgatcttccgacaccatctaacatttcagcactatgaaacttcggatcccttctaggattatgtttaattacccaaatcctcacgggattatttctggccatgcactatacctctgatatctcgaccgcattttcgtcagtcacccacatctgccgagacgttaactacggctgactaattcgaagcttacatgccaacggagcatcattcttctttatttgtctttacatacatattgcacggggcctatactacgggtcctatctttacaaggagacctgaaatattggcgtggtcttatttcttctggtcataataacagccttcgtcggctatgtacttccatgaggacaaatatctttttgaggtgccaccgtaatcactaatctcctctcagcagtcccttatataggggacaccctcgtccaatgaatctgaggcggtttttcagtagacaacgcaactctcacacggttcttcgcattccacttcctcctcccgttcgttgttacnggcgcaacccttatacacctgctatttctacacgaaacaggatcaaataacccagccggtttaaactccgacgcagataaaatttccttccacccatacttctcatataaagaccttcttggctttgtaattatgctactagcccttacttctttatcattattttctcctaacctattaggtgacccagaaaactttaccccagcaaacccactcgtgacaccaccacatatccagccagagtgatatttcttatttgcttatgccattctccgatctattccgaataaactaggaggggttcttgcactattatttagtattctagtgctaatagttgtgccaattttacatacctcaaaacaacgaggactaactttccgtcccataacacaattcttattttgaaccttagttgcagacataatcattctgacatgaattgggggcatacctgtagaacatccatacattattattggccaagttgcatccattctatattttgcactcttcctcattctcatcccagcagcaggatgaatggaaaacaaagccctgaaatgagcc U01318Notemigonuscrysoleucas            atggcaagcctacgaaaaacccacccactaataaaaatcgctaatgacgcactagtcgaccttccaacaccgtctaatatttcagcgctatgaaactttggatctctcctagggttatgtttaattacccaaatccttacgggactattcttagccatgcactacacctccgatatctcaactgcattctcatcagtcacccatatttgccgagacgttaactacggctgacttatacgaaacttacatgccaacggcgcatctttcttcttcatctgtctttacatgcacattgcacgcggactatactatgggtcatatctttataaagagacctgaaacattggtgtagtattattcctcctagttataatgacagcctttgtcggctacgttctgccatgaggacaaatgtctttctgaggtgccaccgtaattaCaaaccttctttcagcagtgccctacataggggacacccttgtacagtgaatctgaggtggcttctcagttgacaacgcaaccctcacacggttcttcgcattccacttcctcctgccattcgtcgtcgccggcgcaactatcctacacttactcttcctacacgaaacaggatcaaacaaccccgccggactaaactcggacgcagacaaaatctctttccacccatacttctcatacaaagaccttcttggatttgtggtcatactactggccctcacctccctggccttattctcccctaacctgctaggtgatccagaaaactttaccccagcaaacccactcgtaacgccaccacatattcagccagaatgatattttctatttgcctatgccattcttcgttccatcccaaataaactaggaggggttcttgcactattgttcagcattctcgtgctaatagttgtgccgatcttacacacctcaaaacaacgaggactaactttccggcctctaacacaactcttattctgaaccctagttgcagacatagttattttgacatgaattggaggcatacccgtagaacacccatatattattattggacaagtcgcatccgtcttatactttgcactctttcttgtccttatcccactagcaggatgggtggaaaataaagcattgaaatgagctNC_008646Notemigonuscrysoleucas         atggcaagcctacgaaaaacccacccactaataaaaatcgctaatgacgcactagtcgaccttccaacaccgtctaatatttcagcgctatgaaactttggatctctcctagggttatgtttaattacccaaatccttacgggattattcttagccatgcactacacctccgatatctcaactgcattctcatcagtcacccatatttgccgggacgttaactacggctgactcatacgaaatttacatgccaacggcgcatccttcttcttcatctgtctttacatgcacattgcacgagggctatactatggctcatatctttataaagagacctgaaacattggtgtagtattatttctcctagttataatgacagcctttgttggctacgttctgccatggggacaaatgtctttctgaggtgccaccgtaatcacaaaccttctttcagcagtgccctacataggggacacccttgtgcagtgaatttgaggtggcttctcagttgacaacgcaaccctcacacgattcttcgcattccacttcctcctgccattcgtcgtcgccggtgcaactgtcctacacttactcttcctacacgaaacaggatcaaacaaccccgccggactaaactcggacgcagacaaaatctctttccacccatacttctcatacaaagaccttcttggatttgtggtcatactactagcccttacctccctagccttattctcccctaacctgctaggtgacccagaaaactttaccccagcaaacccactcgtaacgccaccacatattcagccagaatgatattttctatttgcctatgccatccttcgttccatcccaaataaactaggaggggttcttgcactattattcagcattctcgtgctaatagtcgtgcccatcttacacacctcaaaacaacgaggactaactttccgacctctaacacaactcctattctgaaccctggttgcagatatagttattctgacatgaattggaggcatacccgtagaacacccatatattattattgggcaagtcgcatccatcttatactttgcactctttcttgtgcttatcccactagcaggatgagtggaaaataaagcattgaaatgagccAB127393Notemigonuscrysoleucas          atggcaagcctacgaaaaacccacccactaataaaaatcgctaatgacgcactagtcgaccttccaacaccgtctaatatttcagcgctatgaaactttggatctctcctagggttatgtttaattacccaaatccttacgggattattcttagccatgcactacacctccgatatctcaactgcattctcatcagtcacccatatttgccgggacgttaactacggctgactcatacgaaatttacatgccaacggcgcatccttcttcttcatctgtctttacatgcacattgcacgagggctatactatggctcatatctttataaagagacctgaaacattggtgtagtattatttctcctagttataatgacagcctttgttggctacgttctgccatggggacaaatgtctttctgaggtgccaccgtaatcacaaaccttctttcagcagtgccctacataggggacacccttgtgcagtgaatttgaggtggcttctcagttgacaacgcaaccctcacacgattcttcgcattccacttcctcctgccattcgtcgtcgccggtgcaactgtcctacacttactcttcctacacgaaacaggatcaaacaaccccgccggactaaactcggacgcagacaaaatctctttccacccatacttctcatacaaagaccttcttggatttgtggtcatactactagcccttacctccctagccttattctcccctaacctgctaggtgacccagaaaactttaccccagcaaacccactcgtaacgccaccacatattcagccagaatgatattttctatttgcctatgccatccttcgttccatcccaaataaactaggaggggttcttgcactattattcagcattctcgtgctaatagtcgtgcccatcttacacacctcaaaacaacgaggactaactttccgacctctaacacaactcctattctgaaccctggttgcagatatagttattctgacatgaattggaggcatacccgtagaacacccatatattattattgggcaagtcgcatccatcttatactttgcactctttcttgtgcttatcccactagcaggatgagtggaaaataaagcattgaaatgagccAF090761Pachychilonmacedonicum          ATGGCAAGCCTACGGAAAACCCACCCACTAATAAAAATCGCTAATGATGCACTAGTTGACCTTCCAACACCATCTAATATCTCTGCACTATGAAATTTTGGATCTCTTTTAGGGTTAGGTTTAATTACTCAAATCCTAACAGGACTATTTCTAGCCATACACTATACCTCTGACATCTCGACCGCATTCTCATCAGTTACTCACATCTGCCGAGATGTAAATTATGGCTGACTTATTCGAAATCTCCATGCTAACGGTGCATCCTTCTTTTTCATCTGCATTTATATACACATTGCACGAGGCCTTTACTATGGCTCATACCTTTATAAGGAAACCTGAAACATTGGCGTAGTATTACTTCTTTTAGTTATAATAACAGCCTTTGTCGGGTATGTCCTTCCATGAGGACAGATGTCTTTTTGAGGTGCAACCGTTATCACAAACCTGCTCTCAGCAGTCCCCTACATAGGGGACACTCTTGTCCAATGAATCTGGGGTGGTTTCTCAGTAGATAACGCAACACTTACGCGATTCTTCGCATTCCACTTCCTTTTACCATTCGTCATTGCCGGCGCAACAATCTTACACCTTCTGTTTCTCCACGAAACAGGATCAAACAACCCAGCCGGCCTAAATTCGGACGCGGACAAAATTTCTTTCCACCCATACTTCTCATACAAAGACCTTCTAGGCTTTGTAATCATATTACTAGCTCTTACCTCTCTCGCACTATTTTCGCCCAATTTATTAGGTGACCCAGAAAACTTTACTCCAGCAAACCCACTGGTAACACCCCCACATATCCAGCCAGAATGATATTTCTTATTTGCTTATGCTATCCTCCGATCTATTCCTAATAAACTAGGAGGTGTTCTTGCACTACTATTCAGTATTTTAGTGCTAATAGTTGTGCCCATTCTACATACCTCAAAGCAACGAGGATTAACCTTCCGCCCTATAACCCAATTTTTATTCTGAACCTTAGTTGCAGACATAATTATCTTAACATGAATTGGAGGCATACCCGTAGAACATCCATATATTATCATTGGCCAAATCGCATCTATCTTATATTTTGCACTATTCCTCGTTCTTATTCCACTAGCAGGATGAGCAGAGAATAAAGCATTGAAATGAGCTHM560103Pachychilonmacedonicum          ATGGCAAGCCTACGGAAAACCCACCCACTAATAAAAATCGCTAATGATGCACTAGTTGACCTTCCAACACCATCTAATATCTCTGCACTATGAAATTTTGGATCTCTTTTAGGGTTATGTTTAATTACTCAAATCCTAACAGGACTATTTCTAGCCATGCACTATACCTCTGACATCTCGACCGCATTCTCATCAGTTACTCACATCTGCCGAGATGTAAATTATGGCTGACTTATTCGAAATCTCCATGCTAACGGTGCATCCTTCTTTTTCATCTGCATTTATATACACATTGCACGAGGCCTTTACTATGGCTCATACCTTTATAAGGAAACCTGAAACATTGGCGTAGTATTACTTCTTTTAGTTATAATAACAGCCTTTGTCGGCTATGTCCTTCCATGAGGACAGATGTCTTTTTGAGGTGCAACCGTTATCACAAACCTGCTCTCAGCAGTCCCCTACATAGGGGACACTCTTGTCCAATGAATCTGGGGTGGTTTCTCAGTAGATAACGCAACACTTACGCGATTCTTCGCATTCCACTTCCTTTTACCATTCGTCATTGCCGGCGCAACAATCTTACACCTTCTGTTTCTCCACGAAACGGGATCAAACAACCCAGCCGGCCTAAATTCCGACGCGGACAAAATTTCTTTCCACCCATACTTCTCATACAAAGACCTTCTAGGCTTTGTAATCATATTACTAGCTCTTACCTCTCTCGCACTATTTTCGCCCAATTTATTAGGTGACCCAGAAAACTTTACTCCAGCAAACCCACTGGTAACACCCCCACATATCCAGCCAGAATGATATTTCTTATTTGCTTATGCTATCCTCCGATCTATTCCTAATAAACTAGGAGGTGTTCTTGCACTACTATTTAGTATTTTAGTGCTAATAGTTGTGCCCATTCTACATACCTCAAAGCAACGAGGATTAACCTTCCGCCCTATAACCCAATTTTTATTCTGAACCTTAGTTGCAGACATAATTATCTTAACATGAATTGGAGGCATACCCGTAGAACATCCATATATTATCATTGGCCAAATCGCATCTATTTTATATTTTGCACTATTCCTCGTTCTTATTCCACTAGCAGGATGAGCAGAGAATAAAGCATTGAAATGAGCTAF090762Pachychilonpictum               atggcaagcctacgaaaaacccacccgctaataaaaattgctaatgatgcactagttgaccttccaacgccatctaacatttccgtaatatgaaattttggatctcttttaggattatgtctgattactcagatcctaacgggactatttctagccatgcactacacctctgacatctcaaccgcattttcatcagttacgcacatctgccgagacgttaattacggttgacttattcggaacatccatgccaacggtgcatccttctttttcatctgtatttatatacacatcgcgcgaggcctctactatggctcatacctttataaagaaacctgaaacattggcgtagtattacttctgttagttataataacagcctttgttggctatgtacttccctgaggacagatgtctttctgaggtgcaaccgtcattacaaacctgctctcagcagttccctacataggagacactcttgttcaatgaatctgaggtggtttctcagtagacaacgcaacgctgacgcgctttttcgcattccacttcctgctaccattcattattgccggcgcaactattttacaccttctgttcctccacgaaacaggatcaaacaacccggccggcctgaactccgacgcagataaaatttctttccacccatacttctcgtacaaagaccttctgggtttcgttattatacttctagctctcacctctctcgcgctattttcacctaacttattaggtgacccagaaaactttaccccagcaaatccactggtaacacctccacatattcagcctgagtgatatttcctgtttgcttacgccattctacgatctattcctaataaactaggaggggtacttgcactactatttagtattctagtgctaatagttgtgcccattttacatacttcaaaacaacgaggactaactttccgccctataacccaatttttattctgaaccttagttgcagacatgattattttaacatgaattgggggtatacccgtagaacatccatatatcatcattggccaaattgcatccattttatattttgcacttttccttattcttgttccactagcaggatgagcagaaaataaagcattgaaatgagctHM560102Pachychilonpictum               atggcaagcctacgaaaaacccacccgctaataaaaattgctaatgatgcactagttgaccttccaacgccaTCTAACATTTCCGTAATATGAAATTTTGGATCTCTTTTAGGATTATGTCTGATTACTCAGATCCTAACGGGACTATTTCTAGCCATGCACTACACCTCTGACATCTCAACCGCATTTTCATCAGTTACGCACATCTGCCGAGACGTTAATTACGGTTGACTTATTCGGAACATCCATGCCAACGGTGCATCCTTCTTTTTCATCTGTATTTATATACACATCGCGCGAGGCCTCTACTATGGCTCATACCTTTATAAAGAAACCTGAAACATTGGCGTAGTATTACTTCTGTTAGTTATAATAACAGCCTTTGTTGGCTATGTACTTCCCTGAGGACAGATGTCTTTCTGAGGTGCAACCGTCATTACAAACCTGCTCTCAGCAGTTCCCTACATAGGAGACACTCTTGTTCAATGAATCTGAGGTGGTTTCTCAGTAGACAACGCAACGCTGACGCGCTTTTTCGCATTCCACTTCCTGCTACCATTCATTATTGCCGGCGCAACTATTTTACACCTTCTGTTCCTCCACGAAACAGGATCAAACAACCCGGCCGGCCTGAACTCCGACGCAGATAAAATTTCTTTCCACCCATACTTCTCGTACAAAGACCTTCTGGGTTTCGTTATTATACTTCTAGCTCTCACCTCTCTCGCGCTATTTTCACCTAACTTATTAGGTGACCCAGAAAACTTTACCCCAGCAAATCCACTGGTAACACCTCCACATATTCAGCCTGAGTGATATTTCCTGTTTGCTTACGCCATTCTACGATCTATTCCTAATAAACTAGGAgGGGTACTTGCACTACTATTTAGTATTCTAGTGCTAATAGTTGCGCCCATTTTACATACTTCAAAACAACGAGGACTAACTTTCCGCCCTATAACCCAATTTTTATTCTGAACCTTAGTCGCAGACATGATTATTTTAACATGAATTGGGGGTGTACCCGTAGAACATCCATATATCATCATTGGCCAAATTGCATCCATTTTATATTTTGCACTTTTCCTTATTCTTGTTCCACTAGCAGGATGAGCAGAAAATAAAGCATTGAAATGAGCTHM560104Parachondrostomaarrigonis       atggcaagcctacgaggaacccacctcctgataaaaatcgctaacggcgcactagttgacctcccaacaccatctaatatctcagcaatgtgaaacttcgggtctctcctgggattatgtttaattacccaaatcctaacagggttattcttagccatgcattacacctctgatatctcgaccgcattctcatcagtaacccacatctgccgagatgttaactacggctgacttatccggagtctacatgccaatggggcatcctttttcttcatctgtctttatatacatatcgcacggggcctgtattatggctcatacctttataaagagacctgaaacattggtgtggtcctattccttctggttataatgacagcctttgtcggatacgtccttccgtggggacaaatatccttttgaggcgctaccgtaattacaaacctcctatctgcagtcccctatataggggataccctcgttcaatgaatctgaggtggattctcagtagacaatgcaactctcacacgattcttcgcgttccacttcctactaccgtttgtcgttgccggcgcaaccatcctacatttattgtttttacacgagacggggtcgaacaacccggccggactaaattcagacgcagacaagatttccttccacccgtatttctcatataaggaccttcttggctttgtggccatgttactagcccttacctccctaacattgttttcccctaacctactaggtgacccggaaaactttaccccagcaaaccctctcgtgacacccccacatatccagcctgagtgatacttcttatttgcctacgccattcttcgatctatcccaaataaactaggaggggttctcgcactactattcagcattctagtgctagtagtcgtgccaattttacacacatctaaacaacgaggactaactttccgcccagtgacccaattcttattctgaaccctcgttgcagacatatttattttgacatgaatcgggggtatacccgtagaacacccatatattctcattggccaagtcgcatccgtcctatactttgcactattcctcattcttgtcccactagcaggatgggtggaaaataaagcattgaaatgagccAY568598Parachondrostomaarrigonis       atggcaagcctacgaggaacccacctcctgataaaaatcgctaacggcgcactagttgacctcccaacaccatctaatatctcagcaatgtgaaacttcgggtctctcctgggattatgtttaattacccaaatcctaacagggttattcttagccatgcattacacctctgatatctcgaccgcattctcatcagtaacccacatctgccgagatgttaactacggctgacttatccggagtctacatgccaatggggcatcctttttcttcatctgtctttatatacatatcgcacggggcctgtattatggctcatacctttataaagagacctgaaacattggtgtggtcctattccttctggttataatgacagcctttgtcggatacgtccttccgtggggacaaatatccttttgaggcgctaccgtaattacaaacctcctatctgcagtcccctatataggggataccctcgttcaatgaatctgaggtggattctcagtagacaatgcaactctcacacgattcttcgcgttccacttcctactaccgtttgtcgttgccggcgcaaccatcctacatttattgtttttacacgagacggggtcgaacaacccggccggactaaattcagacgcagacaagatttccttccacccgtatttctcatataaggaccttcttggctttgtggccatgttactagcccttacctccctaacattgttttcccctaacctactaggtgacccggaaaactttaccccagcaaaccctctcgtgacacccccacatatccagcctgagtgatacttcttatttgcctacgccattcttcgatctatcccaaataaactaggaggggttctcgcactactattcagcattctagtgctagtagtcgtgccaattttacacacatctaaacaacgaggactaactttccgcccagtgacccaattcttattctgaaccctcgttgcagacatatttattttgacatgaatcgggggtatacccgtagaacacccatatattctcattggccaagtcgcatccgtcctatactttgcactattcctcattcttgtcccactagcaggatgggtggaaaataaagcattgaaatgagccHM560105Parachondrostomamiegii          atggcaagcctacgaaaaacccaccccctaataaaaatcgctaacggcgcactagtcgacctcccaacaccatctaatatctcagcaatgtgaaacttcgggtctctcctgggattatgtttaattacccaaatcctaacagggttattcttagccatgcattacacctctgatatctcaaccgcattctcatcagtaacccacatctgccgagatgtcaactacggctgacttatccgaagtctacatgccaatggagcgtcctttttcttcatctgtctttatatacatatcgcacggggcctgtattatggctcatacctttataaagagacctgaaacattggtgtggtcttattccttctggttataataacagcctttgtcggatacgtccttccgtggggacaaatatcctcttgaggcgctaccgtaattacaaacctcctatctgcggtcccctatataggagataccctcgttcaatgaatctgaggtggattctcagtagacaatgcaactctcacacgattctttgcgttccactttctactaccgtttgtcgttgccggcgcaaccatcctacatttattgtttttacacgagacggggtcgaataacccggccggactaaattcagacgcagacaagatttctttccacccgtatttctcatataaggaccttcttggctttgtggccatgttactagcccttacctccctaacattattttcccctaacctactaggtgacccggaaaactttaccccagcaaaccctctcgtgacacccccacatatccagcctgaatgatacttcttatttgcctacgccattcttcgatctatcccaaataaactaggaggggttcttgcactactattcagcattctagtgctaatagtcgtgccaattttacacacatctaaacaacgaggactaactttccgcccagtgacccaattcctattctgaaccctcgttgcagacatatttattttgacatgaatcgggggtatacccgtagaacacccatatattgtcattggccaagtcgcatccgtcctatactttgcactattcctcattcttgtcccgctagcaggatgggtggaaaataaagcattgaaatgagccDQ447732Parachondrostomahmiegii         atggcaagcctacgaaaaacccaccccctaataaaaatcgctaacggcgcactagtcgacctcccaacaccatctaatatctcagcaatgtgaaacttcgggtctctcctgggattatgtttaattacccaaatcctaacagggttattcttagccatgcattacacctctgatatctcaaccgcattctcatcagtaacccacatctgccgagatgtcaactacggctgacttatccgaagtctacatgccaatggagcgtcctttttcttcatctgtctttatatacatatcgcacggggcctgtattatggctcatacctttataaagagacctgaaacattggtgtggtcttattccttctggttataataacagcctttgtcggatacgtccttccgtggggacaaatatcctcttgaggcgctaccgtaattacaaacctcctatctgcggtcccctatataggagataccctcgttcaatgaatctgaggtggattctcagtagacaatgcaactctcacacgattctttgcgttccactttctactaccgtttgtcgttgccggcgcaaccatcctacatttattgtttttacacgagacggggtcgaataacccggccggactaaattcagacgcagacaagatttctttccacccgtatttctcatataaggaccttcttggctttgtggccatgttactagcccttacctccctaacattattttcccctaacctactaggtgacccggaaaactttaccccagcaaaccctctcgtgacacccccacatatccagcctgaatgatacttcttatttgcctacgccattcttcgatctatcccaaataaactaggaggggttcttgcactactattcagcattctagtgctaatagtcgtgccaattttacacacatctaaacaacgaggactaactttccgcccagtgacccaattcctattctgaaccctcgttgcagacatatttattttgacatgaatcgggggtatacccgtagaacacccatatattgtcattggccaagtcgcatccgtcctatactttgcactattcctcattcttgtcccgctagcaggatgggtggaaaataaagcattgaaatgagccAF533758Parachondrostomatoxostoma       atggcaagcctacgaaaaacccaccccctaataaaaatcgctaacggcgcactagtcgacctcccaacaccatctaatatctcagcaatgtgaaacttcgggtctctcctgggattatgtttgattacccaaatcctaacagggttattcttagccatgcattacacctctgatatctcgaccgcattctcatcagtaactcactcctgccgagatgttaactacggctgacttatccggagcctacatgccaatggagcatcctttttcttcatctgtctttatatacatatcgcacggggcctgtattatggctcatacctttataaagaaacctgaaacattggtgtagtcctattccttctggttataataacagcctttgtcggctacgtccttccatggggacaaatatccttttgaggcgctaccgtaattacaaacctcctatctgcggtcccctatataggggataccctcgttcaatgaatctgaggtggattctcagtagacaatgcaactctcacacgattcttcgcgttccacttcctactaccatttgtcgttgccggcgcaaccatcctacatttattgtttttacacgaaacggggtcgaataacccggccggactaaattcagacgcagacaagatttccttccacccgtatttctcatataaggaccttcttggctttgtggccatgttactagcccttacctccctaacattattttcccccaacctactaggtgacccggaaaactttaccccagcaaaccctctcgtgacacccccgcatatccagccggaatgatacttcttatttgcctacgccattcttcgatctatcccaaataaactaggaggggttcttgcactactattcagcattctagtgctaatagtcgtgccaattttacacacatccaaacaacgaggactaactttccggccagtgacccaattcctattctgaaccctcgtcgcagatatatttattttgacatgaatcgggggtatacccgtagaacacccatatattgtcattggccaaatcgcatccgtcctatactttgcactattcctcattcttgtcccgctagcaggatgggtggaaaataaagcattgaaatgagccAF533769Parachondrostomatoxostoma       atggcaagcctacgaaaaacccaccccctaataaaaatcgctaacggcgcactagtcgacctcccaacaccatctaatatctcagcaatgtgaaacttcgggtctctcctgggattatgtttgattacccaaatcctaacagggttattcttagccatgcatcacacctctgatatctcgaccgcattctcatcagtaactcacatctgccgagatgttaactacggctgacttatccggagcctgcatgccaatggagcatcctttttcttcatctgtctttatatacatatcgcacggggcctgtattatggctcatacctttataaagagacctgaaacattggtgtagtcctattccttctggttataataacagcctttgtcggctacgtccttccatggggacaaatatccttttgaggcgctaccgtaattacaaacctcctatctgcggtcccctatataggggataccctcgttcaatgaatctgaggtggattctcagtagacaatgcaactctcacacgattcttcgcgttccacttcctactaccgtttgtcgttgccggcgcaaccatcctacatttattgtttttacacgaaacggggtcgaataacccggccggactaaattcagacgcagacaagatttccttccacccgtatttctcatataaggaccttcttggctttgtggccatgttactagcccttacctccctaacattattttcccccaacctactaggtgacccggaaaactttaccccagcaaaccctctcgtgacacccccgcatatccagccggaatgatacttcttatttgcctacgccattcttcgatctatcccaaataaactaggaggggttcttgcactactattcagcattctagtgctaatagtcgtgccaattttacacacatccaaacaacgaggactaactttccggccagtgaccctattcctattctgaaccctcgtcgcagatatatttattttgacgtgaatcgggggtatacccgtagaacacccatatattgtcattggccaaatcgcatccgtcctatactttgcactattcctcattcttgtcccgctagcaggatgggtggaaaataaagcattgaaatgagccDQ447731Parachondrostomaturiense        NNNNNNNNNNTACGAAAAACCCACCCCCTGATAAAAATCGCTAACGGCGCACTAGTCGACCTCCCAACACCATCTAATATCTCAGCAATGTGAAACTTCGGGTCTCTCCTGGGGTTATGTTTAATTACCCAAATCCTAACAGGGTTATTCTTAGCCATGCATTACACCTCTGATATCTCAACCGCATTCTCATCAGTAACCCACATCTGCCGAGATGTTAACTACGGCTGACTTATCCGGAGTCTACATGCCAATGGAGCGTCCTTTTTCTTCATCTGTCTTTATATACATATCGCACGGGGCCTGTATTATGGCTCATACCTTTATAAAGAGACCTGAAACATTGGTGTGGTCCTATTCCTTCTGGTTATAATAACAGCCTTTGTCGGATACGTCCTTCCGTGGGGACAAATATCCTTTTGAGGCGCTACCGTAATTACAAATCTTCTATCTGCAGTCCCCTATATAGGGGATACCCTCGTTCAATGAATCTGAGGTGGATTCTCAGTAGACAATGCAACTCTCACACGATTCTTCGCGTTCCACTTCCTACTACCGTTTGTCGTTGCCGGCGCAACCATCCTACATCTATTGTTTTTACACGAGACGGGGTCGAATAACCCGGCCGGACTAAATTCAGACGCAGACAAGATTTCCTTCCACCCGTATTTCTCATATAAGGACCTTCTTGGCTTTGTGGCCATGTTACTAGCCCTTACCTCCCTAACATTATTTTCCCCTAACCTACTAGGTGACCCGGAAAACTTTACCCCAGCAAACCCTCTCGTGACCCCCCCACATATCCNGCCTGAGTGATACTTCCTGTTTGCCTACGCCATTCTTCGATCTATCCCAAATAAACTAGGAGGGGTTCTTGCGCTACTATTCAGCATTCTAGTGCTAATAGTCGTGCCAATTTTACACACATCTAAACAACGAGGACTAACTTTCCGCCCAGTGACCCAATTCTTATTCTGGACCCTCGTTGCAGACATATTTATTTTGACATGAATCGGGGGTATACCCGTAGAACACCCATATATTGTCATTGGCCAAGTCGCATCCCTCCTATACTTTGCACTATTCCTCATTCTTGTCCCGCTAGCAGGATGGGTGGAAAATAAAGCATTGAAATGAGCCAY568619Parachondrostomaturiensis       atggcaagcctacgaaaaacccaccccctgataaaaatcgctaacggcgcactagttgacctcccaacaccatctaatatctcagcaatgtgaaacttcgggtctctcctgggattatgtttaattacccaaatcctaacagggttattcttagccatgcattacacctctgatatctcaaccgcattctcatcagtaacccacatctgccgagatgttaactacggctgacttatccggagtctacatgccaatggagcgtcctttttcttcatctgtctttatatgcatatcgcacggggcctgtattatggctcatacctttataaagagacctgaaacattggtgtggtcctattccttctggttataataacagcctttgtcggatacgtccttccgtggggacaaatatccttttgaggcgctaccgtaattacaaatcttctatctgcagtcccctatataggggataccctcgttcaatgaatctgaggtggattctcagtagacaatgcaactctcacacgattcttcgcgttccacttcctactaccgtttgtcgttgccggcgcaaccatcctacatctattgtttttacacgagacggggtcgaataacccggccggactaaattcagacgcagacaagatttccttccacccgtatttttcatataaggaccttcttggctttgtggccatgttactagcccttacctccctaacattattttcccctaacctactaggtgacccggaaaactttaccccagcaaaccctctcgtgacacccccacatatccagcctgagtgatacttcttatttgcctacgccattcttcgatctatcccaaataaactaggaggggttcttgcactactattcagcattctagtgctaatagtcgtgccaattttacacacatctaaacaacgaggactaactttccgcccagtgacccaattcctattctgaaccctcgttgcagacatatttattttgacatgaatcgggggtatacccgtagaacacccatatattgtcattggccaagtcgcatccgtcctatactttgcactattcctcattcttgtcccgctagcaggatgggtggaaaataaagcattgaaatgagccHM560106Pelasguslaconicus               NNNNNNNNNNNNNNNNNNNNNNNNNNNNNNNNNNNNNNNNNNNNNNNNGCTCTAGTCGACCTCCCACCCCCATCCAATATTTGAGCATTATGAAGCTTCGGGTCCCTCCTAGGATTGTGTTTAATTACCCAAATCCTAACAGGATTATTTTTAGCTATACACTATACCTCCGATATTTCAACTGCATTTTCATCAGTGACACACATCTGCCGTGATGTTAATTATGGCTGACTCATCCGAAGCCTACACGCTAACGGAGCATCCTTCTTTTTTATCTGTATCTATATACACATCGCACGCGGCCTATATTATGGATCATACCTATACAAAGAAACCTGAAACATTGGCGTAGTTCTACTCCTCCTTGTTATAATAACAGCCTTCGTTGGTTATGTACTTCCTTGAGGTCAGATATCCTTTTGAGGTGCCACCGTAATTACAAACCTCCTCTCAGCAGTACCCTATATAGGGGATGCCCTTGTTCAATGAATCTGGGGCGGCTTCTCCGTTGATAACGCAACCCTAACACGGTTCTTCGCATTTCATTTCCTCTTCCCCTTCGTTATTGCTGGGGCAACGCTTCTACATCTCCTATTTCTACACGAAACAGGATCAAATAACCCCGCCGGATTAAACTCCGACGCAGACAAAATCTCCTTTCACCCCTACTTCTCATATAAAGACCTTCTTGGGTTCGTAATTATATTACTAGCCCTTACCTCCCTAGCCCTATTCTCCCCCAACCTGCTCGGCGACCCTGAAAACTTCACCCCAGCAAACCCACTTGTGACACCCCCACATATCCAACCCGAATGATATTTCCTATTTGCCTACGCCATCCTTCGATCCATTCCTAATAAACTAGGAGGAGTTCTTGCATTATTATTCAGTATTCTAGTACTAATGGTTGTACCAATTCTACACACCTCAAAGCAGCGAGGACTAACCTTCCGCCCCTTAACCCAGTTCCTATTCTGAACCCTCGTTGCAGATATAGTTATTTTGACATGGATTGGAGGCATACCTGTTGAACACCCATATATTATTATTGGCCAACTCGCATCCATTTTATACTTTACACTATTCCTTATTCTTGTGCCTCTAGCAGGATGAGTAGAAAATAAGGCACTAAAATGAGCCHM560107Pelasguslaconicus               NNNNNNNNNNNNNNNNNNNNNNNNNNNNNNNNNNNNNNNNNNNNNNNNGCTCTAGTCGACCTCCCACCCCCATCCAATATTTCAGCATTATGAAACTTCGGGTCCCTCCTAGGATTGTGTTTAATTACCCAAATCCTAACAGGATTATTTTTAGCTATACACTATACCTCCGATATTTCAACTGCATTTTCATCAGTGACACACATCTGCCGTGATGTTAATTATGGCTGACTTATCCGAAGCCTACACGCTAACGGAGCATCCTTCTTTTTTATCTGTATCTATATACACATCGCACGCGGCCTATATTATGGATCATACCTATACAAAGAAACCTGAAACATTGGCGTAGTTCTACTCCTCCTTGTTATAATAACAGCCTTCGTTGGTTATGTACTTCCTTGAGGTCAGATATCCTTTTGAGGTGCCACCGTAATTACAAACCTCCTCTCAGCAGTACCCTATATAGGAGATGCCCTTGTTCAATGAATCTGGGGCGGCTTCTCCGTTGATAACGCAACCCTAACACGGTTCTTCGCATTTCATTTCCTCTTCCCCTTCGTTATTGCTGGGGCAACGCTTCTACATCTCCTATTTCTACACGAAACAGGATCAAATAACCCCGCCGGATTAAACTCCGACGCAGACAAAATCTCCTTTCACCCCTACTTCTCATATAAAGACCTTCTTGGGTTCGTAATTATATTACTAGCCCTTACCTCCCTAGCCCTATTCTCCCCCAACCTGCTCGGCGACCCTGAAAACTTCACCCCAGCAAACCCACTTGTGACACCCCCACATATCCAACCCGAATGATATTTCCTATTTGCCTACGCCATCCTTCGATCCATTCCTAATAAACTAGGAGGAGTTCTTGCATTATTATTCAGTATTCTAGTACTAATGGTTGTACCAATTCTACACACCTCAAAGCAGCGAGGACTAACCTTCCGCCCCTTAACCCAGTTCCTATTCTGAACCCTCGTTGCAGATATAGTTATTTTGACATGGATTGGAGGCATACCTGTTGAACACCCATATATTATTATTGGCCAACTCGCATCCATTTTATACTTTACACTATTCCTTATTCTTGTGCCTCTAGCAGGATGAGTAGAAAATAAGGCACTAAAATGAGCCAF090768Pelasgusmarathonicus            atggcaagcctacgaaaaacacacccgctaataaaaattgctaatcatgccctagttgacctcccaaccccatccaatatttcagcactatgaaacttcgggtccctcctgggattatgcttaattacccaaatcctaacaggattatttttagccatgcattacacctctgatatttcaactgcgttttcatcagtaacgcacatctgccgtgacgttaattatggctggctcatccgaagcctacacgccaacggcgcatcattctttttcatctgtatttatatacacatcgcacgcggcctatattatggctcatacctttacaaaaagacctgaaacattggtgtagtactacttctcctagttataataacggcctttgttggctacgttcttccttggggtcaaatgtccttttgaggcgccaccgtgattacaaacctcctctcagcagtcccctatataggggacacccttgttcaatgaatctgaggcggcttctcagttgataacgcaaccctcacacggttctttgcattccacttcctcttcccctttgttatcactggagcaactctcctgcacctgctatttttacacgaaaccggatcaaacaacccggctggcctgaactccgacgcggacaaaatttcctttcacccctacttctcatataaggaccttcttgggtttgtaatcatattactggccctcacctccctatccttattctcccccaatctgttaggcgaccccgagaacttcactccagcaaacccacttgtgacacctccacatatccaacccgaatgatatttcctgtttgcctacgccatccttcgatctatccctaataaactaggaggagttcttgcactgttattcagtattctagtgctgatagttgtaccggttctacacacctcaaagcaacgaggactaactttccgccctctaacccagttcctattctgaaccctcgttgcagatatagttattttaacatgaattgggggtatgcctgttgaacacccatatattgttatcggccaagtcgcatccattttatactttgcgctattccttattctcgtgcctctagcagggtgaatagaaaacaaagcactgaaatgagccAY838939Pelasgusminutus                 atggcaagcctacgaaaaacacatccgctaataaaaattgctaatcatgccctagtcgacctcccaaccccatccaatatttcagcattatgaaatttcgggtccctcctaggattatgtctaattacccaaatcctaacaggactatttttagccatacattacacctccgatatctcaactgcattttcatcagttacacacatctgccgagacgtgaattatggttgactcatccgaaacctgcacgctaacggcgcatcgttctttttcatctgtatttatatacacatcgcacgcggcctatactatgggtcatacctttataaggagacctgaaacattggggtagtcctacttctcctggttataataacagccttcgttggctatgtacttccctgaggtcaaatatccttctgaggcgccactgtaattacaaatctcctttcagcagttccctacataggagatacccttgttcagtgaatctgagggggcttctcagttgacaacgcaaccctcacgcgattcttcgcatttcacttcctcttcccatttgttattgctggagcaactctcctacacctcctatttttacacgaaaccggatcaaataatccagctggattaaactccgacgcggacaaaatttcctttcacccctacttctcgtacaaagatctttttggatttgtaatcatattactagctctcacctccctagccctgttctcccctaacttgttgggtgaccccgagaactttaccccagcaaacccgcttgtaacacccccacatattcaacccgaatgatatttcctgtttgcctacgccatccttcgatctattcctaataaactaggaggagttcttgcactattatttagtattctagtattaataattgtgccgattctacacacctcaaaacaacgaggactaactttccgtccgctaacgcagttcctattctgagccctcgttgcagatatagttattttgacatgaattggaggcatacctgttgagcatccatatattattattggccaaatcgcatccattttatactttgcactattccttgttctgctacctctagcagggtgggtagaaaacaaagcactgaaatgagccHM560108Pelasgusprespensis              ATGGCAAGCCTACGAAAAACACATCCCCTAATAAAAATTGCTAACCACGCCCTGGTTGACCTCCCAACCCCATCCAACATCTCAGCACTGTGGAACTTTGGATCCCTCCTAGGATTATGTTTAATTACTCAAATCCTGACAGGACTATTTTTAGCCATACATTACACCTCTGATATTTCAACTGCATTCTCATCCGTAACACACATCTGCCGAGACGTTAATTATGGTTGACTCATCCGAAGCCTGCACGCTAACGGCGCGTCATTCTTTTTCATTTGTATTTATATACACATCGCACGCGGCCTATACTATGGGTCATACCTTTACAAAGAGACCTGAAACATCGGTGTAGTACTACTTCTCCTAGTTATAATAACAGCCTTTGTTGGCTATGTACTTCCTTGAGGTCAAATGTCCTTTTGAGGGGCCACCGTAATTACCAACCTCCTCTCAGCAGTCCCCTATATGGGAGACACCCTTGTTCAATGGATCTGAGGCGGCTTCTCAGTTGATAACGCAACCCTCACACGGTTCTTCGCATTTCATTTCCTCTTCCCATTTGTTATTGCTGGAGCAACTCTCCTACATCTGCTATTTTTACACGAAACCGGGTCAAATAACCCAGCCGGATTGAATTCGGACGCGGACAAAATCTCCTTTCACCCCTACTTCTCATATAAGGATCTTCTTGGATTCGTAATCATATTACTAGCCCTCACCTCCCTGGCCTTATTCTCTCCCAACCTGTTAGGCGACCCCGAAAACTTTACCCCAGCAAACCCACTCGTGACACCCCCACATATTCAGCCCGAATGGTATTTCCTATTTGCCTACGCCATCCTTCGATCCATCCCTAATAAACTAGGAGGAGTTCTTGCACTATTATTTAGTATTCTAGTGCTAATGGTTGTGCCAATCTTACACACCTCAAAGCAGCGAGGACTAACTTTCCGCCCCCTAACCCAATTCTTATTCTGAACCCTCGTTGCAGATATGATTATCTTAACATGGATTGGAGGCATACCTGTTGAACACCCATATATTGTTATTGGCCAAGTCGCATCCATTTTATACTTTGCACTATTCCTCGTTCTTGTACCTCTAGCAGGATGAGTAGAAAACAAAGCACTGAAATGAGCCAF090763Pelasgusprespensis              atggcaagcctacgaaaaacacatcccctaataaaaattgctaaccacgccctggttgacctcccaaccccatccaacatctcagcactgtggaactttggatccctcctaggattatgtttaattactcaaatcctgacaggactatttttagccatacattacacctctgatatttcaactgcattctcatccgtaacacacatctgccgagacgttaattatggttgactcatccgaagcctgcacgctaacggcgcgtcattctttttcatttgtatttatatacacatcgcacgcggcctatactatgggtcatacctttacaaagagacctgaaacatcggtgtagtactacttctcctagttataataacagcctttgttggctatgtacttccttgaggtcaaatgtccttttgaggggccaccgtaattaccaacctcctctcagcagtcccctatatgggagacacccttgttcaatggatctgaggcggcttctcagttgataacgcaaccctcacacggttcttcgcatttcatttcctcttcccatttgttattgctggagcaactctcctacatctgctatttttacacgaaaccgggtcaaataacccagccggattgaattcggacgcggacaaaatctcctttcacccctacttctcatataaggatcttcttggattcgtaatcatattactagccctcacctccctggccttattctctcccaacctgttaggcgaccccgaaaactttaccccagcaaacccactcgtgacacccccacatattcagcccgaatggtatttcctatttgcctacgccatccttcgatccatccctaataaactaggaggagttcttgcactattatttagtattctagtgctaatggttgtgccaatcttacacacctcaaagcagcgaggactaactttccgccccctaacccaattcttattctgaaccctcgttgcagatatgattatcttaacatggattggaggcatacctgttgaacacccatatattgttattggccaagtcgcatccattttatactttgcactattcctcgttcttgtacctctagcaggatgagtagaaaacaaagcactgaaatgagccHM560109Pelasgusstymphalicus            ATGGCAAGCCTACGAAAGACACACCCGCTAATAAAAATTGCTAACCACGCCCTAGTTGACCTCCCAACCCCATCCAATATTTCAGCACTATGAAACTTCGGGTCCCTCCTAGGATTGTGCTTAATTACCCAAATCCTAACAGGATTATTTTTAGCCATGCATTACACCTCTGATATTTCAACTGCATTTTCATCAGTAACGCACATCTGCCGTGACGTTAATTATGGCTGACTCATCCGAAATCTACACGCCAACGGCGCATCATTCTTTTTCATCTGTATTTATATACACATCGCACGCGGCCTATATTATGGCTCATACCTTTACAAAGAGACCTGAAACATTGGTGTAGTACTACTTCTCCTAGTTATGATAACGGCCTTTGTTGGCTACGTTCTTCCTTGAGGTCAAATGTCCTTTTGAGGCGCCACGGTAATTACGAACCTCCTCTCAGCAGTCCCCTATATAGGGGACACCCTTGTTCAATGAATCTGAGGGGGCTTCTCAGTTGACAACGCAACCCTCACACGGTTCTTTGCATTCCACTTCCTCTTCCCCTTTGTTATCGCTGGGGCAACTCTCCTACACCTGCTATTTTTACACGAAACCGGATCAAACAACCCAGCTGGCCTAAACTCCGACGCGGACAAAATTTCCTTTCACCCCTACTTCTCATATAAAGACCTTCTTGGGTTTGTAATTATATTGCTGGCCCTCACCTCCCTATCCTTATTCTCCCCCAATCTGTTGGGCGACCCCGAGAACTTCACCCCAGCAAACCCACTTGTGACACCTCCACATATCCAACCCGAGTGATATTTCCTGTTTGCCTACGCCATCCTTCGATCCATCCCTAATAAACTAGGAGGAGTCCTTGCACTGTTATTCAGTATTCTGGTGCTGATAGTTGTGCCGGTTCTACACACCTCAAAGCAACGAGGACTAACTTTTCGCCCTCTAACCCAGTTCCTATTCTGAACTCTCGTTGCAGATATAGTCATTTTAACATGGATTGGAGGCATACCTGTTGAACACCCATATATTGTTATCGGCCAAGTTGCATCCATTTTATATTTTGCACTATTCCTTATTCTCGTGCCTCTGGCAGGGTGAATAGAAAATAAAGCACTGGAATGAGCCAF090769Pelasgusthesproticus            atggcaagcctacgaaaaacacacccgctaataaaaattgctaatcatgccctagttgacctcccaaccccatccaatatctcagcactatgaaacttcgggtccctcctaggattgtgtttaattacccaaatcctaacaggattatttttggccatacattacacctctgatatttcaactgcattttcatcagtaacgcacatctgccgtgacgttaactatggctgactcatccgaagcctacacgccaacggcgcatcattctttttcatttgtatttatatacacatcgcacgcggcctatattatggctcatacctttacaaagagacctgaaacattggtgtagttctacttctcctagttatgataacggcctttgtcggctacgttctcccttggggtcaaatgtccttttgaggcgccaccgtgattacaaacctcctctcagcagtcccctatataggggacgcccttgttcaatgaatctgaggcggcttctcagttgataacgcaacccttacacggttctttgcattccatttcctcttcccctttgttatcgctggggcaactctcctacacctgctatttttacacgaaaccggatcaaataacccggctggcctaaactccgacgcggacaagatttcctttcacccctacttctcgtataaagaccttcttgggtttgtaattatattgctggccctcacctccctgtccttatcctcccccaatctgttaggcgaccccgaaaacttcaccccagcaaacccccttgtaacacccccacatattcaacccgagtgatatttcctgtttgcctacgccatccttcggtctatccctaataaactaggaggagttcttgcactgttattcagtattctagtgctgatggttgtgccagtcctacacacctcaaagcagcgaggactaactttccgccctttaacccaattcctattctggactctcgttgcagatatagttattttaacatgaattggaggcatacctgttgaacacccatatattgttatcggccaaatcgcgtccattttatactttgcactattccttgttctcgcgcctctagcagggtgaatagaaaacaaagcactgaaatgagccNC_008663Pelecuscultratus              ATGGCAAGCCTACGAAAAACTCACCCGCTAATAAAAATCGCTAACGGCGCACTAGTCGACCTCCCGACACCATCTAATATTTCAGCACTATGAAACTTCGGATCCCTCCTAGGACTATGTCTAATTACCCAAATCCTAACGGGACTATTCTTAGCTATACATTATACTTCTGATATCTCAACTGCGTTTTCATCAGTAACTCACATCTGTCGAGACGTAAACTATGGCTGACTTATCCGAAACCTACATGCTAACGGAGCATCATTCTTCTTCATCTGTATCTACATACACATTGCACGAGGCCTATACTACGGGTCATACCTTTATAAAGAAACCTGAAACATTGGTGTAGTCCTACTTCTTCTAGTTATAATAACAGCCTTCGTTGGCTATGTACTTCCATGAGGACAAATATCTTTCTGAGGCGCCACCGTAATTACAAATTTACTCTCAGCAGTCCCTTACATGGGAGACACCCTCGTTCAATGAATCTGAGGGGGCTTCTCAGTAGACAATGCAACTCTCACGCGATTCTTCGCATTCCACTTTCTACTCCCGTTCGTTATTGCCGGCGCAACCATCTTACACCTACTATTCTTACACGAAACGGGATCAAACAACCCAGCTGGATTAAACTCCGACGCAGATAAAATTTCATTCCACCCATACTTCTCATACAAAGACCTCCTTGGCTTTGTAGTAATACTATTAGCCCTCACATCTCTAGCATTGTTTTCCCCTAACCTACTAGGTGACCCAGAAAATTTCACCCCAGCAAACCCCTTAGTGACACCCCCGCATATTCAACCAGAGTGATACTTCTTATTTGCCTATGCCATCCTACGATCTATTCCAAACAAGCTAGGAGGGGTTCTCGCACTATTATTTAGCATTCTAGTGTTAATAGTAGTGCCAATCCTACACACCTCAAAACAACGAGGACTAACTTTCCGCCCCATGACCCAATTCTTATTTTGAACCCTAGTAGCAGACATGATCATCCTGACATGAATTGGAGGCATACCTGTAGAACACCCCTATATTATCATTGGCCAAGTCGCATCTATCTTATACTTTGCGCTCTTCCTTATTCTCATCCCGATAGCAGGATGAATGGAAAATAAGGCATTAAAATGAGCTAB239597Pelecuscultratus                atggcaagcctacgaaaaactcacccgctaataaaaatcgctaacggcgcactagtcgacctcccgacaccatctaatatttcagcactatgaaacttcggatccctcctaggactatgtctaattacccaaatcctaacgggactattcttagctatacattatacttctgatatctcaactgcgttttcatcagtaactcacatctgtcgagacgtaaactatggctgacttatccgaaacctacatgctaacggagcatcattcttcttcatctgtatctacatacacattgcacgaggcctatactacgggtcatacctttataaagaaacctgaaacattggtgtagtcctacttcttctagttataataacagccttcgttggctatgtacttccatgaggacaaatatctttctgaggcgccaccgtaattacaaatttactctcagcagtcccttacatgggagacaccctcgttcaatgaatctgagggggcttctcagtagacaatgcaactctcacgcgattcttcgcattccactttctactcccgttcgttattgccggcgcaaccatcttacacctactattcttacacgaaacgggatcaaacaacccagctggattaaactccgacgcagataaaatttcattccacccatacttctcatacaaagacctccttggctttgtagtaatactattagccctcacatctctagcattgttttcccctaacctactaggtgacccagaaaatttcaccccagcaaaccccttagtgacacccccgcatattcaaccagagtgatacttcttatttgcctatgccatcctacgatctattccaaacaagctaggaggggttctcgcactattatttagcattctagtgttaatagtagtgccaatcctacacacctcaaaacaacgaggactaactttccgccccatgacccaattcttattttgaaccctagtagcagacatgatcatcctgacatgaattggaggcatacctgtagaacacccctatattatcattggccaagtcgcatctatcttatactttgcgctcttccttattctcatcccgatagcaggatgaatggaaaataaggcattaaaatgagctHM560110Petroleuciscusborysthenicus     ATGGCAAGCCTACGAAAAACCCACCCACTGATAAAAATCGCCAATGGCGCACTAGTCGACCTTCCAACACCATCTAATATCTCAGCACTGTGAAACTTCGGGTCCCTCCTAGGATTGTGTTTAATTACCCAAATCCTCACGGGATTATTTTTAGCAATGCACTACACCTCCGACATCTCAACCGCATTCTCATCAGTAACTCACATCTGCCGGGATGTTAACTACGGCTGACTTATTCGAAATCTACATGCCAACGGCGCATCCTTCTTCTTCATCTGCCTTTACATACACATTGCGCGGGGCCTATATTATGGATCATACCTTTATAAAGAAACCTGAAACATTGGTGTAGTCCTATTTCTTCTAGTTATAATAACGGCCTTCGTCGGCTACGTTCTTCCGTGAGGGCAGATGTCCTTTTGAGGTGCCACAGTAATCACGAATCTACTCTCCGCAGTCCCTTACATAGGAGATACCCTTGTGCAATGAATCTGAGGCGGTTNNNNNNNNNNNNNNNNNNNNNNNNNNNNNNNNNNNNNNNNNNNNNNNNNNNNNNNNNNNNNNNNNNNNNNNNNNNNNNNNNNNNNNNNNNNNNNNNNNNNNNNNNNNNNNNNNNNNNNNNNNNNNNNNNNNNNNNNNNNNNNNNNNNNNNNNNNNNNNNNNNNNNNNNNNNNNNNNNNNNNNNNNNNNNNNNNNNNNNNNNNNNNNNNNNNNNNNNNNNNNNNNNNNNNNNNNNNNNNNNNNNNNNNNNNNNNNNNNNNNNNNNNNNNNNNNNNNNNNNNNNNNNNNNNNNNNNNNNNNNNNNNNNNNNNNNNNNNNNNNNNNNNNNNNNNNNNNNNNNNNNNNNNNNNNNNNNNNNNNNNNNNNNNNNNNNNNNNNNNNNNNNNNNNNNNNNNNNNNNNNNNNNNNNNNNNNNNNNNNNNNNNNNNNNNNNNNNNNNNNNNNNNNNNNNNNNNNNNNNNNNNNNNNNNNNNNNNNNNNNNNNNNNNNNNNNNNNNNNNNNNNNNNNNNNNNNNNNNNNNNNNNNNNNNNNNNNNNNNNNNNNNNNNNNNNNNNNNNNNNNNNNNNNNNNNNNNNNNNNNNNNNNNNNNNNNNNNNNNNNNNNNNNNNNNNNNNNNNNNNNNNNNNNNNNNNNNNNNNHM560111Petroleuciscusborysthenicus     ATGGCAAGCCTACGAAAAACCCACCCACTGATAAGAATCGCCAATGGCGCACTAGTCGACCTTCCAACACCATCTAATATCTCAGCACTGTGAAACTTCGGGTCCCTCCTAGGATTGTGTTTAATTACCCAAATCCTCACGGGATTATTTTTAGCAATGCACTACACCTCCGACATCTCAACCGCATTCTCATCAGTAACTCACATCTGCCGGGATGTTAACTACGGCTGACTTATTCGAAATCTACATGCCAACGGAGCATCCTTCTTCTTCATCTGCCTTTACATACACATTGCGCGGGGCCTATATTATGGATCATACCTTTATAAAGAAACCTGAAACATTGGTGTAGTCCTATTTCTTCTAGTTATAATAACGGCCTTCGTCGGCTACGTTCTTCCGTGAGGGCAGATGTCCTTTTGAGGTGCCACAGTAATCACGAATCTACTCTCCGCAGTCCCTTACATAGGAGATACCCTTGTGCAGTGAATCTGAGGCGGCTTCTCCGTAGACAACGCAACCCTCACACGATTCTTTGCCTTCCACTTCCTCCTGCCATTTGTCGTCGCGGGCGCGACCATCCTACACCTTCTATTCTTACACGAGACAGGATCGAACAACCCGGCCGGATTAAATTCGGACGCGGACAAAATTTCTTTCCATCCATACTTCTCATACAAGGACCTTCTTGGCTTTGTTATTATACTACTAGCCCTCACCTCTTTAGCATTATTTTCTCCTAACCTATTAGGTGACCCGGAAAACTTTACCCCAGCAAACCCCCTAGTAACGCCTCCACATATTCAGCCAGAATGATATTTCCTATTTGCCTATGCCATCCTCCGATCCATTCCAAATAAACTAGGAGGGGTTCTTGCACTACTATTTAGCATCCTAGTGCTAATGGTTGTGCCAGTCCTACACACGTCAAAGCGACGAGGACTAACTTTCCGCCCGGTGACCCAGTTCCTATTCTGAACCCTAGTTGCAGATATATTTATCTTGACATGAATTGGAGGCATGCCCGTAGAACACCCATATATTATCATTGGCCAAATCGCATCCATTCTATACTTTGCGCTCTTCCTCATTCTTGTACCACTAGCAGGATGAGTAGAAAATAAAGCATTGAAATGAGCCAJ252813Petroleuciscussmyrnaeus         atggcaagcctacgaaaaacccacccactgataaaaatcgccaatggcgcactagttgaccttccaacaccatctaatatttcagcactatgaaacttcggatccctcctaggattgtgtttaattacccaaatcctcacgggattatttttagcaatgcactacacctccgacatctcaactgcattctcatcagtaactcacatctgccgggatgttaactatggctgacttattcgaaatctgcatgccaacggcgcatccttcttcttcatctgcctttacatacacattgcgcgaggcctgtattatggatcatacctttataaagaaacctgaaacattggtgtagtcctatttcttctagttataataacagccttcgttggctacgttcttccgtgaggacagatgtccttttggggtgccacagtaatcacgaatctactctccgcagtcccttacataggagatatccttgtgcaatgaatctgaggcggtttctccgtagacaacgcaacgctcacacgattcttcgccttccacttcctcctgccatttgtcgtcgcaggcgcaaccatcctacaccttctattcttacacgaaacgggatcgaacaacccagccggattaaattcggacgcggacaaaatttctttccatccatacttctcatacaaggacctccttggctttgttattatgctgctagccctcacctctctagcattattttctcctaacctattaggtgacccggaaaactttaccccagcgaaccccctagtgacgcccccacatattcagccagaatgatacttcctatttgcctatgccatcctccgatctattccaaataaactaggaggggtccttgcactactatttagcattctagtgctaatagttgtgccaattctacacacctcgaagcaacgaggactaactttccgccccgtaacccaattcctattctgaaccctagttgcagacatgtttatcctgacatgaattggaggcatacccgtagaacacccatatatcatcattggccaagtcgcatccactctatactttgcactcttccttgttcttgtcccactagcaggatgggtggagaataaagcattgaaatgagccHM560114Petroleuciscussmyrnaeus         ATGGCAAGCCTACGAAAAACCCACCCACTGATAAAAATCGCCAATGGCGCACTAGTTGACCTTCCAACACCATCTAATATTTCAGCACTATGAAACTTCGGATCCCTTCTAGGATTATGTTTAATTACCCAAATCCTCACGGGATTATTTTTAGCAATGCACTACACCTCCGACATCTCAACTGCATTCTCATCAGTAACTCACATCTGCCGGGATGTTAACTATGGCTGACTTATTCGAAATCTGCATGCCAACGGCGCGTCCTTCTTCTTCATCTGCCTTTACATACACATTGCGCGAGGCCTGTATTATGGATCATACCTTTATAAAGAAACCTGAAACATTGGTGTAGTCCTATTTCTTCTAGTTATAATAACAGCCTTCGTTGGCTACGTTCTCCCGTGAGGGCAGATGTCCTTTTGGGGTGCCACAGTAATCACGAATCTACTCTCCGCAGTCCCTTACATAGGAGATATCCTTGTGCAATGAATCTGAGGCGGTTTCTCCGTAGACAACGCAACGCTCACACGATTCTTCGCCTTCCACTTCCTCCTGCCATTTATCGTCGCAGGCGCGACCATCCTACACCTTCTATTCTTACACGAAACGGGATCGAACAACCCAGCCGGATTAAATTCGGACGCGGACAAAATTTCTTTCCATCCATACTTCTCATACAAGGACCTCCTTGGCTTTGTTATTATGCTGCTAGCCCTCACCTCTCTAGCATTATTTTCTCCTAACCTATTAGGTGACCCGGAAAACTTTACCCCAGCAAACCCCCTAGTAACGCCCCCACATATTCAGCCAGAATGATATTTCCTATTTGCCTATGCTATCCTCCGATCTATTCCAAATAAACTAGGAGGGGTCCTTGCACTACTATTTAGCATTCTAGTGCTAATAGTTGTGCCAATTCTACACACCTCGAAGCAACGAGGACTAACTTTCCGCCCCGTAACCCAATTCCTATTCTGAACCCTAGTTGCAGACATGTTTATCCTGACATGAATTGGAGGCATACCCGTAGAACACCCATATATCATCATTGGCCAAGTCGCATCCATTCTATACTTTGCACTCTTCCTTGTTCTTGTCCCACTAGCAGGATGGGTGGAGAATAAAGCATTGAAATGAGCCHM560117Phoxinellusalepidotus           ATGGCAAGCCTACGAAAAACCCACCCACTAATAAAAATCGCTAACGACGCACTAGTTGACCTCCCAACACCATCTAATATCTCAGTAATGTGAAACTTCGGGTCTCTCCTGGGGTTGTGTCTAATTACCCAAATCCTAACAGGACTATTTTTAGCCATGCATTATACCTCTGATATCTCAACCGCATTCTCATCAGTAACTCACATCTGCCGAGATGTCAACTACGGCTGACTTATCCGAAGCCTACATGCCAACGGCGCATCCTTTTTCTTCATCTGTCTTTACATGCATATCGCACGAGGCCTTTATTACGGCTCATACCTTTATAAAGAGACCTGAAACATTGGCGTAGTCCTGTTCCTTCTAGTTATAATAACAGCCTTCGTCGGCTACGTTCTTCCATGAGGACAAATATCCTTTTGAGGTGCTACCGTAATCACAAACCTCCTCTCAGCGGTCCCTTACATAGGGGACACCCTTGTTCAATGAATCTGAGGCGGCTTCTCAGTAGACAATGCAACTCTCACACGGTTCTTCGCATTCCACTTCCTCCTACCATTTGTCGTCGCCGGCGCAACCATCCTACACTTATTATTTTTACACGAAACGGGATCAAACAACCCGGCTGGACTGAATTCCGACGCAGACAAAATTTCTTTCCACCCATATTTCTCATATAAAGACCTTCTTGGGTTTGTGGTCATATTATTAGCCCTTACCGCTCTAACATTATTTTCTCCCAACCTACTAGGTGACCCAGAAAACTTTACCCCTGCAAACCCACTCGTAACACCCCCACATATCCAGCCAGAATGATACTTCTTATTTGCCTACGCCATCCTTCGATCTATTCCAAATAAACTAGGAGGGGTTCTTGCACTACTATTCAGCATTCTAGTGCTAATAGTCGTACCGATCTTACATACGTCCAAACAACGAGGACTAACTTTTCGCCCAGTGACCCAATTTCTATTCTGAACCCTCGTTGCAGATATATTTATTTTGACATGAATTGGGGGTATACCCGTAGAACACCCATATATTGTCATTGGCCAAGTCGCATCCATCCTATACTTTGCACTATTCCTCGTTCTTGTCCCACTAGCGGGATGGCTGGAGAATAAAGCATTGAAATGAGCCHM560118Phoxinellusalepidotus           ATGGCAAGCCTACGAAAAACCCACCCACTAATAAAAATCGCTAACGACGCACTAGTTGACCTCCCAACACCATCTAATATCTCAGTAATGTGAAACTTCGGGTCTCTCCTGGGGTTGTGTCTAATTACCCAAATCCTAACAGGACTATTTTTAGCCATGCATTATACCTCTGATATCTCAACCGCATTCTCATCAGTAACTCACATCTGCCGAGATGTCAACTACGGCTGACTTATCCGAAGCCTACATGCCAACGGCGCATCCTTTTTCTTCATCTGTCTTTACATGCATATCGCGCGAGGCCTTTATTACGGCTCATACCTTTATAAAGAGACCTGAAACATTGGCGTAGTCCTGTTCCTTCTAGTTATAATAACAGCCTTCGTCGGCTACGTTCTTCCATGAGGACAAATATCCTTTTGAGGTGCTACCGTAATCACAAACCTCCTCTCAGCGGTCCCTTACATAGGGGACACCCTTGTTCAATGAATCTGAGGCGGCTTCTCAGTAGACAATGCAACTCTCACACGGTTCTTCGCATTCCACTTCCTCCTACCATTTGTCGTCGCCGGCGCAACCATCCTACACTTATTATTTTTACACGAAACGGGATCAAACAACCCGGCTGGACTGAATTCCGACGCAGACAAAATTTCTTTCCACCCATATTTCTCATATAAAGACCTTCTTGGGTTTGTGGTCATATTATTAGCCCTTACCGCTCTAACATTATTTTCTCCTAACCTACTAGGTGACCCAGAAAACTTTACCCCTGCAAACCCACTCGTAACACCCCCACATATCCAGCCAGAATGATACTTCTTATTTGCCTACGCCATCCTTCGATCTATTCCAAATAAACTAGGAGGGGTTCTTGCACTACTATTCAGCATTCTAGTGCTAATAGTCGTACCGATCTTACATACATCCAAACAACGAGGACTAACTTTTCGCCCAGTGACCCAATTTCTATTCTGAACCCTCGTTGCAGATATATTTATTTTGACATGAATTGGGGGTATACCCGTAGAACACCCATATATTGTTATTGGCCAAGTCGCATCCATCCTATACTTTGCACTATTCCTCGTTCTTGTCCCACTAGCGGGATGGCTGGAGAATAAAGCATTGAAATGAGCCAY838927Phoxinellusdalmaticus           atggcaagcctacgaaaaacccacccactaatgaaaatcgctaacgacgcactagttgaccttccaacaccatctaatatctcagtaatgtgaaacttcggatctctactggggttgtgtctaattacccaaatcctgacaggactgtttttagccatgcattacacctctgatatctcgaccgcattctcatcagtaactcacatctgccgagatgttaactacggctgacttatccgaagcctacatgccaacggcgcatcctttttcttcatctgtctttatatgcatattgcacgaggcctttattacggctcatacctttatgaagagacctgaaacattggcgtagtcctattccttctggttataataacagccttcgtcggctacgttcttccatgaggacaaatgtccttttgaggtgctaccgtaattacaaatctcctctcagcggtaccttacataggggacacccttgttcaatgaatctgaggcggcttctcagtagacaatgcaactctcacacggttcttcgcattccacttcctcctaccatttgtagtcgccggcgcaaccatcctacacttgttatttttacacgaaacgggatcgaacaacccggctggactgaattccgacgcagacaaaatttctttccacccatatttctcatataaagaccttcttggatttgtggccatattattagcccttaccgctctaacattgttttctcctaacctactaggtgacccagagaactttacccctgcgaatccactcgtaacacccccacatatccagccagaatgatatttcttatttgcctacgccatccttcgatctatcccaaataaactaggaggggtccttgcactactcttcagcattctagtgctaatcgtcgtaccagtcttacatacgtccaaacaacgaggactaacttttcgcccagtcacccaattcctattctggaccctcgttgcagatatatttattttgacatgaattggaggtatacccgtagaacacccatacattgttattggccaaatcgcatccgtcttatactttgcactgttcctcgttcttgtcccactagcgggatgactggagaataaagcattgaaatgagccHM560119Phoxinellusdalmaticus           ATGGCAAGCCTACGAAAAACCCACCCACTAATGAAAATCGCTAACGACGCACTAGTTGACCTTCCAACACCATCTAATATCTCAGTAATGTGAAACTTCGGATCTCTACTGGGGTTGTGTCTAATTACCCAAATCCTGACAGGACTGTTTTTAGCCATGCATTACACCTCTGATATCTCGACCGCATTCTCATCAGTAACTCACATCTGCCGAGATGTTAACTACGGCTGACTTATCCGAAGCCTACATGCCAACGGCGCATCCTTTTTCTTCATCTGTCTTTATATGCATATTGCACGAGGCCTTTATTACGGCTCATACCTTTATAAAGAGACCTGAAACATTGGCGTAGTCCTATTCCTTCTGGTTATAATAACAGCCTTCGTCGGCTACGTTCTTCCATGAGGACAAATGTCCTTTTGAGGTGCTACCGTAATTACAAATCTCCTCTCAGCGGTCCCTTACATAGGGGACACCCTTGTTCAATGAATCTGAGGCGGCTTCTCAGTAGACAATGCAACTCTCACACGGTTCTTCGCATTCCACTTCCTCCTACCATTTGTAGTCGCCGGCGCAACCATCCTACACTTGNTATTTTTACACGAAACGGGATCGAACAACCCGGCTGGACTGAANTCCGACGCAGACAAAATTTCTTTCCACCCATATTTCTCATATAAAGACNTTCTTGGGTTTGTGGCCNTATNATTAGCCCTTACCGCTCTAACATTGTTTTCTCCTAACCTACTAGGTGACCCAGAGAACTTTACCCCTGCGAATCCACTCGTAACACCCCCACATATCCAGCCAGAATGATATTTCTTATTTGCCTACGCCATCCTTCGATCTATCCCAAATAAACTAGGAGGGGTCCTTGCACTACTCTTCAGCATTCTAGTGCTAATCGTCGTACCAGTCTTACATACGTCCAAACAACGAGGACTAACTTTTCGCCCAGTCACCCAATTCCTATTCTGGACCCTCGTTGCAGATATATTTATTTTGACATGAATTGGAGGTATACCCGTAGAACACCCATACATTGTTATTGGCCAAATCGCATCCGTCTTATACTTTGCACTGTTCCTCGTTCTTGTCCCACTAGCGGGATGACTGGAGAATAAAGCATTGAAATGAGCCHM560120Phoxinelluspseudalepidotus      ATGGCAAGCCTACGAAAAACCCACCCACTAATAAAAATCGCTAACGACGCACTAGTTGACCTCCCAACACCATCTAATATCTCAGTAATGTGAAACTTCGGGTCTCTCCTGGGGTTGTGTCTAATTACCCAAATCCTGACAGGACTATTTTTAGCCATGCATTATACCTCTGATATCTCAACCGCATTCTCATCAGTAACTCACATCTGCCGAGATGTCAACTACGGCTGACTTATCCGAAGCCTACATGCCAACGGCGCATCCTTTTTCTTCATCTGCCTTTACATGCATATCGCACGAGGCCTTTATTACGGCTCATACCTTTATAAAGAGACCTGAAACATTGGGGTAGTCCTGTTCCTTCTAGTTATAATAACAGCCTTCGTCGGCTACGTTCTTCCATGGGGACAAATATCCTTTTGAGGTGCTACCGTAATCACAAACCTCCTCTCGGCGGTCCCTTATATAGGGGACACCCTTGTTCAATGAATCTGAGGCGGCTTCTCAGTAGACAATGCAACTCTCACACGGTTCTTCGCATTCCACTTCCTCCTACCATTTGTCGTCGCCGGCGCAACCATCCTGCACTTATTATTTTTACACGAAACGGGATCAAACAACCCGGCTGGTCTCAATTCCGACGCAGACAAAATTTCTTTCCACCCATATTTCTCATATAAAGACCTTCTTGGATTTGTGGTTATATTATTAGCCCTCACCGCTCTAACATTATTTTCTCCTAACCTACTAGGTGACCCAGAAAACTTTACCCCTGCGAACCCACTCGTAACACCCCCACATATCCAGCCAGAATGATACTTCTTGTTTGCCTACGCCATCCTTCGATCTATTCCAAATAAACTAGGGGGGGTTCTTGCACTACTATTTAGCATTCTAGTGTTAATGGTTGTCCCAATCTTACATACATCCAAACAACGAGGACTAACTTTTCGCCCGGTGACCCAATTTCTATTCTGAACCCTCGTTGCAGATATATTTATTTTGACATGAATTGGGGGTATGCCCGTAGAACACCCATATATTGTTATCGGCCAAGTCGCATCCATCCTATACTTCGCACTATTCCTCGTTCTTGTCCCACTAGCGGGATGGCTGGAGAATAAAGCATTGAAATGAGCCHM560121Phoxinelluspseudalepidotus      ATGGCAAGCCTACGAAAAACCCACCCACTAATAAAAATCGCTAACGACGCACTAGTTGACCTCCCAACACCATCTAATATCTCAGTAATGTGAAACTTCGGGTCTCTCCTGGGGTTGTGTCTAATTACCCAAATCCTGACAGGACTATTTTTAGCCATGCATTATACCTCTGATATCTCAACCGCATTCTCATCAGTAACTCACATCTGCCGAGATGTCAACTACGGCTGACTTATCCGAAGCCTACATGCCAACGGCGCATCCTTTTTCTTCATCTGCCTTTACATGCATATCGCACGAGGCCTTTATTACGGCTCATACCTTTATAAGGAGACCTGAAACATTGGGGTAGTCCTGTTCCTTCTAGTTATAATAACAGCCTTCGTCGGCTACGTTCTTCCATGGGGACAAATATCCTTTTGAGGTGCTACCGTAATCACAAACCTCCTCTCAGCGGTCCCTTATATAGGGGACACCCTTGTTCAATGAATCTGAGGCGGCTTCTCAGTAGACAATGCAACTCTCACACGGTTCTTCGCATTCCACTTCCTCCTACCATTTGTCGTCGCCGGCGCAACCATCCTGCACTTATTATTTTTACACGAAACGGGATCAAACAACCCGGCTGGTCTCAATTCCGACGCAGACAAAATTTCTTTCCACCCATATTTCTCATATAAAGACCTCCTTGGATTTGTGGTTATATTATTAGCCCTCACCGCTCTAACATTATTTTCTCCTAACCTACTAGGTGACCCAGAAAACTTTACCCCTGCGAACCCACTCGTAACACCCCCACATATCCAGCCAGAATGATACTTCTTGTTTGCCTACGCCATCCTTCGATCTATTCCAAATAAACTAGGGGGGGTTCTTGCACTACTATTTAGCATTCTAGTGTTAATGGTTGTCCCAATCCTACATACATCCAAACAACGAGGACTAACTTTTCGCCCAGTGACCCAATTTCTATTCTGAACCCTCGTTGCAGATATATTTATTTTGACATGAATTGGGGGTATGCCCGTAGAACACCCATATATTGTTATCGGCCAAGTCGCATCCATCCTATACTTCGCACTATTCCTCGTTCTTGTCCCACTAGCGGGATGGCTGGAGAATAAAGCATTGAAATGAGCCHM560122Phoxinusbigerri                 ATGGCAAGCCTACGAAAGACGCATCCACTAATAAAAATCGCCAACGGCGCACTCGTTGACCTCCCAACCCCCTCTAATATCTCTGCACTCTGGAACTTTGGTTCTCTTCTAGGGCTATGTTTAATTACCCAAATTCTCACAGGACTATTTCTGGCCATACACTATACATCTGATATCTCAACCGCATTTTCATCGGTTACCCATATCTGCCGGGACGTTAATTATGGATGACTAATCCGGAACATACACGCCAACGGCGCATCATTCTTCTTTATCTGTATTTATATGCATATTGCCCGGGGCCTTTATTATGGTTCATATCTTTATAAGGAAACCTGAAATATCGGGGTGGTTCTCCTCCTCTTGGTCATAATAACAGCCTTTGTGGGCTACGTCCTTCCCTGAGGCCAAATATCTTTTTGAGGGGCCACAGTAATTACGAACTTATTATCAGCAGTCCCCTATATAGGAGACATACTAGTTCAATGAATCTGGGGCGGCTTCTCAGTAGATAATGCAACCCTCACGCGATTCTTCGCGTTTCACTTCCTATTCCCATTTGTTATTGCCGGTGCAACCGTCCTGCATCTATTGTTCTTACACGAGACAGGCTCGAATAACCCCGCTGGTTTAAACTCGGATGCGGATAAAATTTCTTTTCACCCATACTTTTCTTATAAAGACCTTCTAGGCTTTGTAGTTATGCTACTAGCCCTTACATCACTGACCCTGTTTTCACCAAGCCTTCTAGGTGACCCAGAAAATTTTACGCCCGCAAACCCCCTCGTTACCCCGCCTCATATTCAGCCAGAATGATATTTCTTGTTTGCCTATGCTATCCTACGATCTATTCCCAACAAGCTAGGAGGCGTCCTTGCACTATTGTTTAGTATTTTGGTACTAATGGTCGTCCCTATCTTACACACCTCGAAGCAACGTGGATTAACTTTCCGCCCATTAACCCAATTTTTATTCTGAACCCTTGTGGCAGACATGCTTATTTTAACATGAATTGGGGGTATACCCGTAGAACATCCATACATTATTATTGGCCAAGTGGCGTCAGTTTTATACTTTGCACTCTTTCTCATCCTCACACCCCTGGCAGGATGGCTAGAGAATAAAACACTAAAATGAGCCHM560124Phoxinuslumaireul               ATGGCAAGCCTACGAAAGACGCATCCACTAATAAAAATCGCCAACGGCGCACTCGTTGACCTCCCGACACCCTCCAATATTTCTGCACTCTGGAACTTTGGTTCTCTTCTAGGACTATGTTTAATTACCCAAATTCTCACAGGACTGTTTCTAGCCATACATTATACATCTGACATCTCAACCGCATTTTCATCAGTTACCCATATCTGCCGAGACGTTAATTATGGGTGACTAATTCGAGATATACACGCCAACGGCGCATCATTCTTCTTTATCTGTATCTATATGCATATTGCCCGGGGTCTTTATTATGGCTCATATCTTTATAAGGAGACCTGAAACATCGGCGTAGTTCTACTCCTCCTGGTCATAATAACGGCCTTTGTAGGCTACGTCCTTCCCTGAGGCCAAATGTCTTTTTGAGGTGCCACAGTGATTACAAACTTGTTATCAGCAGTCCCCTACAAGGGAGACATACTAGTTCAGTGAATCTGGGGCGGCTTCTCAGTAGATAATGCAACCCTCACACGATTCTTCGCATTTCACTTCCTATTCCCATTTGTTATTGCCGGTGCAACCGTCCTGCACTTATTGTTTCTGCACGAGACGGGCTCGAATAACCCCGCCGGATTAAACTCAGATGCGGATAAAATTTCTTTTCACCCATACTTTTCTTATAAAGATCTTCTAGGATTTGTAGTAATACTACTAGCTCTTACATCACTAACCCTATTTTCACCAAGCCTACTAGGTGACCCAGAAAATTTTACCCCTGCAAATCCCCTCGTCACCCCACCTCATATTCAACCGGAGTGGTATTTCTTGTTTGCCTATGCTATCTTACGATCTATCCCTAACAAGTTGGGGGGTGTCCTTGCACTATTATTTAGTATCTTAGTATTAATAGTCGTCCCTATCTTACACACCTCAAAGCAACGAGGATTAACTTTCCGCCCATTAACCCAATTCTTATTCTGAACCCTTGTCGCAGACATAATTATTTTAACATGAATTGGGGGCATGCCCGTGGAGCACCCATACATTATTATTGGCCAAGTAGCGTCGATTTTATACTTTGCACTCTTTCTCGTCCTCACACCACTGGCAGGATGACTAGAAAATAAGACATTAAAATGAGCCY10448Phoxinusphoxinus                  atggcaagcctacgaaagacgcatccactaataaaaatcgccaaccacgctctcgttgacctcccaacaccctccaatatttctgcactctggaactttggctctcttctaggactatgtttgattacccaaattctcacaggactatttctagccatacattacacatctgacatctcaaccgcattttcatcggttacccacatctgccgggacgttaattatgggtgactaattcgaaatatacacgccaacggcgcatcattcttctttatctgtatttatatacatatcgcccgaggtctttattatggttcatatctttataaagaaacctgaaatattggtgtagttctcctcctcctggtcataataacagccttcgtcggctacgtccttccctgaggccaaatgtctttttgaggtgccacagtcattacaaacttattatcggcagtcccttacataggggatatgctggttcaatgaatctggggcggcttctcagtagataatgcaaccctcacacgattcttcgcatttcacttcctattcccatttgttattgccggtgcgaccgtcctgcacttgttatttttacacgagacaggctcaaataaccccgttggattaaactcggatgcagataaaatttcttttcacccatatttttcttataaagaccttctagggtttgtagtaatactactagctcttacatcactaaccctattttcaccaagcctattaggtgacccagaaaattttacccctgccaaccccctcgttaccccgcctcatattcaaccagagtggtatttcctattcgcctatgctatcttacgatctattcctaacaagttaggaggtgtccttgcactattatttagtatcttggtcctgatagtcgtccctattttacacacctccaagcaacgaggactaactttccgcccattaacccaatttttattctgaaccctcgtggcagatatggttattttaacatgaattgggggcatacccgtagaacacccatatattattattggtcaagtagcgtcaattttatactttgcactctttctcatcctcacaccactggcaggatggctagaaaataagacattaaaatgagccHM560124Protochondrostomagenei          ATGGCAAGCCTACGAAAAACCCACCCACTGATAAAAATCGCTAACGGCGCACTAGTCGACCTCCCAACACCATCTAATATCTCAGCAATGTGAAACTTCGGATCTCTCCTAGGATTGTGTTTAATTACCCAAATCCTGACAGGATTGTTCTTAGCCATGCATTACACCTCTGATATCTCAACCGCATTCTCGTCAGTAACCCATATCTGCCGAGACGTTAACTACGGCTGACTCATCCGAAGCCTACATGCCAACGGGGCATCCTTTTTCTTCATCTGTCTCTACATGCACATCGCACGAGGCCTGTATTACGGGTCATACCTCTATAAAGAGACCTGAAACATTGGTGTAGTCCTATTCCTTTTGGTTATAATAACAGCCTTTGTAGGCTACGTTCTTCCATGAGGACAAATGTCCTTTTGAGGTGCTACCGTTATTACAAACCTCCTCTCTGCGGTCCCTTATATGGGAGACACCCTTGTTCAATGGATCTGGGGAGGCTTCTCAGTAGACAACGCAACTCTCACACGATTCTTCGCGTTCCACTTCCTACTTCCGTTCGTCGTTGCCGGCGCAACCATCCTCCACTTATTGTTCTTACACGAAACGGGGTCGAACAACCCGGCCGGACTAAATTCCGACGCAGACAAAATTTCTTTCCACCCATATTTCTCATATAAAGACCTTCTTGGCTTTGTGGCCATGTTACTAGCCCTTACCTCCCTAACATTGTTTTCCCCTAACCTACTAGGTGACCCGGAAAACTTTACCCCAGCAAACCCACTGGTAACACCCCCACATATCCAGCCAGAGTGGTACTTCTTATTTGCCTACGCCATCCTTCGATCTATCCCAAATAAACTAGGAGGGGTTCTTGCACTACTATTCAGCATTCTAGTGCTAATAGTTGTGCCGATCTTGCATACATCCAAACAACGAGGACTAACTTTCCGCCCAGTGACCCAGTTCCTGTTCTGAACCCTAGTTGCAGATATATTTATTCTGACATGAATCGGGGGTATACCCGTAGAACACCCATATATCGTTATTGGCCAAATCGCATCCGTCCTATACTTTGCACTATTCCTGGTTCTTGTCCCACTAGCAGGATGGGTGGAAAATAAAGCATTGAAATGAGCCHM560125Protochondrostomagenei          ATGGCAAGCCTACGAAAAACCCACCCACTGATAAAAATCGCTAACGGCGCACTGGTCGACCTCCCAACACCATCTAATATCTCAGCAATGTGAAACTTCGGATCTCTCCTAGGATTGTGTTTAATTACCCAAATCCTGACAGGATTGTTCTTAGCCATGCATTACACCTCTGATATCTCAACCGCATTCTCGTCAGTAACCCATATCTGCCGAGACGTTAACTACGGCTGACTCATCCGCAGCCTACATGCCAACGGGGCATCCTTTTTCTTCATCTGTCTTTACATGCACATCGCACGAGGCCTGTATTACGGGTCATACCTCTATAAAGAGACCTGAAACATTGGTGTAGTCCTATTCCTTTTGGTTATAATAACAGCCTTTGTAGGCTACGTTCTTCCATGAGGACAAATGTCCTTTTGAGGTGCTACCGTTATTACAAACCTCCTCTCTGCGGTCCCTTATATGGGAGACACCCTTGTTCAATGGATCTGGGGAGGCTTCTCAGTAGACAACGCAACTCTCACACGATTCTTCGCGTTCCACTTCCTACTTCCGTTCGTCGTTGCCGGCGCAACCATCCTCCACTTATTGTTCTTACACGAAACGGGGTCGAACAACCCGGCCGGACTAAATTCCGACGCAGACAAAATTTCTTTCCNCCCATATTTCTCATATAAAGACCTTCTTGGCTTTGTGGCCATGCTACTAGCCCTTACCTCCCTAACATTGTTTTCCCCTAACCTACTAGGTGACCCGGAAAACTTTACCCCAGCAAACCCACTGGTAACACCCCCACATATCCAGCCAGAATGGTACTTCTTATTTGCCTACGCCATCCTTCGATCTATCCCAAATAAACTAGGAGGGGTTCTTGCACTACTATTCAGCATTCTAGTGCTAATAGTTGTGCCGATCTTGCATACATCCAAACAACGAGGACTAACTTTCCGCCCAGTGACCCAGTTCCTGTTCTGAACCCTAGTTGCAGATATATTTATTCTGACATGAATCGGGGGTATACCCGTAGAACACCCATATATCGTTATTGGCCAAATCGCATCCGTCCTATACTTTGCACTATTCCTGGTTCTTGTCCCACTAGCAGGATGGGTGGAGAATAAAGCATTGAAATGAGCCAY568601Pseudochondrostomaduriensis     atggcaagcctacgaaaaacccacccactaataaaaatcgctaacgacgcgctagtcgacctcccaacaccatctaatatctcagtaatgtgaaacttcggatctctcctaggattgtgtttgattacccaaatcctaacagggttattcttagctatacattacacctctgatatctcaaccgcattctcatcagtaacccacatctgccgagatgtcaactacggctgacttatccgaagcctacatgccaacggagcatcctttttcttcatctgtctttatatacatatcgcacggggcctatactatggatcatacctttataaagaaacctgaaacattggtgtagtcctgttccttctggttatgataacggcctttgtcggctacgttcttccatgaggacaaatatccttttgaggtgctactgtgattacaaacctcctgtctgcggtcccctacatgggagatactctcgttcaatgaatctgaggaggattctcagtagacaatgcaactctcacacgattcttcgcattccacttcctgctgccatttgtcgttgccggcgcaaccatcctacacttattgtttttacacgaaacgggatcaaacaacccggccggactgaattccgacgcagacaaaatttctttccacccatatttctcatataaagaccttctcggctttgtagctatattactagccctcaccgccctaacgctattttcccctaacctactaggtgacccggaaaactttaccccagcaaacccactcgtgacacccccacatatccagccagaatgatacttcttatttgcctacgccatccttcgatctattccaaacaaactaggaggggttcttgcactactattcagcattctggtgctattggtcgtgccaattttacacacatccaaacaacgaggactaactttccgcccggtgacccagttcctattctgaaccctggttgcagatatatttattttgacatgaatcgggggtatacccgtagaacacccatatattattattggccaagttgcatccatcctatactttgcactattcctcgttcttgtcccactagcgggatgagtggaaaataaagcattgaaatgagccAY568602Pseudochondrostomaduriensis     atggcaagcctacgaaaaacccacccactaataaaaatcgctaacgacgcgctagtcgacctcccgacaccatctaatatctcagtaatgtgaaacttcggatctctcctaggattgtgtttgattacccaaatcctaacaggattattcttagctatgcattacacctctgatatctcaaccgcattctcatcagtaacccacatctgccgagatgtcaactacggctgacttatccgaagcctacatgccaacggagcatcctctttcttcatctgtctttatatacatatcgcacggggcctatactatggatcatacctttataaagaaacctgaaacattggtgtggtcctgttccttctggttatgataacagcctttgtcggctacgttcttccatgaggacaaatatccttttgaggtgctactgtgattacaaaccttctgtctgcagtcccctacatgggagatactctcgttcaatgaatctgaggaggattctcagtagacaatgcaactctcacacgattcttcgcattccacttcctgctgccatttgtcgttgccggcgcaaccatcctacacttattgtttttacacgaaacgggatcaaacaacccggccggactgaattccgacgcagacaaaatttctttccacccatatttctcatataaagaccttctcggctttgtagctatattactagccctcaccgccctaacgctattttcccctaacctattaggtgacccggaaaactttaccccagcaaacccactcgtgacacccccacatatccagccagaatgatacttcttatttgcctacgccatccttcgatctattccaaacaaactagggggggttcttgcactactattcagcattctggtgctattggtcgtgccaattttacacacatccaaacaacgaggactaactttccgcccggtgacccagttcctattctgaaccctggttgcagatatatttattttgacatgaatcgggggtatacccgtagaacacccatatattattattggccaagttgcatccatcctatactttgcactattcctcgttcttgtcccactagcgggatgagtggaaaataaagcattgaaatgagccAY568610Pseudochondrostomapolylepis     atggcaagcctacgaaaaacccacccactaataaaaatcgctaacgacgcgctagtcgacctcccaacaccatctaatatctcggtaatatgaaacttcggatctctcctaggattgtgtttaattacccaaatcctaacaggattattcttagctatacattacacctctgatatctcaaccgcattctcatcagtaacccacatctgccgagacgtcaactatggctgacttatccgaagcctacatgccaacggagcatcctttttcttcatctgtctttatatacatatcgcacgaggcctatactatgggtcatacctttataaagaaacctgaaacattggtgtagtcctattccttctggttatgataacagcctttgtcggctacgttcttccatgaggacaaatatccttttgaggtgccactgtaattacaaacctcctatctgcagtcccctacatgggagatactctcgttcaatgaatctgaggaggattctcagtagacaatgcaactctcacacgatttttcgcattccacttcctactgccatttgtcgttgccggcgcaaccattctacacttattgtttttacacgagacggggtcaaacaacccggccggactaaattccgacgcagacaaaatttctttccacccatatttctcatataaagaccttctcggctttgtggctatgctactagcccttacctccctaacactgttttcccctaacctacttggtgatccagaaaactttactccagcaaacccactcgtgacacccccacatatccagccagaatgatacttcttatttgcctacgccattcttcgatctattccaaacaagctagggggggttcttgcactactattcagcattctagtgctattagttgtgccaattttacacacatccaaacaacgaggactaactttccgcccggtgacccaatttctattctgaaccctggttgcagatatatttattttgacatgaatcgggggtatacccgtagaacacccatatattattattggccaagtcgcatccatcctatactttgcactattcctcgttcttgtcccactagcaggatgggtagaaaataaagcattgaaatgagccAY568611Pseudochondrostomapolylepis     atggcaagcctacgaaaaacccacccactaataaaaatcgctaacgacgcgctagtcgacctcccaacaccatctaatatctcggtaatatgaaacttcggatctctcctaggattgtgtttaattacccaaatcctaacaggattattcttagctatacattacacctctgatatctcaaccgcattctcatcagtaacccacatctgccgagacgtcaactatggctgacttatccgaagcctacatgccaacggagcatcctttttcttcatctgtctttatatacatatcgcacgaggcctatactatgggtcatacctttataaagaaacctgaaacattggtgtagtcctattccttctggttatgataacagcctttgtcggctacgttcttccatgaggacaaatatccttttgaggtgccactgtaattacaaacctcctatctgcagtcccctacatgggagatactctcgttcaatgaatctgaggaggattctcagtagacaatgcaactctcacacgatttttcgcattccacttcctactgccatttgtcgttgccggcgcaaccatcctacacttattgtttttacacgagacggggtcaaacaacccggccggactaaattccgacgcagacaaaatttctttccacccatatttctcatataaagaccttctcggctttgtggctatgctactagcccttacctccctaacactgttttcccctaacctacttggtgatccagaaaactttactccagcaaacccgctcgtgacacccccacatatccagccagaatgatacttcttatttgcctacgccattcttcgatctattccaaacaagctagggggggttcttgcactactattcagcattctagtgctattagttgtgccaattttacacacatccaaacaacgaggactaactttccgcccggtgacccaatttctattctgaaccctggttgcagatatatttattttgacatgaatcgggggtatacccgtagaacacccatatattattattggccaagtcgcatccatcctatactttgcactattcctcgttcttgtcccactagcaggatgggtggaaaataaagcattgaaatgagccHM560126Pseudochondrostomahwillkommii   ATGGCAAGCCTACGAAAAACTCACCCACTAATAAAAATCGCTAACGACGCGCTAGTCGACCTCCCGACACCATCTAATATCTCGGTAATATGAAACTTCGGATCTCTCCTGGGATTGTGTCTAATTACCCAAATCCTAACAGGATTATTCTTAGCTATGCATTACACCTCTGATATCTCAACCGCATTCTCATCAGTAACCCACATCTGTCGAGATGTCAACTACGGCTGACTTATCCGAAGCCTACATGCCAACGGAGCATCCTTTTTCTTCATCTGTCTTTATATACATATCGCACGGGGCCTATACTATGGATCATACCTTTATAAAGAAACCTGAAACATTGGTGTAGTCCTGTTTCTTCTGGTCATGATAACAGCCTTTGTCGGCTACGTTCTTCCATGGGGACAAATATCCTTTTGAGGTGCTACTGTGATTACAAACCTCCTATCTGCGGTCCCCTACATGGGAGATACTCTCGTTCAATGAATCTGAGGCGGATTCTCAGTAGACAATGCAACTCTCACACGGTTCTTCGCATTCCACTTCCTACTGCCGTTTGTCGTTACCGGCGCAACCATCCTACACTTATTATTTTTACACGAAACGGGCTCAAACAACCCGGCCGGACTAAATTCCGACGCAGACAAAATTTCTTTCCACCCATATTTCTCATATAAAGACCTTCTCGGCTTTGTGGCTATGTTACTAGCTCTTACCTCCCTAACACTATTTTCCCCTAACCTACTAGGTGACCCGGAAAACTTTACACCAGCAAACCCACTCGTGACACCACCACATATCCAGCCAGAATGATACTTCTTATTTGCCTACGCCATCCTTCGGTCTATTCCAAATAAACTAGGGGGAGTTCTTGCACTACTATTCAGCATTCTAGTGCTATTAGTCGTGCCAACTTTACACACATCCAAACAACGAGGACTAACTTTCCGCCCAGTCACCCAGTTCCTATTCTGAACCCTAGTTGCAGACATATTTATTTTGACATGAATTGGGGGTATACCCGTAGAACACCCATATATTATTATTGGCCAAGTCGCATCCATCCTATACTTTGCACTATTCCTCGTTCTTGTCCCACTAGCAGGATGGGTGGAAAATAAAGCATTGAAATGAGCCAY568617Pseudochondrostomahwillkommi    atggcaagcctacgaaaaactcacccactaataaaaatcgctaacgacgcgctagtcgacctcccaacaccatctaatatctcagtaatatgaaacttcggatctctcctaggattgtgtttgattacccaaatcctaacaggattattcttagctatgcattacacctctgatatctcaaccgcattttcatcagtaacccacatctgtcgagatgtcaactacggctgacttatccgaagcctacatgccaacggagcatcctttttcttcatctgtctttatatacatatcgcacggggcctatactatggatcatacctttataaagaaacctgaaacattggtgtagtcctgtttcttctggtcatgataacagcctttgtcggctacgttcttccatggggacaaatatccttttgaggtgctactgtgattacaaacctcctatctgcagtcccctacatgggagatactctcgttcaatgaatctgaggcggattctcagtagacaatgcaactctcacacggttcttcgcattccacttcctactgccatttgtcgttaccggcgcaaccatcctacacttattatttttacacgaaacgggctcaaacaacccggccggactaaattccgacgcagacagaatttctttccacccatatttctcatataaagaccttctcggctttgtggctatattactagcccttacctccctaacactattttcccctaacctactaggtgacccggaaaactttacaccagcaaacccactcgtgacacccccacatatccagccagaatgatacttcttatttgcctacgccatccttcggtctattccaaataaactagggggagttcttgcactactattcagcattctagtgctattagtcgtgccaattttacacacgtccaaacaacgaggactaactttccgcccagtcacccagttcctattctgaaccctagttgcagacatatttattttgacatgaattgggggtatacccgtagaacacccatatattattattggccaagtcgcatccatcctatactttgcactattcctcgttcttgtcccactagcaggatgggtggaaaataaagcattgaaatgagccHM56027Pseudophoxinusalii               ATGGCAAGCCTACGAAAAACCCACCCACTAATAAAAATCGCCAATGACGCACTAGTCGACCTCCCAACACCATCTAATATCTCAGCAATATGAAACTTCGGTTCTCTCCTGGGATTATGTTTAATTACCCAAATCCTAACAGGCCTGTTCTTGGCCATACATTATACCTCTGATATCTCAACCGCATTCTCATCGGTAACTCATATCTGCCGAGATGTTAACTACGGCTGACTTATCCGAAGCTTACACGCTAACGGGGCATCATTTTTCTTCATCTGCCTCTACATACACATCGCACGAGGCCTATATTATGGATCATACCTTTACAAAGAAACCTGAAACATTGGTGTAGTACTATTCCTTTTAGTTATAATGACAGCCTTCGTCGGCTACGTTCTTCCATGGGGACAAATATCCTTTTGAGGTGCTACCGTAATTACAAATCTCCTCTCAGCGGTCCCTTACATAGGGGACACCCTCGTTCAATGAATCTGAGGCGGTTTCTCAGTAGATAATGCAACCCTCACACGATTCTTCGCATTCCATTTCCTCCTGCCATTTGTTGTAACCGGCGCAACCGTTCTACACTTGTTATTCTTGCACGAGACGGGATCGAACAACCCAGCCGGACTAAATTCCGACGCGGACAAAATTTCTTTCCACCCGTACTTCTCATATAAAGACCTTCTTGGCTTTGTGGCCATATTACTAGCCCTTACTTCACTAACACTATTTTCCCCTAACCTACTAGGGGACCCAGAAAACTTTACCCCAGCGAACCCACTCGTAACCCCCCCACATATTCAGCCAGAATGATACTTCTTGTTTGCCTACGCCATCCTCCGATCTATTCCAAATAAACTAGGAGGGGTTCTTGCACTGCTATTCAGCATCCTAGTCCTAATAATTGTGCCGATTTTACACACATCGAAACAACGAGGACTAACTTTCCGCCCAGTAACTCAATTCCTATTCTGAACCTTAGTTGCAGATATATTTATTCTGACATGAATCGGGGGCATACCTGTAGAACACCCTTATATTATTATTGGCCAAATCGCATCCGTCCTATACTTTGCACTCTTCCTCGTCCTTATTCCACTAGCAGGATGAATAGAAAACAAAGCATTGAAATGAGCCHM56028Pseudophoxinusalii               ATGGCAAGCCTACGAAAAACCCACCCACTAATAAAAATCGCCAATGACGCACTAGTCGACCTCCCAACACCATCTAATATCTCAGCAATATGAAACTTCGGTTCTCTCCTGGGATTATGTTTAATTACCCAAATCCTAACAGGCCTGTTCTTGGCCATACATTATACCTCTGATATCTCAACCGCATTCTCATCGGTAACTCATATCTGCCGAGATGTTAACTACGGCTGACTTATCCGAAGCTTACACGCTAACGGGGCATCATTTTTCTTCATCTGCCTCTACATACACATCGCACGAGGCCTATATTATGGATCATACCTTTACAAAGAAACCTGAAACATTGGTGTAGTACTATTCCTTTTAGTTATAATGACAGCCTTCGTCGGCTACGTTCTTCCATGGGGACAAATATCCTTTTGAGGTGCTACCGTAATTACAAATCTCCTCTCAGCGGTCCCTTACATAGGGGACACCCTCGTTCAATGAATCTGAGGCGGTTTCTCAGTAGATAATGCAACCCTCACACGATTCTTCGCATTCCATTTCCTCCTGCCATTTGTTGTAACCGGCGCAACCGTTCTACACTTGTTATTCTTGCACGAGACGGGATCGAACAACCCAGCCGGACTAAATTCCGACGCGGACAAAATTTCTTTCCACCCGTACTTCTCATATAAAGACCTTCTTGGCTTTGTGGCCATATTACTAGCCCTTACTTCACTAACACTATTTTCCCCTAACCTACTAGGGGACCCAGAAAACTTTACCCCAGCGAACCCACTCGTAACCCCCCCACATATTCAGCCAGAATGATACTTCTTGTTTGCCTACGCCATCCTCCGATCTATTCCAAATAAACTAGGAGGGGTTCTTGCACTGCTATTCAGCATCCTAGTCCTAATAATTGTGCCGATTTTACACACATCGAAACAACGAGGACTAACTTTCCGCCCAGTAACTCAATTCCTATTCTGAACCTTAGTTGCAGATATATTTATTCTGACATGAATCGGGGGCATACCTGTAGAACACCCTTATATTATTATTGGCCAAATCGCATCCGTCCTATACTTTGCACTCTTCCTCGTCCTTATTCCACTAGCAGGATGAATAGAAAACAAAGCATTGAAATGAGCCHM560129Pseudophoxinusanatolicus        ATGGCAAGCCTACGAAAAACCCACCCACTGATAAAAATCGCCAATGACGCACTAGTCGACCTCCCAACACCATCCAATATCTCAGCAATGTGAAACTTCGGTTCTCTCCTGGGATTATGTCTAATTACCCAGATCCTAACAGGATTGTTCTTAGCCATACATTACACCTCTGATATCTCAACCGCATTCTCATCAGTAACCCACATCTGCCGAGACGTTAACTACGGCTGACTCATCCGAAGCTTACATGCTAACGGGGCATCCTTTTTCTTCATCTGCCTCTACATACACATCGCACGAGGCCTATATTATGGGTCATACCTTTATAAGGAGACCTGAAACATTGGAGTAGTCCTATTCCTTTTAGTTATAATAACGGCCTTCGTTGGCTACGTTCTCCCATGAGGACAAATATCCTTTTGAGGTGCTACCGTAATTACAAATCTCCTCTCAGCGGTCCCTTACATAGGAGACACCCTCGTGCAATGAATCTGAGGGGGTTTCTCGGTAGACAACGCGACCCTTACACGATTCTTCGCATTCCACTTCCTCCTGCCATTTGTCGTCACCGGCGCCACCGTCCTCCACTTATTATTCTTGCACGAGACGGGGTCGAACAACCCAGCCGGGTTAAACTCCGACGCAGATAAAATTTCTTTCCACCCATACTTCTCATATAAAGACCTTCTTGGCTTCGTGGCCATATTACTAGCCCTTACCTCTCTAACATTATTCTCCCCTAACCTACTAGGTGACCCGGAGAACTTTACCCCCGCAAACCCGCTCGTAACGCCCCCACATATTCAGCCAGAGTGGTACTTCTTGTTTGCCTACGCCATTCTCCGATCTATCCCAAATAAGCTAGGAGGGGTTCTTGCACTACTATTCAGCATCCTAGTGCTAATAGTCGTGCCAATTCTACACACATCAAAACAACGAGGACTAACTTTCCGACCAATAACTCAGTTCCTGTTCTGAACCCTGGTTGCAGATATATTTATTTTGACATGAATCGGGGGTATACCTGTAGAACACCCATATATTATTATTGGCCAAGTCGCATCCGTCCTATACTTTGCACTCTTCCTNNNNNNNNNNNNNNNNNNNNNNNNNNNNNNNNNNNNNNNNNNNNNNNNNNNNHM560130Pseudophoxinusanatolicus        ATGGCAAGCCTACGAAAAACCCACCCACTGATAAAAATCGCCAATGACGCACTAGTCGACCTCCCAACACCATCCAATATCTCAGCAATGTGAAACTTCGGTTCTCTCCTGGGATTATGTCTAATTACCCAGATCCTAACAGGATTGTTCTTAGCCATACATTACACCTCTGATATCTCAACCGCATTCTCATCAGTAACCCACATCTGCCGAGACGTTAACTACGGCTGACTCATCCGAAGCTTACATGCTAACGGGGCATCCTTTTTCTTCATCTGCCTCTACATACACATCGCACGAGGCCTATATTATGGGTCATACCTTTATAAGGAGACCTGAAACATTGGAGTAGTCCTATTCCTTTTAGTTATAATAACGGCCTTCGTTGGCTACGTTCTCCCATGAGGACAAATATCCTTTTGAGGTGCTACCGTAATTACAAATCTCCTCTCAGCGGTCCCTTACATAGGAGACACCCTCGTGCAATGAATCTGAGGAGGTTTCTCGGTAGACAACGCGACCCTTACACGATTCTTCGCATTCCACTTCCTCCTGCCATTTGTCGTCACCGGCGCCACCGTCCTCCACTTATTATTCTTGCACGAGACGGGGTCGAACAACCCAGCCGGGTTAAACTCCGACGCAGATAAAATTTCTTTCCACCCATACTTCTCATATAAAGACCTTCTTGGCTTCGTGGCCATATTACTAGCCCTTACCTCTCTAACATTATTCTCCCCTAACCTACTAGGTGACCCGGAGAACTTTACCCCCGCAAACCCGCTCGTAACGCCCCCACATATTCAGCCAGAGTGGTACTTCTTGTTTGCCTACGCCATTCTCCGATCTATCCCAAATAAGCTAGGAGGGGTTCTTGCACTACTATTCAGCATCCTAGTGCTAATAGTCGTGCCAATTCTACACACATCAAAACAACGAGGACTAACTTTCCGACCAATAACTCAGTTCCTGTTCTGAACCCTGGTTGCAGATATATTTATTTTGACATGAATTGGGGGTATACCTGTAGAACACCCATATATTATTATTGGCCAAGTCGCATCCGTCCTATACTTTGCACTCTTCCTNNNNNNNNNNNNNNNNNNNNNNNNNNNNNNNNNNNNNNNNNNNNNNNNNNNNHM560131Pseudophoxinusantalyae          ATGGCAAGCCTACGGAAAACCCACCCACTAATAAAAATCGCCAATGACGCACTAGTCGACCTCCCAACACCATCTAATATCTCAGCAATGTGAAACTTCGGTTCTCTCCTGGGATTATGTTTAATTACCCAAATCCTAACAGGCCTGTTCTTGGCCATACATTATACCTCTGATATCTCAACCGCATTCTCATCGGTAACTCATATCTGCCGAGATGTTAACTACGGCTGACTTATCCGAAGCTTACACGCTAACGGGGCATCATTTTTCTTCATCTGCCTCTACATACACATCGCACGAGGCCTATATTATGGATCATACCTTTACAAAGAAACCTGAAACATTGGTGTAGTCCTATTCCTTTTAGTTATAATGACAGCCTTCGTCGGCTACGTTCTTCCATGAGGACAAATATCCTTTTGAGGTGCTACCGTAATTACAAATCTCCTCTCAGCGGTCCCTTACATAGGGGACACCCTCGTTCAATGAATCTGAGGCGGTTTCTCAGTAGATAATGCAACCCTCACACGATTCTTCGCATTCCATTTCCTCCTGCCATTTGTTGTAACCGGCGCAACCGTTCTACACTTGTTATTCTTGCACGAAACGGGGTCGAACAACCCAGCCGGACTAAATTCCGACGCGGACAAAATTTCTTTCCACCCGTACTTCTCATATAAAGACCTTCTTGGCTTTGTGGCCATATTACTAGCCCTTACTTCACTAACACTATTTTCCCCTAACCTACTAGGGGACCCAGAAAACTTTACCCCAGCGAACCCACTCGTAACTCCCCCACATATTCAGCCAGAATGATACTTCTTATTTGCCTACGCCATCCTCCGATCTATTCCAAATAGACTAGGGGGGGTTCTTGCACTGCTATTCAGCATCCTAGTCCTAATAATTGTGCCGATTTTACACACATCGAAACAACGAGGACTAACTTTCCGCCCAGTAACTCAATTCCTATTCTGAACCTTAGTTGCAGATATGTTTATTCTGACATGAATCGGGGGCATACCTGTAGAACACCCTTATATTATTATTGGCCAAATCGCATCCGTCCTATACTTTGCACTCTTCCTCATTCTTATCCCACTAGCAGGATGAATAGAAAACAAAGCATTGAAATGAGCCHM560132Pseudophoxinusantalyae          ATGGCAAGCCTACGAAAAACCCACCCACTAATAAAAATCGCCAATGACGCACTAGTCGACCTCCCAACACCATCTAATATCTCAGCAATGTGAAACTTCGGTTCTCTCCTGGGATTATGTTTAATTACCCAAATCCTAACAGGCCTGTTCTTGGCCATACATTATACCTCTGATATCTCAACCGCATTCTCATCGGTAACTCATATCTGCCGAGATGTTAACTACGGCTGACTTATCCGAAGCTTACACGCTAACGGGGCATCATTTTTCTTCATCTGCCTCTACATACACATCGCACGAGGCCTATATTATGGATCATACCTTTACAAAGAAACCTGAAACATTGGTGTAGTCCTATTCCTTTTAGTTATAATGACAGCCTTCGTCGGCTACGTTCTTCCATGAGGACAAATATCCTTTTGAGGTGCTACCGTAATTACAAATCTCCTCTCAGCGGTCCCTTACATAGGGGACCCCCTCGTTCAATGAATCTGAGGCGGTTTCTCAGTAGATAATGCAACCCTCACACGATTCTTCGCATTCCATTTCCTCCTGCCATTTGTTGTAACCGGCGCAACCGTTCTACACTTGTTATTCTTGCACGAAACGGGGTCGAACAACCCAGCCGGACTAAATTCCGACGCGGACAAAATTTCTTTCCACCCGTACTTCTCATATAAAGACCTTCTTGGCTTTGTGGCCATATTACTAGCCCTTACTTCACTAACACTATTTTCCCCTAACCTACTAGGGGACCCAGAAAACTTTACCCCAGCGAACCCACTCGTAACTCCCCCACATATTCAGCCAGAATGATACTTCTTATTTGCCTACGCCATCCTCCGATCTATTCCAAATAAACTAGGAGGGGTTCTTGCACTGCTATTCAGCATCCTAGTCCTAATAATTGTGCCGATTTTACACACATCGAAACAACGAGGACTAACTTTCCGCCCGGTAACTCAATTCCTATTCTGAACCTTAGTTGCAGATATGTTTATTCTGACATGAATCGGGGGCATACCTGTAGAACACCCTTATATTATTATTGGCCAAATCGCATCCGTCCTATACTTTGCACTCTTCCTCATTCTTGTCCCACTAGCAGGATGAATAGAAAACAAAGCATTGAAATGAGCCAY494748Pseudophoxinusbattalgilae       atggcaagcctacgaaaaacccacccgctcataaaaatcgctaacggagcattagttgacctcccgacgccctccaatatctcagcaatgtgaaacttcggttctctactgggattatgtttaattactcaratcctaacaggattattcttagccatacattacacctctgatatctcgaccgcattctcatcagtaacccacatctgtcgagacgtcaactacggctgactcatccgtagcttacatgctaatggtgcctcctttttcttcatctgcctttacatacacatcgcccgaggcctgtattacggatcatacctttataaagaaacctgaaacattggcgtagtcctgttccttctagtcataataacggcctttgtcggctacgtccttccatgaggacaaatgtccttttgaggtgctacagtaattacaaatctcctctcagcagtcccctacatgggtgacacccttgttcagtgaatctgaggaggtttctcagtagacaatgcaactctcacacgattcttcgcattccatttcctcctaccattcgtcgtcgccggcgcaactattctccatctgttgttcctccacgaaacaggctcaaacaaccctgctggcctaaattccgacgcagacaaaatttctttccatccatacttctcatataaagaccttcttggctttgtggccatgttactagcccttacctctctaacattgttctccccgaaccttctgggtgacccggaaaactttacccctgcaaaccccctcgtgactcctccacacatccaaccagagtgatacttcttgtttgcctacgccatcctccgatccattccaaataaactgggaggggttcttgcactactattcagcatcctagtactaatagtcgtgccgattttacacacatcaaaacaacgaggactaactttccgcccagtractcagtttctattctgaaccttagttgcagatatatttattttgacatggatcgggggcatacccgtggaacatccatacatcattattggtcaagtcgcatccattctatactttgcactcttcctcattcttgtcccgctagcaggatgagtggaaaacaaagcattgaaatgagccHM560133Pseudophoxinuscallensis         ATGGCAAGCCTACGGAAAACCCACCCACTAATAAAAATCGCTAATGACGCACTAGTTGACCTCCCAACACCATCGAACATTTCAGCGCTATGGAACTTCGGGTCCCTTCTGGGACTATGTTTAATTACCCAAATCTTAACAGGACTGTTCCTAGCTATGCACTACACCTCTGATATCTCAACCGCATTTTCATCAGTGACACACATTTGTCGAGATGTTAATTACGGCTGACTAATTCGAAGTCTACATGCCAACGGAGCATCCTTCTTCTTCATCTGCCTTTATATGCACATTGCACGAGGCCTATACTATGGATCCTATCTTTACAAAGAAACTTGAAATATCGGCGTAGTACTATTTCTTTTAGTTATGATAACAGCCTTCGTCGGTTATGTACTCCCATGAGGGCAAATATCCTTTTGAGGCGCCACCGTTATTACGAATCTCCTCTCAGCAGTTCCTTACATAGGAGACACCCTTGTCCAATGAATCTGAGGTGGATTCTCAGTAGACAACGCAACCCTCACACGATTCTTCGCATTCCACTTCCTCCTGCCATTTGTCGTAGCGGGCGCAACTATCCTACACCTGCTATTCCTACACGAGACAGGATCGAACAACCCAGCCGGATTGAATTCCGACGCGGACAAGATTTCCTTTCACCCGTATTTTTCATATAAAGACCTTCTTGGCTTCGTAATTATACTACTAGCCCTCACATCTTTAGCATTGTTTTCTCCCAACCTATTGGGTGACCCTGAAAATTTTACTCCAGCGAACCCACTTGTAACACCCCCACATATCCAGCCAGAGTGATACTTCCTGTTTGCCTACGCCATTCTTCGGTCCATCCCAAATAAACTAGGAGGTGTTCTTGCACTACTATTTAGCATTCTTGTACTAATAGTTGTGCCCATTTTACATACTTCAAAACAACGAGGACTAACTTTCCGCCCTATGACTCAATTCTTATTCTGAACCCTAGTTGCAGACATAATTATCTTGACATGAATTGGGGGTATACCCGTAGAACATCCATACATCATTATTGGCCAAATCGCATCCATTTTATACTTTGCACTATTTCTTATTCTTATCCCACTAGCAGGACTAATGGAAAACAAGGCACTGAAATGAGCCHM560134Pseudophoxinuscallensis         ATGGCAAGCCTACGGAAAACCCACCCACTAATAAAAATCGCTAATAACGCACTAGTTGACCTCCCAACACCATCAAACATTTCAGCGCTATGGAACTTCGGGTCCCTTCTGGGACTATGTTTAATTACCCAAATCTTAACAGGACTGTTCCTAGCTATGCACTACACCTCTGATATCTCAACCGCATTTTCATCAGTGACACACATTTGTCGAGATGTTAATTACGGCTGACTAATTCGAAGTCTACATGCCAACGGAGCATCCTTCTTCTTCATCTGCCTTTATATGCACATTGCACGAGGCCTATACTATGGATCCTATCTTTACAAAGAAACTTGAAATATCGGCGTAGTACTATTTCTTTTAGTTATGATAACAGCCTTCGTCGGTTATGTACTCCCATGAGGGCAAATATCCTTTTGAGGCGCCACCGTTATTACGAATCTCCTCTCAGCAGTTCCTTACATAGGAGACACCCTTGTCCAATGAATCTGAGGTGGATTCTCAGTAGACAACGCAACCCTCACACGATTCTTCGCATTCCACTTCCTCCTGCCATTTGTCGTAGCGGGCGCAACTATCCTACACCTGCTATTCCTACACGAGACAGGATCGAACAACCCGGCCGGATTGAATTCCGACGCGGACAAGATTTCCTTTCACCCGTATTTTTCATATAAAGACCTTCTTGGCTTCGTAATTATACTACTAGCCCTCACATCTTTAGCATTATTTTCTCCCAACCTATTGGGTGACCCTGAAAATTTTACTCCAGCGAACCCACTTGTAACACCCCCACATATCCAGCCAGAGTGATACTTCCTGTTTGCCTACGCCATTCTTCGGTCcATCCCAAATAAACTAGGAGGTGTTCTTGCACTACTATTTAGCATTCTTGTACTAATAGTTGTGCCCATTTTACATACTTCAAAACAACGAGGACTAACTTTCCGCCCTATGACTCAATTCTTGTTCTGAACCCTAGTTGCAGACATAATTATCTTGACATGAATTGGGGGTATACCCGTAGAACACCCATACATCATTATTGGCCAAATCGCATCCATTTTATACTTTGCACTATTTCTTATTCTTATCCCACTAGCAGGATTAATGGAAAACAAGGCACTGAAATGAGCCHM560135Pseudophoxinuschaignoni         ATGGCAAGCCTACGAAAAACCCACCCACTAATAAAAATCGCTAATGACGCACTGGTTGATCTCCCAACACCATCTAACATTTCAGCGCTATGAAACTTCGGATCCCTTCTGGGATTATGTTTAATTACCCAAATCCTAACAGGATTGTTCCTGGCCATGCACTACACCTCTGACATCTCAACCGCATTTTCATCAGTTACACACATCTGCCGGGACGTCAACTATGGCTGACTAATCCGAAGTCTGCATGCCAACGGAGCATCCTTCTTCTTCATCTGCCTTTACATGCATATTGCACGAGGCCTGTATTATGGATCATATCTTTATAAAGAAACCTGAAATATTGGCGTGGTACTATTTCTTTTGGTTATAATAACAGCCTTTGTCGGCTATGTACTTCCGTGGGGACAAATATCCTTTTGAGGCGCCACCGTAATTACAAATCTTCTCTCAGCAGTTCCCTACATAGGAGACACCCTTGTCCAATGAATCTGAGGCGGATTCTCGGTGGACAACGCAACCCTCACGCGATTCTTCGCATTCCACTTCCTCCTACCATTCGTCGTGGCAGGCGCAACCGTACTCCACCTTCTATTCCTACACGAAACGGGGTCAAACAACCCAGCCGGGCTAAACTCCGACGCAGACAAAATCTCCTTCCATCCGTATTTTTCTTACAAAGACCTTCTTGGCTTCGTAGTTATATTACTAGCCCTTACATCTCTAGCATTATTTTCTCCTAACCTATTGGGTGACCCTGAAAATTTTACTCCGGCAAACCCACTTGTGACACCCCCACATATCCAGCCAGAATGATACTTCCTATTTGCCTATGCCATTCTTCGGTCCATTCCAAACAAACTGGGAGGTGTTCTTGCACTACTATTCAGCATCCTCGTACTAATAGTTGTGCCTATTCTACACACTTCAAAGCAACGAGGACTAACTTTCCGACCTATGACTCAATTCCTTTTCTGAACCCTAGTTGCAGATATAATTATCTTAACATGGATTGGAGGCATACCCGTAGAACACCCATATATCATTATTGGCCAAATCGCATCCATCTTATACTTTGCTCTCTTCCTTATCCTTATTCCACTAGCAGGGTTAGTAGAGAACAAGGCATTGAAATGAGCCHM560136Pseudophoxinuschaignoni         ATGGCAAGCCTACGAAAGACCCACCCACTAATAAAAATCGCTAATGACGCACTAGTTGATCTCCCAACACCATCTAACATTTCAGCGCTATGAAACTTCGGATCCCTTCTAGGATTATGTTTAATTACCCAAATCCTAACAGGATTGTTTCTGGCCATGCACTACACCTCTGACATCTCAACCGCATTTTCATCAGTTACACACATCTGCCAGGACGTCAACTACGGCTGACTAATCCGAAGCCTGCATGCCAACGGAGCATCCTTCTTCTTCATCTGCCTTTATATGCATATTGCACGAGGCCTGTACTATGGATCATACCTTTATAAAGAAACCTGAAATATTGGCGTAGTACTATTTCTTTTGGTTATAATAACGGCCTTTGTCGGCTATGTACTTCCATGGGGACAAATATCCTTTTGAGGCGCCACCGTAATTACGAATCTTCTCTCAGCAGTTCCCTACATGGGGGACACCCTTGTCCAATGAATCTGAGGCGGATTCTCGGTAGAAAACGCAACCCTTACGCGATTCTTCGCATTCCACTTCCTCTTACCATTCGTCGTGGCAGGCGCGACCGTACTCCACCTTCTATTCCTACACGAAACGGGGTCAAACAACCCGGCCGGGCTAAACTCCGACGCAGACAAAATCTCCTTCCATCCGTATTTTTCTTACAAAGACCTTCTTGGCTTCGTAGTTATATTACTAGCCCTTACATCTCTAGCATTATTTTCTCCTAACCTATTGGGTGACCCTGAAAATTTTACTCCGGCAAACCCACTTGTGACACCCCCACATATCCAGCCAGAATGATACTTCCTATTTGCCTATGCCATTCTTCGGTCCATTCCAAATAAGCTGGGAGGTGTTCTTGCACTACTATTCAGCATCCTCGTACTAATAATTGTGCCTATTCTACACACTTCAAAACAACGAGGACTAACTTTCCGACCTATGACTCAATTCCTTTTCTGAACCTTAGTTGCGGATATAATTATCCTAACATGAATTGGGGGCATACCCGTAGAGCACCCATATATCATTATTGGCCAAGTCGCATCCATCTTATACTTTGCTCTCTTCCTTATCCTTATCCCACTAGCAGGGTTAGTAGAGAACAAGGCATCGAAATGAGCCHM560147Pseudophoxinuscfkervillei       ATGGCAACCCTACGGAAAACCCACCCCCTCATGAAAATCGCCAACGACGCGCTAGTCGACCTCCCAACCCCATCTAATATCTCAGCAATATGAAACTTCGGCTCTCTCCTAGGCCTATGCTTAATTACCCAAATCCTGACAGGATTATTTTTAGCCATGCACTACACCTCTGACATCTCAACCGCATTCTCATCAGTAACGCACATCTGCCGAGATGTCAACTACGGCTGACTTATCCGAAGTATACATGCTAACGGCGCATCCTTTTTTTTCATCTGCCTTTATATGCACATCGCACGGGGCCTATATTATGGCTCATATCTCTATAAGGAGACCTGAAATATTGGCGTGGTCTTATTTCTACTGGTCATGATGACCGCCTTCGTTGGCTACGTTCTTCCATGAGGACAAATATCCTTTTGAGGTGCAACCGTAATTACAAATCTGCTTTCCGCGGTCCCTTACATGGGGGACGCCCTCGTCCAATGAATCTGAGGCGGCTTCTCGGTAGACAATGCAACACTCACACGATTCTTTGCGTTCCACTTCCTCCTGCCATTTGTCGTCATCGGCGCCACTATTTTACATTTGTTGTTCTTGCATGAAACGGGATCAAATAACCCCGCCGGACTAAATTCCAACGCAGACAAAATTTCTTTTCACCCATACTTCTCATATAAGGACCTCCTTGGTTTTGTAGTTATATTGCTAGCCCTCGCCTCTCTAACTCTGTTCTCCCCTAACCTCTTAGGCGACCCAGAAAACTTTACCCCTGCTAACCCGCTCGTAACTCCCCCACATATCCAACCCGAATGATATTTCCTGTTTGCCTACGCCATCCTTCGGTCTATCCCAAATAAACTGGGAGGCGTTCTTGCATTACTATTCAGCATCCTGGTGCTAATAATCGTGCCAATCTTACACACATCAAAACAACGTGGACTAACTTTCCGCCCGGTGACCCAATTCCTGTTCTGAACCCTGGTTGCAGATATATTTATTCTAACGTGAATTGGGGGTATACCAGTGGAACATCCATATATCATCATTGGCCAAGTCGCATCCGTCCTGTATTTTGCACTCTTCCTCATTCTTACCCCCCTAGCGGGATGGCTGGAAAATAAAGCATTGAAATGAGCCHM560148Pseudophoxinuscfkervillei       ATGGCAACCCTACGGAAAACCCACCCCCTCATGAAAATCGCCAACGACGCGCTAGTCGACCTCCCAACCCCATCTAATATCTCAGCAATATGAAACTTCGGCTCTCTCCTAGGCCTATGCTTAATTACCCAAATCCTGACAGGATTATTTTTAGCCATGCACTACACCTCTGACATCTCAACCGCATTCTCATCAGTAACGCACATCTGCCGAGATGTCAACTACGGCTGACTTATCCGAAGTATACATGCTAACGGCGCATCCTTTTTTTTCATCTGCCTTTATATGCACATCGCACGGGGCCTATATTATGGCTCATATCTCTATAAGGAGACCTGAAATATTGGCGTGGTCTTATTTCTACTGGTCATGATGACCGCCTTCGTTGGCTACGTTCTTCCATGAGGACAAATATCCTTTTGAGGTGCAACCGTAATTACAAATCTGCTTTCCGCGGTCCCTTACATGGGGGACGCCCTCGTCCAATGAATCTGAGGCGGCTTCTCGGTAGACAATGCAACACTCACACGATTCTTTGCGTTCCACTTCCTCCTGCCATTTGTCGTCATCGGCGCCACTATTTTACATTTGTTGTTCTTGCATGAAACGGGATCAAATAACCCCGCCGGACTAAATTCCAACGCAGACAAAATTTCTTTTCACCCATACTTCTCATATAAGGACCTCCTTGGTTTTGTAGTTATATTGCTAGCCCTCGCCTCTCTAACTCTGTTCTCCCCTAACCTCTTAGGCGACCCAGAAAACTTTACCCCTGCTAACCCGCTCGTAACTCCCCCACATATCCAACCCGAATGATATTTCCTGTTTGCCTACGCCATCCTTCGGTCTATCCCAAATAAACTGGGAGGCGTTCTTGCATTACTATTCAGCATCCTGGTGCTAATAATCGTGCCAATCTTACACACATCAAAACAACGTGGACTAACTTTCCGCCCGGTGACCCAATTCCTGTTCTGAACCCTGGTTGCAGATATATTTATTCTAACGTGAATTGGGGGTATACCAGTGGAACATCCATATATCATCATTGGCCAAGTCGCATCCGTCCTGTATTTTGCACTCTTCCTCATTCTTACCCCCCTAGCGGGATGGCTGGAAAATAAAGCATTGAAATGAGCCHM560137Pseudophoxinuscrassus           ATGGCAAGCCTACGAAAAACCCACCCACTGATAAAAATCGCCAATGACGCACTAGTCGACCTCCCAACACCATCCAATATCTCAGCAATGTGAAACTTCGGTTCTCTCCTGGGATTATGTTTAATTACCCAGATCCTAACAGGATTGTTCTTGGCCATACATTACACCTCTGATATCTCAACCGCATTCTCATCAGTAACCCACATCTGCCGAGACGTTAACTACGGCTGACTCATCCGAAGCTTACATGCTAACGGGGCATCCTTTTTCTTCATCTGCCTCTACATACACATCGCACGAGGCCTATATTATGGGTCATACCTTTATAAGGAAACCTGAAACATTGGAGTAGTCCTATTCCTTTTAGTTATAATAACGGCCTTCGTTGGCTACGTTCTCCCATGAGGACAAATATCCTTTTGAGGTGCTACCGTAATTACAAATCTCCTCTCAGCGGTCCCTTACATAGGAGACACCCTCGTGCAATGAATCTGAGGGGGTTTCTCGGTAGACAACGCGACCCTTACACGATTCTTCGCATTCCACTTCCTCCTGCCATTTGTCGTCACCGGCGCCACCGTCCTCCACTTATTGTTCTTGCACGAGACGGGGTCGAACAACCCAGCCGGGTTAAACTCCGACGCAGATAAAATTTCTTTCCACCCATACTTCTCATATAAAGACCTTCTTGGCTTCGTGGCCATATTACTAGCCCTTACCTCTCTAACATTATTCTCCCCTAACCTACTAGGTGACCCGGAGAACTTTACCCCCGCAAACCCGCTCGTAACGCCCCCACACATTCAGCCAGAGTGGTACTTCTTGTTTGCCTACGCCATTCTCCGATCTATCCCAAATAAGCTAGGAGGGGTTCTTGCACTACTATTCAGCATCCTAGTGCTAATAGTCGTGCCAATTCTACACACATCAAAACAACGAGGACTAACTTTCCGACCAATAACTCAGTTCCTATTCTGAACCCTGGTTGCAGATATATTTATTTTGACATGAATCGGGGGTATACCTGTAGAACACCCATATATTATTATTGGCCAAGTCGCATCCGTCCTATACTTTGCACTCTTCCTCATTCTTGTCCCGCTAGCAGGATGAATGGAAAACAAAGCATTGAAATGAGCCHM560138Pseudophoxinuscrassus           ATGGCAAGCCTACGAAAAACCCACCCACTGATAAAAATCGCCAATGACGCACTAGTCGACCTCCCAACACCATCCAATATCTCAGCAATGTGAAACTTCGGTTCTCTCCTGGGATTATGTTTAATTACCCAGATCCTAACAGGATTGTTCTTGGCCATACATTACACCTCTGATATCTCAACCGCATTCTCATCAGTAACCCACATCTGCCGAGACGTTAACTACGGCTGACTCATCCGAAGCTTACATGCTAACGGGGCATCCTTTTTCTTCATCTGCCTCTACATACACATCGCACGAGGCCTATATTATGGGTCATACCTTTATAAGGAAACCTGAAACATTGGAGTAGTCCTATTCCTTTTAGTTATAATAACGGCCTTCGTTGGCTACGTTCTCCCATGAGGACAAATATCCTTTTGAGGTGCTACCGTAATTACAAATCTCCTCTCAGCGGTCCCTTACATAGGAGACACCCTCGTGCAATGAATCTGAGGGGGTTTCTCGGTAGACAACGCGACCCTTACACGATTCTTCGCATTCCACTTCCTCCTGCCATTTGTCGTCACCGGCGCCACCGTCCTCCACTTATTGTTCTTGCACGAGACGGGGTCGAACAACCCAGCCGGGTTAAACTCCGACGCAGATAAAATTTCTTTCCACCCATACTTCTCATATAAAGACCTTCTTGGCTTCGTGGCCATATTACTAGCCCTTACCTCTCTAACATTATTCTCCCCTAACCTACTAGGTGACCCGGAGAACTTTACCCCCGCAAACCCGCTCGTAACGCCCCCACACATTCAGCCAGAGTGGTACTTCTTGTTTGCCTACGCCATTCTCCGATCTATCCCAAATAAGCTAGGAGGGGTTCTTGCACTACTATTCAGCATCCTAGTGCTAATAGTCGTGCCAATTCTACACACATCAAAACAACGAGGACTAACTTTCCGACCAATAACTCAGTTCCTATTCTGAACCCTGGTTGCAGATATATTTATTTTGACATGAATCGGGGGTATACCTGTAGAACACCCATATATTATTATTGGCCAAGTCGCATCCGTCCTATACTTTGCACTCTTCCTCATTCTTGTCCCGCTAGCAGGATGAATGGAAAACAAAGCATTGAAATGAGCCHM5600139Pseudophoxinusegridiri         ATGGCAAGCCTACGAAAAACCCACCCACTAATAAAAATCGCCAATGATGCACTAGTTGACCTTCCAACTCCATCTAATATTTCCGCACTATGAAACTTTGGATCCCTCCTAGGATTATGTTTAATTACCCAAATTCTCACAGGATTATTTCTAGCTATACACTACACCTCTGACATCTCAACTGCATTCTCATCTGTAACACACATCTGCCGAGATGTGAACTACGGCTGACTTATCCGAAGCCTACATGCTAACGGCGCATCATTCTTCTTTATCTGTATTTACATACATATTGCGCGAGGCCTATATTACGGGTCATATCTCTATAAAGAGACTTGAAACATCGGTGTAGTCCTGCTTCTCTTAGTTATAATAACGGCCTTCGTCGGCTACGTACTACCATGGGGGCAAATATCATTCTGAGGTGCCACCGTAATTACAAATCTCCTCTCGGCAGTTCCTTATATAGGGGACACCCTCGTTCAATGGATTTGGGGCGGCTTCTCAGTAGATAACGCAACCCTTACACGGTTCTTCGCATTTCACTTCCTCTTTCCATTCGTCATTGCCGGCGCAACTATCTTACACCTTCTATTTCTCCACGAAACGGGATCAAACAACCCAGCCGGCTTGAACTCCGACGCCGACAAAATCTCCTTTCACCCATATTTCTCATACAAAGACCTTCTTGGCTTCGTAATTATACTCCTGGCCCTCACCTCGCTAGCGCTATTTTCCCCCAACCTGCTAGGTGACCCAGAAAATTTTACCCCTGCAAACCCCCTCGTGACACCCCCACATATTCAACCAGAATGATATTTCTTATTTGCTTACGCCATCCTCCGATCAATTCCAAATAAACTAGGTGGGGTCCTTGCACTATTATTTAGCATTCTCGTACTAATGGTTGTGCCTATTCTACACACCTCGAAACAACGAGGACTAACCTTCCGTCCAGTAACCCAATTTTTATTCTGAACCCTGGTTGCAGACATACTAATTCTGACTTGGATTGGAGGCATACCCGTAGAACACCCCTACATTATTATTGGGCAAATCGCATCCATTTTATACTTCGCACTCTTCCTTGTTCTTACCCCCCTAGCAGGATGGGTGGAAAATAAAGCATTGAAATGAGCCHM5600140Pseudophoxinusegridiri         ATGGCAAGCCTACGAAAAACCCACCCACTAATAAAAATCGCCAATGATGCACTAGTTGACCTTCCAACTCCATCTAATATTTCCGCACTATGAAACTTTGGATCCCTCCTAGGATTATGTTTAATTACCCAAATTCTCACAGGATTATTTCTAGCTATACACTACACCTCTGACATCTCAACTGCATTCTCATCTGTAACACACATCTGCCGAGATGTGAACTACGGCTGACTTATCCGAAGCCTACATGCTAACGGCGCATCATTCTTCTTTATCTGTATTTACATACATATTGCGCGAGGCCTATATTACGGGTCATATCTCTATAAAGAGACTTGAAACATCGGTGTAGTCCTGCTTCTCTTAGTTATAATAACGGCCTTCGTCGGCTACGTACTACCATGGGGGCAAATATCATTCTGAGGTGCCACCGTAATTACAAATCTCCTCTCGGCAGTTCCTTATATAGGGGACACCCTCGTTCAATGGATTTGGGGCGGCTTCTCAGTAGATAACGCAACCCTTACACGGTTCTTCGCATTTCACTTCCTCTTTCCATTCGTCATTGCCGGCGCAACTATCTTACACCTTCTATTTCTCCACGAAACGGGATCAAACAACCCAGCCGGCTTGAACTCCGACGCCGACAAAATCTCCTTTCACCCATATTTCTCATACAAAGACCTTCTTGGCTTCGTAATTATACTCCTGGCCCTCACCTCGCTAGCGCTATTTTCCCCCAACCTGCTAGGTGACCCAGAAAATTTTACCCCTGCAAACCCCCTCGTGACACCCCCACATATTCAACCAGAATGATATTTCTTATTTGCTTACGCCATCCTCCGATCAATTCCAAATAAACTAGGTGGGGTCCTTGCACTATTATTTAGCATTCTCGTACTAATGGTTGTGCCTATTCTACACACCTCGAAACAACGAGGACTAACCTTCCGTCCAGTAACCCAATTTTTATTCTGAACCCTGGTTGCAGACATACTAATTCTGACTTGGATTGGAGGCATACCCGTAGAACACCCCTACATTATTATTGGGCAAATCGCATCCATTTTATACTTCGCACTCTTCCTTGTTCTTACCCCCCTAGCAGGATGGGTGGAAAATAAAGCATTGAAATGAGCCAY494747Pseudophoxinusaelizavetae       atggcaagcctacgaaaaacccacccacttataaaaatcgctaatggagcactagttgacctcccaacgccctccaatatctcagcaatatgaaactttggttctctcctgggattatgtctaattacccaaatcctaacaggattattcttagccatacattacacctctgacatctcaaccgcattctcatcagtaacccacatctgccgagatgtcaactacggctgacttatccgtagcttacatgctaacggggcctcctttttcttcatctgtctttacatgcacatcgcgcgaggcctgtattatggatcatacctttataaagaaacctgaaacattggcgtagtcttatttcttctagttataataacagccttcgtcggctacgttcttccgtgggggcaaatatccttttgaggcgctaccgtaattacaaacctcctctcagcggtcccttatatgggggacacccttgttcaatgaatctgaggtggattctcagtagacaatgcaaccctcacacggttcttcgcattccacttcctcctaccattcgtcgtcgccggtgcaaccctcctacacttattgtttttacacgagacaggatcgaacaatccagccggactaaattccgacgcagacaagatttctttccacccatacttctcgtataaggaccttcttggctttgtggccatattactagcccttacctctgtagcattattctcccctaacctattaggtgacccggagaattttaccccagcaaaccccctcgtaacacccccacatatccagccagaatgatacttcttatttgcctacgccatcctccgatccatcccgaataagctgggaggagtcctcgcgctactatttagcatcctggtactaatagtcgtgccaattttacacacgtcaaaacaacgaggattaacttttcgcccagtgacgcaattcctattctgaaccttagttgcagacatatttattttgacatgaattggaggtatacccgtagaacacccatatattattattggccaaatcgcatccgtcctatacttcgcactcttccttgtccttgtcccactagcaggatgagtggagaacaaagcattgaaatgagccAY494759Pseudophoxinusevliyae           gtggcaagcctacgaaaaacccacccactaataaaaatcgccaatgatgcactagtcgacctcccaacaccatctaatatctcagcaatatgaaacttcggttcgctcctaggattatgtttaattacccaaatcctaacagggttgttcttggctatacattatacctctgatatctcaaccgcattctcatcagtaactcatatctgccgagatgttaactacggctgacttatccgaagcttacatgccaacggggcatcctttttcttcatctgtctctatatacacatcgcacgaggcctatattatggatcgtacctttataaagaaacctgaaacattggtgtagtactattccttttagttataataacagccttcgtcggctacgttctcccatgaggacaaatatccttttgaggtgctaccgtgattacaaatctcctctcagcggtcccttacataggggacaccctcgttcaatgaatctgaggaggtttctcagtagataatgcaacccttacacgattcttcgcatttcacttccttctgccatttgtcgtagccggcgcaaccattctgcacttattattcttgcacgaaacagggtcaaacaacccagccggattaaactccgacgcagacaaaatttctttccacccgtacttctcatataaagaccttcttggctttgtggccatgttactagcccttacctcgctaacattattctcccctaacctactaggtgacccagaaaactttaccccagcgaacccgctcgtaacaccgcctcatattcagccagaatggtacttcttatttgcctacgccatccttcgatctatcccaaataaactaggaggggttcttgcactactgttcagcatcctagtgctaatgattgtgccaattctacacacatccaaacaacgaggactaactttccgcccaataactcaattcctattctgaaccctggttgcagatatatttattttgacatgaatcggaggcatacctgtagaacacccatatattattattggtcaaatcgcatccgtcctatactttgcactctttctcattcttgtcccattggcaggatgagcggagaacaaagcattaaaatgagccAY494760Pseudophoxinusevliyae           atggcaagcctacgaaaaacccacccactaataaaaatcgccaatgatgcactagtcgacctcccaacaccatctaatatctcagcaatgtgaaacttcggttcgctcctaggattatgtttaattacccaaatcctgacagggttgttcttggccatacattatacctctgatatctcaaccgcattctcatcagtaactcatatctgccgagatgttaactacggctgacttatccgaagcttacatgccaacggggcatcctttttcttcatctgtctctatatacacatcgcacgaggcctatattatggatcgtacctttataaagaaacctgaaacattggtgtagtactattccttttagttataataacagccttcgtcggctacgttctcccatggggacaaatatccttttgaggtgctaccgtgattacaaatctcctctcagcggtcccttacataggggacaccctcgttcagtgaatctgaggaggtttctcagtagataatgcaacccttacacgattcttcgcatttcacttccttctgccatttgtcgtagccggcgcaaccattctgcacttattattcttgcacgaaacagggtcaaacaacccagccggattaaactccgacgcagacaaaatttctttccacccgtacttctcatataaagaccttcttggctttgtggccatgttactagcccttacctcgctaacattattctcccctaacctactaggtgacccagaaaactttaccccagcgaacccgcttgtaacaccccctcatattcagccagaatgatacttcttatttgcctacgccatccttcgatctatcccaaataaactaggaggggttcttgcactactgttcagcatcctagtgctaatgattgtgccaattttacacacatccaaacaacgaggactaactttccgcccaataactcaattcctattctgaaccctggttgcagatatatttattttgacatgaatcggaggcatacctgtagaacacccttatattattattggtcaaatcgcatccgtcctatactttgcactctttctcgttcttgtcccattagcagggtgagcggaaaacaaagcattaaaatgagccAY494766Pseudophoxinusfahrettini        atggcaagcctacgaaaaacccacccactaataaaaatcgccaatggcgcactagtcgacctcccaacaccatctaatatctcagcaatatgaaactttggttcgcttctgggattatgtttaattacccaaatcctgacagggttgttcttagccatacattacacctctgacatctcaaccgcattctcatcagtaacccacatctgccgagacgttaactacggctggctcatccgaagcttacatgctaacggggcatcctttttcttcatctgcctctatatacatatcgcgcgaggcctatattacggatcatacctttataaagagacctgaaacatcggggtagtcctgttccttttagttataatgacggccttcgttggctacgttctcccatgaggacaaatgtccttttgaggtgctaccgtaatcacaaatctcctctcagcggtcccttacataggagacaccctcgttcaatgaatctgagggggtttctcagtagacaacgcaaccctcacacgattcttcgcattccacttcctcctgccatttgtcgtcaccggcgccactgttatccacttattattcttgcacgagacgggatcgaacaacccagccggattaaactccgacgcagataagatttctttccacccatacttctcatataaagaccttcttggctttgtggccatgttactagcccttacctctctaacattgttttcccctaacctactgggtgacccggaaaactttaccccagcaaacccgctcgtaacacccccacatattcagccagaatggtacttcttgtttgcctacgccatcctccggtctattccaaataaactaggaggggttcttgcactactattcagcatcctagtgctaatagtcgtgccgattttacacacatcaaaacaacgaggactaactttccgaccgataactcagttcctattctgaaccttagttgcagatatatttattttaacatgaattgggggcatacctgtagaacacccatacattattattggccaaatcgcatccgtcctatactttgcactcttcctcattcttgtcccactagcaggatgaatggaaaacaaagcattgaaatgagccAY494767Pseudophoxinusfirati            atggcaagcctacgaaaaacccacccactgataaaaatcgctaacgatgcattggtagacctcccaacaccatctaatatctcagcaatatgaaacttcggttctctcctaggattatgcttaattacccaratcctaacaggactattcttagccatacactacacctctgacatctcaaccgcattctcatcagtaactcacatctgccgagatgttaactacggctggctcattcgaaacctacatgctaacggggcatcctttttctttatctgtctttacatacacattgcacgaggcctgtattatggctcatacctttataaagaaacctgaaacattggtgtaattttatttcttctggtcataatgacagcctttgtcggctacgtccttccatgaggacaratgtccttttgaggcgctacggtaattacaaacctcctctcagcagtcccttacatgggagacacccttgtccaatgaatctgagggggcttctcagtagacaatgcaactctcacacggttcttcgcattccacttcctcctaccatttgtcgttgccggcgcaaccgtcctacacttattatttttacacgaaacgggatcaaacaacccagccggactaaattccgacgcggacaagatttctttccacccatacttctcatacaaagacctccttggctttgtggccatattactagcccttacatctctgacattgttctcccctaacctcttaggtgacccggaaaactttaccccggcaaaccccctggtaacccctccacacatccagccagagtgatacttcctgtttgcctacgccatccttcgatccattccaaataaactaggaggggttcttgcgttactgtttagtatcctagtgctaatggtcgtgccaattttacacacgtcaaaacaacggggactaactttccggccggtaacgcaattcctattctgaaccctagtcgcagatatatttatcttgacatgaattgggggaataccagtagaacacccgtacattgttattggccaagtcgcatccctcctatactttgcacttttcctcattcttgtcccactagcaggatgactggaaaataaagcattaaaatgagccHM560141Pseudophoxinuskervillei         AtggCaagCCTaCGAAAAtCCCACCCtCTAATAAAAATcgCTAATGACGCACTAGTCGACCTtCCGACaCCATCTAACATcTCAGCAATaTGAAACTTcGgcccTCTtCTAggattatGtTTAATTACCCAAATCCTaACAGGactaTTCCTAGCcATaCACTACACCTCtGAcATCTCAtcCGCATTcTCATCaGTaacccATATcTGccGAGacgttatCtacGGCTGaCTaaTccgaagCctacAcgCCAtGGGaNCatCCTtcTtcTTaATCtgtctttACATNcaNaTcGcccgaGgNctaTATTAtGGCTCATAtCtTtAtAAGgANNCCTGAAAcATtGGGGTAGtCCTATtTCTCCTGGTtAtgAtaaCaccCTTNNtgggctacgtNctNNcaTGNGGACAAATATCCTTTTGAGGCGCAACCGTAATTACAAACCTGCTTTCCGCGGTCCCTTATATGGGAGACGCCCTCGTCCAATGAATCTGGGGGGGCTTCTCGGTAGACAATGCAACACTCACGCGATTCTTTGCGTTCCACTTCCTCCTACCATTTGTAGTCATCGGCGCCACTATCATACACTTGTTGTTCTTGCACGAGACGGGGTCAAATAACCCGGCCGGACTAAATTCCGACGCAGACAAAATTTCTTTTCACCCATACTTCTCATATAAGGACCTTCTTGGTTTTGTAGCTATATTGCTAGCCCTCGCCTCGCTAACGCTGTTCTCCCCTAACCTCTTAGGGGACCCAGAAAACTTTACCCCTGCTAACCCACTCGTAACACCCCCACATATCCAACCCGAATGATACTTCTTATTTGCCTACGCCATCCTTCGGTCCATCCCAAATAAATTAGGAGGCGTTCTTGCATTACTGTTCAGCATCCTCGCGCTGATAATCGTGCCAATTTTACACACATCAAAACAACGAGGACTAACTTTCCGCCCGGTGACCCAATTCCTGTTCTGAACCCTGGTTGCAGATATATTTATCCTAACATGGATTGGAGGCATGCCGGTAGAACACCCATATATCATCATTGGCCAAGTCGCATCCGTCCTATATTTTGCACTCTTCCTNNNNNNNNNNNNNNNNNNNNNNNNNNNNNNNNNNNNNNNNNNNNNNNNNNNNHM560142Pseudophoxinuskervillei         ATGGCAAGCCTACGAAAATCCCACCCtCTAATAAAAATCGCTAATGACGCACTAGTCGACCTtCCGACACCATCTAACATCTCAGCAATATGAAACTTCGGCCCTCTTCTAGGATTATGTTTAATTACCCAAATCCTAACAGGACTATTCCTAGCCATACACTACACCTCTGACATCTCATCCGCATTCTCATCAGTAACCCATATCTGCCGAGACGTTATCTACGGCTGACTAATCCGAAGCCTACACGCCATGGGANCATCCTTCTTCTTAATCTGTCTTTACATNCANATCGCCCGAGGNCTATATTATGGCTCATATCTTTATAAGGANNCCTGAAACATTGGGGTAGTCCTATTTCTCCTGGTTATGATAACACCCTTNNTGGGCTACGTNCTNNCATGNGGACAAATATCCTTTTGAGGCGCAACCGTAATTACAAACCTGCTTTCCGCGGTCCCTTATATGGGAGACGCCCTCGTCCAATGAATCTGGGGGGGCTTCTCGGTAGACAATGCAACACTCACGCGATTCTTTGCGTTCCACTTCCTCCTACCATTTGTAGTCATCGGCGCCACTATCATACACTTGTTGTTCTTGCACGAGACGGGGTCAAATAACCCGGCCGGACTAAATTCCGACGCAGACAAAATTTCTTTTCACCCATACTTCTCATATAAGGACCTTCTTGGTTTTGTAGCTATATTGCTAGCCCTCGCCTCGCTAACGCTGTTCTCCCCTAACCTCTTAGGGGACCCAGAAAACTTTACCCCTGCTAACCCACTCGTAACACCCCCACATATCCAACCCGAATGATACTTCTTATTTGCCTACGCCATCCTTCGGTCCATCCCAAATAAATTAGGAGGCGTTCTTGCATTACTGTTCAGCATCCTCGCGCTGATAATCGTGCCAATTTTACACACATCAAAACAACGAGGACTAACTTTCCGCCCGGTGACCCAATTCCTGTTCTGAACCCTGGTTGCAGATATATTTATCCTAACATGGATTGGAGGCATGCCGGTAGAACACCCATATATCATCATTGGCCAAGTCGCATCCGTCCTATATTTTGCACTCTTCCTNNNNNNNNNNNNNNNNNNNNNNNNNNNNNNNNNNNNNNNNNNNNNNNNNNNNHM560145Pseudophoxinusninae             ATGGCAAGCCTACGAAAAACCCACCCACTAATAAAAATCGCCAATGATGCACTAGTCGACCTCCCAACACCATCTAATATCTCAGCAATGTGAAACTTCGGTTCTCTCCTGGGATTATGTTTAATTACCCAAATCCTAACAGGGTTATTCTTGGCCATACATTATACCTCTGATATCTCAACCGCATTCTCATCAGTAACTCATATCTGCCGAGATGTTAACTACGGCTGACTTATCCGAAGCTTACATGCTAATGGAGCATCCTTTTTCTTCATCTGCCTCTACATACACATCGCACGAGGCCTATATTATGGATCATACCTTTATAAAGAAACCTGAAACATTGGTGTAATCCTATTCCTGTTAGTTATAATAACGGCCTTCGTCGGCTACGTTCTTCCATGAGGACAAATATCCTTTTGAGGTGCTACAGTAATTACAAATCTCCTCTCAGCGGTCCCTTACATGGGGGACACCCTAGTTCAATGAATCTGAGGGGGTTTCTCAGTAGATAACGCAACCCTCACACGATCCTTCGCATTCCACTTCCTCCTACCATTTGTCGTAGCCGGCGCAACCATTCTGCACTTGTTATTCTTGCACGAAACAGGCTCAAACAACCCAGCCGGACTAAACTCCGACGCAGACAAAATTTCTTTCCACCCATACTTCTCATATAAAGACCTTCTTGGCTTTGTGGCCATGTTACTAGCCCTTACCTCACTAACATTATTCTCCCCTAACCTACTAGGTGACCCAGAAAACTTTACCCCAGCAAACCCGCTAGTAACGCCCCCCCATATTCAGCCTGAATGGTACTTCTTGTTTGCCTACGCCATCCTCCGATCTATTCCAAATAAGCTAGGAGGGGTCCTTGCACTACTATTTAGCATCCTGGTGCTAATAGTTGTGCCAATTTTACACACATCGAAACAACGAGGACTAACTTTCCGTCCATTAACTCAGTTTCTATTCTGAACCCTAGTTGCAGATATGTTTATTTTGACATGAATCGGGGGCATACCTGTAGAACACCCATATATTATTATTGGCCAAATCGCATCCGTCCTATACTTTGCACTCTTCCTCATTCTTGTTCCACTAGCAGGATGAATGGAAAACAAAGCATTAAGATGAGCCHM560146Pseudophoxinusninae             ATGGCAAGCCTACGAAAAACCCACCCACTAATAAAAATCGCCAATGATGCACTAGTCGACCTCCCAACACCATCTAATATCTCAGCAATGTGAAACTTCGGTTCTCTCCTGGGATTATGTTTAATTACCCAAATCCTAACAGGGTTATTCTTGGCCATACATTATACCTCTGATATCTCAACCGCATTCTCATCAGTAACTCATATCTGCCGAGATGTTAACTACGGCTGACTTATCCGAAGCTTACATGCTAATGGAGCATCCTTTTTCTTCATCTGCCTCTACATACACATCGCACGAGGCCTATATTATGGATCATACCTTTATAAAGAAACCTGAAACATTGGTGTAGTCCTATTCCTGTTAGTTATAATAACAGCCTTCGTCGGCTACGTTCTTCCATGAGGACAAATATCCTTTTGAGGTGCTACAGTAATTACAAATCTCCTCTCAGCGGTCCCTTACATGGGGGACACCCTAGTTCAATGAATCTGAGGGGGTTTCTCAGTAGATAACGCAACCCTCACACGATTCTTCGCATTCCACTTCCTCCTGCCATTTGTCGTAGCCGGCGCAACCATTCTGCACTTGTTATTCTTGCACGAAACAGGCTCAAACAACCCAGCCGGACTAAACTCCGACGCAGACAAAATTTCTTTCCACCCATACTTCTCATATAAAGACCTTCTTGGCTTTGTGGCCATGTTACTAGCCCTTACCTCACTAGCATTATTCTCCCCTAACCTACTAGGTGACCCAGAAAACTTTACCCCAGCAAACCCGCTAGTAACGCCCCCCCATATTCAGCCCGAATGGTACTTCTTATTTGCCTACGCCATCCTCCGATCTATTCCAAATAAACTAGGAGGGGTCCTTGCACTACTATTTAGCATCCTGGTGCTAATAGTTGTGCCAATTTTACACACATCGAAACAACGAGGACTAACTTTCCGTCCATTAACTCAGTTTCTATTCTGGACCCTAGTTGCAGATATGTTTATTTTGACATGAATCGGGGGCATACCTGTAGAACACCCATATATTATTATTGGCCAAATCGCATCCGTCCTATACTTTGCACTCTTCCTCATTCTTGTCCCACTAGCAGGATGAATGGAAAACAAAGCATTAAAATGAGCCHM560143Pseudophoxinuspunicus           ATGGCAAGCCTACGAAAAACCCACCCACTGATAAAAATCGCTAATAGCGCACTAGTTGACCTTCCAACACCCTCTAATATCTCAGCAATGTGAAACTTCGGATCTCTCCTAGGATTATGTTTGATTACCCAGATTCTAACAGGATTATTCCTCGCCATACACTACACCTCTGATATTTCCACTGCGTTCTCATCAGTAACCCATATCTGCCGAGACGTCAACTACGGCTGGCTAATTCGCAACCTGCATGCCAACGGAGCATCGTTCTTCTTTATCTGTTTGTATATACATATTGCACGAGGTTTATATTATGGCTCATATCTTTATAAGGAGACCTGAAATATTGGAGTGGTATTGTTTCTTCTCGTTATAATGACGGCTTTCGTGGGCTATGTCCTTCCATGAGGACAAATGTCCTTTTGAGGCGCCACTGTGATTACCAACCTCCTCTCAGCGGTTCCCTACATGGGTGATACCCTTGTTCAATGGATCTGAGGGGGCTTTTCAGTAGATAATGCAACCCTTACACGATTCTTCGCATTCCACTTCCTCTTACCATTCGTCGTTGCAGGCGCGACCCTCCTCCACTTACTATTCCTACACGAAACAGGATCGAACAACCCGGCCGGACTAAACTCTGACGCGGACAAAATTTCTTTTCACCCGTATTTCTCTTACAAAGACCTTCTCGGCTTCGTGATCATATTGCTGGCCCTCACCTCGCTGGCGCTATTTTCCCCCAACCTCCTAGGTGATCCAGAGAATTTTACCCCAGCAAACCCACTTGTAACACCTCCACACATTCAGCCAGAGTGATACTTCTTATTTGCATACGCCATCCTTCGGTCCATTCCTAATAAACTAGGCGGAGTCCTTGCACTATTATTTAGTATTCTAGTGCTAATGGTTGTGCCAATCTTACATACCTCGAAACAACGAGGACTAACTTTCCGCCCTGTAACTCAATTCCTATTCTGAACCCTAGTTGCAGACATGATTATCCTGACATGAATTGGGGGCATACCCGTAGAGCACCCATACATTATTATTGGCCAAGTCGCATCCGTCCTATACTTTGCACTTTTCCTTATTCTCGCCCCACTAGCAGGCTTAGTGGAAAATAAAGCATTAAAATGAGCCHM560144Pseudophoxinuspunicus           ATGGCAAGCCTACGAAAAACCCACCCACTGATAAAAATCGCTAATAGCGCACTAGTTGACCTTCCAACACCCTCTAATATCTCAGCAATGTGAAACTTCGGATCTCTCCTAGGATTATGTTTGATTACCCAGATTCTAACAGGATTATTCCTCGCCATACACTACACCTCTGATATTTCCACTGCGTTCTCATCAGTAACCCATATCTGCCGAGACGTCAACTACGGCTGGCTAATTCGCAACCTGCATGCCAACGGAGCATCGTTCTTCTTTATCTGTTTGTATATACATATTGCACGAGGTTTATATTATGGCTCATATCTTTATAAGGAGACCTGAAATATTGGAGTGGTATTGTTTCTTCTCGTTATAATGACGGCTTTCGTGGGCTATGTCCTTCCATGAGGACAAATGTCCTTTTGAGGCGCCACTGTGATTACCAACCTCCTCTCAGCGGTTCCCTACATGGGTGATACCCTTGTTCAATGGATCTGAGGGGGCTTTTCAGTAGATAATGCAACCCTTACACGATTCTTCGCATTCCACTTCCTCTTACCATTCGTCGTTGCAGGCGCGACCCTCCTCCACTTACTATTCCTACACGAAGCAGGATCGAACAACCCGGCCGGACTAAACTCTGACGCGGACAAAATTTCTTTTCACCCGTATTTCTCTTACAAAGACCTTCTCGGCTTCGTGATCATATTGCTGGCCCTCACCTCGCTGGCGCTATTTTCCCCCAACCTCCTAGGTGATCCAGAGAATTTTACCCCAGCAAACCCACTTGTAACACCTCCACACATTCAGCCAGAATGATACTTCTTATTTGCATACACCATCCTTCGGTCCATTCCTAATAAACTAGGCGGAGTCCTTGCACTATTATTTAGTATTCTAGTGCTAATGGTTGTGCCAATCTTACATACCTCGAAACAACGAGGACTAACTTTCCGCCCTGTAACTCAATTCCTATTCTGAACCCTAGTTGCAGACATGATTATCCTGACATGAATTGGGGGCATACCCGTAGAGCACCCATACATTATTATTGGCCAAGTCGCATCCGTCCTATACTTTGCACTTTTCCTTATTCTCGCCCCACTAGCAGGCTTAGTGGAAAATAAAGCATTAAAATGAGCCHM560149Pseudophoxinussp                ATGGCAAGCCTACGAAAAACCCATCCATTAATAAAAATCGCCAATGGCGCACTAGTCGACCTCCCAACACCATCTAATATCTCAGCAATATGAAACTTCGGTTCGCTTCTGGGATTGTGTTTGATTACCCAAATCCTAACAGGGTTATTCTTGGCCATACATTACACCTCTGACATCTCAACCGCATTCTCATCAGTGACCCATATCTGCCGGGACGTAAACTACGGCTGGCTCATCCGAAGCTTACATGCTAACGGGGCATCCTTTTTCTTCATCTGCCTCTATATGCATATCGCGCGTGGCCTATATTATGGCTCATACCTTTATAAAGAAACCTGAAACATCGGGGTAGTCCTATTCCTTTTAGTTATGATGACGGCCTTCGTTGGCTACGTTCTCCCATGAGGACAAATATCCTTTTGAGGTGCTACCGTAATTACAAATCTCCTCTCAGCGGTCCCTTACATAGGAGACACCCTCGTTCAATGAATCTGAGGGGGTTTCTCAGTAGACAANNNNNNNNNNNNNNNNNNNNNNNNNNNNNNNNNNNNNNNNNNNNNNNNNNNNNNNNNNNNNNNNNNNNNNNNNNNNNNNNNNNNNNNNNNNNNNNNNNNNNNNNNNNNNNNNNNNNNNNNNNNNNNNNNNNNNNNNNNNNNNNNNNNNNNNNNNNNNNNNNNNNNNNNNNNNNNNNNNNNNNNNNNNNNNNNNNNNNNNNNNNNNNNNNNNNNNNNNNNNNNNNNNNNNNNNNNNNNNNNNNNNNNNNNNNNNNNNNNNNNNNNNNNNNNNNNNNNNNNNNNNNNNNNNNNNNNNNNNNNNNNNNNNNNNNNNNNNNNNNNNNNNNNNNNNNNNNNNNNNNNNNNNNNNNNNNNNNNNNNNNNNNNNNNNNNNNNNNNNNNNNNNNNNNNNNNNNNNNNNNNNNNNNNNNNNNNNNNNNNNNNNNNNNNNNNNNNNNNNNNNNNNNNNNNNNNNNNNNNNNNNNNNNNNNNNNNNNNNNNNNNNNNNNNNNNNNNNNNNNNNNNNNNNNNNNNNNNNNNNNNNNNNNNNNNNNNNNNNNNNNNNNNNNNNNNNNNNNNNNNNNNNNNNNNNNNNNNNNNNNNNNNNNNNNNNNNNNNNNNNNNNNNNNNNNNNNNNNNNNNNNNNHM560150PseudophoxinuspEflatum          ATGGCAAGCCTACGAAAAACCCATCCATTAATAAAAATCGCCAATGGCGCACTAGTCGACCTCCCAACACCATCTAATATCTCAGCAATATGAAACTTCGGTTCGCTTCTAGGATTGTGTTTGATTACCCAAATCCTAACAGGGTTATTCTTGGCCATGCATTACACCTCTGACATCTCAACCGCATTCTCATCAGTGACCCATATCTGCCGGGACGTAAACTACGGCTGGCTCATCCGAAGCTTACATGCTAACGGGGCATCCTTTTTCTTCATCTGCCTCTATATGCATATCGCGCGTGGCCTATATTATGGCTCATACCTTTATAAAGAAACCTGAAACATCGGGGTAGTCCTATTCCTTTTAGTTATAATGACGGCCTTCGTTGGCTACGTTCTCCCATGAGGACAAATATCCTTTTGAGGTGCTACCGTAATTACAAATCTCCTCTCAGCGGTCCCTTACATGGGAGACACCCTCGTTCAATGAATCTGAGGGGGTTTCTNNNNNNNNNNNNNNNNNNNNNNNNNNNNNNNNNNNNNNNNNNNNNNNNNNNNNNNNNNNNNNNNNNNNNNNNNNNNNNNNNNNNNNNNNNNNNNNNNNNNNNNNNNNNNNNNNNNNNNNNNNNNNNNNNNNNNNNNNNNNNNNNNNNNNNNNNNNNNNNNNNNNNNNNNNNNNNNNNNNNNNNNNNNNNNNNNNNNNNNNNNNNNNNNNNNNNNNNNNNNNNNNNNNNNNNNNNNNNNNNNNNNNNNNNNNNNNNNNNNNNNNNNNNNNNNNNNNNNNNNNNNNNNNNNNNNNNNNNNNNNNNNNNNNNNNNNNNNNNNNNNNNNNNNNNNNNNNNNNNNNNNNNNNNNNNNNNNNNNNNNNNNNNNNNNNNNNNNNNNNNNNNNNNNNNNNNNNNNNNNNNNNNNNNNNNNNNNNNNNNNNNNNNNNNNNNNNNNNNNNNNNNNNNNNNNNNNNNNNNNNNNNNNNNNNNNNNNNNNNNNNNNNNNNNNNNNNNNNNNNNNNNNNNNNNNNNNNNNNNNNNNNNNNNNNNNNNNNNNNNNNNNNNNNNNNNNNNNNNNNNNNNNNNNNNNNNNNNNNNNNNNNNNNNNNNNNNNNNNNNNNNNNNNNNNNNNNNNNNNNNNNNNNNNNNNNNNNHM560151Pseudophoxinuszeregi            ATGGCAAGCCTACGAAAAACCCATCCACTTATAAAAATCGCTAACGACGCACTAGTCGACCTCCCAACACCATCTAATATTTCAGCAATATGAAACTTTGGCTCCCTCCTAGGATTATGCTTATTTACCCAAATCCTGACGGGATTATTTTTAGCCATACACTACACCTCTGACATCTCCACCGCATTCTCATCGGTAACCCACATTTGCCGAGATGTTAATTATGGCTGACTTATCCGAAGCCTGCATGCTAACGGAGCATCCTTTTTCTTCATCTGCCTTTATATACATATTGCACGAGGCCTATATTATGGCTCATACCTTTACAAAGAAACCTGAAACATTGGTGTAATCTTATTTCTTCTGGTTATAATGACAGCCTTCGTTGGCTACGTTCTTCCATGGGGACAAATATCCTTTTGAGGTGCAACCGTAATTACAAACCTCCTATCAGCAGTCCCTTATATGGGAGACACCCTTGTCCAGTGAATCTGGNNNNNNNNNNNNNNNNNNNNNNNNNNNNNNNNNNNNNNNNNNNNNNNNNNNNNNNNNNNNNNNNNNNNNNNNNNNNNNNNNNNNNNNNNNNNNNNNNNNNNNNNNNNNNNNNNNNNNNNNNNNNNNNNNNNNNNNNNNNNNNNNNNNNNNNNNNNNNNNNNNNNNNNNNNNNNNNNNNNNNNNNNNNNNNNNNNNNNNNNNNNNNNNNNNNNNNNNNNNNNNNNNNNNNNNNNNNNNNNNNNNNNNNNNNNNNNNNNNNNNNNNNNNNNNNNNNNNNNNNNNNNNNNNNNNNNNNNNNNNNNNNNNNNNNNNNNNNNNNNNNNNNNNNNNNNNNNNNNNNNNNNNNNNNNNNNNNNNNNNNNNNNNNNNNNNNNNNNNNNNNNNNNNNNNNNNNNNNNNNNNNNNNNNNNNNNNNNNNNNNNNNNNNNNNNNNNNNNNNNNNNNNNNNNNNNNNNNNNNNNNNNNNNNNNNNNNNNNNNNNNNNNNNNNNNNNNNNNNNNNNNNNNNNNNNNNNNNNNNNNNNNNNNNNNNNNNNNNNNNNNNNNNNNNNNNNNNNNNNNNNNNNNNNNNNNNNNNNNNNNNNNNNNNNNNNNNNNNNNNNNNNNNNNNNNNNNNNNNNNNNNNNNNNNNNNNNNNNNNNNNNNNHM560152Pseudophoxinuszeregi            ATGGCAAGCCTACGAAAAACCCATCCACTTATAAAAATCGCTAACGACGCACTAGTCGACCTCCCAACACCATCTAATATTTCAGCAATATGAAACTTTGGCTCCCTCCTAGGATTATGCTTATTTACCCAAATCCTGACGGGATTATTTTTAGCCATACACTACACCTCTGACATCTCCACCGCATTCTCATCGGTAACCCACATTTGCCGAGATGTTAATTATGGCTGACTTATCCGAAGCCTGCATGCTAACGGAGCATCCTTTTTCTTCATCTGCCTTTATATACATATTGCACGAGGCCTATATTATGGCTCATACCTTTACAAAGAAACCTGAAACATTGGTGTAATCTTATTTCTTCTGGTTATAATGACAGCCTTCGTTGGCTACGTTCTTCCATGGGGACAAATATCCTTTTGAGGTGCAACCGTAATTACAAACCTCCTATCAGCAGTCCCTTATATGGGAGACACCCTTGTCCAGTGAATCTNNNNNNNNNNNNNNNNNNNNNNNNNNNNNNNNNNNNNNNNNNNNNNNNNNNNNNNNNNNNNNNNNNNNNNNNNNNNNNNNNNNNNNNNNNNNNNNNNNNNNNNNNNNNNNNNNNNNNNNNNNNNNNNNNNNNNNNNNNNNNNNNNNNNNNNNNNNNNNNNNNNNNNNNNNNNNNNNNNNNNNNNNNNNNNNNNNNNNNNNNNNNNNNNNNNNNNNNNNNNNNNNNNNNNNNNNNNNNNNNNNNNNNNNNNNNNNNNNNNNNNNNNNNNNNNNNNNNNNNNNNNNNNNNNNNNNNNNNNNNNNNNNNNNNNNNNNNNNNNNNNNNNNNNNNNNNNNNNNNNNNNNNNNNNNNNNNNNNNNNNNNNNNNNNNNNNNNNNNNNNNNNNNNNNNNNNNNNNNNNNNNNNNNNNNNNNNNNNNNNNNNNNNNNNNNNNNNNNNNNNNNNNNNNNNNNNNNNNNNNNNNNNNNNNNNNNNNNNNNNNNNNNNNNNNNNNNNNNNNNNNNNNNNNNNNNNNNNNNNNNNNNNNNNNNNNNNNNNNNNNNNNNNNNNNNNNNNNNNNNNNNNNNNNNNNNNNNNNNNNNNNNNNNNNNNNNNNNNNNNNNNNNNNNNNNNNNNNNNNNNNNNNNNNNNNNNNNNNNNNNNNNNNNHM560153Pseudophoxinuszekayi            ATGGCAAGCCTACGAAAAACCCACCCACTGATAAAAATCGCTAACGACGCATTAGTTGACCTCCCAACACCATCTAATATTTCCGCAATATGAAACTTCGGCTCTCTCCTAGGGTTATGTTTAATTACCCAAATCCTAACAGGATTATTCTTAGCCATACATTATACCTCTGACATCTCAACCGCATTCTCATCAGTAACCCACATCTGCCGAGACGTCAACTACGGCTGACTAATTCGAAGCCTACATGCCAACGGAGCATCCTTTTTCTTCATCTGCCTCTACATGCATATTGCACGAGGCCTGTATTATGGCTCCTACCTTTATAAAGAAACCTGAAACATTGGTGTAGTCTTATTTCTTCTGGTCATAATAACAGCCTTCGTTGGCTACGTCCTTCCATGGGGACAAATGTCCTTTTGAGGTGCTACCGTGATCACAAACCTCCTCTCAGCAGTCCCTTACATAGGGGACACCCTTGTTCAGTGAATCTGAGGGGGTTTCTCAGTAGACAATGCAACTCTCACACGGTTCTTCGCATTCCACTTCCTCCTGCCATTTGTCGTCGCCGGCGCAACCATTCTACACTTGTTATTCTTACACGAAACGGGATCAAACAACCCGGCCGGACTAAATTCCGACGCAGACAAGATTTCTTTCCACCCCTACTTCTCATATAAAGACCTTCTTGGCTTTGTAACCATATTACTAGCACTTACAGCCCTAACGTTGTTCTCCCCTAACCTTCTAGGTGACCCGGAGAACTTTACCCCAGCAAACCCACTCGTAACCCCCCCACATATCCAGCCAGAGTGATACTTCTTGTTTGCCTACGCCATCCTTCGATCTATTCCAAATAAACTAGGAGGGGTTCTTGCGTTACTGTTCAGCATCCTAGTGCTAATGGTTGTGCCAATTTTACACACGTCAAAACAACGAGGACTAACTTTCCGACCGGTAACTCAATTCCTATTCTGAACCCTAGTCGCAGACATATTTATTTTAACATGAATCGGGGGCATACCCGTAGAACACCCATATATTATTATTGGCCAAATCGCATCCGTCCTATACTTTGCACTCTTCCTCGTTCTTGTCCCACTAGCAGGATGACTAGAGAATAAAGCATTGAAATGAGCCHM560154Pseudophoxinuszekayii           ATGGCAAGCCTACGAAAAACCCACCCACTGATAAAAATCGCTAACGACGCATTAGTTGACCTCCCAACACCATCTAATATTTCCGCAATATGAAACTTCGGCTCTCTCCTAGGGTTATGTTTAATTACCCAAATCCTAACAGGATTATTCTTAGCCATACATTATACCTCTGACATCTCAACCGCATTCTCATCAGTAACCCACATCTGCCGAGACGTCAACTACGGCTGACTAATTCGAAGCCTACATGCCAACGGAGCATCCTTTTTCTTCATCTGCCTCTACATACATATTGCACGAGGCCTGTATTATGGCTCCTACCTTTATAAAGAAACCTGAAACATTGGTGTAGTCTTATTTCTTCTGGTCATAATAACAGCCTTCGTTGGCTACGTCCTTCCATGGGGACAAATGTCCTTTTGAGGTGCTACCGTGATCACAAACCTCCTCTCAGCAGTCCCTTACATAGGGGACACCCTTGTTCAGTGAATCTGAGGGGGTTTCTCAGTAGACAATGCAACTCTCACACGGTTCTTCGCATTCCACTTCCTCCTGCCATTTGTCGTCGCCGGCGCAACCATTCTACACTTGTTATTCTTACACGAAACGGGATCAAACAACCCGGCCGGACTAAATTCCGACGCAGACAAGATTTCTTTCCACCCATACTTCTCATATAAAGACCTTCTTGGCTTTGTAACCATATTACTAGCACTTACAGCCCTAACGTTGTTCTCCCCTAACCTTCTAGGTGACCCGGAAAACTTTACCCCAGCAAACCCACTCGTAACCCCCCCACATATCCAGCCAGAGTGATACTTCTTGTTTGCCTACGCCATCCTTCGATCTATTCCAAATAAACTAGGAGGGGTTCTTGCGTTACTGTTCAGCATCCTAGTGCTAATGGTTGTGCCAATTTTACACACGTCAAAACAACGAGGACTAACTTTCCGACCGGTAACTCAATTCCTATTCTGAACCCTAGTCGCAGACATATTTATTTTAACATGAATCGGGGGCATACCCGTAGAACACCCATATATTATTATTGGCCAAATCGCATCCGTCCTATACTTTGCACTCTTCCTCGTTCTTGTCCCACTAGCAGGATGACTAGAGAATAAAGCATTGAAATGAGCCHM560155Pseudorasboraparva              ATGGCAAGCCTACGAAAAACCCACCCACTAATTAAAATCGCTAACGACGCACTAGTTGATCTACCAACCCCCTCTAACATCTCAGTGTGATGAAACTTTGGATCCCTTTTAGGCCTATGTTTAATTGCACAAATCCTAACAGGACTATTCTTAGCCATACACTACACCTCTGACATCTCAACTGCATTTTCATCGGTGGCCCACATCTGTCGAGACGTTAACTACGGTTGATTTATTCGAAATATACACGCCAACGGAGCATCATTCTTTTTCATTTGTATTTATATACACGTTGCCCGAAGTCTATACTATGGCTCTTACTTATATAAAGAGACCTGAAACATCGGTGTGGTTCTCCTTCTTCTAGTAATAATGACAGCCTTCGTTGGCTACGTCCTTCCATGAGGACAAATATCCTTCTGAGGGGCCACAGTAATTACAAACTTATTATCAGCAGTTCCGTACATAGGAGATATATTAGTTCAATGAATTTGAGGGGGTTTTTCAGTAGATAATGCAACACTAACACGATTCTTCGCATTCCACTTCCTTCTCCCATTTATTATTGCTGCCGCAACGGTTATTCATCTTCTATTCCTACACGAAACAGGATCAAACAACCCAGCCGGGCTAAATTCTGACGCAGATAAAATTTCTTTCCACCCATACTTTTCATACAAAGACCTTCTTGGCTTTGTATTAATATTGTTAGCCCTTACATCCCTGGCGCTATTTTCCCCTAACCTATTAGGAGACCCCGACAACTTCACACCCGCCAACCCAATAGTCACTCCCCCACATATTAAGCCTGAATGATACTTTCTATTTGCCTACGCCATCCTACGATCCATCCCAAACAAACTAGGAGGCGTTCTTGCACTATTGTTTTCTATCCTGATCCTAATAGTAGTTCCCATCCTTCACACCTCTAAACAACGAGGACTCACCTTCCGCCCAATGACTCAATTTTTATTCTGAACACTAGTAGCAGATATAATAATCTTAACATGAATTGGAGGAATACCTGTAGAACACCCCTATGTGATTATTGGCCAAATCGCATCCGTCCTATATTTTGCACTTTTCCTAATCCTCATCCCACTAGCAGGATGAATGGAAAGTAAAGCGTTAAAATGAGCTAB109007Rhodeusatremius                 nnnnnnnnnnnnnnnnnnnnnnnnnnacttattaaaattgctaacgacgcactagtcgacctaccaacaccctccaatatctcaatctgatgaaattttggctctcttctggggctgtgtttaatctctcaaatcctaacgggcctatttctggccatgcactacacctcagacatttcaaccgcattctcttcagttaaccacatctgccgagacgtaaattacggctgacttattcggaacctccacgccaacggcgcatccttcttcttcatctgcatttatatgcacattgcccgtggcctttattatggctcctacctctataaagagacctgaaatattggtgtcatcctgcttctactagtaataataaccgcctttgtgggctacgtactaccatggggccaaatatccttctgaggggctacagttatcactaatctgctttcagcagtcccctacatgggagacgccctagttcagtgaatctgaggcgggttctcagttgacaacgcaaccctgacccgattctttgcctttcatttccttctcccctttgcagtcgcagcggcaaccatcctccaccttctttttctacacgaaacaggatcaaacaaccccgccggtctaaactccgacgcggataagatctccttccacccctacttctcatacaaagacctgctagggtttgttctcatgcttatggctttaacatctctggcactattctcacccaacctgttaggtgacccagagaactttacccccgctaaccctcttgttaccccaccacacatcaagcccgagtgatactttttatttgcctacgccatcttacggtccatcccaaacaagcttggaggcgtccttgcattattattttctattttagtacttatagtagtcccaattctgcacacatcgaaacagcgcggactcacctttcgcccaatcacccagttcctattttgaaccttagtggcagatataataatcttgacatgaattggaggcatacctgttgaacacccatacatcnnnnnnnnnnnnnnnnnnnnnnnnnnnnnnnnnnnnnnnnnnnnnnnnnnnnnnnnnnnnnnnnnnnnnnnnnnnnnnnnnnnnnnnnnnnnnnnnHM560156Rhodeusamarus                   ATGGCAAGCCTACGAAAAACCCATCCACTTATTAAAATCGCCAATGACGCGCTAGTTGACCTACCAACACCCTCTAACATCTCAGTCTGGTGAAATTTTGGATCTCTACTGGGATTATGCTTAATTTCCCAAATCTTGACAGGCCTTTTCTTAGCTATGCACTACACCTCAGACATTTCAACCGCATTTTCTTCAGTAAACCATATCTGCCGCGATGTAAATTATGGCTGACTGATCCGAAATTTACACGCCAACGGCGCATCATTCTTTTTTATCTGCATCTACATACACATCGCCCGCGGTCTGTACTACGGGTCCTATCTCTACAAAGAGACCTGAAATATTGGTGTTATCCTCTTTCTGCTCGTCATAATAACCGCTTTCGTAGGCTACGTTTTACCCTGAGGTCAAATATCATTCTGGGGCGCCACAGTAATTACCAATCTACTCTCAGCGGTCCCTTATATAGGGGACGCTCTGGTTCAATGAATTTGAGGCGGGTTCTCAGTAGACAACGCAACACTAACCCGATTTTTCGCCTTTCACTTCCTTCTTCCATTTGTTATCGTAGCCGCAACCATCCTCCATCTCCTCTTCCTCCACGAGACAGGGTCGAATAACCCCGCGGGATTAAACTCCGACGCAGATAAAATTTCTTTCCACCCCTACTTCTCCTACAAAGATCTGCTAGGATTTGTACTTATACTAATAACCTTAACAGCCTTAGCATTATTTTCACCTAACCTACTAGGTGACCCAGAAAATTTTACTCCTGCCAACCCACTCGTTACACCACCGCACATCCAGCCCGAGTGATATTTTCTATTTGCCTACGCCATCTTACGATCTATCCCAAACAAACTTGGAGGTGTCCTAGCACTATTATTTTCTATTCTTGTACTTATAGTAGTGCCATTGCTCCATACATCCAAACAACGCGGACTAACCTACCGTCCAATCACTCAATTCTTATTTTGAACATTAGTGGCAGATATAATTATTCTGACATGGATTGGAGGCATACCCGTAGAGCACCCATACGTTATTATTGGGCAAATTGCGTCAGTGCTGTACTTCGCACTTTTTCTCGTCCTCGCGCCATTAGCCGGATGACTAGAAAATAAAGCATTAAAATGAGCTAB109009Rhodeusocellatus                nnnnnnnnnnnnnnnnnnnnnnnnnngctcattaaaatcgccaacgatgcactagttgatctgccaacaccctctaacatttcaatttgatgaaattttggctctcttcttggactttgcttaatttcccaaattctaacaggtttatttctggctatgcactacacctcagacatttcaaccgcattttcctcagttaaccacatctgccgtgatgttaactatggctgacttattcgaaacctacacgccaacggcgcatcattcttcttcatctgcatttatatgcacattgcccgaggcctatattatgggtcctatttatataaggaaacctgaaacatcggagttgtcctgcttctcctggtaataataaccgccttcgtaggctacgtcctgccctgaggccaaatatcattttgaggggccacagtcattactaatttactttcagcagtaccctacataggggacgccctagttcaatggatttgaggcggattttcagtagacaacgcaacactaacccgatttttcgcctttcacttcctatttccatttgtaatcgccgcggcaaccgtcctccacctcctatttcttcacgaaacaggatcaaacaaccccgccggcctaaactccgacgccgacaaaatttccttccacccctacttctcttataaagatctattaggttttgttcttatactaatagcattaacgtctctagcattattttcacctaacctattaggtgacccagaaaacttcacccccgccaacccccttgtcaccccaccacacattaagccagaatgatattttttatttgcctacgccatcttacgctccattcctaacaaattaggaggtgttcttgcgttattattttctattcttgtacttatagtggtcccaatcctgcatacgtcaaaacaacgaggactaacctatcgtcccatcacccaattcttattctgaaccctagtggcagatataatcattctgacatgaattggcggcatacctgtagaacatccatttattnnnnnnnnnnnnnnnnnnnnnnnnnnnnnnnnnnnnnnnnnnnnnnnnnnnnnnnnnnnnnnnnnnnnnnnnnnnnnnnnnnnnnnnnnnnnnnnnHM560157Rutilusaula                     ATGGCAAGCCTACGAaAAACCCACCCACTAATAAAAATCGCCAATGACGCGCTAGTCGACCTCCCAACACCATCTAATATTTCAGCAATATGAAACTTCGGATCCCTACTAGGATTATGTTTAATTACCCAAATCCTAACAGGATTATTCTTAGCCATGCACTATACCTCTGACATCTCAACCGCATTTTCATCGGTAACCCACATCTGCCGAGACGTCAACTATGGCTGACTTATTCGAAGTCTACACGCCAATGGGGCATCCTTCTTCTTCATCTGCCTTTATATACATATCGCACGTGGCCTATACTATGGGTCATACCTTTATAAAGAAACCTGAAACATTGGTGTAGTCCTATTTCTCCTAGTTATGATGACGGCCTTCGTCGGCTACGTACTGCCATGAGGACAAATATCATTCTGAGGCGCCACCGTAATCACGAACCTCCTCTCAGCAGTACCCTACATAGGGGACACCCTCGTTCAATGAATCTGGGGCGGTTTCTCAGTAGACAACGCAACCCTTACACGATTCTTCGCATTCCACTTCCTTCTCCCATTTGTCGTCACCGGCGCAACCATTCTACACCTACTATTTTTACACGAAACGGGGTCAAACAACCCGGCCGGACTAAATTCCGACGCAGATAAAATTTCTTTCCACCCATATTTCTCATATAAAGACCTCCTTGGCTTTGTAATTATATTACTAGCTCTCACCTCCTTAGCATTATTTTCTCCTAACCTATTAGGTGACCCAGAAAACTTTACGCCAGCAAACCCACTCGTAACACCTCCACATATTCAGCCAGAATGATACTTCTTATTTGCCTACGCCATTCTCCGATCCATCCCAAACAAACTAGGAGGGGTCCTTGCACTACTGTTCAGCATTCTAGTGCTAATAGTTGTCCCCCTCTTACACACCTCAAAACAGCGAGGACTAACTTTCCGCCCCATGACACAATTCTTATTCTGAACCCTAGTTGCAGATATGATTATCTTAACATGAATTGGAGGCATGCCTGTAGAACACCCATATATTATTATTGGCCAAATCGCATCAATTCTATACTTCGCACTCTTCCTCATTCTCGTCCCGCTAGCAGGATGAGTGGAAAATAAAGCATTGAAATGAGCCHM560158Rutilusaula                     ATGGCAAGCCTACGAAAAACCCACCCACTAATAAAAATCGCCAATGACGCGCTAGTCGACCTCCCAACACCATCTAATATTTCAGCAATATGAAACTTCGGATCCCTACTAGGATTATGTTTAATTACCCAAATCCTAACAGGATTATTCTTAGCCATGCACTATACCTCTGACATCTCAACCGCATTTTCATCGGTAACCCACATCTGCCGAGACGTCAACTATGGCTGACTTATTCGAAGTCTACACGCCAATGGGGCATCCTTCTTCTTCATCTGCCTTTATATACATATCGCACGTGGCCTATACTATGGGTCATACCTTTATAAAGAAACCTGAAACATTGGTGTAGTCCTATTTCTCCTAGTTATGATGACGGCCTTCGTCGGCTACGTACTGCCATGAGGACAAATATCATTCTGAGGCGCCACCGTAATCACGAACCTCCTCTCAGCAGTACCCTACATAGGGGACACCCTCGTTCAATGAATCTGGGGCGGTTTCTCAGTAGACAACGCAACCCTTACACGATTCTTCGCATTCCACTTCCTTCTCCCATTTGTCGTCACCGGCGCAACCATTCTACACCTACTATTTTTACACGAAACGGGGTCAAACAACCCGGCCGGACTAAATTCCAACGCAGATAAAATTTCTTTCCACCCATATTTCTCATATAAAGACCTCCTTGGCTTTGTAATTATATTACTAGCTCTCACCTCCTTAGCATTATTTTCTCCTAACCTATTAGGTGACCCAGAAAACTTTACGCCAGCAAACCCACTCGTAACACCTCCACATATTCAGCCAGAATGATACTTCTTATTTGCCTACGCCATTCTCCGATCCATCCCAAACAAACTAGGAGGGGTCCTTGCACTACTGTTCAGCATTCTAGTGCTAATAGTTGTCCCCCTCTTACACACCTCAAAACAGCGAGGACTAACTTTCCGCCCCATGACACAATTCTTATTCTGAACCCTAGTTGCAGATATGATTATCTTAACATGAATTGGAGGCATGCCTGTAGAACACCCATATATTATTATTGGCCAAATCGCATCAATTCTATACTTCGCACTCTTCCTCATTCTCGTCCCGCTAGCAGGATGAGTGGAAAATAAAGCATTGAAATGAGCCHM560159Rutilusbasak                    ATGGCAAGCCTACGAAAAACCCATCCACTAATAAAAATCGCCAATGACGCGCTAGTTGACCTCCCAACACCATCTAATATCTCAGCAATGTGAAACTTCGGGTCCCTACTAGGATTGTGTTTAATTACCCAAATCCTGACAGGATTGTTCTTAGCCATGCACTATACCTCTGACATCTCAACCGCATTTTCACCGGTGACTCACATCTGCCGAGACGTCAACTATGGCTGACTTATTCGAAGTCTACACGCCAATGGGGCATCCTTCTTCTTCATCTGTCTTTATATACATATCGCACGTGGCCTATACTATGGATCATACCTTTATAGAGAAACCTGAAACATTGGTGTAGTACTATTCCTCCTAGTTATAATGACAGCCTTCGTCGGCTACGTACTGCCATGAGGACAAATATCATTCTGGGGCGCCACCGTAATCACGAACCTCCTCTCAGCAGTCCCCTACATAGGGGACACCCTCGTTCAATGAATCTGGGGCGGTTTCTCAGTAGACAACGCAACCCTTACACGATTCTTCGCATTCCACTTCCTTCTCCCATTTGTCGTCGCCGGCGCAACCATTCTGCACCTACTATTTTTACACGAAACGGGGTCAAACAACCCAGCCGGACTAAATTCCGACGCAGATAAAATTTCTTTCCACCCATATTTCTCATATAAAGACCTCCTTGGCTTTGTAATTATGTTACTAGCTCTCACCTCCCTAGCATTGTTTTCTCCTAACCTATTAGGTGACCCAGAAAACTTTACGCCAGCAAACCCACTCGTAACACCTCCACATATTCAGCCAGAATGATACTTCTTATTTGCCTACACCATTCTCCGATCCACCCCAAACAAACTAGGAGGGGTCCTTGCACTACTATTCAGCATTCTAGTGCTAATAGTTGTCCCCATCTTACATACCTCAAAACAGCGAGGACTAACCTTCCGCCCCGTGACACAATTCTTATTCTGAACCCTAGTTGCAGATATGATTATCTTGACATGAATTGGAGGCATGCCTGTAGAACACCCATATATCATTATTGGCCAAATCGCATCCATTCTATACTTCGCGCTCTTCCTCGTTCTCGTCCCGCTAGCAGGGTGAGTGGAAAATAAAGCATTGAAATGAGCCHM560160Rutilusbasak                    ATGGCAAGCCTACGAAAAACCCATCCACTGATAAAAATCGCCAATGACGCGCTAGTTGACCTCCCAACACCATCTAATATCTCAGCAATGTGAAACTTCGGGTCCCTACTAGGATTGTGTTTAATTACCCAAATCCTGACAGGATTGTTCTTAGCCATGCACTATACCTCTGACATCTCAACCGCATTTTCATCGGTGACTCACATCTGCCGAGACGTCAACTATGGCTGACTTATTCGAAGTCTACACGCCAATGGGGCATCCTTCTTCTTCATCTGTCTTTATATACATATCGCACGTGGCCTATACTATGGATCATACCTTTATAAAGAAACCTGAAACATTGGTGTAGTACTATTCCTCCTAGTTATAATGACAGCCTTCGTCGGCTACGTACTGCCATGAGGACAAATATCATTCTGAGGCGCCACCGTAATCACGAACCTCCTCTCAGCAGTCCCCTACATAGGGGACACCCTCGTTCAATGAATCTGGGGCGGTTTCTCAGTAGACAACGCAACCCTTACACGATTCTTCGCATTCCACTTCCTTCTCCCATTTGTCGTCGCCGGCGCAACCATTCTGCACCTACTATTTTTACACGAAACGGGGTCAAACAACCCAGCCGGACTAAATTCCGACGCAGATAAAATTTCTTTCCACCCATATTTCTCATATAAAGACCTCCTTGGCTTTGTAATTATGTTACTAGCTCTCACCTCCCTAGCATTGTTTTCTCCTAACCTATTAGGTGACCCAGAAAACTTTACGCCAGCAAACCCACTCGTAACACCTCCACATATTCAGCCAGAATGATACTTCTTATTTGCCTACGCCATTCTCCGATCCATCCCAAACAAACTAGGAGGGGTCCTTGCACTACTATTCAGCATTCTAGTGCTAATAGTTGTCCCCATCTTACATACCTCAAAACAGCGAGGACTAACCTTCCGCCCCGTGACACAATTCTTATTCTGAACCCTAGTTGCAGATATGATTATCTTGACATGAATTGGAGGCATGCCTGTAGAACACCCATATATCATTATTGGCCAAATCGCATCCATTCTATACTTCGCGCTCTTCCTCGTTCTCGTCCCGCTAGCAGGGTGAGTGGAAAATAAAGCATTGAAATGAGCCAF095610Rutilusfrisii                   atggcaagcctacgaaaaacccatccactaataaaaatcgctaatgacgcactagtcgacctcccaacaccatctaatatctcagcaatgtgaaacttcggatccctactaggattatgtttaattacccaaatcctgacaggattattcttagctatgcactatacctctgacatctcaaccgcattttcatcggtaacccatatttgccgagacgtcaactatggctggcttatccgaaacctacacgctaatggggcatccttcttcttcatctgtntttatatacatatcgcacgaggcctatactacggatcatacctttataaagaaacctgaaacattggtgtagtcctattcctcctagttataatgacagccttcgtcggctacgtattgccatgaggacaaatatccttctgaggtgccaccgtaatcacgaacctcctctcagcagtcccttacataggagataccctcgttcaatgaatctgaggcggtttctcagtagacaacgcaacccttacacgattcttcgcattccacttcctcctaacgtttgtcgtcgccggcgcaaccatcctacacctactatttttacacgaaacggggtcaaacaacccggccgggctaaactccgacgcagataaaatttctttccacccatatttctcatataaagacctccttggctttgtaattatgttactagctctcacctctttagcattattttctcctaacctattaggtgacccagaaaactttacgccagcaaacccactcgtgacacccccacatattcagccagaatgatacttcttatttgcctacgccattctccgatccatcccaaacaaactaggaggggtcctcgcactactattcagcattctagtgctaatagttgtcccaatcttacatacctcaaaacaacgaggactaactttccgccccgtgacacaatttttattctgaaccctagttgcagatatatttatcttaacatgaattggaggcatgcctgtagaacacccatacattattattggccaaattgcatccattctatacttcgcgctcttcctcattctcgtcccactagcaggatgagtggaaaataaagcattgaaatgagccHM560161Rutilusfrisii                   ATGGCAAGCCTACGAAAAACCCATCCACTAATAAAAATCGCTAATGACGCACTAGTCGACCTCCCAACACCATCTAATATCTCAGCAATGTGAAACTTCGGATCCCTACTAGGATTATGTTTAATTACCCAAATCCTGACAGGATTATTCTTAGCTATGCACTATACCTCTGACATCTCAACCGCATTTTCATCGGTAACCCATATTTGCCGAGACGTCAACTATGGCTGGCTTATCCGAAACCTACACGCTAATGGGGCATCCTTCTTCTTCATCTGTCTTTATATACATATCGCACGAGGCCTATACTACGGATCATACCTTTATAAAGAAACCTGAAACATTGGTGTAGTCCTATTCCTCCTAGTTATAATGACAGCCTTCGTCGGCTACGTATTGCCATGAGGACAAATATCCTTCTGAGGTGCCACCGTAATCACGAACCTCCTCTCAGCAGTCCCTTACATAGGAGATACCCTCGTTCAATGAATCTGAGGCGGTTTCTCAGTAGACAACGCAACCCTTACACGATTCTTCGCATTCCACTTCCTCCTACCGTTTGTCGTCGCCGGCGCAACCATCCTACACCTACTATTTTTACACGAAACGGGGTCAAACAACCCGGCCGGGCTAAACTCCGACGCAGATAAAATTTCTTTCCACCCATATTTCTCATATAAAGACCTCCTTGGCTTTGTAATTATGTTACTAGCTCTCACCTCTTTAGCATTATTTTCTCCTAACCTATTAGGTGACCCAGAAAACTTTACGCCAGCAAACCCACTCGTGACACCCCCACATATTCAGCCAGAATGATACTTCTTATTTGCCTACGCCATTCTCCGATCCATCCCAAACAAACTAGGAGGGGTCCTCGCACTACTATTCAGCATTCTAGTGCTAATAGTTGTCCCAATCTTACATACCTCAAAACAACNAGNACTAACTTTCCGCCCCGTGACACAATTTTTATTCTGAACCCTAGTTGCAGATATATTTATCTTAACATGAATTGGAGGCATGCCTGTAGAACACCCATACATTATTATTGGCCAAATTGCATCCATTCTATACTTCGCGCTCTTCCTCATTCTCGTCCCACTAGCAGGATGAGTGGAAAATAAAGCATTGAAATGAGCCEU285047Rutilusfrisii                   ATGGCAAGCCTACGAAAAACCCATCCACTAATAAAAATCGCTAATGACGCACTAGTCGACCTCCCAACACCATCTAATATCTCAGCAATGTGAAACTTCGGATCCCTACTAGGATTATGTTTAATTACCCAAATCCTAACAGGATTATTCTTAGCTATGCACTATACCTCTGACATCTCAACCGCATTTTCATCAGTAACCCATATTTGCCGAGACGTCAACTATGGCTGGCTTATCCGAAACCTGCACGCTAATGGGGCATCCTTCTTCTTCATCTGTCTTTATATACATATCGCACGAGGCCTATACTACGGATCATACCTTTATAAAGAAACCTGAAACATTGGTGTAGTCCTATTCCTCCTAGTTATAATGACAGCCTTCGTCGGCTACGTATTGCCATGAGGACAAATATCCTTCTGAGGTGCCACCGTAATCACGAACCTCCTCTCAGCAGTCCCTTACATAGGGGATACCCTCGTTCAATGAATCTGAGGCGGTTTCTCAGTAGACAACGCAACCCTTACACGATTCTTCGCATTCCACTTCCTCCTACCGTTTATCGTCGCCGGCGCAACCATCCTACACCTACTATTTTTACACGAAACAGGGTCAAACAACCCGGCCGGGCTAAACTCCGACGCAGATAAAATTTCTTTCCACCCATATTTCTCATATAAAGACCTCCTTGGCTTTGTAATTATGTTACTAGCTCTCACCTCTTTAGCATTATTTTCTCCTAACCTATTAGGTGACCCAGAAAACTTTACGCCAGCAAACCCACTCGTGACACCCCCACATATTCAGCCAGAATGATACTTCTTATTTGCCTACGCCATTCTCCGATCCATCCCAAACAAACTAGGAGGGGTCCTCGCACTACTATTCAGCATTCTAGTGCTAATAGTTGTCCCAATCTTACATACCTCAAAACAACGAGGACTAACTTTCCGCCCCGTGACACAATTTTTATTCTGAACCCTAGTTGCAGATATATTTATCTTAACATGAATTGGAGGCATGCCTGTAGAACACCCATACATTATTATTGGCCAAATTGCATCCATTCTATACTTCGCGCTCTTCCTCATTCTCGTCCCACTAGCAGGATGAGTGGAAAATAAAGCATTGAAATGAGCCEU285044Rutilusfrisii                   ATGGCAAGCCTACGAAAAACCCATCCACTAATAAAAATCGCTAATGACGCACTAGTCGACCTCCCAACACCATCTAATATCTCAGCAATGTGAAACTTCGGATCCCTACTAGGATTATGTTTAATTACCCAAATCCTAACAGGATTATTCTTAGCTATGCACTATACCTCTGACATCTCAACCGCATTTTCATCAGTAACCCATATTTGCCGAGACGTCAACTATGGCTGGCTTATCCGAAACCTGCACGCTAATGGGGCATCCTTCTTCTTCATCTGTCTTTATATACATATCGCACGAGGCCTATACTACGGATCATACCTTTATAAAGAAACCTGAAACATTGGTGTAGTCCTATTCCTCCTAGTTATAATGACAGCCTTCGTCGGCTACGTATTGCCATGAGGACAAATATCCTTCTGAGGTGCCACCGTAATCACGAACCTCCTCTCAGCAGTCCCTTACATAGGGGATACCCTCGTTCAATGAATCTGAGGCGGTTTCTCAGTAGACAACGCAACCCTTACACGATTCTTCGCATTCCACTTCCTCCTACCGTTTGTCGTCGCCGGCGCAACCATCCTACACCTACTATTTTTACACGAAACAGGGTCAAACAACCCGGCCGGGCTAAACTCCGACGCAGATAAAATTTCTTTCCACCCATATTTCTCATATAAAGACCTCCTTGGCTTTGTAATTATGTTACTAGCTCTCACCTCTTTAGCATTATTTTCTCCTAACCTATTAGGTGACCCAGAAAACTTTACGCCAGCAAACCCACTCGTGACACCCCCACATATTCAGCCAGAATGATACTTCTTATTTGCCTACGCCATTCTCCGATCCATCCCAAACAAACTAGGAGGGGTCCTCGCACTACTATTCAGCATTCTAGTGCTAATAGTTGTCCCAATCTTACATACCTCAAAACAACGAGGACTAACTTTCCGCCCCGTGACACAATTTTTATTCTGAACCCTAGTTGCAGATATATTTATCTTAACATGAATTGGAGGCATGCCTGTAGAACACCCATACATTATTATTGGCCAAATTGCATCCATTCTATACTTCGCGCTCTTCCTCATTCTCGTCCCACTAGCAGGATGAGTGGAAAATAAAGCATTGAAATGAGCCAF090772Rutilusheckeli                  atggcaagcctacgaaaaacccatccactaataaaaatcgctaatgacgcgctagtcgaccttccaacaccatctaacatctcagcactatgaaatttcgggtccctgctaggattatgtttaattacccaaatcctaacaggactattcttagccatgcactacacctcagacatctcaaccgcattttcatcggtaacccacatctgccgagacgtcaactacggctgacttatccgaagcctacacgctaacggagcatccttcttcttcatctgcctttacatacatatcgcacgaggcctatactacgggtcatatctttataaagaaacctgaaacattggtgtcgttctattcctcctggttataatgacagccttcgttggctacgtactgccatgaggccaaatatccttctgaggtgccaccgtgatcacaaacctcctctcagcagtcccttacatgggggatactcttgttcagtgaatctggggaggtttctcggtagacaacgcaacccttacacgaatcttcgcattccactttctcttcccatttgtcgtcgccggcgcaaccgttctacacttactgtttttacacgaaacgggatcaaacaacccggctggactaaactccgacgcagataaaatttctttccatccatatttttcatacaaagacctccttggctttgtaattatattactagctctcacttccctaacactattttctcctaacctattaggtgacccagaaaactttacgccagcaaacccactcgtgacacccccacatattcagccagaatggtacttcttatttgcctacgccatcctccgatccatcccaaacaaactaggaggggtcctcgcactactgttcagcattctagtgctaatggttgtcccaatcttacacacctcaaaacaacgaggactaactttccgccctgtaacacaatttttattctgaaccctagttgcagatatatttatcttaacatgaatcggaggcatgcccgtagaacacccatatatcgttattggccaaattgcatccattctatactttgcacttttcctcgttcttgtcccactagcaggatgagcggaaaataaagcattgaaatgagccFJ025074Rutilusheckeli                  ATGGCAAGCCTACGAAAAACCCATCCACTAATAAAAATCGCTAATGACGCGCTAGTCGACCTTCCAACACCATCTAACATCTCAGCACTATGAAATTTCGGGTCCCTGCTAGGATTATGTTTAATTACCCAAATCCTAACAGGACTATTCTTAGCCATGCACTACACCTCAGACATCTCAACCGCATTTTCATCGGTAACCCACATCTGCCGAGACGTCAACTACGGCTGACTTATCCGAAGCCTACACGCTAACGGAGCATCCTTCTTCTTCATCTGCCTTTACATGCATATCGCACGAGGCCTATACTACGGGTCATATCTTTATAAAGAAACCTGAAACATTGGTGTCGTTCTATTCCTCCTGGTTATAATGACAGCCTTCGTTGGCTACGTACTGCCATGAGGCCAAATATCCTTCTGAGGTGCCACCGTGATCACAAACCTCCTCTCAGCAGTCCCTTACATAGGGGATACTCTTGTTCAGTGAATCTGAGGAGGTTTCTCGGTAGACAACGCAACCCTTACACGATTCTTCGCATTCCACTTTCTCTTCCCATTTGTCGTCGCCGGCGCAACCGTTCTACACTTACTGTTTTTACACGAAACGGGATCAAACAACCCGGCTGGACTAAACTCCGACGCAGATAAAATTTCTTTCCATCCATATTTTTCATACAAAGACCTCCTTGGCTTTGTAATTATATTACTAGCTCTCACTTCCCTAACACTATTTTCTCCTAACCTATTAGGTGACCCAGAAAACTTTACGCCAGCAAACCCACTCGTGACACCCCCACATATTCAGCCAGAATGGTACTTCTTATTTGCCTACGCCATCCTCCGATCCATCCCAAACAAACTAGGAGGGGTCCTCGCACTACTGTTCAGCATTCTAGTGCTAATAGTTGTCCCAATCTTACACACCTCAAAACAACGAGGACTAACTTTCCGCCCTGTGACACAATTTTTATTCTGAACCCTGGTTGCAGATATATTTATCTTAACATGAATCGGAGGCATGCCCGTAGAACACCCATATATCGTTATTGGCCAAATTGCATCCATTCTATACTTTGCACTTTTCCTCGTTCTTGTCCCACTAGCAGGATGAGCGGAAAATAAAGCATTGAAATGAGCCFJ025085Rutilusohridanus                ATGGCAAGCCTACGAAAAACCCACCCACTAATAAAAATCGCCAACGACGCGCTAGTCGACCTCCCAACACCATCTAATATCTCAGCAATATGAAACTTCGGATCCCTACTAGGATTATGTTTAATTACCCAAATCCTAACAGGATTATTCTTAGCCATGCACTACACCTCTGACATCTCAACCGCATTTTCATCAGTAACCCACATCTGCCGAGAAGTAAACTACGGCTGACTTATTCGAAGCCTACATGCCAACGGAGCATCCTTCTTCTTCATCTGTCTTTATATACATATCGCACGGGGCCTATATTATGGATCATACCTTTACAAAGAAACCTGAAATATTGGTGTAGTCCTATTCCTTTTGGTTATGATAACGGCCTTCGTCGGTTACGTACTTCCATGAGGGCAAATGTCCTTTTGGGGTGCCACCGTAATTACAAACCTCCTCTCAGCAGTCCCCTACATAGGAGACACCCTTGTTCAGTGAATCTGAGGCGGTTTCTCAGTAGACAACGCGACCCTAACACGATTCTTCGCGTTCCACTTCCTTCTGCCGTTTGTCGTCGCGGGCGCAACCATTCTACACTTACTGTTTCTACACGAAACGGGGTCAAACAACCCGGCCGGACTAAATTCCGACGCAGACAAAATTTCTTTCCACCCGTACTTTTCATATAAAGACCTCCTTGGCTTTGTAATTATGTTACTAGCTCTCACCTCCTTAGCATTATTTTCTCCTAACCTATTAGGGGACCCAGAAAACTTTACGCCAGCAAACCCACTCGTAACGCCTCCACATATTCAGCCAGAATGATACTTCTTATTTGCCTACGCCATTCTCCGATCCATCCCAAACAAACTAGGAGGGGTACTTGCACTACTATTCAGCATTCTAGTGCTAATAGTTGTCCCCATCTTACACACCTCAAAACAGCGAGGACTAACTTTCCGCCCCGTGACACAATTCTTATTCTGAACCCTAGTTGCAGATATGATTATTTTAACATGAATTGGAGGCATGCCTGTAGAACACCCATATATCATTATTGGCCAAATCGCATCCATTCTATACTTCGCGCTCTTCCTCGTTCTCGTCCCGCTAGCAGGATGGATAGAAAATAAAGCATTGAAATGAGCCFJ025071Rutiluspanosi                   ATGGCAAGCCTACGAAAAACCCATCCACTAATGAAAATCGCTAATGACGCACTAGTCGACCTTCCAACACCATCTAATATCTCAGCAATGTGAAACTTCGGATCCCTGCTGGGATTATGTCTAATTACCCAAATCCTAACGGGATTATTCTTAGCCATGCACTATACCTCTGATATCTCAACCGCGTTTTCGTCAGTAACTCACATCTGCCGAGACGTCAACTATGGCTGACTTATTCGAAGCCTGCACGCCAATGGAGCGTCCTTCTTCTTCATCTGTCTTTATATGCACATCGCACGAGGCCTGTACTATGGATCATACCTTTATAAAGAAACCTGAAACATTGGCGTAGTCCTATTCCTCCTAGTTATAATGACAGCCTTCGTCGGCTACGTACTCCCATGGGGACAAATGTCGTTCTGGGGCGCCACCGTGATCACAAACCTTCTCTCGGCAGTCCCCTATATGGGGGACACCCTTGTTCAATGAATCTGGGGGGGCTTCTCGGTAGACAACGCGACTCTTACGCGATTCTTCGCATTCCACTTCCTTCTACCATTTGTCGTCGCCGGCGCAACCGTCCTACACTTACTATTTTTACACGAAACGGGGTCAAACAACCCGGCCGGACTAAATTCCGACGCAGATAAAATTTCTTTCCACCCATATTTCTCATATAAAGACCTCCTTGGCTTTGTAATTATGTTACTAGCTCTCACCTCCCTAGCATTGTTTTCTCCTAACCTATTAGGTGACCCAGAAAACTTTACGCCAGCAAACCCACTGGTGACACCCCCACATATTCAGCCAGAATGATACTTCTTATTTGCCTACGCCATTCTCCGATCTATCCCGAATAAACTAGGAGGGGTACTCGCACTACTGTTCAGCATTCTAGTGCTAATGGTTGTCCCAATCTTACACACCTCAAAGCAACGAGGACTAACTTTCCGCCCTGTAACACAATTCTTATTCTGAACCCTAGTTGCAGATATGATTATCTTAACATGGATTGGAGGCATGCCTGTAGAACACCCATATATCGTTATTGGCCAAGTCGCATCCGTCCTGTACTTCGCGCTCTTCCTCATTCTCGTCCCGCTAGCAGGATGAATGGAAAATAAAGCATTGAAATGAGCCAF090774Rutiluspanosi                   atggcaagcctacgaaaaacccatccactaatgaaaatcgctaatgacgcactagtcgacctcccaacaccatctaatatctcagcaatgtgaaacttcggatccctgctgggattatgtctaattacccaaatcctaacgggattattcttagccatgcactatacctctgatatctcaaccgcgttttcgtcagtaacccacatctgccgagacgtcaactatggctgacttattcgaagcctgcacgccaatggagcgtccttcttcttcatctgtctttatatgcacatcgcacgaggcctgtactatggatcatacctttataaagaaacctgaaacattggcgtagtcctattcctcctagttataatgacagccttcgtcggatacgtactcccatggggacaaatgtcgttctggggcgccaccgtgatcacaaaccttctctcggcagtcccctatatgggggacacccttgttcaatgaatctggggaggcttctcggtagacaacgcgactcttacgcgattcttcgcattccacttccttctaccatttgtcgtcgccggcgcaaccgtcctacacttactatttttacacgaaacggggtcaaacaacccggccggactaaattccgacgcagataaaatttctttccacccatatttctcatataaagacctccttggctttgtaattatgttactagctctcacttccctagcattgttttctcctaacctattaggtgacccagaaaactttacgccagcaaacccactggtgacacccccacatattcagccagaatgatacttcttatttgcctacgccattctccgatctatcccgaataaactaggaggggtactcgcactactgttcagcattctagtgctaatggttgtcccaatcttacatacctcaaagcaacgaggactaactttccgccctgtaacacaattcttattctgaaccctagttgcagatatgattatcttaacatggattggaggcatgcctgtagaacacccatatatcgttattggccaagtcgcatccgtcctatacttcgcgctcttcctcattctcgtcccgctagcaggatgaatggaaaataaagcattgaaatgagccHM560162Rutiluspigus                    ATGGCAAGCCTACGAAAAACCCATCCACTAATAAAAATCGCTAATGACGCACTAGTTGACCTTCCAACACCATCTAATATCTCAGCACTATGAAACTTCGGGTCCCTACTAGGATTGTGTTTAATTACTCAAATCCTAACAGGATTATTCTTAGCTATGCACTACACCTCTGACATCTCAACCGCATTTTCATCAGTGACCCATATCTGCCGAGACGTCAACTACGGTTGACTTATCCGAAGCCTACATGCCAATGGAGCATCCTTCTTCTTCATCTGTCTTTATATACATATCGCACGAGGCCTGTACTACGGGTCATACCTTTATAAAGAAACCTGAAACATTGGTGTAGTTCTGTTCCTCCTAGTTATAATGACAGCCTTTGTCGGCTATGTACTGCCGTGGGGACAAATATCCTTCTGAGGTGCCACCGTAATCACGAACCTCCTCTCAGCAGTCCCTTACATAGGGGACACCCTCGTTCAATGAATCTGAGGTGGTTTCTCAGTAGATAACGCAACCCTTACGCGGTTCTTTGCATTCCACTTCCTCCTCCCATTTGTCGTCGCCGGCGCAACTGTCCTGCACCTACTATTTTTACACGAAACAGGGTCAAACAACCCAGCCGGACTAAACTCCGACGCGGATAAAATTTCTTTCCACCCATATTTCTCATATAAAGACCTCCTCGGCTTTGTAATTATGTTACTAGCTCTCACTTCCCTAACACTGTTTTCTCCTAACCTACTAGGTGACCCAGAAAACTTTACGCCAGCAAACCCACTCGTGACACCCCCACATATTCAGCCAGAATGATACTTCTTATTTGCCTACGCCATCCTCCGATCTATCCCAAATAAACTAGGAGGGGTCCTCGCACTACTATTTAGCATTCTAGTGCTAATAGTCGTCCCAATTTTACACACCTCTAAACAACGAGGACTAACCTTCCGCCCCGTAACACAATTTCTATTCTGAACCCTAGTTGCAGATATATTTATCTTAACATGGATTGGAGGCATACCCGTAGAACACCCATATATTATTATTGGCCAAATTGCATCCATTCTATACTTTGCGCTCTTCCTCGTTCTTGTCCCACTAGCAGGATGAGTGGAAAATAAAGCATTGAAATGAGCCHM560163Rutiluspigus                    ATGGCAAGCCTACGAAAAACCCATCCACTAATAAAAATCGCTAATGACGCACTAGTTGACCTTCCAACACCATCTAATATCTCAGCACTATGAAACTTCGGGTCCCTACTAGGATTGTGTTTAATTACTCAAATCCTAACAGGATTATTCTTAGCTATGCACTACACCTCTGACATCTCAACCGCATTTTCATCAGTGACCCATATCTGCCGAGACGTCAACTACGGTTGACTTATCCGAAGCCTACATGCCAATGGAGCATCCTTCTTCTTCATCTGTCTTTATATACATATCGCACGAGGCCTGTACTACGGGTCATACCTTTATAAAGAAACCTGAAACATTGGTGTAGTTCTGTTCCTCCTAGTTATAATGACAGCCTTTGTCGGCTATGTACTGCCGTGGGGACAAATATCCTTCTGAGGTGCCACCGTAATCACGAACCTCCTCTCAGCAGTCCCTTACATAGGGGACACCCTCGTTCAATGAATCTGAGGTGGTTTCTCAGTAGATAACGCAACCCTTACGCGGTTCTTTGCATTCCACTTCCTCCTCCCATTTGTCGTCGCCGGCGCAACTGTCCTGCACCTACTATTTTTACACGAAACAGGGTCAAACAACCCGGCCGGACTAAACTCCGACGCGGATAAAATTTCTTTCCACCCATATTTCTCATATAAAGACCTCCTCGGCTTTGTAATTATGTTACTAGCTCTCACTTCCCTAACACTGTTTTCTCCTAACCTACTAGGTGACCCAGAAAACTTTACGCCAGCAAACCCACTCGTGACACCCCCACATATTCAGCCNGAATGATACTTCTTATTTGCCTACGCCATCCTCCGATCTATCCCAAATAAACTAGGAGGGGTCCTCGCACTACTATTTAGCATTCTAGTGCTAATAGTCGTCCCAATTTTACACACCTCTAAACAACGAGGACTAACTTTCCGCCCCGTAACACAATTTCTATTCTGAACCCTAGTTGCAGATATATTTATCTTAACATGGATTGGAGGCATACCCGTAGAACACCCATATATTATTATTGGCCAAATTGCATCCATTCTATACTTTGCACTCTTCCTCGTTCTTGTCCCACTAGCAGGATGAGTGGAAAATAAAGCATTGAAATGAGCCAF090771Rutilusprespensis               gtggcaagcctacgaaaaacccacccactaataaaaatcgccaacgacgcgctagtcgaccttccaacaccatctaatatctcagcaatatgaaacttcggatccctactaggattatgtttaattacccaaatcctaacaggattattcttagccatgcactacacctctgacatctcaaccgcattttcatcagtaacccacatctgccgagacgtcaattatggctgacttattcgaagcctacacgccaatggggcgtccttcttcttcatctgtctttatatacatatcgcacggggcctatactatggatcatacctttataaagaaacctgaaacattggtgtagtcctattcctcctagttataataacagccttcgtcggctacgtgctgccatgaggacaaatatcattctggggcgccaccgtaattacgaacctcctctcagcagtcccctacataggtgacacccttgttcaatgaatctggggcggtttctcagtagacaacgcaacccttacacgattcttcgcattccacttccttctcccatttgtcgtcgccggcgcaaccatcctacacttactatttttacacgaaacggggtcaaacaacccggccggactaaattccgacgcagacaaaatttctttccacccgtacttttcatataaagacctccttagctttgtaattatgttactagctctcacctccttagcattattttctcctaacctattaggtgacccagaaaactttacgccagcaaacccactcgtaacgcctccacatattcagccagaatgatacttcttatttgcctacgccattctccgatccatcccaaacaaactaggaggggtacttgcactactattcagcattctagtgctaatagttgtccccatcttacacacctcgaaacagcgaggactaactttccgccccgtgacacaattcttattctgaaccctagttgcagatatgattattttaacatgaattggaggcatgcctgtagaacacccatatatcattattggccaaatcgcatccattctatacttcgcgctcttcctcgttctcgtcccgctagcaggatggatagaaaataaagcattgaaatgagccHM560164Rutilusprespensis               ATGGCAAGCCTACGAAAAACCCACCCACTAATAAAAATCGCCAACGACGCGCTAGTCGACCTTCCAACACCATCTAATATCTCAGCAATATGAAACTTCGGATCCCTACTAGGATTATGTTTAATTACCCAAATCCTAACAGGATTATTCTTAGCCATGCACTACACCTCTGACATCTCAACCGCATTTTCATCAGTAACCCACATCTGCCGAGACGTCAATTATGGCTGACTTATTCGAAGCCTACACGCCAATGGGGCGTCCTTCTTCTTCATCTGTCTTTATATACATATCGCACGGGGCCTATACTATGGATCATACCTTTATAAAGAAACCTGAAACATTGGTGTAGTCCTATTCCTCCTAGTTATAATAACAGCCTTCGTCGGCTACGTGCTGCCATGAGGACAAATATCATTCTGGGGCGCCACCGTAATTACGAACCTCCTCTCAGCAGTCCCCTACATAGGTGACACCCTTGTTCAATGAATCTGGGGCGGTTTCTCAGTAGACAACGCAACCCTTACACGATTCTTCGCATTCCACTTCCTTCTCCCATTTGTCGTCGCCGGCGCAACCATCCTACACTTACTATTTTTACACGAAACGGGGTCAAACAACCCGGCCGGACTAAATTCCGACGCAGACAAAATTTCTTTCCACCCGTACTTTTCATATAAAGACCTCCTTGGCTTTGAAATTATGTTACTAGCTCTCACCTCCTTAGCATTATTTTCTCCTAACCTATTAGGTGACCCAGAAAACTTTACGCCAGCAAACCCACTCGTAACGCCTCCACATATTCAGGCAGAATGATACTTCTTATTTGCCTACGCCATTCTCCGATCCATCCCAAACAAACTAGGAGGGGTACTTGCACTACTATTCAGCATTCTAGTGCTAATAGTTGTCCCCATCTTACACACCTCGAAACAGCGAGGACTAACTTTCCGCCCCGTGACACAATTCTTATTCTGAACCCTAGTTGCAGATATGATTATTTTAACATGAATTGGAGGcatgcctgtagaacacccatatatcattattggccaaatcgcatccattctatacttcgcgctcttcctcgttctcgtcccgctagcaggatggatagaaaataaagcattgaaatgagccHM560165Rutilusrubilio                  ATGGCAAGCCTACGAAAAACCCAnCCGCTAATAAAAATCgCTAACGACgCGCTagTCAACCTTccaACACCAtcTAANATCTCagNAATGTGAAACTTCGGATCCCTGCTAGGATTATGCTTAATTACTCANATCCTAACAGGACTATTCTTAGCCATGCACTATACCTCTGACATCTCGACCGCATTTTCATCAGTAACCCACATCTGCCGAGACGTCAACTATGGTTGGCTTATTCNAAGCCTACACGCCAACGGAGCATCCTTCTTTTTCATCTGTCTTTACATACATATTGCACGAGGCCTATACTACGGATCATACCTTTATAAAGAAACCTGAAACATTGGTGTAGTTCTATTCCTTTTAGTTATAATGACAGcCTTTGTAGGCTACGtACTGCCATGAGGACAAATGTCCTttTGAGGTGCCACTGtCaTTACgAACCTCCTCTCAgCAGtCCCTTAcAtaGGAgATaCTCTTGtTCAGtGAATCTGAgGCGGTTCCTCCGTAGACAACGCGACCCTcACACGATTTTTCGCGTTCCACTTCCTCCCCCCGTTtGTCGTCACCGGCGCAACCGTTCTGCACCTACTATTTTTACACGAAACTGGGTCAAACAACCCAGCCGGGcTAAACTCCGACGCAGATAAAATTTCTTTCCACCCATATTTCTCATATAAAGATCTCCTCGGCTTTGTAATTATATTACTAGCTCTCACCTCCTTAGCATTATTTTCTCCCAACCTATTGGGTGACCCGGAGAACTTTACGCCGGCAAACCCACTCGTGACACCCCCACATATTCAGCCAGAATGATACTTCTTATTTGCCTACGCCATTCTCCGGTCCATCCCAAACAAACTAGGAGGGGTCCTCGCACTACTATTCAGCATTCTAGTGCTAATGGTTGTCCCAATCTTACACACGTCGAAACAACGAGGACTAACCTTCCGCCCCGTAACACAATTCTTATTcTGAACCCTGGTTGCAGATATATTTATCCTAACATGGATTGGAGGCATGCCCGTAGAACACCCATATATTATTATTGGCCAAGTNGCATcCNTTcTAtACTtCgcGTTGtTNnnnnnnnnnnnnnnnnnnnnnnnnnnnnnnnnnnnnnnnnnnnnnnnnnnnnnnHM560166Rutilusrubilio                  ATGGCAAGCCTACGAAAAACCCACCCACTAATAAAAATCGCCAATGACGCGCTAGTCGACCTTCCGACACCATCTAATATCTCAGCAATGTGAAACTTCGGATCCCTGCTAGGATTATGCTTAATTACTCAAATCCTAACAGGACTATTCTTAGCCATGCACTATACCTCTGACATCTCGACCGCATTTTCATCAGTAACCCACATCTGCCGAGACGTCAACTATGGTTGGCTTATTCGAAGCCTACACGCCAACGGAGCATCCTTCTTTTTCATCTGTCTTTACATACATATTGCACGAGGCCTATACTACGGATCATACCTTTATAAAGAAACCTGAAACATTGGTGTAGTTCTATTCCTTTTAGTTATAATGACAGCCTTTGTAGGCTACGTACTGCCATGAGGACAAATGTCCTTCTGAGGTGCCACTGTCATTACGAACCTCCTCTCAGCAGTCCCTTACATAGGAGATACTCTTGTTCAGTGAATCTGAGGCGGTTTCTCCGTAGACAACGCGACCCTTACACGATTCTTCGCGTTCCACTTCCTCCTCCCATTTGTCGTCACCGGCGCAACCGTTCTGCACCTACTATTTTTACACGAAACTGGGTCAAACAACCCAGCCGGGCTAAACTCCGACGCAGATAAAATTTCTTTCCACCCATACTTCTCATATAAAGATCTCCTCGGCTTTGTGATTATATTACTAGCTCTCACCTCCTTAGCATTATTTTCTCCCAACCTATTGGGTGACCCGGAGAACTTTACGCCGGCAAACCCACTCGTGACACCCCCACATATTCAGCCAGAATGATACTTCTTATTTGCCTACGCCATTCTCCGGTCCATCCCAAACAAACTAGGAGGGGTCCTCGCACTACTATTCAGCATTCTAGTGCTAATGGTTGTCCCAATCTTACACACGTCGAAACAACGAGGACTAACCTTCCGCCCCGTAACACAATTCTTATTCTGAACCCTGGTTGCAGATATATTTATCCTAACATGGATTGGAGGCATGCCCGTAGAACACCCATATATTATTATTGGCCAAATTGCATCCATTCTATACTTCGCGCTCTTCCTCGTTCTCGTCCCGCTGGCAGGATGAGTAGAGAATAAAGCACTGAAATGAGCCHM560167Rutilusrutilus                  ATGGCAAGCCTACGAAAAACCCATCCACTAATAAAAATCGCTAATGACGCGCTAGTCGACCTTCCGACACCATCTAACATCTCAGCACTATGAAACTTCGGGTCCCTGCTAGGGTTATGTTTAATTACCCAAATCCCGACAGGACTATTCTTAGCTATACACTATACCTCTGACATCTCAACCGCGTTTTCATCGGTGACCCACATCTGCCGAGACGTCAACTACGGCTGACTTATCCGAAACCTACATGCTAATGGAGCATCCTTCTTCTTCATCTGTCTTTATATACATATCGCACGAGGCCTATATTACGGGTCATACCTTTATAAGGAAACCTGAAACATTGGTGTGGTTCTATTCCTCCTGGTTATAATGACAGCCTTCGTTGGCTACGTACTACCATGGGGGCAAACATCCTTCTGAGGCGCCACCGTAATCACAAACCTCCTCTCAGCGGTCCCTTACATGGGAGATACCCTTGTTCAGTGAATCTGGGGAGGTTTCTCAGTAGATAACGCAACCCTTACACGGTTCTTCGCATTCCACTTTCTCTTTCCATTTGTCGTCGCCGGCGCAACCGTTCTACACTTACTGTTTTTACACGAGACGGGATCAAACAACCCGGCCGGACTAAACTCCGACGCAGATAAAATTTCTTTCCACCCGTATTTTTCATACAAAGACCTCCTTGGCTTTGTAATTATGTTACTAGCTCTCACCTCCCTAACATTATTTTCTCCTAACCTATTAGGTGACCCAGAAAACTTTACGCCAGCAAACCCACTCGTGACACCCCCACATATTCAGCCAGAATGATACTTCTTATTTGCCTACGCCATCCTCCGATCCATCCCGAACAAGCTAGGAGGGGTCCTCGCACTACTATTCAGCATTCTAGTGCTAATAGTTGTCCCAGTCTTACACACCTCAAAACAACGAGGACTAACTTTCCGCCCTGTGACACAATTTTTATTCTGAACCCTAGTTGCAGATATATTTATCTTAACATGAATCGGAGGCATGCCCGTAGAACACCCATATATCATTATTGGCCAAGTTGCATCCATTCTATACTTTGCACTTTTCCTCGTTCTTGTCCCGCTAGCAGGATGAGCGGAAAATAAAGCATTGAAATGAGCCHM560168Rutilusrutilus                  ATGGCAAGCCTACGAAAAACCCATCCACTAATAAAAATCGCTAATGACGCGCTAGTCGACCTTCCGACACCATCTAACATCTCAGCACTATGAAACTTCGGGTCCCTGCTAGGGTTATGTTTAATTACCCAAATCCTGACAGGACTATTCTTAGCTATACACTATACCTCTGACATCTCAACCGCGTTTTCATCGGTGACCCACATCTGCCGAGACGTCAACTACGGCTGACTTATCCGAAACCTACATGCTAATGGAGCATCCTTCTTCTTCATCTGTCTTTATATACATATCGCACGAGGCCTATACTACGGGTCATACCTTTATAAGGAAACCTGAAATATTGGTGTGGTTCTATTCCTCCTGGTTATAATGACAGCCTTCGTTGGCTACGTACTACCATGAGGGCAAATATCCTTCTGAGGCGCCACCGTAATCACAAACCTCCTCTCAGCGGTCCCTTACATGGGAGATACCCTTGTTCAGTGAATCTGAGGAGGTTTCTCAGTAGANNNNNNNNNNNNNNNNNNNNNNNNNNNNNNNNNNNNNNNNNNNNNNNNNNNNNNNNNNNNNNNNNNNNNNNNNNNNNNNNNNNNNNNNNNNNNNNNNNNNNNNNNNNNNNNNNNNNNNNNNNNNNNNNNNNNNNNNNNNNNNNNNNNNNNNNNNNNNNNNNNNNNNNNNNNNNNNNNNNNNNNNNNNNNNNNNNNNNNNNNNNNNNNNNNNNNNNNNNNNNNNNNNNNNNNNNNNNNNNNNNNNNNNNNNNNNNNNNNNNNNNNNNNNNNNNNNNNNNNNNNNNNNNNNNNNNNNNNNNNNNNNNNNNNNNNNNNNNNNNNNNNNNNNNNNNNNNNNNNNNNNNNNNNNNNNNNNNNNNNNNNNNNNNNNNNNNNNNNNNNNNNNNNNNNNNNNNNNNNNNNNNNNNNNNNNNNNNNNNNNNNNNNNNNNNNNNNNNNNNNNNNNNNNNNNNNNNNNNNNNNNNNNNNNNNNNNNNNNNNNNNNNNNNNNNNNNNNNNNNNNNNNNNNNNNNNNNNNNNNNNNNNNNNNNNNNNNNNNNNNNNNNNNNNNNNNNNNNNNNNNNNNNNNNNNNNNNNNNNNNNNNNNNNNNNNNNNNNNNNNNNNNNNNNNNNNNNNNNNNNNNNNNNNAF090773Rutilusylikiensis               atggcaagcctacgaaaaacccatccactaataaaaatcgctaatgacgcactagtcgacctcccaacaccatctaatatctcagcaatgtgaaacttcgggtccctgctaggattatgtttaattacccaaatcctaacgggattattcttagccatgcactatacctctgacatctcaaccgcattttcatcagtaacccacatctgccgagacgtcaactatggctgacttattcgaagcctgcacgccaatggagcatccttcttcttcatctgtctttatatgcatatcgcacgaggcctgtactatggatcatacctttataaggagacctgaaacattggtgtagtcctatttctcctagttataatgacagccttcgtcggctacgtgctcccatggggacaaatatcattctggggcgccactgtaatcacgaacctcctctcagcagtcccctatataggagacatactcgttcaatgaatctgaggcggcttcccggtagacaacgcaacccttacacgattcttcgcattccacttccttctaccatttgtcgtcgccggcgcaaccattctacacttactatttttgcacgaaacggggtcgaacaacccggccggactaaattccgacgcagataaaatttctttccacccatatttctcatataaagacctccttggctttgtaattatgttactagctctcacctccctagcattattttctcctaacctattaggtgacccagaaaactttacgccagcaaacccactcgtgacgcccccacatattcagccagaatgatacttcttatttgcctacgccattctccgatctatcccgaataaactaggaggggtactcgcactactattcagcattctagtgctaatagtcgtcccggtcttacatacctcaaagcagcgaggactaactttccgccctgtaacacaattcttattctgaaccctagttgcagacatgattatcttaacatgaattgggggcatgccggtagaacacccatatatcatcattggccaagtcgcatccatcctatacttcgcgctcttcctcatcctcgtcccgctagcagggtgaatggaaaataaagcattgaaatgagccFJ025070Rutilusylikiensis               ATGGCAAGCCTACGAAAAACCCATCCACTAATAAAAATCGCTAATGACGCACTAGTCGACCTCCCAACACCATCTAATATCTCAGCAATGTGAAACTTCGGGTCCCTGCTAGGATTATGTTTAATTACCCAAATCCTAACGGGATTATTCTTAGCCATGCACTATACCTCTGACATCTCAACCGCATTTTCATCAGTAACCCACATCTGCCGAGACGTCAACTATGGCTGACTTATTCGAAGCCTGCACGCCAATGGAGCATCCTTCTTCTTCATCTGTCTTTATATGCATATCGCACGAGGCCTGTACTATGGATCATACCTTTATAAGGAGACCTGAAACATTGGTGTAGTCCTATTTCTCCTAGTTATAATGACAGCCTTCGTCGGCTACGTGCTCCCATGGGGACAAATATCATTCTGGGGCGCCACTGTAATCACGAACCTCCTCTCAGCAGTCCCCTATATAGGAGACATACTCGTTCAATGAATCTGAGGCGGCTTCTCGGTAGACAACGCAACCCTTACACGATTCTTCGCATTCCACTTCCTTCTACCATTTGTCGTCGCCGGCGCAACCATTCTACACTTACTATTTTTGCACGAAACGGGGTCGAACAACCCGGCCGGACTAAATTCCGACGCAGATAAAATTTCTTTCCACCCATATTTCTCATATAAAGACCTCCTTGGCTTTGTAATTATGTTACTAGCTCTCACCTCCCTAGCATTATTTTCTCCTAACCTATTAGGTGACCCAGAAAACTTTACGCCAGCAAACCCACTCGTGACGCCCCCACATATTCAGCCAGAATGATACTTCTTATTTGCCTACGCCATTCTCCGATCTATCCCGAATAAACTAGGAGGGGTACTCGCACTACTATTCAGCATTCTAGTGCTAATAGTCGTCCCGGTCTTACATACCTCAAAGCAGCGAGGACTAACTTTCCGCCCTGTAACACAATTCTTATTCTGAACCCTAGTTGCAGACATGATTATCTTAACATGAATTGGGGGCATGCCGGTAGAACACCCATATATCATCATTGGCCAAGTCGCATCCATCCTATACTTCGCGCTCTTCCTCATCCTCGTCCCGCTAGCAGGGTGAATGGAAAATAAAGCATTGAAATGAGCCHM560169Scardiniusacarnanicus           ATGGCAAGCCTACGAAAAACCCACCCACTAATTAAAATCGCTAATGACGCACTAGTCGATCTCCCAACACCCTCTAATATCTCAGCGCTATGAAACTTCGGGTCCCTCCTAGGACTATGTTTGATTACTCAAATCCTAACAGGGCTGTTCCTAGCCATACACTACACCTCTGACATCTCAACCGCATTTTCATCAGTAACCCACATTTGCCGAGACGTTAACTACGGCTGACTCATCCGAAGCCTACATGCCAACGGAGCATCCTTCTTCTTCATCTGTCTTTATATACATATCGCACGAGGACTATATTATGGATCATACCTTTACAAAGAAACCTGAAATATCGGTGTAGTCCTATTTCTTTTAGTTATGATGACGGCCTTCGTCGGTTATGTACTTCCATGAGGACAAATGTCCTTTTGAGGTGCTACCGTAATTACAAACCTCCTCTCAGCAGTCCCCTACATGGGGGATACCCTTGTTCAATGAATCTGGGGCGGTTTCTCGGTGGACAACGCGACCCTAACACGATTCTTCGCATTCCACTTCCTCCTGCCATTCGTCGTCGCAGGCGCAACCATCCTACACTTACTATTTCTACACGAAACAGGGTCGAACAACCCGGCCGGACTAAATTCCGACGCGGACAAAATTTCTTTCCACCCGTACTTCTCATACAAAGACCTTCTTGGCTTCGTAATTATGCTGCTTGCCCTTACAGCTTTAGCACTATTCTCCCCAAACCTATTAGGGGACCCTGAGAATTTTACCCCAGCAAACCCACTCGTGACACCCCCACACATCCAGCCAGAATGATATTTCTTGTTTGCCTACGCCATCCTCCGATCTATCCCAAATAAACTAGGAGGGGTCCTTGCACTATTATTTAGCATCCTAGTGCTAATGGTTGTGCCAATTCTACACACCTCAAAGCAGCGAGGACTAACTTTCCGCCCCGTGACCCAATTTTTATTCTGGACCCTAGTTGCAGACATAATTATCTTAACATGAATTGGCGGCATACCCGTAGAACACCCATATATTGTTATTGGCCAAGTCGCATCCATTTTATACTTTGCACTTTTCCTTATTCTCATTCCACTAGCAGGATTAATGGAAAATAAAGCATTGAAATGAGCCHM560170Scardiniusacarnanicus           ATGGCAAGCCTACGAAAAACCCACCCACTAATTAAAATCGCTAATGACGCACTAGTCGATCTCCCAACACCCTCTAATATCTCAGCGCTATGAAACTTCGGGTCCCTCCTAGGACTATGTTTGATTACTCAAATCCTAACAGGGCTGTTCCTAGCCATACACTATACCTCTGACATCTCAACCGCATTTTCATCAGTAACCCACATTTGCCGAGACGTTAACTACGGCTGACTCATCCGAAGCCTACATGCCAACGGAGCATCCTTCTTCTTCATCTGTCTTTATATACATATCGCACGAGGACTATATTATGGATCATACCTTTACAAAGAAACCTGAAATATCGGTGTAGTCCTATTTCTTTTAGTTATGATGACGGCCTTCGTCGGTTATGTACTTCCATGAGGACAAATGTCCTTTTGAGGTGCTACCGTAATTACAAACCTCCTCTCAGCAGTCCCCTACATGGGGGATACCCTTGTTCAATGAATCTGGGGCGGTTTCTCGGTGGACAACGCGACCCTAACACGATTCTTCGCATTCCACTTCCTCCTGCCATTCGTCGTCGCAGGCGCAACCATCCTACACTTACTATTTCTACACGAAACAGGGTCGAACAACCCGGCCGGACTAAATTCCGACGCGGACAAAATTTCTTTCCACCCGTACTTCTCATACAAAGACCTTCTTGGCTTCGTAATTATGCTGCTTGCCCTTACAGCTTTAGCACTATTCTCCCCAAACCTATTAGGGGACCCTGAGAATTTTACCCCAGCAAACCCACTCGTGACACCCCCACACATCCAGCCAGAATGATATTTCTTGTTTGCCTACGCCATCCTCCGATCTATCCCAAATAAACTAGGAGGGGTCCTTGCACTATTATTTAGCATCCTAGTGCTAATGGTTGTGCCAATTCTACACACCTCAAAGCAGCGAGGACTAACTTTCCGCCCCGTGACCCAATTTTTATTCTGGACCCTAGTTGCAGACATAATTATCTTAACATGAATTGGCGGCATGCCCGTAGAACACCCATATATTGTTATTGGCCAAGTCGCATCCATTTTATACTTTGCACTTTTCCTTATTCTCATTCCACTAGCAGGATTAATGGAAAATAAAGCATTGAAATGAGCCHM560171Scardiniuserythrophthalmus      ATGGCAAGCCTACGAAAAACCCACCCGCTAATTAAAATCGCTAATGACGCACTAGTCGACCTCCCAACACCCTCTAATATTTCAGCGCTATGAAACTTCGGATCCCTCCTAGGACTATGTCTAATTACTCAAATCCTAACAGGGCTGTTTCTAGCCATGCGCTACACCTCTGACATCTCAACCGCATTTTCATCAGTAACCCATATTTGCCGAGACGTTAACTACGGCTGACTTATCCGAAGCCTACATGCCAACGGAGCATCCTTCTTCTTCATCTGTCTTTATATACATATCGCACGGGGACTATATTATGGGTCATACCTTTACAAAGAGACCTGAAATATTGGTGTAGTCCTATTTCTTTTGGTTATGATGACGGCCTTCGTCGGTTACGTACTTCCATGAGGGCAAATGTCCTTTTGAGGTGCTACCGTAATTACAAACCTCCTCTCAGCAGTCCCCTACATGGGTGATACCCTTGTTCAATGAATCTGAGGCGGTTTCTCAGTAGACAACGCGACCCTAACACGATTCTTCGCATTCCACTTCCTCCTGCCATTCGTCGTCGCAGGCGCAACCATCCTGCACTTGCTGTTTCTACACGAAACAGGATCGAACAACCCGGCCGGACTAAATTCCGACGCGGACAAAATTTCCTTCCACCCGTACTTCTCGTACAAGGACCTTCTTGGCTTCGTAATTATACTGCTTGCCCTTACAGCTTTGGCACTATTCTCCCCAAACCTTTTAGGGGACCCTGAGAATTTTACCCCAGCAAACCCACTCGTGACACCCCCACATATCCAGCCAGAGTGATATTTCTTATTTGCCTATGCCATCCTCCGATCTATCCCAAATAAGCTAGGAGGGGTTCTTGCACTATTATTTAGCATCCTAGTGCTAATAGTTGTGCCAATTCTACACACCTCAAAGCAGCGAGGACTAACTTTCCGCCCCGTGACTCAATTTTTATTCTGAACCCTGGTTGCAGACATGATTATCCTAACATGAATTGGGGGCATACCCGTAGAGCACCCATACATTGTTATTGGTCAGGTCGCATCCATCTTATACTTTGCACTCTTCCTTATTCTCATTCCACTAGCAGGATTAATGGAAAATAAAGCATTGAAATGAGCCY10444Scardiniuserythrophthalmus        atggcaagcctacgaaaaacccacccgctaattaaaatcgctaatgacgcactagtcgacctcccaacaccctctaatatttcagcgctatgaaacttcggatccctcctaggactatgtctaattactcaaatcctaacagggctgtttctagccatgcactacacctctgacatctcaaccgcattttcatcagtaacccatatttgccgagacgttaactacggctgacttatccgaagcctacatgccaacggagcatccttcttcttcatctgtctttatatacatatcgcacggggactatattatgggtcatacctttacaaagagacctgaaatattggtgtagtcctatttcttttggttatgatgacggccttcgtcggttacgtacttccatgagggcaaatgtccttttgaggtgctaccgtaattacaaacctcctctcagcagtcccctacatgggtgatacccttgttcaatgaatctgaggcggtttctcagtagacaacgcgaccctaacacgattcttcgcattccacttcctcctgccattcgtcgtcgcaggcgcaaccatcctgcacttgctgttcctacacgaaacaggatcgaacaacccggccggactaaattccgacgcggacaaaatttccttccacccgtacttctcgtacaaggacctccttggcttcgtaattatactgcttgcccttacagctttggcactattctccccaaaccttttaggggaccctgagaattttaccccagcaaacccactcgtgacacccccacatatccagccagagtgatatttcttatttgcctatgccatcctccgatctatcccaaataaactaggaggggttcttgcactattatttagcattctagtgctaatagttgtgccaattctacacacctcaaagcagcgaggactaactttccggcccgtgactcaatttttattctgaaccctggttgcagacatgattatcctaacatgaattgggggcatacccgtagagcacccatacattgttattggtcaggtcgcatccatcttatactttgcgctcttccttattctcattccactagcaggattaatggaaaataaagcattgaaataacccAY509848Scardiniuserythrophthalmus      atggcaagcctacgaaaaacccacccgctaattaaaatcgctaatgacgcactagtcgacctcccaacaccctctaatatttcagcgctatgaaacttcggatccctcctaggactatgtctaattactcaaatcctaacagggctgtttctagccatgcactacacctctgacatctcaaccgcattttcatcagtaacccatatttgccgagacgttaactacggctgacttatccgaagcctacatgccaacggagcatccttcttcttcatctgtctttatatacatatcgcacggggactatattatggatcatacctttacaaagagacctgaaatattggtgtagtcctatttcttttggttatgatgacggccttcgtcggttacgtacttccatgagggcaaatgtccttttgaggtgctaccgtaattacaaacctcctctcagcagtcccctacatgggtgatacccttgttcaatgaatctgaggcggtttctcagtagacaacgcgaccctaacacgattcttcgcattccacttcctcctgccattcgtcgtcgcaggcgcaaccatcctgcacttgctgtttctacacgaaacaggatcgaacaacccggccggactaaattccgacgcggacaaaatttccttccacccgtacttctcgtacaaggaccttcttggcttcgtaattatactgcttgcccttacagctttggcactattctccccaaaccttttaggggaccctgagaattttaccccagcaaacccactcgtgacacccccacatatccagccagagtgatatttcttatttgcctatgccatcctccgatctatcccaaataaactaggaggggttcttgcactattatttagcatcctagtgctaatagttgtgccaattctacacacctcaaagcagcgaggactaactttccgccccgtgactcaatttttattctgaaccctggttgcagacatgattatcctaacatgaattgggggcatacccgtagagcacccatacattgttattggtcaagtcgcatccatcttatactttgcactcttcctattccatgtcccaccaggagaatgggtggaaataaaagcattggaatggcccAY549459Scardiniusgraecus               atggcaagcctacgaaaaacccacccgctaattaaaatcgctaatgacgcactagtcgatctcccaacaccctctaatatttcagcgctatgaaacttcgggtccctcctaggactatgtttaattactcaaatcctaacagggctgtttctagccatgcactacacctctgacatctcaaccgcattttcatcagtaacccatatttgccgagacgttaactacggctgacttatccgaagcctacatgccaacggagcatccttcttcttcatctgtctttatatacatatcgcacgaggactatattatgggtcatacctttacaaagagacctgaaatattggtgtagtcctatttcttttagttatgataacggccttcgtcggttacgtacttccatgaggacaaatgtccttttgaggtgctaccgtaattacaaatctcctctcagcagtcccctacatgggagatacccttgttcagtgaatctgaggcggcttctcagtagacaacgcgaccctaacacgattcttcgcattccacttcctcctgccattcgtcgtcgcaggcgcaaccatcctacacttactgtttctacacgaaacaggatcgaacaacccagccggactaaattccgacgcggacaaaatttccttccacccgtacttctcatacaaggaccttcttggcttcgtaattatgctgcttgccctcacagccttagcactattctccccaaaccttttaggggaccctgagaattttaccccagcaaacccactcgtgacacccccacatatccagccagaatgatatttcttgtttgcctacgccatcctccgatctatcccaaataaactaggaggggtccttgcactattatttagcatcctagtgctaatggttgtgccaattctacacacctcaaagcagcgaggactaactttccgccccgtgactcaatttttattctgaaccctagttgcagacatgattatcctaacatgaatcgggggcatgcccgtagagcacccatacattgttattggccaagtcgcatccatcttatactttgcactcttccttattctcattccactagcaggattaatggaaaataaagcattgaaatgagccAY509832Scardiniusgraecus               atggcaagcctacgaaaaacccacccgctaattaaaatcgctaatgacgcactagtcgatctcccaacaccctctaatatttcagcgctatgaaacttcgggtccctcctaggactatgtttaattactcaaatcctaacagggctgtttctagccatgcactacacctctgacatctcaaccgcattttcatcagtaacccatatttgccgagacgttaactacggctgacttatccgaagcctacatgccaacggagcatccttcttcttcatctgtctttatatacatatcgcacgaggactatattatgggtcatacctttacaaagagacctgaaatattggtgtagtcctatttcttttagttatgataacggccttcgtcggttatgtacttccatgaggacaaatgtccttttgaggtgctaccgtaattacaaatctcctgtcagcagtcccctacatgggagatacccttgttcaaggaatctgaggcggttcttcagtagacaactcgaccctaacacgattcttcgcattccacttcctcctgccattcgtcgtcgcaggcgcaaccatcctgcacttgctgttcctacacgaaacaggatcgaacaacccggccggactaaattccgacgcggacaaaatttcctttcacccgtacttttcatacaaggaccttcttggcttcgtaattatgctgcttgccctcacagccttagcactattctccccaaaccttttaggggaccctgagaattttaccccagcaaacccactcgtgacacccccacatatccagccagaatgatatttcttgtttgcctacgccatcctccgatctatcccaaataaactaggaggggttcttgcactattatttagcatcctagtgctaatggttgtgccaattctacacacctcaaagcagcgaggactaaatttccgccccgtgactcaatttttattctgaaccctagttgcagacatgattatcctaacatgaatcgggggcatccccgtagagcacccatacattgttattggccaagtcgcatccatcttatactttgcactcttccttattctcattccactagcaggattaatggaaaataaagcattggaatgagccHM560172Scardiniushesperidicus          ATGGCAAGCCTACGAAAAACCCACCCGCTAATTAAAATCGCTAATGACGCACTAGTCGATCTCCCAACACCCTCTAATATTTCAGCGCTATGAAACTTCGGGTCCCTCCTAGGACTATGTTTAATTACTCAAATCCTAACAGGGCTGTTTCTAGCCATGCACTATACCTCTGACATCTCAACCGCATTTTCATCAGTAACCCATATTTGCCGAGACGTTAACTACGGCTGACTTATCCGAAGCCTACATGCCAACGGAGCATCCTTCTTCTTCATCTGCCTTTATATACATATCGCACGAGGACTATATTATGGATCATACCTTTACAAAGAGACCTGAAACATCGGTGTGGTCCTATTCCTTTTGGTTATGATGACGGCCTTCGTCGGTTACGTACTTCCATGAGGGCAAATGTCCTTTTGAGGTGCTACCGTAATTACAAACCTCCTCTCAGCAGTCCCCTACATAGGAGATACCCTTGTTCAGTGAATCTGAGGCGGTTTCTCAGTAGACAACGCGACCCTAACACGATTCTTCGCGTTCCACTTCCTCCTGCCGTTCGTCGTCGCAGGCGCAACCATCCTACACTTACTGTTTCTACACGAGACAGGATCAAACAACCCAGCCGGACTAAATTCCGACGCGGACAAAATTTCCTTCCACCCGTACTTCTCATATAAGGACCTTCTTGGCTTCGTAATTATGCTGCTTGCCCTTACAGCTTTGGCGCTATTTTCCCCAAACCTTTTAGGGGATCCTGAGAATTTTACTCCAGCAAACCCACTCGTGACACCCCCACACATCCAGCCAGAGTGATATTTCTTATTTGCCTACGCCATCCTCCGATCCATCCCAAATAAACTAGGAGGGGTTCTTGCACTATTATTTAGCATCCTAGTGCTAATAGTTGTGCCAATTCTACATACCTCAAAGCAGCGAGGACTAACTTTCCGCCCCGTGACTCAATTTTTATTCTGAACCCTGGTTGCAGACATAATTATCCTAACATGAATTGGGGGCATGCCCGTAGAGCACCCGTACATTGTTATTGGTCAAGTCGCATCCATCTTATACTTTGCACTCTTCCTTATTCTTATTCCACTAGCAGGATTAATGGGAAATAAAGCATTGAAATGAGCCHM560173Scardiniushesperidicus          ATGGCAAGCCTACGAAAAACCCACCCGCTAATTAAAATCGCTAATGACGCACTAGTCGATCTCCCAACACCCTCTAATATTTCAGCGCTATGAAACTTCGGGTCCCTCCTAGGACTATGTTTAATTACTCAAATCCTAACAGGGCTGTTTCTAGCCATGCACTATACCTCTGACATCTCAACCGCATTTTCATCAGTAACCCATATTTGCCGAGACGTTAACTACGGCTGACTTATCCGAAGCCTACATGCCAACGGAGCATCCTTCTTCTTCATCTGTCTTTATATACATATCGCACGAGGACTATATTATGGATCATACCTTTACAAAGAAACCTGAAATATTGGTGTAGTCCTATTCCTTTTGGTTATGATAACGGCCTTCGTCGGTTACGTACTTCCATGAGGGCAAATGTCCTTTTGAGGTGCTACCGTAATTACAAACCTCCTCTCAGCAGTCCCCTACATAGGAGATACCCTTGTTCAGTGAATCTGAGGCGGTTTCTCAGTAGACAACGCGACCCTAACACGATTCTTCGCGTTCCACTTCCTACTGCCGTTCGTCGTCGCAGGCGCAACCATCCTACACTTACTGTTTCTACACGAGACAGGATCAAACAACCCAGCCGGACTAAATTCCGACGCGGACAAAATTTCCTTCCACCCGTACTTCTCATACAAGGACCTTCTTGGCTTCGTAATTATGCTGCTTGCCCTTACAGCTTTGGCACTATTTTCCCCAAACCTTTTAGGGGACCCTGAGAATTTTACTCCAGCAAACCCACTCGTGACACCCCCACACATCCAGCCAGAGTGATATTTCTTATTTGCCTACGCCATCCTCCGATCCATCCCAAATAAACTAGGAGGGGTTCTTGCACTATTATTTAGCATCCTAGTGCTAATAGTTGTGCCAATTCTACATACCTCAAAGCAGCGAGGACTAACTTTCCGCCCCGTGACTCAATTTTTATTCTGAACCCTGGTTGCAGACATAATTATCCTAACATGAATTGGGGGCATGCCCGTAGAGCACCCGTACATTGTTATTGGTCAAGTCGCATCCATCTTATACTTTGCACTCTTCCTTATTCTTATTCCACTAGCAGGATTAATGGAAAATAAAGCATTGAAATGAGCCHM560174Scardiniushesperidicus          ATGGCAAGCCTACGAAAAACCCACCCGCTAATTAAAATCGCTAATGACGCACTAGTCGATCTCCCAACACCCTCTAATATTTCAGCGCTATGAAACTTCGGGTCCCTCCTAGGACTATGTTTAATTACTCAAATCCTAACAGGGCTGTTTCTAGCCATGCACTATACCTCTGACATCTCAACCGCATTTTCATCAGTAACCCATATTTGCCGAGACGTTAACTACGGCTGACTTATCCGAAGCCTACATGCCAACGGAGCATCCTTCTTCTTCATCTGTCTTTATATACATATCGCACGAGGACTATATTATGGATCATACCTTTACAAAGAGACCTGAAATATTGGTGTAGTCCTATTCCTTTTGGTTATGATGACGGCCTTCGTCGGTTACGTACTTCCATGAGGGCAAATGTCCTTTTGAGGTGCTACCGTAATTACAAACCTCCTCTCAGCAGTCCCCTACATAGGAGATACCCTTGTTCAGTGAATCTGAGGCGGTTTCTCAGTAGACAACGCGACCCTAACACGATTCTTCGCGTTCCACTTCCTCCTGCCGTTCGTCGTCGCAGGCGCAACCATCCTACACTTACTGTTTCTACACGAGACAGGATCAAACAACCCAGCCGGACTAAATTCCGACGCGGACAAAATTTCCTTCCACCCGTACTTCTCATATAAGGACCTTCTTGGCTTCGTAATTATGCTGCTTGCCCTTACAGCTTTGGCACTATTTTCCCCAAACCTTTTAGGGGACCCCGAGAATTTTACTCCAGCAAACCCACTCGTGACACCCCCACACATCCAGCCAGAGTGATATTTCTTATTTGCCTACGCCATCCTCCGATCCATCCCAAATAAACTAGGAGGGGTTCTTGCACTATTATTTAGCATCCTAGTGCTAATAGTTGTGCCAATTCTACATACCTCAAAGCAGCGAGGACTAACTTTCCGCCCCGTGACTCAATTTTTATTCTGAACCCTGGTTGCAGACATAATTATCCTAACATGAATTGGGGGCATGCCCGTAGAGCACCCGTACATTGTTATTGGTCAAGTCGCATCCATCTTATACTTTGCACTCTTCCTTATTCTTATTCCACTAGCAGGATTAATGGAAAATAAAGCATTGAAATGAGCCAY509847Scardiniushesperidicus          atggcaagcctacgaaaaacccacccgctaattaaaatcgctaatgacgcactagtcgatctcccaacaccctctaatatttcagcgctatgaaacttcgggtccctcctaggactatgtttaattactcaaatcctaacagggctgtttctagccatgcactatacctctgacatctcaaccgcattttcatcagtaacccatatttgccgagacgttaactacggctgacttatccgaagcctacatgccaacggagcatccttcttcttcatctgtctttatatacatatcgcacgaggactatattatggatcatacctttacaaagagacctgaaatattggtgtagtcctaatccttttggttatgatgacggccttcgtcggttacgtacttccatgagggcaaatgtccttttgaggtgctgccgtaattacaaacctcctctcagcagtcccctacataggagatacccttgttcagtgaatctgaggcggtttctcagtagacaacgcgaccctaacacgattcttcgcattccacttcctcctgccattcgtcgtcgcaggcgcgatcatcctacacttactgtttctacacgagacaggatcaaacagcccagccggactaaattccgacgcggacaaaatttccttccacccgtacttctgatataaggaccttcttggcttcgtaattatgctgcttgcccttacagctttggcactatttttcccaaaccttttaggggatcctgagaattttactccagcaaacccactcgtgacacccccacacatccagccagagtgatatttcttatttgcctacgccatcctccgatctatcccaaataaactatgaggggctcttgcactattatttatcatcctagtgctaatagttgtgccaattctacatacctcaaagcagcgaggactaactttccgccccgtgactcaatttttattctgaaccctggttgcagacatgattatcctaacatgaattgggggcatccccgtagagcacccgtacattgttattggtcaagtcgcatccatcttatactttgcactcttccttattcttattccactagcaggattaatggaaaataaagcattgcaatgacccAY509846Scardiniushesperidicus          atggcaagcctacgaaaaacccacccgctaattaaaatcgctaatgacgcactagtcgatctcccaacaccctctaatatttcagcgctatgaaacttcgggtccctcctaggactatgtttaattactcaaatcctaacagggctgtttctagccatgcactatacctctgacatctcaaccgcattttcatcagtaacccatatttgccgagacgttaactacggctgacttatccgaagcctacatgccaacggagcatccttcttcttcatctgtctttatatacatatcgcacgaggactatattatggatcatacctttacaaagagacctgaaatattggtgtagtcctaatccttttggttatgatgacggccttcgtcggttacgtacttccatgagggcaaatgtccttttgaggtgctgccgtaattacaaacctcctctcagcagtcccctacataggagatacccttgttcagtgaatctgaggcggtttctcagtagacaacgcgaccctaacacgattcttcgcattccacttcctcctgccattcgtcgtcgcaggcgcgatcatcctacacttactgtttctacacgagacaggatcaaacagcccagccggactaaattccgacgcggacaaaatttccttccacccgtacttctgatataaggaccttcttggcttcgtaattatgctgcttgcccttacagctttggcactatttttcccaaaccttttaggggatcctgagaattttactccagcaaacccactcgtgacacccccacacatccagccagagtgatatttcttatttgcctacgccatcctccgatctatcccaaataaactatgaggggctcttgcactattatttatcatcctagtgctaatagttgtgccaattctacatacctcaaagcagcgaggactaactttccgccccgtgactcaatttttattctgaaccctggttgcagacatgattatcctaacatgaattgggggcatccccgtagagcacccgtacattgttattggtcaagtcgcatccatcttatactttgcactcttccttattcttattccactagcaggattaatggaaaataaagcattgcaatgacccHM560175Scardiniusplotizza              ATGGCAAGCCTACGAAAAACCCACCCGCTAATTAAAATCGCTAATGACGCACTAGTCGATCTCCCAACACCCTCTAATATTTCAGCGCTATGAAACTTCGGGTCCCTCCTAGGACTATGTTTAATTACTCAAATCCTAACAGGGCTGTTTCTAGCCATGCACTATACCTCTGACATCTCAACCGCATTTTCATCAGTAACCCATATTTGCCGAGACGTTAACTACGGCTGACTTATCCGAAGCCTACATGCCAACGGAGCATCCTTCTTCTTCATCTGTCTTTATATACATATCGCACGAGGACTATATTATGGATCATACCTTTACAAGGAGACCTGAAATATTGGTGTAGTCCTATTCCTTTTGGTTATGATGACGGCTTTCGTCGGTTACGTACTTCCATGAGGGCAAATGTCCTTTTGAGGTGCTACCGTAATTACAAACCTCCTCTCAGCAGTCCCCTACATAGGAGACACCCTTGTTCAATGAATCTGAGGCGGTTTCTCAGTAGACAACGCGACCCTAACACGATTCTTCGCATTCCACTTCCTCCTGCCGTTCGTCGTCGCAGGCGCAACCATCCTACACTTACTGTTTCTACACGAGACAGGATCAAACAACCCAGCCGGACTAAATTCCGACGCGGACAAAATTTCCTTCCACCCGTACTTCTCATACAAGGACCTTCTTGGCTTCGTAATTATGCTGCTTGCCCTTACAGCTTTGGCACTATTCTCCCCAAACCTTTTAGGGGACCCTGAAAATTTTACCCCAGCAAACCCACTCGTGACACCCCCACACATCCAGCCAGAGTGATATTTCTTATTTGCCTACGCCATCCTCCGATCCATCCCAAATAAACTAGGAGGGGTTCTTGCACTATTATTTAGCATCCTAGTGCTAATAGTTGTGCCAATTCTACATACCTCAAAGCAGCGAGGACTAACTTTCCGCCCCGTGACTCAATTTTTATTCTGAACCCTGGTTGCAGACATAATTATCCTAACATGAATTGGGGGCATGCCCGTAGAGCACCCGTACATTGTTATTGGTCAAGTCGCATCCATCTTATACTTTGCACTCTTCCTTATTCTCATTCCACTAGCAGGATTAATGGAAAATAAAGCATTGAAATGAGCCHM560176Scardiniusplotizza              ATGGCAGGCCTACGAAAAACCCACCCGCTAATTAAAATCGCTAATGACGCACTAGTCGATCTCCCAACACCCTCTAATATTTCAGCGCTATGAAACTTCGGGTCCCTCCTAGGACTATGTTTAATTACTCAAATCCTAACAGGGCTGTTTCTAGCCATGCACTATACCTCTGACATCTCAACCGCATTTTCATCAGTAACCCATATTTGCCGAGACGTTAACTACGGCTGACTTATCCGAAGCCTACATGCCAACGGAGCATCCTTCTTCTTCATCTGTCTTTATATACATATCGCACGAGGACTATATTATGGATCATACCTTTACAAGGAGACCTGAAATATTGGTGTAGTCCTATTCCTTTTAGTTATGATGACGGCTTTCGTCGGTTACGTACTTCCATGAGGGCAAATGTCCTTTTGAGGTGCTACCGTAATTACAAACCTCCTCTCAGCAGTCCCTTACATAGGAGATACCCTTGTTCAATGAATCTGAGGCGGTTTCTCAGTAGACAACGCGACCCTAACACGATTCTTCGCATTCCACTTCCTCCTGCCGTTCGTCGTCGCAGGCGCAACCATCCTACACTTACTGTTTCTACACGAGACAGGATCAAACAACCCAGCCGGACTAAATTCCGACGCGGACAAAATTTCCTTCCACCCGTACTTCTCATACAAGGACCTTCTTGGCTTCGTAATTATGCTGCTTGCCCTTACAGCTTTGGCACTATTCTCCCCAAACCTTTTAGGGGACCCTGAAAATTTTACCCCAGCAAACCCACTCGTGACACCCCCACACATCCAGCCAGAGTGATATTTCTTATTTGCCTACGCCATCCTCCGATCCATCCCAAATAAACTAGGAGGGGTTCTTGCACTATTATTTAGCATCCTAGTGCTAATAGTTGTGCCAATTCTACATACCTCAAAGCAGCGAGGACTAACTTTCCGCCCCGTGACTCAATTTTTATTCTGAACCCTGGTTGCAGACATAATTATCCTAACATGAATTGGGGGCATGCCCGTAGAGCACCCGTACATTGTTATTGGTCAAGTCGCATCCATCTTATACTTTGCACTCTTCCTTATTCTCATTCCACTAGCAGGATTAATGGAAAATAAAGCATTGAAATGAGCCAY509833Scardiniusscardafa              atggcaagcctacgaaaaacccacccgctaattaaaatcgctaatgacgcactagtcgatctcccaacaccctctaatatttcagcgctatgaaacttcgggtccctcctaggactatgtttaattactcaaatcctaacagggctgtttctagccatgcactatacctctgacatctcaaccgcattttcatcagtaacccatatttgccgagacgttaactacggctgacttatccgaagcctacatgccaacggagcatccttcttcttcatctgtctttatatacatatcgcacgaggactatattatggatcatacctttacaaagagacctgaaatattggtgtaggcctattccttttggttatgatgacggccttcgcacgtcacgacctagcatgagggcaaatgtccttttgaggtgctcccgtaattaccaacctcttttcagcagtcctctacaagggagatacccttgttcaatgaatctgaggcggttttacagtagacaacgcgaccctaacacgattcttcgcattccacttcctcctgccattcgtcgtcgcaggcgcgaccatcctacacttactgtttctacacgagacaggatcaaacaacccagccggactaaattccgacgcggacaaaatttccttccacccgtacttctgatacaatgaccttcttggcttcataattatgctgcttgcccttacagctttggcactatttttcccaaaccttttaggggaccctgagaattttactccaccaaacccactcgtgacacccccacacatgcagccagagtgatatttcttatttgcctacgccatcctccgatccatcccaaataaactatgaggggttcttgcactattatttagcattctagtgctaataattgtgccaattctacatacctcaaagcattgaggactaactttccgccccgtgactcaatttttattctgaaccctggttgcacacatgattatcctaacatgaattgggggcatgcccgtagagcacccgtacattggtattggtcaagtcgcatccatcttgtactttgcactcttccttattcttatttcactagcaggattaatggaaaataaagcattgaaatgagccAY549460Squaliusalbus                   atggcaagcctacgaaaaacccacccactaataaaaatcgccaatggcgcactagtcgaccttccgacaccatctaatatctcagcactatgaaacttcgggtctctcctgggattatgtttaattacccaaatcctaacaggactattcctagcaatacactacacctccgacatctcaaccgcattttcatcggtaacccatatctgccgagatgttaactacggctgacttattcgaagtctacatgccaatggagcctccttcttcttcatctgtctttacatacacattgcacgaggactatattatggctcgtacctatacaaggaaacctgaaacattggtgtagtcctatttctcctagttataatgacagcctttgtcggttacgtacttccatgaggacaaatgtccttttgaggtgccacggtaattacaaacctcctctcagcagtcccttacataggggacacgcttgttcaatgaatttgaggcggattctccgtagataacgcaaccctcacccgattcttcgcgttccacttcctcctgccatttgtcgtcgcaggtgcaaccgtcctacacctgctatttctacatgaaacaggatcaaacaacccagccggactaaattccgacgccgacaaaatttctttccacccgtacttctcatataaagaccttcttggcttcgttatcatattattagcccttacctccttagcattattttctcctaacctactaggtgacccagaaaactttaccccagcaaacccactggtgacgcccccacatattcaaccagaatgatatttcttatttgcctacgccattctccgatctattccaaacaaactaggaggggtccttgcactactattcagcatcctagtgctgatagttgtgccaatcttacacacctctaaacaacgaggactaactttccgccccgtaactcaatttctattttgaaccctagttgcagatatacttatcctgacatgaattgggggtatacctgtagaacacccatatatcatcattggccaagtcgcatccattctatattttgcgctcttcctcgttcttgtcccactagcaggatgagtggaaaataaagcactgaaatgagccHM560177Petroleuciscusaphipsi           ATGGCAAGCCTACGAAAAACCCACCCCCTAATAAAAATCGCCAATGGCGCACTAGTCGACCTTCCAACACCATCTAATATTTCAGCACTATGAAACTTCGGGTCCCTCCTAGGATTGTGTTTAATTACCCAAATCCTCACGGGATTATTTTTAGCAATGCACTACACCTCCGACATCTCAACCGCATTTTCATCAGTAACTCACATCTGCCGGGATGTTAACTACGGCTGACTTATTCGAAATCTGCATGCCAACGGAGCATCCTTCTTCTTCATCTGCCTTTACATACACATTGCGCGAGGCCTGTATTATGGATCATACCTTTATAAAGAAACCTGAAACATTGGTGTGGTCCTATTTCTTCTGGTTATAATGACAGCCTTCGTCGGCTACGTACTTCCATGGGGACAAATGTCCTTTTGGGGTGCCACAGTAATTACAAATCTACTCTCCGCAGTCCCTTACATAGGGGATACCCTTGTTCAGTGAATCTGAGGCGGTTTCTCCGTAGACAACGCAACACTCACACGATTCTTCGCGTTCCACTTCCTCCTGCCATTTGTCGTCGCAGGCGCAACCATCCTACATCTTCTATTTTTACACGAAACAGGATCAAACAACCCGGCCGGATTGAATTCGGACGCGGACAAAATTTCCTTCCATCCATACTTCTCATACAAGGACCTTCTTGGCTTTGTTATTATGCTACTAGCCCTCACCTCTCTAGCATTATTTTCTCCTAACCTATTAGGTGACCCAGAGAACTTTACCCCAGCGAACCCGCTAGTAACGCCTCCACACATCCAGCCAGAATGATACTTCCTATTTGCCTATGCCATCCTCCGATCTATTCCAAATAAACTAGGAGGGGTCCTTGCACTACTATTCAGCATCCTAGTACTAATAGTTGTGCCAATTCTACACACGTCAAAACAACGAGGACTAACTTTCCGCCCTGTGACTCAATTCCTATTCTGAACCCTAGTTGCAGATATATTTATCTTGACATGAATTGGGGGCATGCCCGTAGAACACCCATATATTATCATTGGCCAAGTCGCATCCGTTTTATACTTTGCACTCTTCCTCATTCTTGTCCCACTAGCAGGATGAGTAGAAAATAAAGCATTGAAATGAGCCHM560178Petroleuciscusaphipsi           ATGGCAAGCCTACGAAAAACCCACCCCTTAATAAAAATCGCCAATGGCGCACTAGTCGACCTTCCAACACCATCTAATATTTCAGCACTATGAAACTTCGGGTCCCTCCTAGGATTGTGTTTAATTACCCAAATCCTCACGGGATTATTTTTAGCAATGCACTACACCTCCGACATCTCAACCGCATTTTCATCAGTAACTCACATCTGCCGGGATGTTAACTACGGCTGACTTATTCGAAATCTGCATGCCAACGGAGCATCCTTCTTCTTCATCTGCCTTTACATACACATTGCGCGAGGCCTGTATTATGGATCATACCTTTATAAAGAAACCTGAAACATTGGTGTGGTCCTATTTCTTCTGGTTATAATGACAGCCTTCGTCGGCTACGTACTTCCATGGGGACAAATGTCCTTTTGGGGTGCCACAGTAATTACAAATCTACTCTCCGCAGTCCCTTACATAGGGGATACCCTTGTTCAGTGAATCTGAGGCGGTTTCTCCGTAGACAACGCAACACTCACACGATTCTTCGCGTTCCACTTCCTCCTGCCATTTGTCGTCGCAGGCGCAACCATCCTACATCTTCTATTTTTACACGAAACAGGATCAAACAACCCGGCCGGATTGAATTCGGACGCGGACAAAATTTCCTTCCATCCATACTTCTCATACAAGGACCTTCTTGGCTTTGTTATTATGCTACTAGCCCTCACCTCTCTAGCATTATTTTCTCCTAACCTATTAGGTGACCCAGAGAACTTTACCCCAGCGAACCCGCTAGTAACGCCTCCACACATCCAGCCAGAATGATACTTCCTATTTGCCTATGCCATCCTCCGATCTATTCCAAATAAACTAGGAGGGGTCCTTGCACTACTATTCAGCATCCTAGTACTAATAGTTGTGCCAATTCTACACACGTCAAAACAACGAGGACTAACTTTCCGCCCTGTGACTCAATTCCTATTCTGAACCCTAGTTGCAGATATATTTATCTTGACATGAATTGGGGGCATGCCCGTAGAACACCCATATATTATCATTGGCCAAATCGCATCCGTTTTATACTTTGCACTCTTCCTCATTCTTGTCCCACTAGCAGGATGAGTAGAAAATAAAGCATTGAAATGAGCCHM560179Squaliusaradensis               ATGGCAAGCCTACGAAAAACCCACCCACTGATAAAAATCGCCAACGGCGCACTAGTTGACCTTCCAACACCATCTAATATCTCAGCACTATGAAATTTCGGGTCCCTCCTAGGGCTGTGTTTAATTACCCAAATCCTCACGGGACTATTCCTAGCAATACATTACACCTCCGACATCTCAACCGCATTTTCATCTGTAACCCATATCTGCCGAGATGTAAACTACGGCTGACTTATTCGAAGTCTGCATGCCAACGGAGCATCCTTCTTTTTTATTTGCCTTTATATACACATTGCACGAGGCCTATATTATGGCTCGTACCTATATAAAGAAACCTGAAACATCGGGGTAGTACTATTTCTCCTAGTTATAATAACAGCCTTCGTCGGCTACGTACTTCCGTGAGGACAAATGTCCTTTTGAGGTGCCACAGTAATTACTAACCTCCTATCAGCAGTCCCTTACATAGGGGATACCCTTGTTCAATGAATCTGGGGCGGTTTTTCCGTAGACAACGCGACACTTACCCGATTCTTCGCGTTCCACTTCCTCCTGCCATTTGTCGTCGCAGGCGCGACCATCCTGCACCTATTATTCCTACACGAAACAGGATCAAACAACCCAGCCGGCCTAAATTCCGACGCCGACAAAATTTCTTTCCACCCATACTTCTCATATAAGGACCTTCTTGGCTTTGTTATTATGTTGCTAGCCCTCACCTCTCTGACGCTATTTTCCCCCAATCTGCTTGGTGACCCAGAGAACTTTACCCCAGCGAACCCCCTAGTAACACCCCCACATATTCAGCCAGAGTGGTACTTCCTATTTGCCTATGCCATCCTCCGATCTATTCCAAACAAACTGGGAGGGGTCCTCGCACTACTATTCAGCATCCTAGTACTAATGGTTGTACCAATTTTACACACCTCGAAGCAACGAGGACTAACTTTCCGCCCCGTGACACAATTCTTATTCTGGACCCTGGTTGCAGATATATTTATCCTAACATGAATTGGGGGCATACCTGTAGAGCATCCGTACATTGTCATTGGCCAAATCGCATCCGTCCTATATTTTGCACTATTCCTCATTCTTGTCCCACTAGCAGGATGAGCGGAGAATAAAGCATTGAAATGAGCTHM560180Squaliusaradensis               ATGGCAAGCCTACGAAAAACCCACCCACTGATAAAAATCGCCAACGGCGCACTAGTTGACCTTCCAACACCATCTAATATCTCAGCACTATGAAATTTCGGGTCCCTCCTAGGGCTGTGTTTAATTACCCAAATCCTCACGGGACTATTCCTAGCAATACATTACACCTCCGACATCTCAACCGCATTTTCATCTGTAACCCATATCTGCCGAGATGTAAACTACGGCTGACTTATTCGAAGTCTGCATGCCAACGGAGCATCCTTCTTTTTTATTTGCCTTTATATACACATTGCACGAGGCCTATATTATGGCTCGTACCTATATAAAGAAACCTGAAACATCGGGGTAGTACTATTTCTCCTAGTTATAATAACAGCCTTCGTCGGCTACGTACTTCCGTGAGGACAAATGTCCTTTTGAGGTGCCACAGTAATTACTAACCTCCTATCAGCAGTCCCTTACATAGGGGATACCCTTGTTCAATGAATCTGGGGCGGTTTTTCCGTAGACAACGCGACACTTACCCGATTCTTCGCGTTCCACTTCCTCCTGCCATTTGTCGTCGCAGGCGCGACCATCCTGCACCTATTATTCCTACACGAAACAGGATCAAACAACCCAGCCGGCCTAAATTCCGACGCCGACAAAATTTCTTTCCACCCATACTTCTCATATAAGGACCTTCTTGGCTTTGTTATTATGTTGCTAGCCCTCACCTCTCTGACGCTATTTTCCCCCAATCTGCTTGGTGACCCAGAGAACTTTACCCCAGCGAACCCCCTAGTAACACCCCCACATATTCAGCCAGAGTGGTACTTCCTATTTGCCTATGCCATCCTCCGATCTATTCCAAACAAACTGGGAGGGGTCCTCGCACTACTATTCAGCATCCTAGTACTAATGGTTGTACCAATTTTACACACCTCGAAGCAACGAGGACTAACTTTCCGCCCCGTGACACAATTCTTATTCTGGACCCTGGTTGCAGATATATTTATCCTAACATGAATTGGGGGCATACCTGTAGAGCATCCGTACATTGTCATTGGCCAAATCGCATCCGTCCTATATTTTGCACTATTCCTCATTCTTGTCCCACTAGCAGGATGAGCGGAGAATAAAGCATTGAAATGAGCTHM560181Squaliuscarolitertii            ATGGCAAGCCTACGAAAGACCCACCCACTAATAAAAATCGCCAACGGCGCACTAGTCGACCTTCCAACACCCTCTAATATCTCAGCACTATGAAACTTCGGGTCCCTCCTGGGACTATGCTTAATTACCCAAATCCTGACCGGGCTATTCTTAGCGATACACTACACCTCCGACATCTCGACCGCATTTTCATCTGTAACCCATATCTGCCGAGATGTTAACTACGGCTGACTTATCCGAAGTCTGCATGCCAATGGAGCATCCTTCTTCTTTATCTGTCTTTATATACATATTGCGCGGGGCCTGTATTATGGCTCGTACCTGTATAAAGAAACCTGAAACATCGGTGTAGTCCTGTTTCTCCTAGTTATAATGACGGCCTTCGTCGGCTACGTACTTCCATGGGGACAAATATCCTTTTGAGGTGCCACAGTAATCACCAATCTCCTGTCAGCAGTCCCCTACATAGGAGATACCCTTGTTCAATGAATCTGGGGTGGTTTCTCTGTAGACAACGCAACGCTCACCCGATTCTTCGCATTCCACTTCCTCCTCCCATTTGTCGTTGCAGGCGCAACCATCTTACACCTGTTATTTCTACACGAAACAGGATCAAACAACCCGGCCGGGCTGAATTCCGACGCCGACAAAATTTCCTTCCATCCATACTTCTCATATAAAGACCTTCTTGGCTTTGTTATCATATTGCTAGCCCTCACCTCTCTAGCGTTGTTTTCCCCCAACCTACTAGGTGACCCAGAAAACTTTACCCCAGCGAACCCGCTAGTAACACCCCCACATATTCAGCCAGAATGATACTTCTTGTTTGCCTACGCCATCCTACGATCTATCCCAAATAAACTAGGAGGGGTCCTTGCACTACTATTCAGCATCCTGGTGCTAATAGTTGTGCCAATTTTACACACCTCGAAACAACGAGGACTAACTTTCCGCCCCGCAACTCAATTCCTATTCTGAACCCTAGTCGCAGATATACTCATCCTGACATGAATTGGAGGCATACCCGTAGAACATCCATACATTGTCATTGGCCAAATCGCATCCATTCTATACTTTGCGCTATTCCTCGTTCTTGTCCCACTAGCAGGGTGAATGGAAAATAAAGCATTAAAATGAGCTHM560182Squaliuscarolitertii            ATGGCAAGCCTACGAAAAACCCACCCACTAATAAAAATCGCCAACGGCGCACTAGTCGACCTTCCAACACCCTCTAATATCTCAGCACTATGAAACTTCGGGTCCCTCCTGGGACTATGCTTAATTACCCAAATCCTGACCGGGCTATTCTTAGCGATACATTACACCTCCGACATCTCGACCGCATTTTCATCTGTAACCCATATCTGCCGAGATGTTAACTACGGCTGACTTATCCGAAGTCTGCATGCCAATGAAGCATCCTTCTTCTTTATCTGTCTTTATATACATATTGCGCGGGGCCTGTATTATGGCTCGTACCTGTATAAAGAAACCCGAAACATCGGTGTAGTCCTGTTTCTCCTAGTTATAATGACGGCCTTCGTCGGCTACGTACTTCCATGGGGACAAATATCCTTTTGAGGTGCCACAGTAATTACCAATCTCCTGTCAGCAGTCCCCTACATAGGAGATACCCTTGTTCAATGAATCTGGGGTGGTTTCTCTGTAGACAACGCAACGCTCACCCGATTCTTCGCATTCCACTTCCTCCTCCCATTTGTCGTTGCAGGCGCAACCATCTTACACCTGTTATTTCTACACGAAACAGGATCAAACAACCCGGCCGGGCTGAATTCCGACGCCGACAAAATTTCCTTCCATCCATACTTCTCATATAAAGACCTTCTTGGCTTTGTTATCATATTGCTAGCCCTCACCTCTCTAGCGTTGTTTTCCCCCAACCTACTAGGTGACCCAGAAAACTTTACCCCAGCGAACCCGCTAGTAACACCCCCACATATTCAGCCAGAATGATACTTCTTGTTTGCCTACGCCATCCTACGATCTATCCCAAATAAACTAGGAGGGGTCCTTGCACTACTATTCAGCATCCTGGTGCTAATAGTTGTGCCAATTTTACACACCTCGAAACAACGAGGACTAACTTTCCGCCCCGCAACTCAATTCCTATTCTGAACCCTAGTCGCAGATATACTCATCCTGACATGAATTGGAGGCATACCCGTAGAACATCCATACATTGTCATTGGCCAAGTCGCATCCATTCTATACTTTGCGCTATTCCTCGTTCTTGTCCCACTAGCAGGGTGAATGGAAAATAAAGCATTAAAATGAGCTDQ521423Squaliuscastellanus             ATGGCAAGCCTACGAAAAACCCACCCACTAATAAAAATCGCCAACGGCGCACTAGTTGACCTTCCAACACCCTCTAATATCTCAGCACTATGAAACTTCGGCTCCCTCCTGGGACTATGCTTAATTACCCAAATCCTGACCGGGCTATTCTTAGCAATACACTACACCTCCGACATCTCTACCGCATTTTCATCTGTAACCCATATCTGCCGGGATGTTAACTACGGCTGACTTATCCGAAGTCTACATGCCAATGGAGCATCCTTCTTCTTTATCTGTCTTTATATACATATTGCGCGGGGTCTATATTATGGCTCATACCTGTATAAAGAGACCTGAAACATCGGTGTAGTCCTGTTTCTCCTAGTTATGATGACGGCCTTCGTCGGCTACGTACTTCCATGAGGACAAATGTCCTTTTGAGGTGCCACAGTGATTACCAATCTCCTATCAGCAGTCCCCTATATAGGAGATACCCTTGTTCAATGAATCTGAGGGGGTTTTTCCGTAGACAACGCAACGCTCACCCGATTCTTCGCATTCCACTTCCTCCTTCCATTTGTCGGTGCAGGCGCAACCATCTTACACCTGTTATTTCTACACGAAACAGGATCAACCAACCCGGCCGGGCTCAATTCCGACGCCGACAAAATTTCCTTCCACCCGTACTTCTCATATAAAGACCTTCTTGGCTTTGTTATCATATTGCTAGCCCTCACCTCCCTAGCGTTGTTTTCCCCCAACCTATTAGGTGATCCAGAAAACTTTACCCCAGCGAACCCGCTAGTAACGCCCCCACATATTCAGCCAGAATGATACTTCTTGTTTGCCTACGCCATCCTACGATCTATCCCAAATAAATTAGGAGGGGTCCTTGCGCTACTATTCAGCATCCTAGTGCTAATGGTTGTGCCAATTTTACACACCTCAAAACAACGGGGACTAACTTTCCGCCCCGCAACTCAGTTCCTATTCTGAACCCTAGTCGCAGATATATTCATCCTGACATGAATTGGGGGCATACCCGTAGAACATCCATACATTGTCATTGGCCAAGTCGCATCCATTCTATACTTTGCGCTATTCCTAGTTCTTGTCCCGCTAGCAGGGTGAATGGAAAATAAAGCATTGAAATGAGCT                  AJ252807Squaliuscephalus                atggcaagcctacgaaaaacccacccactaatgaaaatcgccaatggcgcactagtcgaccttccaacaccatctaatatctcagcactatgaaacttcgggtctctcctaggattatgtttaattacccaaatcctaacgggattattcttagcgatgcactacacctccgacatctcaaccgcattttcatcagtaacccacatctgccgagatgtaaactacggctgacttattcgaagtctgcatgccaatggagcctccttcttcttcatctgtctttacatgcacattgcacgaggactatactatggctcatacctatataaagaaacctgaaacattggtgtagtcctgttcctcctagttataataacagcctttgtcggctacgtacttccgtggggacagatgtctttttgaggcgccacggtaattacgaacctgctctcagcggtcccttacataggggacatgcttgttcaatgaatctgaggtggtttctcagtagataatgcaacccttacccgattcttcgcattccacttcctcctaccgtttgtcgtcgcaggcgcaaccatcctgcacctgctgtttctacacgaaacaggatcaaacaacccagccggactaaactccgacgccgacaaaatttctttccacccatacttctcatataaagaccttcttggctttgttatcatattactagcccttacctctctagcattattttctcccaacctactgggtgacccagaaaactttaccccagcaaacccactagtgacgcccccacatattcagccagaatggtacttcttatttgcctacgccattctccggtctatcccaaacaaactaggaggggtcctggcgctactattcagtatcctagtgctactagttgtgccaatcttacacacctcaaaacaacgaggactaactttccgccccgtaactcaattcctattctgaaccctagttgcagatatatttatcctgacatgaattgggggcatacctgtagaacacccatatattatcattggccaagtcgcatccattctatactttgcgctcttcctcgtccttgtcccgctagcaggatgagtcgaaaataaagcactgaaatgagccAY549461Squaliuscephalus                atggcaagcctacgaaaaacccacccactaatgaaaatcgccaatggcgcactagtcgaccttccaacaccatctaatatctcagcactatgaaacttcgggtctctcctaggattatgtttaattacccaaatcctaacgggattattcttagcgatgcactacacctccgacatctcaaccgcattttcatcagtaacccacatctgccgagatgtaaactacggctgacttattcgaagtctgcatgccaatggagcctccttcttcttcatctgtctttacatgcacattgcacgaggactatactatggctcatacctatataaagaaacctgaaacattggtgtagtcctgttcctcctagttataataacagcctttgtcggctacgtacttccgtggggacagatgtctttttgaggcgccacggtaattacgaacctgctctcagcggtcccttacataggggacatgcttgttcaatgaatctgaggtggtttctcagtagataatgcaacccttacccgattcttcgcattccacttcctcctaccgtttgtcgtcgcaggcgcaaccatcctgcacctgctgtttctacacgaaacaggatcaaacaacccagccggactaaactccgacgccgacaaaatttctttccacccatacttctcatataaagaccttcttggctttgttatcatattactagcccttacctctctagcattattttctcccaacctactgggtgacccagaaaactttaccccagcaaacccactagtgacgcccccacatattcagccagaatggtacttcttatttgcctacgccattctccggtctatcccaaacaaactaggaggggtcctggcgctactattcagtatcctagtgctactagttgtgccaatcttacacacctcaaaacaacgaggactaactttccgccccgtaactcaattcctattctgaaccctagttgcagatatatttatcctgacatgaattgggggcatacctgtagaacacccatatattatcattggccaagtcgcatccattctatactttgcgctcttcctcgtccttgtcccgctagcaggatgagtcgaaaataaagcactgaaatgagccHM560183Squaliusillyricus               ATGGCAAGCCTACGAAAAACTCACCCACTAATAAAAATCGCCAACGGCGCACTAGTTGACCTTCCAACACCATCTAATATCTCAGCACTATGAAACTTCGGTTCCCTCCTAGGATTATGTTTAATTACCCAGATCCTGACAGGACTATTCTTAGCAATGCACTACACCTCCGACATCTCAACCGCATTTTCATCTGTGACCCATATCTGCCGAGATGTTAACTATGGCTGACTTATTCGAAATCTGCATGCCAATGGGGCATCCTTCTTCTTCATTTGCCTTTACATGCATATTGCACGGGGCCTATATTATGGCTCGTACCTATACAAAGAGACCTGAAACATCGGTGTGGTCCTGTTTCTCCTAGTTATAATGACAGCCTTCGTCGGCTACGTACTTCCATGAGGACAAATGTCCTATTGAGGTGCCACGGTAATTACAAATCTCCTCTCAGCAGTCCCCTACATAGGGGATACTCTTGTTCAATGAATCTGGGGCGGTTTCTCCGTAGACAACGCAACCCTCACCCGATTCTTCGCATTCCACTTCCTCCTGCCGTTTGTCGTCGCGGGCGCAACCATCCTGCACCTGTTATTCCTACACGAAAGAGGATCGAACAACCCAGCCGGACTAAATTCTGACGCCGACAAGATTTCTTTCCACCCGTACTTCTCATATAAAGACCTTCTTGGCTTTGTTATCATATTACTAGCCCTTACCTCTCTAACATTGTTTTCTCCCAACCTATTAGGTGACCCAGAGAACTTTACCCCAGCAAACCCACTAGTAACGCCCCCACACATTCAGCCAGAGTGGTACTTCTTATTTGCCTACGCCATCCTCCGATCTATTCCAAATAAACTAGGAGGGGTCCTTGCACTACTATTCAGCATCCTAGTGCTAATGGTTGTGCCAATCTTACATACCTCAAAACAACGAGGACTAACTTTCCGCCCCGTAACTCAATTCCTATTCTGAACCCTAGTTGCAGATATATTTATCCTGACATGAATCGGGGGCATACCTGTAGAACACCCATATATTATCATTGGCCAAGTCGCATCCGTTTTATACTTTGCACTATTCCTCATTCTTGTCCCACTGGCAGGGTGAATAGAAAATAAAGCATTGAAATGAGCCHM560184Squaliusillyricus               ATGGCAAGCCTACGAAAAACTCACCCACTAATAAAAATCGCCAACGGCGCACTAGTTGACCTTCCAACACCATCTAATATCTCAGCACTATGAAACTTCGGTTCCCTCCTAGGATTATGTTTAATTACCCAGATCCTGACAGGACTATTCTTAGCAATGCACTACACCTCCGACATCTCAACCGCATTTTCATCTGTGACCCATATCTGCCGAGATGTTAACTATGGCTGACTTATTCGAAATCTGCATGCCAATGGGGCATCCTCCTTCTTCATTTGCCTTTACATGCATATTGCACGGGGCCTATATTATGGCTCGTACCTATACAAAGAGACCTGAAACATCGGTGTGGTCCTGTTTCTCCTAGTTATAATGACAGCCTTCGTCGGCTACGTACTTCCATGAGGACAAATGTCCTTTTGAGGTGCCACGGTAATTACAAATCTCCTCTCAGCAGTCCCCTACATAGGGGATACTCTTGTTCAATGAATCTGGGGCGGTTTCTCCGTAGACAACGCAACCCTCACCCGATTCTTCGCATTTCACTTCCTCCTGCCGTTTGTCGTCGCGGGCGCAACCATCCTGCACCTGTTATTCCTACACGAAACAGGATCGAACAACCCAGCCGGACTAAATTCTGACGCCGACAAGATTTCTTTCCACCCGTACTTCTCATATAAAGACCTTCTTGGCTTTGTTATCATATTACTAGCCCTTACCTCTCTAACATTGTTTTCTCCCAACCTATTAGGTGACCCAGAGAACTTTACCCCAGCAAACCCACTAGTAACGCCCCCACACATTCAGCCAGAGTGGTACTTCTTATTTGCCTACGCCATCCTCCGATCTATTCCAAATAAACTAGGAGGGGTCCTTGCACTACTATTCAGCATCCTAGTGCTAATGGTTGTGCCAATCTTACATACCTCAAAACAACGAGGACTAACTTTCCGCCCCGTAACTCAATTCCTATTCTGAACCCTAGTTGCAGATATATTTATCCTGACATGAATCGGGGGCATACCTGTAGAACACCCATATATTATCATTGGCCAAGTCGCATCCGTTTTATACTTTGCACTATTCCTCATTCTTGTCCCACTGGCAGGGTGAATAGAAAATAAAGCATTGAAATGAGCCHM560185Squaliuskeadicus                ATGGCAAGCCTACGAAAGACCCACCCACTAATAAAAATCGCTAACGGCGCACTAGTCGATCTTCCAACACCATCTAATATTTCAGCAATATGAAACTTCGGATCCCTTCTAGGATTGTGTTTAATTACCCAAATCCTAACGGGACTATTTTTAGCAATACATTACACCTCCGATATCTCCACCGCATTTTCGTCCGTGACCCATATCTGCCGAGATGTTAACTACGGATGACTTATCCGAAGTCTACATGCCAATGGAGCATCCTTCTTCTTTATCTGTCTTTATATACATATTGCACGAGGCCTATATTATGGCTCGTACCTATACAAGGAAACCTGAAACATTGGTGTGGTCCTGTTTCTCCTGGTTATGATGACGGCCTTCGTCGGCTATGTGCTTCCATGGGGACAGATGTCCTTTTGGGGCGCCACAGTAATTACGAATCTCCTCTCAGCAGTCCCCTATATGGGGGACACTCTTGTTCAATGAATCTGGGGCGGTTTCTCCGTGGACAACGCAACTCTCACCCGATTCTTCGCGTTCCACTTCCTACTGCCATTTGTCGTCGCAGGCGCAGCGGTTCTCCACCTGTTATTTTTACACGAAACAGGATCGAACAACCCAGCCGGACTAAATTCTGACGCCGACAAGATTTCTTTCCACCCGTACTTCTCCTATAAAGACCTTCTTGGCTTTGTTGTAATATTACTAGCCCTAACCTCTCTAGCATTATTTTCTCCTAACCTATTAGGTGACCCAGAAAACTTCACCCCTGCGAACCCATTAGTAACGCCCCCGCATATTCAGCCAGAATGATACTTCTTGTTTGCCTACGCCATCCTCCGATCCATTCCAAATAAGCTAGGAGGGGTCCTTGCACTACTGTTTAGCATTCTGGTACTAATAGTTGTGCCCATTTTACACACCTCGAAACAACGAGGACTAACTTTCCGCCCCGTAACTCAATTCCTATTCTGAACCCTAGTTGCAGATATATTTATCCTAACATGAATTGGAGGCATACCTGTAGAACACCCATATATTATCATTGGCCAAATCGCATCCATTTTATACTTTACACTATTCCTAGTCCTCGTCCCACTAGCAGGCTGAATGGAAAATAAGGCATTGAAATGAGCCHM560186Squaliuskeadicus                ATGGCAAGCCTACGAAAGACCCACCCACTAATAAAAATCGCTAACGGCGCACTAGTCGATCTTCCAACACCATCTAATATTTCGGCAATATGAAACTTCGGATCCCTTCTAGGATTATGTTTAATTACCCAAATCCTAACGGGACTATTTTTAGCAATACATTACACCTCCGATATCTCCACCGCATTTTCGTCCGTGACCCATATCTGCCGAGATGTTAACTACGGATGACTTATCCGAAGTCTACATGCCAATGGAGCATCCTTCTTCTTTATCTGTCTTTATATACATATTGCACGAGGCCTATATTATGGCTCGTACCTATACAAGGAAACCTGAAACATTGGTGTAGTCCTGTTTCTCCTGGTTATGATGACGGCCTTCGTCGGCTATGTGCTTCCATGGGGACAGATGTCCTTTTGGGGCGCCACAGTAATTACGAATCTCCTCTCAGCAGTCCCCTATATGGGGGATACTCTTGTTCAATGAATCTGGGGCGGTTTCTCCGTGGACAACGCAACTCTCACCCGATTCTTCGCGTTCCACTTCCTACTGCCATTTGTCGTCGCAGGCGCAACGGTTCTCCACCTGTTATTTTTACACGAAACAGGATCGAACAACCCAGCCGGACTAAATTCTGACGCCGACAAGATTTCTTTCCACCCGTACTTCTCCTATAAAGACCTTCTTGGATTTGTTGTAATATTACTAGCCCTAACCTCTCTAGCATTATTTTCTCCTAACCTATTAGGTGACCCAGAAAACTTCACCCCTGCGAACCCATTAGTAACACCCCCGCATATTCAGCCAGAATGATACTTCTTGTTTGCCTACGCCATCCTCCGATCCATTCCAAATAAGCTAGGAGGGGTCCTTGCACTACTGTTTAGCATTCTGGTACTAATAGTTGTGCCCATTTTACACACCTCGAAACAACGAGGACTAACTTTCCGCCCCGTAACTCAATTCCTATTCTGAACCCTAGTTGCAGATATATTTATCCTAACATGAATTGGAGGCATACCTGTAGAACACCCATATATTATCATTGGCCAAATCGCATCCATTTTATACTTTACACTATTCCTAGTCCTCGTCCCACTAGCAGGCTGAATGGAAAATAAGGCATTGAAATGAGCCAJ252801Squaliuskosswigi                atggcaagcctacgaaaaacccacccactaataaaaatcgccaatggcgcactagtcgacctcccaacaccatctaatatctcagcactgtgaaacttcggatctcttctaggattatgtttaattacccagattctgacaggactattcttagcaatgcactacacctctgacatctcgaccgcgttttcatcagtaacccacatctgccgggatgttaactacggctgacttatccgaagcctgcatgccaatggagcctccttcttcttcatctgtctttatatacacattgcacgaggactatattatggctcatacctatataaagaaacctgaaacattggtgtagtcctgtttctcctagttataatgacagcctttgtcggctacgtacttccgtgaggacaaatatccttttgaggtgccacagtgattacaaacctcctctcagcagtcccttacatgggggacactcttgttcaatgaatctgaggcggtttctccgtagataacgcaacccttacccgattcttcgcattccacttcctcctgccatttgtcgtcgcaggcgcaaccatcctacacctgctatttctacacgagacaggatcaaacaacccagccggactaaattccgacgccgacaaaatttctttccacccgtacttctcgtacaaagaccttcttggctttgttattatactactagcccttacctctctagcactattttctcctaacctactgggtgacccagaaaactttaccccagcaaacccactagtaacgcccccacatattcagccagaatgatacttcctatttgcctacgccattctccgatctatcccaaacaaactaggaggggtactcgcactactattcagcatcctggtgctaatagttgtgccgatcttacacacctctaaacaacgaggactaactttccgccccgtaactcaattcctattctgaaccctagttgcagatatatttatcctgacatggattgggggcatacctgtagaacacccatatattatcattggccaagtcgcatccattctatattttgcactcttcctcattcttgtcccactagcaggatgaatggaaaataaagcattaaaatgagccAJ252804Squaliuskotelati                atggcaagcctacgaaaaacccacccactaataaaaatcgccaatgacgcactagtcgaccttccaacaccatctaatatctcagcactatgaaacttcgggtctctcctaggattatgtttaattacccaaatcctaacaggactattcttagcaatgcactacacctccgacatctcgaccgcattttcatcagtaacccatatctgccgagacgttaactacggctgacttattcggagtctgcatgccaacggagcctccttcttcttcatctgtctttatatgcacattgcacgaggactatattatggctcatacctatataaagaaacctgaaacattggtgtggtcctatttctcctagtcatgataacagcctttgttggctacgtcctcccatgaggacaaatgtccttctgaggcgccacagtgattacaaacctcctctcagcagtcccttacataggggacactcttgttcaatgaatctgaggcggtttctccgtagataatgcaaccctcacccgattcttcgcattccacttcctcctgccatttgtcgtcgcaggcgcaaccatcctgcacctgctgtttctacacgagacggggtcgaacaacccagccggactgaattccgacgccgacaagatttctttccacccgtacttctcatataaggaccttcttggctttgttatcatattactagcccttacctctctagcattattttctcctaacctactaggtgacccagaaaactttaccccagcaaacccactagtgacacccccacatattcagccagagtggtacttcttatttgcctacgccattctccgatctatcccaaataaactagggggggtccttgcactactgttcagcatcctagtgctaatggttgtgccaatcttacacacctcaaaacaacgaggactaactttccgccccgtaactcaattcctattctgaaccctagttgcagatatatttatcctgacatgaattggaggcatacccgtagaacatccatatattatcattggccaagtcgcatccgttctatactttgcactcttcctcgttcttgtcccactagcagggtgagtagaaaataaagcattgaaatgagccHM560187Squaliuslaietanus               ATGGCAAGCCTACGAAAAACCCACCCACTAATAAAAATCGCCAATGACGCACTAGTCGACCTTCCAACACCATCTAATATCTCAGCAATATGAAACTTCGGATCTCTCCTAGGATTGTGTTTAATTACCCAAATCCTAACAGGACTATTCTTAGCAATGCACTACACCTCTGACATCTCAACCGCATTTTCATCCGTAACCCACATCTGCCGAGATGTTAACTACGGCTGACTTATTCGGAGTCTGCATGCCAATGGGGCCTCCTTCTTCTTCATCTGTCTTTATATGCACATTGCACGAGGACTATATTATGGCTCATACCTATATAAAGAAACCTGAAACATTGGTGTAGTCCTGTTTCTCCTAGTTATAATGACGGCCTTTGTCGGCTACGTACTTCCATGAGGGCAAATGTCCTTTTGAGGTGCCACAGTAATTACGAACCTCCTCTCAGCAGTCCCTTACATGGGAGACACTCTTGTTCAATGAATCTGGGGCGGTTTCTCCGTAGACAACGCGACCCTCACCCGATTCTTCGCGTTCCACTTCCTTCTGCCGTTTGTCGTCGCGGGCGCAACCATCCTGCACCTGCTATTTCTACACGAAACGGGGTCAAACAACCCAGCCGGACTAAATTCCGACGCCGACAAAATTTCTTTCCACCCGTACTTCTCATATAAAGACCTTCTTGGCTTTGTTATCATATTACTAGCCCTTACCTCTCTAGCACTATTTTCTCCTAACCTACTAGGTGACCCAGAAAACTTTACCCCAGCAAACCCACTGGTAACGCCCCCACATATTCAACCAGAGTGATACTTCTTGTTTGCCTACGCCATTCTCCGGTCTATTCCAAACAAACTAGGAGGGGTCCTTGCACTGCTATTTAGCATCCTAGTGCTAATGGTTGTGCCAATCTTACACACCTCTAAACAACGAGGACTAACTTTCCGCCCCGTAACTCAATTCCTATTCTGAACCCTAGTTGCAGATATATTTATCCTGACATGAATTGGGGGCATACCTGTAGAACACCCGTACATTATCATTGGCCAAGTCGCATCCATCCTATATTTTGCGCTCTTCCTCATTCTTATCCCGCTGGCAGGATGAGTAGAAAACAAAGCATTGAAATGAGCCHM560188Squaliuslaietanus               ATGGCAAGCCTACGAAAAACCCACCCACTAATAAAAATCGCCAATGACGCACTAGTCGACCTTCCAACACCATCTAATATCTCAGCAATATGAAACTTCGGATCTCTCCTAGGATTGTGTTTAATTACCCAAATCCTAACAGGACTATTCTTAGCAATGCACTACACCTCTGACATCTCAACCGCATTTTCATCCGTAACCCACATCTGCCGAGATGTTAACTACGGCTGACTTATTCGGAGTCTGCATGCCAATGGGGCCTCCTTCTTCTTCATCTGTCTTTATATGCACATTGCACGAGGACTATATTATGGCTCATACCTATATAAAGAAACCTGAAACATTGGTGTAGTCCTGTTTCTCCTAGTTATAATGACGGCCTTTGTCGGCTACGTACTTCCATGAGGGCAAATGTCCTTTTGAGGTGCCACAGTAATTACGAACCTCCTCTCAGCAGTCCCTTACATGGGAGACACTCTTGTTCAATGAATCTGGGGCGGTTTCTCCGTAGACAACGCGACCCTCACCCGATTCTTCGCGTTCCACTTCCTTCTGCCGTCTGTCGTCGCGGGCGCAACCATCCTGCACCTGCTATTTCTACACGAAACGGGGTCAAACAACCCAGCCGGACTAAATTCCGACGCCGACAAAATTTCTTTCCACCCGTACTTCTCATATAAAGACCTTCTTGGCTTTGTTATCATATTACTAGCCCTTACCTCTCTAGCACTATTTTCTCCTAACCTACTAGGTGACCCAGAAAACTTTACCCCAGCAAACCCACTGGTAGCGCCCCCACGTATTCAACCAGAGTGATACTTCTTGTTTGCCTACGCCATTCTCCGGTCTATTCCAAACAAACTAGGAGGGGTCCTTGCACTGCTATTTAGCATCCTAGTGCTAATGGTTGTGCCAATCTTACACACCTCTAAACAACGAGGACTAACTTTCCGCCCCGTAACTCAATTCCTATTCTGAACCCTAGTTGCAGATATATTTATCCTGACATGAATTGGGGGCATACCTGTAGAACACCCGTACATTATCATTGGCCAAGTCGCATCCATCCTATATTTTGCGCTCTTCCTCATTCTTATCCCGCTAGCAGGATGAGTAGAAAACAAAGCATTGAAATGAGCCAJ252806Squaliuslepidus                 atggcaagcctacgaaaaacccacccactaataaaaatcgccaatgacgcactagtcgaccttccgacaccgtctaatatctcagcattgtgaaacttcgggtctctcctaggattgtgtttaattacccaaatcctaacaggactattcctagcaatgcactacacctccgacatctcaaccgcattttcttccgtaacccatatctgccgagacgttaactacggctgacttattcggagtttgcatgccaatggggcctccttcttcttcatctgtctttatatgcacattgcacgaggactatattatggctcgtacctatataaagaaacctgaaacattggtgtagtcctattcctcctagttataatgacagcctttgtcggctacgtactcccatggggacaaatgtccttttgaggcgccacagtaattacaaacctcctctcagcagtcccttacatgggggacactcttgttcaatgaatctggggtggtttctccgtagataatgcaacccttacccgattcttcgcgttccacttcctcctcccatttgtcgtcgcaggcgcaaccatcctacacctgctatttctacacgagacgggatcgaacaacccagccgggctaaattccgacgccgacaaaatttctttccacccgtacttctcatataaggaccttcttggctttgttatcatattactagcccttacctctctagcattattttctcctaacctactaggtgacccagagaactttaccccagcaaacccactagtgacgcccccacatattcagccagaatggtacttcttatttgcctacgccattctccgatctatcccaaataagctaggaggggtccttgcactattattcagcatcctggtgctaatagttgtgccagtcttacacacctcaaaacaacgaggactaactttccgccccgtaactcaattcctattctgaaccctggttgcagatatatttatcttgacatgaattgggggcatacctgtagaacacccatacattatcattggccaagtcgcatccattctatactttgcactcttcctcatccttgtcccactagcaggatgagtagaaaataaagcattaaaatgagccHM560189Squaliuslucumonis               ATGGCAAGCCTACGAAAGACCCACCCACTAATAAAAATCGCCAACGACGCACTAGTCGACCTTCCAACACCGTCTAATATCTCAGCATTGTGAAACTTCGGTTCCCTCCTAGGATTATGTTTAATTACCCAAATCCTGACAGGACTATTCCTGGCAATGCACTACACTTCCGACATCTCAACCGCGTTTTCATCCGTCACCCATATCTGCCGAGATGTTAACTACGGCTGACTTATTCGGAATCTGCATGCCAATGGAGCATCCTTCTTCTTCATCTGTCTCTACATACATATTGCACGGGGCCTATATTATGGCTCGTACCTATACAAAGAAACCTGAAACATTGGTGTAGTCCTGTTTCTCCTAGTTATAATGACAGCCTTCGTCGGCTACGTACTTCCATGAGGACAAATGTCCTTTTGAGGTGCCACGGTAATTACAAATCTCCTCTCAGCAGTCCCTTACATAGGGGATACTCTTGTTCAATGAATCTGAGGCGGTTTCTCCGTAGACAACGCAACCCTCACCCGATTCTTCGCATTCCACTTCCTCCTACCGTTTGTCGTCGCGGGCGCAACCATCCTACACCTACTATTCCTACACGAAACAGGATCGAACAACCCAGCCGGGCTAAATTCCGACGCCGACAAAATCTCCTTCCACCCGTACTTCTCATATAAAGACCTCCTTGGCTTTGTTATCATATTACTAGCCCTTACCTCTTTAACATTATTTTCTCCTAACCTACTAGGTGACCCAGAGAACTTTACTCCAGCGAACCCACTAGTGACACCCCCACATATTCAGCCAGAATGGTATTTCTTATTTGCCTACGCCATCCTGCGGTCCATTCCAAATAAACTAGGAGGGGTCCTTGCACTACTGTTCAGCATCCTAGTGCTAATAGTTGTGCCAATTTTACACACCTCGAAACAACGAGGACTAACTTTCCGCCCTATAACTCAATTCCTATTCTGAACCCTAGTTGCAGATATACTTATTCTGACATGAACCGGGGGCATGCCTGTAGAACACCCATATATTATCATTGGCCAAGTCGCATCCATTTTATATTTTGCACTATTCCTCATTCTTGTCCCACTAGCAGGGTGAATAGAAAATAAAGCATTGAAATGAGCCHM560190Squaliuslucumonis               ATGGCAAGCCTACGAAAGACCCACCCACTAATAAAAATCGCCAACGACGCACTAGTCGACCTTCCAACACCGTCTAATATCTCAGCATTGTGAAACTTCGGTTCCCTCCTAGGATTATGTTTAATTACCCAAATCCTGACAGGACTATTCCTGGCAATGCACTACACTTCCGACATCTCAACCGCGTTTTCATCCGTCACCCATATCTGCCGAGATGTTAACTACGGCTGACTTATTCGGAATCTGCATGCCAATGGAGCATCCTTCTTCTTCATCTGTCTCTACATACATATTGCACGGGGCCTATATTATGGCTCGTACCTATACAAAGAAACCTGAAACATTGGTGTAGTCCTGTTTCTCCTAGTTATAATGACAGCCTTCGTCGGCTACGTACTTCCATGAGGACAAATGTCCTTTTGAGGTGCCACGGTAATTACAAATCTCCTCTCAGCAGTCCCTTACATAGGGGATACTCTTGTTCAATGAATCTGAGGCGGTTTCTCCGTAGACAACGCAACCCTCACCCGATTCTTCGCATTCCACTTCCTCCTGCCGTTTGTCGTCGCGGGCGCAACCATCCTACACCTACTATTCCTACACGAAACAGGATCGAACAACCCAGCCGGGCTAAATTCCGACGCCGACAAAATCTCCTTCCACCCGTACTTCTCATATAAAGACCTCCTTGGCTTTGTTATCATATTACTAGCCCTTACCTCTTTAACATTATTTTCTCCTAACCTACTAGGTGACCCAGAGAACTTTACTCCAGCGAACCCACTGGTGACACCCCCACATATTCAGCCAGAATGGTATTTCTTATTTGCCTACGCCATCCTGCGGTCCATTCCAAATAAACTAGGAGGGGTCCTTGCACTACTGTTCAGCATCCTAGTGCTAATAGTTGTGCCAATTTTACACACCTCGAAACAACGAGGACTAACTTTCCGCCCTATAACTCAATTCCTATTCTGAACCCTAGTTGCAGATATACTTATTCTGACATGAATCGAGGGCATGCCTGTAGAACACCCATATATTATCATTGGCCAAGTCGCATCCATTTTATATTTTGCACTATTCCTCATTCTTGTCCCACTAGCAGGGTGAATAGAAAATAAAGCATTGAAATGAGCCHM560192Squaliusmalacitanus             ATGGCAAGCCTACGAAAAACCCACCCACTGATAAAAATCGCCAACGGCGCACTAGTCGACCTCCCAACACCCTCTAATATCTCAGCACTATGAAACTTTGGATCCCTCCTCGGACTGTGCTTAATTACCCAAATCCTAACCGGACTATTCTTGGCCATGCACTACACCTCGGACATCTCAACCGCATTTTCATCCGTAACCCATATCTGCCGGGATGTTAACTACGGCTGACTTATTCGAAGCCTGCATGCCAATGGAGCATCCTTTTTCTTTATCTGTCTTTATATACATATTGCGCGAGGCCTATATTATGGCTCCTACCTCTACAAAGAAACCTGAAGCATCGGTGTAGTCCTGTTTCTCCTGGTTATAATAACGGCCTTCGTCGGCTACGTACTTCCGTGGGGACAAATATCCTTTTGGGGTGCCACAGTGATTACGAATCTCCTATCAGCAGTCCCCTACATAGGAGACACCCTTGTTCAGTGAATCTGGGGAGGTTTCTCCGTAGACAACGCAACACTCACCCGGTTCTTCGCATTCCACTTCCTCCTTCCATTTGTCGTTGCAGGCGCAACCATCTTGCACCTATTATTCCTCCACGAAACAGGATCAAACAACCCAGCCGGGCTAAATTCCGACGCCGACAAAATCTCCTTCCACCCATACTTTTCATATAAAGACCTTCTTGGCTTTGTTATCATACTACTAGCCCTCACCTCTCTGGCATTGTTTTCCCCTAATCTACTAGGTGACCCAGAGAACTTTACCCCAGCGAACCCGCTAGTAACTCCTCCCCATATTCAGCCAGAGTGATACTTCTTGTTTGCCTATGCCATCCTCCGGTCTATCCCAAACAAATTAGGAGGGGTCCTTGCACTACTATTCAGCATTCTAGTGCTATTAGTTGTGCCGATTTTACACACCTCAAAGCAGCGAGGACTAACGTTCCGCCCCGCAACGCAGCTCCTATTCTGAACCCTCGTTGCAGATATACTCATCCTGACGTGAATTGGGGGCATACCTGTAGAACATCCGTACATCGTCATTGGTCAAGTCGCATCCATTCTATACTTTGCACTCTTCCTCGTTCTTGTCCCACTAGCGGGGTGAATGGAAAATAAAGCATTAAAATGAGCTHM560191Squaliusmalacitanus             ATGGCAAGCCTACGAAAAACCCACCCACTGATAAAAATCGCCAACGGCGCACTAGTCGACCTCCCAACACCCCCTAATATCTCAGCACTATGAAACTTTGGATCCCTCCTCGGACTGTGCTTAATTACCCAAATCCTAACCGGACTATTCTTGGCCATGCACTACACCTCGGACATCTCAACCGCATTTTCATCTGTAACCCATATCTGCCGGGATGTTAACTACGGCTGACTTATTCGAAGCCTGCATGCCAATGGAGCATCCTTTTTCTTTATCTGTCTTTATATACATATTGCGCGAGGCCTATATTATGGCTCCTACCTCTACAAAGAAACCTGAAGCATCGGTGTAGTCCTGTTTCTCCTGGTTATAATAACGGCCTTCGTCGGCTACGTACTTCCGTGGGGACAAATATCCTTTTGGGGTGCCACAGTGATTACGAATCTCCTATCAGCAGTCCCCTACATAGGAGACACCCTTGTTCAGTGAATCTGGGGAGGTTTCTCCGTAGACAACGCAACACTCACCCGGTTCTTCGCATTCCACTTCCTCCTTCCATTTGTCGTTGCAGGCGCAACCATCTTGCACCTATTATTCCTCCACGAAACAGGATCAAACAACCCAGCCGGGCTAAATTCCGACGCCGACAAAATTTCCTTCCACCCATACTTTTCATATAAAGACCTTCTTGGCTTTGTTATCATACTACTAGCCCTCACCTCTCTGGCATTGTTTTCCCCTAATCTACTAGGTGACCCAGAGAACTTTACCCCAGCGAACCCGCTAGTAACTCCTCCCCATATTCAGCCAGAGTGATACTTCTTGTTTGCCTATGCCATCCTCCGGTCTATCCCAAACAAATTAGGAGGGGTCCTTGCACTACTATTCAGCATTCTAGTGCTATTAGTTGTGCCGATTTTACACACCTCAAAGCAGCGAGGACTAACGTTCCGCCCCGCAACGCAGTTCCTATTCTGAACCCTCGTTGCAGATATACTCATCCTGACGTGAATTGGGGGCATACCTGTAGAACATCCGTACATCGTCATTGGTCAAGTCGCATCCATTCTATACTTTGCACTCTTCCTCGTTCTTGTCCCACTAGCGGGGTGAATGGAAAATAAAGCATTAAAATGAGCTHM560193Squaliusmicrolepis              ATGGCAAGCCTACGAAAAACCCACCCACTAATAAAAATCGCCAACGGCGCACTAGTCGACCTTCCGACACCCTCTAATATCTCAGCACTATGAAACTTCGGTTCCCTCCTAGGATTATGTTTAATTACCCAGATCCTGACAGGACTATTCTTGGCAATGCACTACACCTCCGACATCTCAACCGCATTTTCATCTGTCACCCATATCTGCCGAGATGTTAACTACGGCTGACTTATTCGAAATCTGCATGCCAATGGAGCATCCTTCTTCTTCATCTGTCTTTACATACATATTGCACGGGGCCTATATTATGGCTCATACCTATACAAAGAAACCTGAAACATTGGTGTAGTCCTGTTTCTCCTAGTTATAATGACAGCCTTCGTCGGCTACGTACTTCCATGAGGACAAATGTCCTTTTGAGGTGCCACGGTAATTACAAATCTCCTCTCAGCAGTCCCTTACATAGGGGATACTCTTGTTCAATGAATCTGAGGCGGTTTCTCCGTAGACAACGCAACCCTCACCCGATTCTTCGCATTCCACTTCCTCCTACCATTTGTCGTCGCAGGCGCAACCATCCTGCACCTGTTATTCCTACACGAAACAGGATCGAACAACCCAGCCGGACTAAATTCTGACGCCGACAAAATCTCTTTCCACCCGTACTTCTCATATAAAGACCTTCTTGGCTTTGTTATCATATTACTAGCCCTTACCTCTTTAACATTATTTTCTCCCAACCTATTAGGTGACCCAGAAAACTTTACCCCAGCAAACCCACTGGTGACACCCCCACATATTCAGCCAGAATGGTATTTCTTATTTGCCTACGCCATCCTCCGATCTATTCCAAGTAAACTAGGAGGGGTCCTTGCACTACTGTTCAGCATCCTAGTGCTAATAGTTGTGCCAATTTTACACACCTCAAAACAACGAGGACTAACTTTCCGCCCCATAACTCAATTCCTATTCTGAACCCTAGTTGCAGATATATTTATTCTGACATGAATCGGGGGCATACCTGTAGAACACCCATATATTATCATTGGCCAAGTCGCATCTATTTTATATTTTGCGCTATTCCTCGTTCTTGTCCCACTGGCAGGGTGAATAGAAAATAAAGCATTGAAATGAGCCHM930194Squaliusmicrolepis              ATGGCAAGCCTACGAAAAACCCACCCACTAATAAAAATCGCCAACGGCGCACTAGTCGACCTTCCGACACCCTCTAATATCTCAGCACTATGAAACTTCGGTTCCCTCCTAGGATTATGTTTAATTACCCAGATCCTGACAGGACTATTCTTGGCAATGCACTACACCTCCGACATCTCAACCGCATTTTCATCTGTCACCCATATCTGCCGAGATGTTAACTACGGCTGACTTATTCGAAATCTGCATGCCAATGGAGCATCCTTCTTCTTCATCTGTCTTTACATACATATTGCACGGGGCCTATATTATGGCTCATACCTATACAAAGAAACCTGAAACATTGGGGTAGTCCTGTTTCTCCTAGTTATAATGACGGCCTTCGTCGGCTACGTACTTCCATGAGGACAAATGTCCTTTTGAGGTGCCACGGTAATTACAAATCTCCTCTCAGCAGTCCCTTACATAGGGGATACTCTTGTTCAATGAATCTGAGGCGGTTTCTCCGTAGACAACGCAACCCTCACCCGATTCTTCGCATTCCACTTCCTCCTACCATTTGTCGTCGCAGGCGCAACCATCCTGCACCTGTTATTCCTACACGAAACAGGATCGAACAACCCAGCCGGACTAAATTCCGACGCCGACAAAATCTCTTTCCACCCGTACTTCTCATATAAAGACCTTCTTGGCTTTATTATCATATTACTAGCCCTTACCTCTTTAACATTATTTTCTCCCAACCTATTAGGTGACCCAGAGAACTTTACCCCAGCAAACCCACTGGTGACACCCCCGCATATTCAGCCAGAATGGTATTTCTTATTTGCCTACGCCATCCTCCGATCTATTCCAAATAAACTAGGAGGGGTCCTTGCACTACTGTTCAGCATCCTAGTGCTAATAGTTGTGCCAATTTTACACACCTCAAAACAACGAGGACTAACTTTCCGCCCCATAACTCAATTCCTATTCTGAACCCTAGTTGCAGATATATTTATTCTGACATGAATCGGGGGCATACCTGTAGAACACCCATATATTATCATTGGCCAAGTCGCATCTATTTTATATTTTGCGCTATTCCTCGTTCTTGTCCCACTGGCAGGGTGAATAGAAAATAAAGCATTGAAATGAGCCAF090758Squaliusmoreoticus              atggcaagcctacgaaaaacccacccactaataaaaatcgccaatggcgcactagtcgaccttccgacaccatctaatatctcagcactatgaaacttcggatctctcctaggattatgtttaattacccaaatcctaacggggctattcctagcgatgcactacacctccgacatctcgaccgcattttcgtcggtgacccacatctgccgagatgttaactacggctgacttattcgaagtctacatgccaatggagcctccttcttcttcatctgcctttacatacacattgcacgaggactatattatggctcgtacctatataaggaaacctgaaacattggtgtagtcctgtttctcctagttataatgacagcctttgtcggttacgtacttccatgaggacaaatgtccttttggggtgccacggtaattacaaacctcctctcagcagtcccttacataggggacactcttgttcaatgaatctgaagcggtttctccgtagataacgcgaccctcaaccgattcttcgcgttccacttcctcctaacatttgtcgtcgcaggtgcaaccgtcctacacttgctgtttctacacgagacgggatcaaacaacccagccggcttaaattcggacgccgacaaaatttctttccacccatacttctcatataaagaccttcttggtttcgttatcatattattagcccttacctccttagcattattttctcctaacctactgggtgacccagaaaacttcacccctgcgaacccactggtgactcccccacatattcagccggagtggtatttcttgtttgcctacgccattctccgatctattccaaacaaactaggaggggtccttgcgctactgttcagcatcctagtgctaatggttgtgccaatcttacacacctctaaacaacgaggactaactttccgccccataactcaattcctattctgaaccctagttgcagatatatttatcctgacatgaattgggggcatacctgtagaacacccatatattatcattggccaagtcgcatccattctatattttgcactcttcctcgttcttgtcccactagcaggatgagtggaaaataaagcactgaaatgagccHM560195Squaliusorientalis              NNNNNNNNNNNNNNNNNNNNNNNNNNNNNNNNNNNNNNNNNNNNNNNNNNNNNNNNNNACCTTCCAACACCATCTAATATCTCAGCACTATGAAACTTCGGGTCTCTCCTAGGATTATGTTTAATTACCCAAATCCTAACGGGATTATTCTTAGCGATGCACTACACCTCCGACATCTCAACCGCATTTTCGTCAGTAACCCACATCTGCCGAGATGTAAACTACGGCTGACTTATTCGAAGTCTGCATGCCAATGGAGCCTCCTTCTTCTTCATCTGTCTTTACATGCACATTGCACGAGGACTATACTATGGCTCATACCTATATAAAGAAACCTGAAACATTGGTGTAGTCCTGTTCCTCCTAGTTATAATAACAGCCTTTGTCGGCTACGTACTTCCGTGGGGACAGATGTCTTTTTGGGGCGCCACGGTAATTACGAACCTGCTCTCAGCGGTCCCTTACATAGGGGACATGCTTGTTCAATGAATCTGAGGTGGTTTCTCAGTAGATAATGCAACCCTTACCCGATTCTTCGCATTCCACTTCCTCCTACCATTTGTCGTCGCAGGCGCAACCATCCTGCACCTGCTGTTTCTACACGAAACAGGATCAAACAACCCAGCCGGACTAAACTCCGACGCCGACAAAATTTCTTTCCACCCGTACTTCTCATATAAAGACCTTCTTGGCTTTGTTATCATATTACTAGCCCTTACCTCTCTAGCATTATTTTCTCCCAACCTACTGGGTGACCCAGAAAACTTTACCCCAGCAAACCCACTAGTGACACCCCCACATATTCAGCCAGAGTGGTACTTCTTATTTGCCTACGCCATTCTCCGGTCTATCCCAAACAAACTAGGAGGGGTCCTGGCGCTACTATTCAGTATCCTAGTGCTACTAGTTGTGCCAATCTTACACACCTCAAAACAACGAGGACTAACTTTCCGCCCCGTAACTCAATTCCTATTCTGAACCCTAGTTGCAGATATACTTATCCTGACATGAATTGGGGGCATACCTGTAGAACACCCATATATTATCATTGGCCAAGTTGCATCCATTCTATACTCTGCGCTCTTCCTCGTNNNNNNNNNNNNNNNNNNNNNNNNNNNNNNNNNNNNNNNNNNNNNNNNNHM560196Squaliusorientalis              ATGGCAAGCCTACGAAAAACCCACCCACTAATGAAAATCGCCAATGGCGCACTAGTCGACCTTCCAACACCATCTAATATCTCAGCACTATGAAATTTCGGGTCTCTCCTAGGATTATGTTTAATTACCCAAATCCTAACGGGATTATTCTTAGCAATGCACTACACCTCCGACATCTCAACCGCATTTTCGTCAGTAACCCACATCTGCCGAGATGTAAACTACGGCTGACTTATTCGAAGTCTGCATGCCAATGGAGCCTCCTTCTTCTTCATCTGTCTTTACATGCACATTGCACGAGGACTATACTATGGCTCATACCTATATAAAGAAACCTGAAACATTGGTGTAGTCCTGTTCCTCCTAGTTACAATAACAGCCTTTGTCGGCTACGTACTTCCGTGGGGACAGATGTCTTTTTGGGGCGCCACGGTAATTACGAACCTGCTCTCAGCGGTCCCTTACATAGGGGACATGCTTGTTCAATGAATCTGAGGTGGTTTCTCAGTAGATAATGCAACCCTTACCCGATTCTTCGCATTCCACTTCCTCCTACCATTTGTCGTCGCAGGCGCAACCATCCTGCACCTGCTGTTTCTACACGAAACAGGATCAAACAACCCAGCCGGACTAAACTCCGACGCCGACAAAATTTCTTTCCACCCATACTTCTCATATAAAGACCTTCTTGGCTTTGTTATCATATTACTAGCCCTTACCTCTCTAGCATTATTTTCTCCCAACCTACTGGGTGACCCAGAAAACTTTACCCCAGCAAACCCACTAGTGACACCCCCACATATTCAGCCAGAGTGGTACTTCTTATTTGCCTACGCCATTCTCCGGTCTATCCCAAACAAACTAGGAGGGGTCCTGGCGCTACTATTCAGTATCCTAGTGCTACTAGTTGTGCCAATCTTACACACCTCAAAACAACGAGGACTAACTTTCCGCCCCGTAACTCAATTCCTATTCTGAACCCTAGTTGCAGATATATTTATCCTGACATGAATTGGGGGCATACCTGTAGAACACCCATATATTATCATTGGCCAAGTCGCATCCATTCTATACTTTGCGCTCTTCCTCGTCCTTGTCCCGCTAGCAGGATGAGTCGAAAATAAAGCACTGAAATGAGCCAF090752Squaliusorpheus                 atggcaagcctacgaaaaacccacccactaataaaaatcgccaatgacgcactagtcgaccttccaacaccatctaacatctcagctatatgaaacttcggatctctcctgggattatgtttaattacccaaatcctaacgggactattcctagcaatgcactacacctctgacatctcaaccgcattttcatccgtaacccacatctgccgagatgttaactacggctgacttattcggagtctgcatgccaatggagcctccttcttcttcatctgtctttatatgcacattgcacgangactatattatggctcatacctatataaagaaacctgaaacattggtgtagtcctgtttctcctagttataatgacggcctttgtcggctacgtacttccatgaggacaaatgtccttttgaggtgctacggtaattacgaacctcctctcagcagtcccttacataggggatactcttgttcaatgaatctgaggcggtttctccgtagacaacgcaaccctcatccgattcttcgcgttccacttcctcctgccatttgtcgtagcaggcgcaaccatcatgcacttgctatttctacacgaaacggggtcaaacaacccggccggactaaattccgacgccgacaaaatttctttccacccgtacttctactataaagaccttcttggctttgttattatattactagcccttacctccctagcactattttctcctaacctactaggtgacccagaaaactttaccccagcaaacccactggtgacgcccccacatattcagccagaatgatacttcttgtttgcctacgccattctccgatctattcccaacaagctaggaggggtccttgcactactattcagcatcctggtgctaatagttgtgccaatcttacacacctctaaacaacgaggactaactttccgccccgtaactcaattcctattctgaaccctagttgcagatatacttatcctgacatgaattgggggcatacctgtagaacacccgtacattatcattggccaagtcgcatccattctatattttgcgctcttcctcgttcttgtcccactagcaggatgagtagaaaataaagcattgaaatgagccAF090756Squaliuspamvoticus              ATGGCAAGCCTACGAAAAACCCACCCACTAATAAAAATCGCCAATGGCGCACTAGTTGACCTTCCGACACCATCTAATATCTCAGCACTATGAAACTTCGGGTCTCTCCTAGGATTATGTTTAATTACCCAAATCCTAACAGGACTATTCCTAGCAATGCACTATACCTCCGACATCTCGACCGCATTTTCGTCGGTAACCCACATCTGCCGAGATGTTAACTACGGCTGACTTATTCGAAGTCTACATGCCAATGGAGCCTCCTTCTTCTTCATCTGCCTTTACATACACATTGCACGAGGACTATATTATGGCTCGTACCTATATAAAGAGACCTGAAACATTGGTGTGGTCCTGTTTCTCCTAGTTATGATGACAGCCTTTGTCGGCTACGTACTTCCGTGAGGACAAATGTCCTTTTGGGGTGCCACAGTAATTACAAACCTCCTCTCAGCAGTCCCTTACATAGGGGACACTCTTGTTCAGTGAATCTGGGGCGGTTTCTCCGTAGATAACGCAACCCTCACCCGATTCTTCGCGTTCCACTTCCTCCTGCCGTTTGTCGTCGCGGGTGCAACCGTCCTACACCTACTATTTCTACACGAAACGGGATCAAACAACCCAGCTGGGCTGAATTCCGACGCCGACAAGATTTCTTTCCACCCGTACTTCTCATATAAAGATCTTCTTGGCTTCGTTATCATATTATTAGCCCTTACCTCCTTAGCATTATTTTCTCCTAACCTACTAGGTGACCCAGAAAACTTTACCCCGGCAAACCCACTGGTGACGCCCCCACATATTCAGCCAGAGTGATATTTCTTGTTTGCCTACGCCATTCTCCGGTCTATTCCAAACAAACTAGGAGGGGTCCTTGCACTACTATTCAGCATCCTAGTGCTAATGGTTGTGCCGATCTTACACACCTCTAAACAACGAGGACTAACTTTCCGCCCCGCAACTCAGTTCCTATTCTGAACCCTAGTTGCAGATATATTTATCCTGACATGAATTGGGGGCATACCTGTAGAACACCCATATATTGTCATTGGCCAAGTCGCATCCATTCTATATTTTGCGCTCTTCCTCATTCTTGTCCCACTAGCAGGATGAGTGGAAAATAAAGCACTGAAATGAGCCAF090757Squaliuspeloponesis             atggcaagcctacgaaaaacccacccactaataaaaatcgccaatggcgcactagtcgaccttccgacaccatctaatatctcagcactatggaacttcggatcgctcctaggattatgtttaattacccaaatcctaacggggctattcctagcgatgcactacacctccgacatctcgaccgcattttcgtcggtgacccacatctgccgagatgttaactacggctggcttattcgaagtctacatgccaatggagcctccttcttcttcatctgcctttacatacacattgcacgaggactatactatggttcgtacctatataaggaaacctgaaacattggtgtaatcctgtttctcctagttataatgacagcctttgtcggttacgtacttccatgaggacaaatgtccttttggggtgccacggtaattacaaacctcctctcagcagtcccttacataggggacactcttgttcaatgaatctgaggcggtttctccgtagataacgcgaccctcacccgattcttcgcgttccacttcctcctgccatttgtcgtcgcaggtgcaaccatcctacacctgctatttctacacgagacgggatcaaacaacccagccggcctaaattccgatgccgacaaaatttctttccacccgtacttctcatataaagaccttcttggtttcgttatcatattgttagcccttacctccttagcattattttctcctaacctactgggtgacccggaaaacttcaccccagcaaacccactggtgactcccccacatattcagccggagtggtatttcttgtttgcctacgccattctccgatctatcccaaacaaactaggaggggtccttgcactactgttcagcatcctagtgctaatggttgtgccaatcttacacacctctaaacaacgaggactaactttccgccccgtaactcaattcctattctgaaccctagttgcagatatatttatcctgacatgaattgggggcatacctgtagaacacccatatattatcattggccaagtcgcatccattctatattttgcactcttcctcattcttgtcccactagcaggatgagtggaaaataaagcactgaaatgagccAJ252798Squaliusprespensis              atggcaagcctacgaaaaacccacccactaataaaaatcgccaatgacgcactagtcgaccttccgacaccatctaatatctcagcactatgaaacttcgggtctctcctgggattatgtttaattacccaaatcctaacaggactattcctagcaatgcactacacctccgacatctcaaccgcattttcatcggtaacccatatctgccgagatgttaactacggctgacttattcgaagtctacatgccaatggagcctccttcttcttcatctgtctttacatacacattgcacgaggactatattatggctcgtacctatataaggaaacctgaaacattggtgtagtcctatttctcctagttataatgacagcctttgtcggttacgtacttccatgaggacaaatgtccttttggggtgccacagtaattacaaacctcctctcagcagtcccttacataggagacactcttgttcaatgaatctgaggcggtttctccgtagataacgcaaccctcacccgattcttcgcgttccacttcctcctgccatttgtcgtcgcaggtgcaaccgtcctacacctgctatttctacatgaaacgggatcaaacaacccagccgggctaaattccgacgccgacaaaatttctttccacccgtacttctcatataaagaccttcttggcttcgttatcatattattagcccttacctccttagcattattttctcctaacctactaggtgacccagaaaactttaccccagcaaacccactagtgacacccccacatattcagccagagtgatatttcttgtttgcctacgccattctccgatctattccaaacaaactaggaggggtccttgcactactattcagcatcctagtgctaatagttgtgccaatcttacacacctctaaacaacgaggactaactttccgccctgtaactcaatttctattttgaaccctagttgcagatatactcatcctgacatgaattgggggcatacctgtagagcacccatatatcatcattggccaagtcgcatccattctatattttgcactattcctcgttcttgtcccactagcaggatgagtggaaaataaagcactgaaatgagccHM560197Squaliusprespensis              ATGGCAAGCCTACGAAAAACCCACCCACTAATAAAAATCGCCAATGACGCACTAGTCGACCTTCCAACACCATCTAATATCTCAGCACTATGAAACTTCGGGTCTCTCCTGGGATTATGTTTAATTACCCAAATCCTAACAGGACTATTCCTAGCAATGCACTATACCTCCGACATCTCAACCGCATTTTCATCAGTAACCCATATCTGCCGGGATGTTAACTACGGCTGACTTATTCGAAGTCTACATGCCAATGGAGCCTCCTTCTTCTTCATCTGTCTTTACATACACATTGCACGAGGACTATATTATGGCTCGTACCTATATAAGGAAACCTGAAACATTGGTGTAGTCCTATTCCTCCTAGTTATGATGACAGCCTTTGTCGGTTACGTACTTCCATGAGGGCAAATGTCCTTTTGGGGTGCCACGGTAATTACGAACCTCCTCTCAGCAGTCCCTTACATAGGAGACACTCTTGTTCAATGAATCTGAGGCGGTTTCTCCGTAGATAACGCAACCCTCACCCGATTCTTCGCGTTCCACTTCCTCCTGCCATTTGTCGTCGCAGGTGCAACCGTCCTACACCTGCTATTTCTACATGAAACGGGATCAAACAACCCAGCCGGGCTAAATTCCGACGCCGACAAAATTTCTTTCCACCCGTACTTCTCATATAAAGACCTTCTTGGCTTCGTTATCATATTATTAGCCCTTACCTCCTTAGCATTATTTTCTCCTAACCTACTAGGTGACCCAGAAAACTTTACCCCAGCAAACCCACTGGTGACACCCCCACATATTCAGCCAGAGTGATATTTCTTGTTTGCCTACGCCATTCTCCGGTCTATTCCAAACAAACTAGGAGGGGTCCTTGCACTACTATTCAGCATCCTAGTGCTAATAGTTGTGCCAATCTTACACACCTCTAAACAACGAGGACTAACTTTCCGCCCCGTAACTCAATTTCTATTTTGAACCCTAGTTGCAGATATACTTATCCTGACATGAATTGGAGGCATACCTGTAGAACACCCATATATCATCATTGGCCAAGTCGCATCCATTTTATATTTTGCGCTATTCCTCGTTCTTGTCCCACTAGCAGGATGAGTGGAAAATAAAGCACTGAAATGAGCCAF090753Squaliusprespensis              atggcaagcctacgaaaaacccacccactaataaaaatcgccaatgacgcactagtcgaccttccaacaccatctaatatctcagcactatgaaacttcgggtctctcctgggattatgtttaattacccaaatcctaacaggactattcctagcgatgcactacacctccgacatctcaaccgcattttcatcagtaacccatatctgccgggatgttaactacggctgacttattcgaagtctacatgccaatggagcctccttcttcttcatctgtctttacatacacattgcacgaggactatattatggctcgtacctatataaggaaacctgaaacattggtgtagtcctattcctcctagttatgatgacagcctttgtcggttacgtacttccatgagggcaaatgtccttttggggtgccacggtaattacgaacctcctctcagcagtcccttacataggagacactcttgttcaatgaatctgaggcggtttctccgtagataacgcaaccctcacccgattcttcgcgttccacttcctcctgccatttgtcgtcgcaggtgcaaccgtcctacacctgctatttctacatgaaacgggatcaaacaacccagccgggctaaattccgacgccgacaaaatttctttccacccgtacttctcatataaagaccttcttggcttcgttatcatattattagcccttacctccttagcattattttctcctaacctactaggtgacccagaaaactttaccccagcaaacccactggtgacacccccacatattcagccagagtgatatttcttgtttgcctacgccattctccggtctattccaaacaaactaggaggggtccttgtactactattcagcatcctagtgctaatagttgtgccaatcttacacacctctaaacaacgaggactaactttccgccccgtaactcaatttctattttgaaccctagttgcagatatacttatcctgacatgaattgggggcatacctgtagaacacccatatatcatcattggccaagtcgcatccattttatattttgcactattcctcgttcttgtcccactagcaggatgagtggaaaataaagcactgaaatgagccHM560198Squaliuspyrenaicus              ATGGCAAGCCTACGAAAAACCCACCCACTAATAAAAATCGCCAACGGCGCACTAGTCGACCTTCCAACACCCCCTAATATCTCAGCACTATGAAACTTCGGATCCCTCCTGGGACTATGCTTAATTACCCAAATCCTGACCGGACTATTCTTAGCAATACACTACACCTCCGACATCTCAACCGCATTTTCATCTGTAACCCATATCTGCCGGGATGTAAACTACGGCTGACTTATTCGAAGCCTACATGCCAATGGGGCATCCTTCTTCTTCATCTGTCTTTATATACATATTGCGCGGGGCCTATATTATGGCTCCTACCTCTATAAAGAAACCTGAAATATCGGCGTAGTCCTGTTTCTCCTGGTCATGATGACGGCTTTCGTCGGCTACGTGCTTCCATGAGGGCAAATGTCCTTTTGAGGTGCCACAGTAATTACAAATCTCCTATCAGCAGTCCCCTACATGGGAGATACCCTTGTTCAATGAATCTGGGGAGGTTTCTCCGTAGACAACGCAACGCTCACCCGATTCTTTGCGTTCCACTTCCTCCTTCCATTTGTCGTTGCAGGCGCAACCATCCTGCACCTATTATTCCTACACGAAACAGGATCAAACAACCCGGCCGGACTCAATTCCGACGCCGACAAAATTTCCTTCCACCCGTACTTCTCATATAAAGACCTTCTTGGCTTTGTTATCATATTACTAGCCCTTACCTCTCTAGCATTGTTTTCCCCNAACCTACTAGGTGACCCAGAAAACTTTACCCCAGCGAACCCGCTAGTAACACCCCCACATATTCAGCCAGAATGATACTTCTTATTTGCCTACGCCATTCTCCGATCTATCCCAAATAAACTAGGAGGGGTCCTTGCACTACTATTTAGCATCCTGGTGCTAATAGTTGTGCCAATTTTACACACCTCGAAACAGCGAGGACTAACTTTCCGCCCCGCAACTCAATTCCTATTCTGAACCCTAGTCGCAGATATATTAATCCTGACATGAATTGGGGGCATACCTGTAGAACACCCATACATTATCATTGGCCAAGTCGCATCCATTCTATACTTTGCACTATTCCTCGTTCTTGTCCCACTAGCAGGGTGAATGGAAAATAAAGCATTGAAATGAGCTHM560199Squaliuspyrenaicus              ATGGCAAGCCTACGAAAAACCCACCCACTAATAAAAATCGCCAACGGCGCACTAGTCGACCTTCCAACACCCTCTAATATCTCAGCACTATGAAACTTCGGATCCCTCCTGGGACTATGCTTAATTACCCAAATCCTGACCGGACTATTCTTAGCAATACACTACACCTCCGACATCTCAACCGCATTTTCATCTGTAACCCATATCTGCCGGGATGTAAACTACGGCTGACTTATTCGAAGCCTACATGCCAACGGGGCATCCTTCTTCTTCATCTGTCTTTATATACATATTGCGCGGGGCCTATATTATGGCTCCTACCTCTATAAAGAAACCTGAAATATTGGCGTAGTCCTATTTCTCCTGGTCATGATGACGGCTTTCGTCGGCTACGTGCTTCCATGAGGGCAAATGTCCTTTTGAGGTGCCACAGTAATTACAAATCTCCTATCAGCAGTCCCCTACATGGGAGATACCCTTGTTCAATGAATCTGGGGAGGTTTCTCCGTAGACAACGCAACGCTCACCCGATTCTTTGCGTTCCACTTCCTCCTTCCATTTGTCGTTGCAGGCGCAACCATCCTGCACCTGTTGTTCCTACACGAAACAGGATCAAACAACCCGGCCGGACTCAATTCCGACGCCGACAAAATTTCCTTCCACCCGTACTTCTCATATAAAGACCTTCTTGGCTTTGTTATCATATTACTAGCCCTTACCTCTCTAGCATTGTTTTCCCCCAACCTACTAGGTGACCCGGAAAACTTTACCCCAGCGAACCCGCTAGTAACACCCCCACATATTCAGCCAGAATGATACTTCTTATTTGCCTACGCCATTCTCCGATCTATCCCAAATAAACTAGGAGGGGTCCTTGCACTACTATTTAGCATCCTGGTGCTAATAGTTGTGCCAATTTTACACACCTCGAAACAGCGAGGACTAACTTTCCGCCCCGCAACTCAATTCCTATTCTGAACCCTAGTCGCAGATATATTAATCCTGACATGAATTGGGGGTATACCTGTAGAACACCCATACATTGTCATTGGCCAAATCGCATCCATTCTATACTTTGCACTATTCCTCGTTCTTGTCCCACTAGCAGGGTGAATGGAAAATAAAGCATTGAAATGAGCTHM560200Squaliussp                      ATGGCAAGCCTACGAAAAACCCATCCACTAATAAAAATCGCCAACGGCGCACTAGTTGACCTCCCAACACCCTCTAATATCTCAGCACTATGAAACTTTGGATCCCTCCTGGGACTATGCTTGATTACCCGAATCCTGACCGGACTATTCTTGGCAATGCACTACACCTCGGACATCTCAACCGCATTTTCATCTGTAACCCATATCTGCCGGGATGTTAACTACGGCTGACTTATTCGAAGCCTACATGCCAATGGAGCATCCTTTTTCTTTATCTGCCTTTATATGCATATTGCGCGGGGCCTATATTATGGCTCCTACCTCTATAAAGAAACCTGAAACATCGGTGTAGTCCTGTTTCTCCTGGTTATAATAACGGCCTTCGTCGGCTACGTACTTCCGTGGGGACAAATATCCTTTTGAGGTGCCACAGTAATTACGAATCTCCTATCAGCAGTCCCCTACATAGGAGACACCCTTGTTCAATGAATCTGAGGAGGTTTCTCCGTAGACAACGCGACGCTCACCCGATTCTTCGCATTCCACTTCCTCCTTCCATTTGTCGTTGCAGGCGCAACCATCTTGCACCTATTATTCCTGCACGAAACAGGATCAAACAACCCAGCCGGGCTAAATTCCGACGCCGACAAAATTTCCTTCCATCCGTACTTTTCATATAAAGACCTTCTTGGCTTTGTTATTATACTGCTAGCCCTCACCTCTCTGGCATTGTTTTCCCCTAACCTACTAGGTGACCCAGAAAACTTTACCCCGGCGAACCCGCTAGTAACTCCCCCACATATTCAGCCAGAGTGATACTTCTTGTTTGCCTATGCCATCCTCCGGTCTATCCCAAACAAATTAGGAGGGGTCCTTGCGCTACTATTCAGCATTCTAGTGCTATTGGTTGTGCCAATTTTACACACCTCAAAGCAACGAGGACTAACGTTCCGCCCCGCAACCCAGTTCCTATTCTGAACCCTCGTTGCGGATATACTAATCCTGACATGAATTGGGGGCATACCTGTAGAACATCCGTACATCATCATTGGTCAGATCGCATCCATTCTGTACTTTGCACTGTTCCTCGTTCTTGTTCCACTAGCAGGGTGAATGGAAAATAAAGCATTAAAATGAGCTHM560201Squaliussp                      ATGGCAAGCCTACGAAAAACCCATCCACTAATAAAAATCGCCAACGGCGCACTAGTTGACCTCCCAACACCCTCTAATATCTCAGCACTATGAAACTTTGGATCCCTCCTGGGACTATGCTTGATTACCCAAATCCTGACCGGACTATTCTTGGCAATGCACTACACCTCGGACATCTCAACCGCATTTTCATCTGTAACCCATATCTGCCGGGATGTTAACTACGGCTGACTTATTCGAAGCCTACATGCCAATGGAGCATCCTTTTTCTTTATCTGTCTTTATATGCATATTGCGCGGGGCCTATATTATGGCTCTTACCTCTATAAAGAAACCTGAAACATCGGTGTAGTCCTGTTTCTCCTGGTTATAATAACGGCCTTCGTCGGCTACGTACTTCCGTGGGGACAAATATCCTTTTGAGGTGCCACAGTAATTACGAATCTCCTATCAGCAGTCCCCTACATAGGAGACACCCTTGTTCAATGAATCTGGGGAGGTTTCTCCGTAGACAACGCGACGCTCACCCGGTTCTTCGCATTCCACTTCCTCCTTCCATTTGTCGTTGCAGGCGCAACCATCTTGCACCTATTATTCCTGCACGAAACAGGATCAAACAACCCAGCCGGGCTAAATTCCGACGCCGACAAAATTTCCTTCCATCCGTACTTTTCATATAAAGACCTTCTTGGCTTTGTTATTATACTGCTAGCCCTCACCTCTCTGGCATTGTTTTCCCCTAACCTACTAGGTGACCCAGAAAACTTTACCCCGGCGAACCCGCTAGTAACTCCCCCACATATTCAGCCAGAGTGATACTTCTTGTTTGCCTATGCCATCCTCCGGTCTATCCCAAACAAATTAGGAGGGGTCCTTGCGCTACTATTCAGCATTCTAGTGCTATTGGTTGTGCCAATTTTACATACCTCAAAGCAACGAGGACTAACGTTCCGCCCCGCAACCCAGTTCCTATTCTGAACCCTCGTTGCGGATATACTAATCCTGACATGAATTGGGGGCATACCCGTAGAACATCCGTACATCATCATTGGTCAGATCGCATCCATTCTGTACTTTGCACTGTTCCTCGTTCTTGTTCCACTAGCAGGGTGAATGGAAAATAAAGCATTAAAATGAGCTAJ252811Squaliussp                      atggcaagcctacgaaaaacccacccactaatgaaaatcgccaatggcgcactagtcgaccttccaacaccatctaatatctcagcaatatgaaacttcgggtctctcctaggattatgtttaattacccaaatcctaacgggactattcttagcgatgcactacacctccgacatctcgaccgcattttcgtcagtagcccacatctgccgggatgtaaactacggctgacttattcgaagtctgcatgccaatggagcctccttcttcttcatctgtctttacatgcacattgcacgaggactatactatggctcatacctatataaagagacctgaaacattggtgtggtcctgttcctcctagttataataacagcctttgtcggctacgtacttccgtgaggacagatgtctttttgaggcgccacggtaattacgaacctgctctcagcagtcccttacatgggggacatgcttgttcaatgaatctgaggtggtttctcagtagataatgcaacccttacccgattcttcgcattccacttcctcctgccgtttgtcgtcgcaggcgcaaccatcctgcacctgctgtttctacacgaaacaggatcaaacaacccagccggactaaactccgacgccgacaaaatttctttccacccatacttctcatataaagaccttcttggctttgttatcatattactagcccttacctctctagcattattttctcccaacctactgggtgacccagaaaactttaccccagcaaacccactagtgacgcccccacatattcagccagagtggtacttcttatttgcctacgccattctccggtctatcccaaacaagctaggaggggtcctggcgctactattcagcatcctagtgctactagttgtgccaatcttacacacctcaaaacaacgaggactaactttccgccccgtaactcaattcctattctgaaccctagttgcagatatatttatcctgacatgaattgggggcatacctgtagaacacccatatattatcattggccaagtcgcatccattctatactttgcgctcttcctcgtccttgtcccgctagcaggatgagtagaaaataaagcactgaaatgagccHM560203Squaliussp                      ATGGCAAGCCTACGAAAAACCCACCCACTGATAAAAATCGCCAACGGCGCACTAGTCGACCTTCCAACACCATCTAATATCTCAGCACTATGAAACTTCGGATCTCTTCTAGGATTATGTTTAATTACCCAAATCCTAACAGGACTGTTCTTAGCAATGCACTACACCTCTGACATCTCAACCGCATTTTCATCGGTAACCCACATCTGCCGAGATGTTAACTACGGCTGACTTATTCGGAGCCTGCATGCCAATGGAGCCTCCTTCTTCTTCATCTGTCTTTATATACACATTGCACGAGGACTATATTATGGCTCATACCTATATAAAGAAACCTGAAACATTGGTGTAGTTCTATTTCTCCTAGTTATGATGACAGCCTTTGTCGGCTACGTACTTCCATGGGGGCAAATGTCCTTTTGAGGTGCCACGGTAATTACAAACCTCCTCTCAGCAGTCCCTTACATGGGGGACACTCTTGTTCAATGAATCTGAGGCGGTTTCTCCGTAGATAACGCAACCCTCACTCGATTCTTCGCATTCCACTTCCTCCTGCCATTTGTCGTCGCAGGCGCAACCATTCTGCACCTGCTGTTTCTACACGAAACGGGATCAAACAACCCAGCCGGACTAAATTCCGACGCCGACAAAATTTCTTTCCACCCGTACTTCTCATATAAAGACCTTCTTGGCTTTGTTATTATACTATTAGCCCTTACCTCTCTAGCACTATTTTCTCCTAACCTACTAGGTGACCCAGAAAACTTTACCCCAGCAAACCCACTGGTAACGCCTCCACATATTCAGCCAGAGTGATACTTCTTGTTTGCCTACGCCATTCTCCGGTCTATCCCAAACAAACTAGGAGGGGTACTTGCACTACTATTCAGCATCCTAGTGCTAATGGTTGTACCAATCTTACACACCTCTAAACAACGAGGACTAACTTTCCGCCCCGTAACTCAATTCCTATTCTGAACCCTGGTTGCAGATATATTTATCCTGACATGAATTGGGGGCGTACCTGTAGAACACCCATATATTATCATTGGCCAAGTCGCATCCATTCTATATTTTGCACTCTTCCTCATCCTTGTCCCACTAGCAGGATGAGTGGAAAATAAAGCATTGAAATGAGCCAJ252805Squaliussp                      atggcaagcctacgaaaaacccacccactgataaaaatcgccaacggcgcactagtcgaccttccaacaccatctaatatctcagcactatgaaacttcggatctcttctaggattatgtttaattacccaaatcctaacaggactgttcttagcaatgcactacacctctgacatctcaaccgcattttcatcggtaacccacatctgccgagatgttaactacggctgacttattcggagcctgcatgccaatggagcctccttcttcttcatctgtctttatatacacattgcacgaggactatattatggctcatacctatataaagaaacctgaaacattggtgtagtcctatttctcctagttatgatgacagcctttgtcggctacgtacttccatgggggcaaatgtccttttgaggtgccacggtaattacaaacctcctctcagcagtcccttacatgggggacactcttgttcaatgaatctgaggcggtttctccgtagataacgcaaccctcactcgattcttcgcattccacttcctcctgccatttgtcgtcgcaggcgcaaccattctgcacctgctgtttctacacgaaacgggatcaaacaacccagccggactaaattccgacgccgacaaaatttctttccacccgtacttctcatataaagaccttcttggctttgttattatactattagcccttacctctctagcactattttctcctaacctactaggtgacccagaaaactttaccccagcaaacccactggtaacgcctccacatattcagccagagtgatacttcttgtttgcctacgccattctccggtctatcccaaacaaactaggaggggtacttgcactactattcagcatcctagtgctaatggttgtaccaatcttacacacctctaaacaacgaggactaactttccgccccgtaactcaattcctattctgaaccctggttgcagatatatttatcctgacatgaattgggggcatacctgtagaacacccatatattatcattggccaagtcgcatccattctatattttgcactcttcctcatccttgtcccactagcaggatgagtggaaaataaagcattgaaatgagccAF090755Squaliuscforpheus               atggcaagcctacgaaaaacccacccactaataaaaatcgccaatgacgcactagtcgaccttccaacaccatctaatatctcagcaatatgaaacttcggatctctcctaggattatgtttaattacccaaatcctaacgggactattcctagcaatgcactatacctctgacatctcaaccgcattttcatccgtaacccacatctgccgagatgttaactacggctgacttattcggagtctgcatgccaatggagcctccttcttcttcatctgtctttatatgcacattgcacggggactatattatggctcatacctatataaagaaacctgaaacattggtgtagtcctgtttctcctggttataatgacggcctttgtcggctacgtgcttccatgagggcaaatgtccttttgaggtgctacagtaattacgaacctcctctcagcagtcccttacataggggatactcctgttcaatgaatctgaggcggtttctccgtagacaacgcgaccctcacccgattcttcgcgttccacttcctcctgccatttgtcgtaacaggcgcaaccatcctgcacctgctatttctacacgaaacggggtcaaacaacccagccggactaaattccgacgccgacaaaatttctttccacccgtacttctcatataaagaccttcttggctttgttattatattactagcccttgcctccctagcactattttctcctaacctactaggtgacccagaaaactttaccccagcaaacccactggtgacgcccccacatattcaaccagaatgatacttcttgtttgcctacgccattctccgatctattcccaacaagctaggaggggtccttgcactactattcagcatcctggtgctaatagttgtgccaatcttacacacctctaaacaacgaggactaactttccgccccgtaactcaattcctattctgaaccctagttgcggatatacttatcctgacatgaattgggggcatacctgtagaacacccatatattatcattggccaagtcgcatccattctatattttgcgctcttcctcgttcttatcccgctagcaggatgagtagaaaataaagcattgaaatgagccHM560202Squaliusaffvardarensis          ATGGCAAGCCTACGAAAAACCCACCCACTGATAAAAATCGCCAATGACGCACTAGTCGACCTTCCAACACCATCTAATATCTCAGCACTGTGAAACTTCGGATCTCTTCTAGGATTATGTTTAATTACCCAAATCCTAACAGGACTATTCTTAGCAATGCACTACACCTCCGACATCTCAACCGCATTTTCATCGGTAACCCACATCTGCCGGGATGTTAACTACGGCTGACTTATTCGAAGCCTACATGCCAATGGAGCCTCCTTCTTCTTCATCTGTCTTTATATACACATTGCACGAGGACTATATTATGGCTCATACCTATATAAAGAAACCTGAAACATTGGTGTAGTCCTGTTTCTCCTAGTTATAATGACAGCCTTTGTCGGCTACGTACTTCCGTGAGGACAAATATCCTTTTGAGGTGCCACAGTAATTACAAACCTCCTCTCAGCAGTCCCTTACATGGGGGACACTCTTGTTCAATGAATCTGAGGCGGTTTCTCCGTAGATAACGCAACCCTCACCCGATTCTTCGCATTCCACTTCCTCCTGCCATTTGTCGTCGCAGGCGCAACCATTCTACACCTGCTATTTCTACACGAAACGGGATCAAACAACCCAGCCGGACTAAATTCCGACGCCGACAAAATTTCTTTCCACCCGTACTTCTCATATAAAGACCTTCTTGGCTTTTGTATTATACTACTAGCCCTTACCTCTCTAGCACTATTTTCTCCTAACCTACTAGGTGACCCAGAAAACTTTACCCCAGCAAACCCACTAGTAACACCCCCACATATTCAGCCAGAGTGATACTTCTTATTTGCCTACGCCATTCTCCGATCTATCCCAAACAAACTAGGAGGGGTACTTGCACTACTATTCAGCATCCTAGTGCTAATAGTTGTGCCAATCTTACACACCTCTAAACAACGAGGACTAACTTTCCGCCCCGTAACTCAATTCCTATTCTGAACCCTAGTTGCAGATATATTTATCCTGACATGAATTGGGGGCATACCTGTAGAACACCCATATATTATCATTGGCCAAATCGCATCCATTCTATATTTTGCACTCTTCCTCATTCTTGTCCCGCTAGCAGGATGAGTGGAAAATAAAGCATTAAAATGAGCCHM560204Squaliussqualus                 ATGGCAAGCCTACGAAAAACCCACCCACTAATAAAAATCGCCAATGACGCACTAGTCGACCTTCCGACACCATCTAATATCTCAGCACTATGAAACTTCGGGTCTCTCCTGGGATTATGTTTAATTACCCAAATCCTAACAGGACTATTCCTAGCAATGCACTACACCTCCGACATCTCAACCGCATTTTCATCGGTAACCCATATCTGCCGAGATGTTAACTACGGCTGACTTATTCGAAGTCTACATGCCAATGGAGCCTCCTTCTTCTTCATCTGTCTTTACATACACATTGCACGAGGACTATATTATGGCTCGTACCTATATAAGGAAACCTGAAACATTGGTGTAGTCCTATTTCTCCTAGTTATAATGACAGCCTTTGTCGGTTACGTGCTTCCATGAGGACAAATGTCCTTTTGGGGTGCCACGGTAATTACAAACCTCCTCTCAGCAGTCCCTTACATAGGGGACACTCTTGTTCAATGAATCTGAGGCGGATTCTCCGTAGATAACGCAACCCTCACCCGATTCTTCGCGTTCCACTTCCTCCTGCCATTTGTCGTCGCAGGTGCAACCGTCCTACACCTGCTATTTCTACATGAAACAGGATCAAACAACCCAGCCGGGCTAAATTCCGACGCCGACAAAATTTCTTTCCACCCGTACTTCTCATGTAAAGACCTTCTTGGCTTCGTTATCATATTATTAGCCCTTACCTCCTTAGCATTATTTTCTCCTAACCTACTAGGTGACCCAGAAAACTTTaCCCCAGCAAACCCACTGGTGACGCCCCCACATATTCAGCCAGAATGATATTTCTTGTTTGCCTACGCCATTCTCCGATCTATTCCAAACAAACTAGGAGGGGTCCTTGCACTACTATTTAGCATCCTAGTGCTAATAGTTGTGCCAATCTTACACACCTCTAAACAACGAGGACTAACTTTCCGCCCCGTAACTCAATTTCTATTTTGAACCCTAGTTGCAGATATACTTATTCTGACATGAATTGGGGGCATACCTGTAGAACACCCATATATCATCATTGGCCAAGTCGCATCCATTCTATATTTTGCGCTCTTCCTCGTTCTTGTCCCACTAGCAGGATGAGTGGAAAATAAAGCACTGAAATGAGCC     HM560205Squaliussqualus                 ATGGCAAGCCTACGAAAAACCCACCCACTAATAAAAATCGCCAATGACGCACTAGTCGACCTTCCGACACCATCTAATATCTCAGCACTATGAAACTTCGGGTCTCTCCTGGGATTATGTTTAATTACCCAAATCCTAACGGGACTATTCCTAGCAATGCACTACACCTCCGACATCTCAACCGCATTTTCATCGGTAACCCATATCTGCCGAGATGTTAACTACGGCTGACTTATTCGAAGTCTACATGCCAATGGAGCCTCCTTCTTCTTCATCTGTCTTTACATACACATTGCACGAGGACTATATTATGGCTCGTACCTATATAAGGAAACCTGAAACATTGGTGTAGTCCTATTTCTCCTAGTTATAATGACAGCCTTTGTCGGTTACGTACTTCCATGAGGACAAATGTCCTTTTGGGGTGCCACGGTAATTACAAACCTCCTCTCAGCAGTCCCTTACATAGGGGACACTCTTGTTCAATGAATCTGAGGCGGATTCTCCGTAGATAACGCAACCCTCACCCGATTCTTCGCGTTCCACTTCCTCCTACCATTTGTCGTCGCAGGTGCNACCGTCCTACACCTGTTATTTCTACATGAAACAGGATCAAACAACCCAGCCGGGCTAAATTCCGACGCCGACAAAATTTCTTTCCACCCGTACTTCTCATATAAAGACCTTCTTGGCTTCGTTATCATATTATTAGCCCTTACCTCCTTAGCATTATTTTCTCCTAACCTACTAGGTGACCCGGAAAACTTTACCCCAGCAAACCCACTAGTGACACCCCCACATATTCAACCAGAATGATATTTCTTGTTTGCCTACGCCATTCTCCGATCTATTCCAAACAAACTAGGAGGGGTCCTTGCACTACTATTTAGCATCCTAGTGCTAATAGTTGTGCCGATCTTACACACCTCTAAACAACGAGGACTAACTTTCCGCCCCGTAACTCAATTTCTATTTTGAACCCTAGTTGCAGATATACTTATCCTGACATGAATTGGGGGCATACCTGTAGAACACCCATATATCATTATTGGCCAAGTCGCATCCATTCTATATTTTGCGCTCTTCCTCATTCTTGTCCCACTAGCAGGATGAGTAGAAAATAAAGCACTGAAATGAGCCHM560206Squaliussvallize                ATGGCAAGCCTACGAAAAACTCACCCACTAATAAAAATCGCCAACGGCGCACTAGTTGACCTCCCAACACCATCTAATATCTCAGCACTATGAAACTTCGGTTCCCTCCTAGGATTATGTTTAATTACCCAAATCCTCACGGGACTATTCTTAGCAATGCACTACACCTCCGACATCTCAACCGCATTTTCATCTGTGACCCATATCTGCCGAGATGTTAACTATGGCTGACTTATTCGAAATCTGCATGCCAATGGGGCATCCTTCTTCTTCATTTGCCTTTACATGCATATTGCACGGGGCCTATATTATGGCTCGTACCTATACAAAGAGACCTGAAACATCGGTGTAGTGCTGTTTCTCCTAGTTATAATGACAGCCTTCGTCGGCTACGTACTTCCATGGGGACAAATGTCCTTTTGAGGTGCCACAGTAATTACAAATCTCCTCTCAGCAGTCCCCTACATAGGGGATACTCTTGTTCAATGAATCTGAGGCGGTTTCTCCGTAGACAACGCAACCCTCACCCGATTCTTCGCATTCCACTTCCTCCTGCCGTTTGTCGTCGCGGGCGCAACCATCCTGCACCTGTTATTCCTACACGAAACAGGATCGAACAACCCAGCCGGGCTAAATTCTGACGCCGACAAGATTTCTTTCCACCCGTACTTCTCATATAAAGACCTTCTTGGCTTTGTTATCATGTTACTAGCCCTTACCTCTCTAACATTATTTTCTCCCAACCTATTAGGTGACCCAGAGAATTTTACCCCAGCAAACCCACTAGTAACACCCCCACACATTCAGCCAGAGTGCTACTTCTTATTTGCCTACGCCATCCTTCGATCTATTCCAAACAAACTAGGAGGGGTCCTTGCACTATTATTCAGCATCCTAGTGCTAATGGTTGTGCCAATTTTACATACCTCAAAACAACGAAGACTAACTTTCCGCCCCGTAACTCGATTCTTCTTTTGAACCCTTGTTGCAGATATATTAATCGTAACATGACTTGGGGGCATACCTGTAGAACACCCATATATTATCATTGGCCAAGTCGCATCCATTTTATACTTTACACTATTCCTCATTCTTGTCCCACTGGCGGGGTGAATAGAAAATAAAGCATTGAAATGAGCCHM560207Squaliussvallize                ATGGCAAGCCTACGAAAAACTCACCCACTAATAAAAATCGCCAACGGCGCACTAGTTGACCTCCCAACACCATCTAATATCTCAGCACTATGAAACTTCGGTTCCCTCCTAGGATTATGTTTAATTACCCAAATCCTCACGGGACTATTCTCAGCAATGCACTACACCTCCGACATCTCAACCGCATTTTCATCTGTGACCCATATCTGCCGAGATGTTAACTATGGCTGACTTATTCGAAATCTGCATGCCAATGGGGCATCCTTCTTCTTCATTTGCCTTTACATGCATATTGCACGGGGCCTATATTATGGCTCGTACCTATACAAAGAGACCTGAAACATCGGTGTAGTACTGTTTCTCCTAGTTATAATGACAGCCTTCGTCGGCTACGTACTTCCATGGGGACAAATGTCCTTTTGAGGTGCCACAGTAATTACAAATCTCCTCTCAGCAGTCCCCTACATAGGGGATACTCTTGTTCAATGAATCTGAGGCGGTTTCTCCGTAGACAACGCAACCCTCACCCGATTCTTCGCATTCCACTTCCTCCTGCCGTTTGTCGTCGCGGGCGCAACCATCCTGCACCTGTTATTCCTACACGAAACAGGATCGAACAACCCAGCCGGGcTAAATTCTGACGCCGACAAGATTTCTTTCCACCCGTACTTCTCATATAAAGACCTTCTTGGCTTTGTTATCATGTTACTAGCCCTTACCTCTCTAACATTATTTTCTCCCAACCTATTAGGTGACCCAGAGAATTTTACCCCAGCAAACCCACTAGTAACACCCCCACACATTCAGCCAGAGTGGTACTTCTTATTTGCCTACGCCATCCTTCGATCTATTCCAAACAAACTAGGAGGGGTCCTTGCACTATTATTCAGCATCCTAGTGCTAATGGTTGTGCCAATTTTACATACCTCAAAACAACGAGGACTAACTTTCCGCCCCGTAACTCAATTCCTATTCTGAACCCTAGTTGCAGATATATTAATCCTGACATGAATTGGGGGCATACCTGTAGAACACCCATATATTATCATTGGCCAAGTCGCATCCATTTTATACTTTGCACTATTCCTCATTCTTGTCCCACTGGCGGGGTGAATAGAAAATAAAGCATTGAAATGAGCCHM560208Squaliustenellus                ATGGCAAGCCTACGAAAAACCCACCCACTAATAAAAATCGCCAACGGCGCACTAGTCGACCTTCCGACACCGTCTAATATCTCAGCACTATGAAACTTCGGTTCCCTCCTAGGATTATGTTTAATTACCCAGATCCTGACAGGACTATTCTTGGCAATGCACTACACCTCCGACATCTCAACCGCATTTTCATCTGTCACCCATATCTGCCGAGATGTTAACTACGGCTGACTTATTCGAAATCTGCATGCCAATGGAGCATCCTTCTTCTTCATCTGTCTTTACATACATATTGCACGGGGCCTATATTATGGCTCATACCTATACAAAGAAACCTGAAACATTGGTGTAGTCCTGTTTCTCCTAGTTATAATGACAGCCTTCGTCGGCTACGTACTTCCATGAGGACAAATGTCCTTTTGAGGTGCCACAGTAATTACAAATCTCCTCTCAGCAGTCCCTTACATGGGGGATACTCTTGTTCAATGAATCTGAGGCGGTTTCTCCGTAGACAACGCAACCCTCACCCGATTCTTCGCATTCCACTTCCTCCTACCATTTGTCGTCGCAGGCGCAACCATCCTGCACCTGTTATTCCTACACGAAACAGGATCGAACAACCCAGCCGGACTAAATTCTGACGCCGACAAAATCTCTTTCCACCCGTACTTCTCATATAAAGACCTTCTTGGCTTTGTTATCATATTACTAGCCCTTACCTCTTTAACATTATTTTCTCCCAACCTATTAGGTGACCCAGAGAACTTTACCCCAGCAAACCCACTGGTGACGCCCCCACATATTCAGCCAGAATGGTATTTCTTATTTGCCTACGCCATCCTCCGATCTATTCCAAATAAACTAGGAGGGGTCCTTGCACTACTGTTCAGCATCCTAGTGCTAATAGTTGTGCCAATTTTACACACCTCAAAACAACGAGGACTAACTTTCCGCCCTATAACTCAATTCCTATTCTGAACCCTAGTTGCAGATATATTTATTCTGACATGAATCGGGGGCATACCTGTAGAACACCCATATATTATCATTGGCCAAGTCGCATCTATTTTATATTTTGCACTATTCCTCGTTCTTGTCCCACTGGCAGGGTGAATAGAAAATAAAGCATTGAAATGAGCCHM560209Squaliustenellus                ATGGCAAGCCTACGAAAAACCCACCCACTAATAAAAATCGCCAACGGCGCACTAGTCGACCTTCCGACACCGTCTAATATCTCAGCACTATGAAACTTCGGTTCCCTCCTAGGATTATGTTTAATTACCCAGATCCTGACAGGACTATTCTTGGCAATGCACTACACCTCCGACATCTCAACCGCATTTTCATCTGTCACCCATATCTGCCGAGATGTTAACTACGGCTGACTTATTCGAAATCTGCATGCCAATGGAGCATCCTTCTTCTTCATCTGTCTTTACATACATATTGCACGGGGCCTATATTATGGCTCATACCTTTACAAAGAAACCTGAAACATTGGTGTAGTCCTGTTTCTCCTAGTTATAATGACAGCCTTCGTCGGCTACGTACTTCCATGAGGACAAATGTCCTTTTGAGGTGCCACAGTAATTACAAATCTCCTCTCAGCAGTCCCTTACATAGGGGATACTCTTGTTCAATGAATCTGAGGCGGTTTCTCCGTAGACAACGCAACCCTCACCCGATTCTTCGCATTCCACTTCCTCCTACCATTTGTCGTCGCAGGCGCAACCATCCTGCACCTGTTATTCCTACACGAAACAGGATCGAACAACCCAGCCGGACTAAATTCTGACGCCGACAAAATCTCTTTCCACCCGTACTTCTCATATAAAGACCTTCTTGGCTTTGtTATCATATTACTAGCCCTTACCTCTTTAACATTATTTTCTCCCAACCTATTAGGTGACCCAGAGAACTTTACCCCAGCAAACCCACTGGTGACACCCCCACATATTCAGCCAGAATGGTATTTCTTATTTGCCTACGCCATCCTCCGATCTATTCCAAATAAACTAGGAGGGGTCCTTGCACTACTGTTCAGCATCCTAGTGCTAATAGTTGTGCCAATTTTACACACCTCAAAACAACGAGGACTAACTTTCCGCCCTATAACTCAATTCCTATTCTGAACCCTAGTTGCAGATATATTTATTCTGACATGAATCGGGGGCATACCTGTAGAACACCCATATATTATCATTGGCCAAGTCGCATCTATTTTATATTTTGCACTATTCCTCGTTCTTGTCCCACTGGCAGGGTGAATAGAAAATAAAGCATTGAAATGAGCCDQ061934Squaliustorgalensis             NNNNNNNNNNNNNNNNNNNNNNNNNNNNNNNNNNNNNNNNNNNNNNNNNNNNNNgttgaccttccaacaccatctaacatctcagcactatgaaatttcgggtccctcctaggattatgtttaattacccaaatcctcacaggactattcctagcgatgcattacacctccgacatctcaaccgcattttcatctgtaacccacatttgccgagatgtaaacNacggctgacttattcgaagtctgcatgccaacggagcatcattcttctttatttgcctcTatatacacattgcacgaggcctatattacggctcatacctatataaagaaacctgaaacatcggtgtaatcctatttctcctagttataataacagccttcgtcggctacgtacttccgtgagggcaaatgtccttttgaggtgctacagtaattactaacctcctatcagcagtcccttacataggagatacccttgttcaatgaatctggggcggtttttccgtagacaacgcaacacttacccgattcttcgcattccacttcctcctgccgtttatcgtcgcaggcgcaaccgtcctacacctattattcctacacgaaacaggatcgaacaacccagctgggctaaattccgacgccgacaaaatttctttccacccatacttctcttataaagaccttcttggcttcgttatcatgttactagccctcgcctctctaacgctattttcccccaacctgctaggcgacccagagaactttaccccagcgaaccccctggtaacacccccgcatattcagccagaatgatacttcctatttgcctatgccatcctccggtctattccaaacaaactgggaggggtcctcgcgctgctgttcagcatcctagtgctaatagttgtaccaattttacacacctcaaagcagcgaggactaacttttcgccccgtgacgcaattcttattctgaaccctggttgcagatatatttatcctaacgtgaattgggggcatacctgtagaacacccatacattgtcattggccaaatcgcatccattctatattttgcgctattcctcgttcttgtcNNNNNNNNNNNNNNNNNNNNNNNNNNNNNNNNNNNNNNNNNNHM560210Squaliustorgalensis             ATGGCAAGCCTACGGAGGacCcACCCactGATAAAGAtcgCCAACGACGCACTAGTTGACCTTCCAACACCATCTAACATCTCAGCACTATGAAATTTCGGGTCCCTCCTAGGATTATGTTTAATTACCCAAATCCTCACAGGACTATTCCTAGCGATGCATTACACCTCCGACATCTCAACCGCATTTTCATCTGTAACCCATATTTGCCGAGATGTAAACTACGGCTGACTTATTCGAAGTCTGCATGCCAACGGAGCATCATTCTTCTTTATTTGCCTCTATATACACATTGCACGAGGCCTATATTACGGCTCATACCTATATAAAGAAACCTGAAACATCGGTGTAATCCTATTTCTCCTAGTTATAATAACAGCCTTCGTCGGCTACGTACTTCCGTGAGGGCAAATGTCCTTTTGAGGTGCTACAGTAATTACTAACCTCCTATCAGCAGTCCCTTACATAGGAGATACCCTTGTTCAATGAATCTGGGGCGGTTTTTCCGTAGACAACGCAACACTTACCCGATTCTTCGCATTCCACTTCCTCCTGCCGTTTATCGTCGCAGGCGCAACCGTCCTACACCTATTATTCCTACACGAAACAGGATCGAACAACCCAGCTGGGCTAAATTCCGACGCCGACAAAATTTCTTTCCACCCATACTTCTCTTATAAAGACCTTCTTGGCTTCGTTATCATGTTACTAGCCCTCGCCTCTCTAACGCTATTTTCCCCCAACCTGCTAGGCGACCCAGAGAACTTTACCCCAGCGAACCCCCTGGTAACACCCCCGCATATTCAGCCAGAATGATACTTCCTATTTGCCTATGCCATCCTCCGGTCTATTCCAAACAAACTGGGAGGGGTCCTCGCGCTGCTGTTCAGCATCCTAGTGCTAATAGTTGTACCAATTTTACACACCTCAAAGCAGCGAGGACTAACTTTTCGCCCCGTGACGCAATTCTTATTCTGAACCCTGGTTGCAGATATATTTATCCTAACGTGAATTGGGGGCATACCTGTAGAACACCCATACATTGTCATTGGCCAAATCGCATCCATTCTATATTTTGCGCTATTCCTCGTTCTTGTCCCACTAGCAGGATGGGCCGAGAATAAgGCATTGAAAtGAGCTHM560211Squaliusvalentinus              ATGGCAAGCCTACGAAAAACCCACCCACTAATAAAAATCGCCAACGGCGCACTAGTCGATCTTCCAACACCCTCTAATATCTCAGCACTATGAAACTTCGGATCCCTCCTGGGACTATGCTTAATTACCCAAATCCTGACCGGATTATTCTTAGCAATGCACTACACCTCCGACATCTCAACCGCATTTTCATCTGTAACCCATATCTGCCGGGATGTAAACTACGGCTGACTTATTCGAAGCCTACATGCCAATGGGGCATCCTTCTTCTTCATCTGTCTTTATATACATATTGCGCGGGGCCTGTACTATGGCTCCTACCTCTATAAAGAAACCTGAAATATCGGCGTAGTCCTGTTTCTCCTGGTTATGATGACGGCCTTCGTCGGCTACGTGCTTCCGTGGGGACAAATGTCCTTTTGAGGTGCCACAGTAATTACAAATCTCCTATCAGCAGTCCCCTACATGGGGGATACCCTTGTTCAATGAATCTGGGGAGGTTTCTCCGTAGATAACGCAACGCTCACCCGATTCTTTGCGTTCCATTTCCTCCTTCCATTTATCGTTGCAGGCGCAACCATCCTGCACCTGTTATTCCTACACGAAACAGGATCAAACAACCCGGCCGGACTCAATTCCGACGCTGACAAAATTTCCTTCCATCCGTACTTCTCATATAAAGACCTTCTTGGCTTTGTTATCATATTACTAGCCCTTACCTCTCTAGCATTGTTTTCCCCCAACCTACTGGGTGACCCGGAAAACTTTACCCCAGCGAACCCGCTAGTAACACCCCCACATATTCAGCCAGAATGATACTTCTTATTTGCCTACGCCATTCTCCGATCTATCCCAAATAAACTAGGAGGGGTCCTTGCACTACTATTCAGCATCCTGGTGCTAATAGTTGTGCCAATTTTACACACCTCAAAACAGCGAGGACTAACTTTCCGCCCCGCAACTCAGTTCCTATTCTGAACCCTAGTCGCAGATATATTAATCCTGACATGAATTGGGGGCATACCTGTAGAACACCCATACATTGTCATTGGCCAAGTCGCATCCATTCTATACTTTGCGCTATTTCTCGTTCTTGTCCCACTAGCAGGGTGAATGGAAAATAAAGCATTAAAATGAGCTHM560212Squaliusvalentinus              ATGGCAAGCCTACGAAAAACCCACCCACTAATAAAAATCGCCAACGGCGCACTAGTCGATCTTCCAACACCCTCTAATATCTCAGCACTATGAAACTTCGGATCCCTCCTGGGACTATGCTTAATTACCCAAATCCTGACCGGATTATTCTTAGCAATGCACTACACCTCCGACATCTCAACCGCATTTTCATCTGTAACCCATATCTGCCGGGATGTAAACTACGGCTGACTTATTCGAAGCCTACATGCCAATGGGGCATCCTTCTTCTTCATCTGTCTTTATATACATATTGCGCGGGGCCTGTACTATGGCTCCTACCTCTATAAAGAAACCTGAAATATCGGCGTAGTCCTGTTTCTCCTGGTTATGATGACGGCCTTCGTCGGCTACGTGCTTCCGTGGGGACAAATGTCCTTTTGAGGTGCCACAGTAATTACAAATCTCCTATCAGCAGTCCCCTACATGGGGGATACCCTTGTTCAATGAATCTGGGGAGGTTTCTCCGTAGATAACGCAACGCTCACCCGATTCTTTGCGTTCCATTTCCTCCTTCCATTTATCGTTGCAGGCGCAACCATCCTGCACCTGTTATTCCTACACGAAACAGGATCAAACAACCCGGCCGGACTCAATTCCGACGCTGACAAAATTTCCTTCCATCCGTACTTCTCATATAAAGACCTTCTTGGCTTTGTTATCATATTACTAGCCCTTACCTCTCTAGCATTGTTTTCCCCCAACCTACTGGGTGACCCGGAAAACTTTACCCCAGCGAACCCGCTAGTAACACCCCCACATATTCAGCCAGAATGATACTTCTTATTTGCCTACGCCATTCTCCGATCTATCCCAAATAAACTAGGAGGGGTCCTTGCACTACTATTCAGCATCCTGGTGCTAATAGTTGTGCCAATTTTACACACCTCAAAACAGCGAGGACTAACTTTCCGCCCCGCAACTCAGTTCCTATTCTGAACCCTAGTCGCAGATATATTAATCCTGACATGAATTGGGGGCATACCTGTAGAACACCCATACATTGTCATTGGCCAAGTCGCATCCATTCTATACTTTGCGCTATTTCTCGTTCTTGTCCCACTAGCAGGGTGAATGGAAAATAAAGCATTAAAATGAGCTAF090754Squaliusvardariensis            atggcaagcctacgaaaaacccacccactgataaaaatcgccaatgacgcactagtcgaccttccaacaccatctaatatctcagcactatgaaacttcggatctcttctaggattatgtttaattacccaaatcctaacaggactattcttagcgatgcactacacctccgacatctcaaccgcattttcatcggtaacccacatctgccgggatgttaactacggctgacttattcgaagcctacatgccaatggagcctccttcttcttcatctgtctttatatacacattgcacgaggactatattatggctcatacctatataaagaaacctgaaacattggtgtagtcctgtttctcctagttataatgacagcctttgtcggctacgtacttccgtgaggacaaatatccttttgaggtgccacagtaattacaaacctcctctcagcagtcccttacatgggggacactcttgttcaatgaatctgaggcggtttctccgtagataacgcaaccctcacccgattcttcgcattccacttcctcctgccatttgtcgtcgcaggcgcaaccattctacacctgctatttctacacgaaacgggatcaaacaacccagccggactaaattccgacgcggacaaaatttctttccacccatacttctcatataaagaccttcttggctttgttattatactactagcccttacctctctagcactattttctcctaacctactaggtgacccagaaaactttaccccagcaaacccactagtaacacccccacatattcagccagagtgatacttcttgtttgcctacgccattctccgatctatcccaaacaaactaggaggggtacttgcagtactactcagcatcctggtgctaatagttgtgccgatcttacacacctctaaacaacgaggactaactttccgccccgtaactcaattcctattctgaaccctagttgcagatatatttatcctgacatgaattgggggcatacctgtagaacacccatatattatcattggccaagtcgcatccattctatattttgcactcttcctcattcttgtcccactagcaggatgagtggaaaataaagcattaaaatgagccAJ251093Squaliuszrmanjae                atggcaagcctacgaaaaactcacccactaataaaaatcgccaacggcgcactagttgaccttccaacaccatctaatatctcagcactatgaaacttcggttccctcctaggattatgtttaattacccaaatcctcacgggactattcttagcaatgcactacacctccgacatctcaaccgcattttcatctgtgacccatatctgccgagatgttaactatggctgacttattcgaaatctgcatgccaatggggcatccttcttcttcatttgcctttacatgcatattgcacggggcctatattatggctcgtacctatacaaagagacctgaaacatcggtgtagtgctgtttctcctagttataatgacagccttcgtcggctacgtacttccatgaggacaaatgtccttttgaggtgccacagtaattacaaatctcctctcagcagtcccctacataggagatactcttgttcaatgaatctgaggcggtttctccgtagacaacgcaaccctcacccgattcttcgcgttccacttcctcctgccgtttgtcgtcgcgggcgcaaccatcctgcacctgttatttctacacgaaacaggatcaaacaacccagccgggctaaattctgacgccgacaagatttctttccatccgtacttctcatataaagaccttcttggctttgttatcatgttactagcccttacctctctaacattattttctcccaacctattaggtgacccggagaactttaccccagcaaacccactagtaacacccccacacattcagccagagtggtacttcttatttgcctacgccatccttcgatctattccaaataaactaggaggggtccttgcactattattcagcatcctagtgctaatggttgtgccaattttacatacctcaaaacaacgaggactaactttccgccccgtaactcaattcctattctgaaccctagttgcagatatattaatcctgacatgaatcgggggtatacctgtagaacacccatatattatcattggccaagtcgcatccattttatactttgcactattcctcattcttgtcccactggcggggtgaatagaaaataaagcattgaaatgagccHM560213Squaliuszrmanjae                ATGGCAAGCCTACGAAAAACTCACCCACTAATAAAAATCGCCAACGGCGCACTAGTTGACCTTCCAACACCATCTAATATCTCAGCACTATGAAACTTCGGTTCCCTCCTAGGATTATGTTTAATTACCCAAATCCTCACGGGACTATTCTTAGCAATGCACTACACCTCCGACATCTCAACCGCATTTTCATCTGTGACCCATATCTGCCGAGATGTTAACTATGGCTGACTTATTCGAAATCTGCATGCCAATGGGGCATCCTTCTTCTTCATTTGCCTTTACATGCATATTGCACGGGGCCTATATTATGGCTCGTACCTATACAAAGAGACCTGAAACATCGGTGTAGTGCTGTTTCTCCTAGTTATAATGACAGCCTTCGTCGGCTACGTACTTCCATGAGGACAAATGTCCTTTTGAGGTGCCACAGTAATTACAAATCTCCTCTCAGCAGTCCCCTACATAGGAGATACTCTTGTTCAATGAATCTGAGGCGGTTTCTCCGTAGACAACGCAACCCTCACCCGATTCTTCGCGTTCCACTTCCTCCTGCCGTTTGTCGTCGCGGGCGCAACCATCCTGCACCTGTTATTTCTACACGAAACAGGATCAAACAACCCAGCCGGGCTAAATTCTGACGCCGACAAGATTTCTTTCCATCCGTACTTCTCATATAAAGACCTTCTTGGCTTTGTTATCATGTTACTAGCCCTTACCTCTCTAACATTATTTTCTCCCAACCTATTAGGTGACCCGGAGAACTTTACCCCAGCAAACCCACTAGTAACACCCCCACACATTCAGCCAGAGTGGTACTTCTTATTTGCCTACGCCATCCTTCGATCTATTCCAAATAAACTAGGAGGGGTCCTTGCACTATTATTCAGCATCCTAGTGCTAATGGTTGTGCCAATTTTACATACCTCAAAACAACGAGGACTAACTTTCCGCCCCGTAACTCAATTCCTATTCTGAACCCTAGTTGCAGATATATTAATCCTGACATGAATCGGGGGTATACCTGTAGAACACCCATATATTATCATTGGCCAAGTCGCATCCATTTTATACTTTGCACTATTCCTCATTCTTGTCCCACTGGCGGGGTGAATAGAAAATAAAGCATTGAAATGAGCCAF090765Telestesalfiensis               atggcaagcctacgaaaaacccacccactaataaagatcgctaatgatgcactagtcgacctcccaacaccatctaatatctcagtgatatgaaacttcggctctcgcctagggttatgtttaattacccaaatcctaacgggattgttcttagccatacattatacctctgatatctcagccgcattctcgtcggtcacccacatttgccgagacgttaactacggctgacttatccggagcctacatgctaatggggcatcctttttcttcatctgtctttacatgcatatcgcacgaggcctatactacgggtcatacctttataaagagacctgaaacattggtgtagtactattcctcttggttatgatgacagccttcgttggctacgttctcccatgaggacaaatgtccttctgaggcgctaccgtgattacaaacctcctgtcagccgtcccttacatgggagacacccttgttcaatgaatctggggcggtttctcagtagacaatgcaactctcacacgattcttcgcattccacttcctcctaccattcgtcgtcaccggcgcaaccattctgcatttattgtttttacacgaaacagggtcaaacaacccggccgggttaaattccgacgcagacaaaatctctttccacccatacttctcgtacaaagaccttcttggctttgtggcaatattactagcccttacctccctaacattattctcccctaacctattaggcgacccagagaactttaccccagcaaatccactcgtgacacccccacatatccagccagagtgatacttcttgtttgcctacgccatcctccgctctatcccaaataaactaggaggggttcttgcgctactattcagcatcctggtgctattggtcgtgcccattttacatacatcaaagcagcgaggactaactttccgcccagcgactcaattcctattctgaacccttgttgcagatatatttattttgacatgaatcgggggcatacccgtagaacacccatatattattattggccaaatcgcatccgtcctatattttgcactcttcctcgtccttgtcccactagcagggtgggtggaaaataaagcattaaaatgagccAF090770Telestesbeoticus                atggcaagcctacgaaaaacccacccactaataaaaatcgctaatgacgcactagttgacctcccaacaccgtctaatatctccgtgatatgaaacttcggctctctcctaggattgtgtttaattacccaaatcctaacaggattattcttagccatacattatacctctgatatctcgaccgcattctcgtcagtaacccacatttgccgagacgtcaactacggctgacttatccgaagcctacatgctaatggggcatcctttttcttcatttgtctttatatgcatatcgcacgaggcctgtactatggctcgtatctttacaaagagacctgaaacattggcgtagtattattcctcttggtcatgatgacagccttcgtcggctacgttcttccatgaggacaaatgtccttttgaggtgctaccgtgattacaaacctcctctcagcagtcccttacataggagatacccttgtccaatgaatctgaggcggtttctcagtagacaatgcgactctcacacgattcttcgcattccacttcctcctcccgttcgtcgtcgccggcgcaacccttctgcatttattattcttacacgaaacaggatcaaacaacccagccggattaaattccgacgcggacaaaatttctttccacccatacttctcatataaagaccttcttggctttgtggcaatgttgctggcgcttacctctctaacattattctcccctaaccttctaggtgacccggaaaactttaccccagcaaacccgctcgtaacacccccgcatatccagccagagtgatacttcttatttgcctacgccattctccggtccatcccaaataagctaggaggggtccttgcactactatttagcatcctggtgttaatagtcgtgccaattttacacacgtcaaaacaacgaggactaactttccgcccgatgactcaattcctattctgaacccttgttgcagatatatttattttgacatgaatcgggggcatacccgtagaacacccatatattattattggccaaatcgcatccgtcctatattttgcactcttcctcgtccttgtcccactagcaggatgggtggaaaataaagcattgaaatgagccAY509828Telestescroaticus               atggcaagcctacgaaaaacccacccactgataaaaatcgctaatggcgcactagtcgacctcccaacaccatctaatatctcagcaatatgaaacttcggttctctcctaggactatgtttaattacccaaatcctaacgggattattcttagccatacattacacctctgacatctcaaccgcattctcatcggtaacccacatctgccgagacgtcaactacggctggcttatccgaagcttacatgctaacggagcatcctttttcttcatctgcctctatatgcatatcgcacgaggcctgtattatggatcatacctttataacgagacctgaaacattggtgtgggcctattcctcttagttatgatgacagccttcgttggctacgttctcccatgaggacaaatgtccttctgaggcgctaccgtaattacgaatctcctgtcagcggtcccttacataggggacacccttgtgcaatgaatctggggcggtttctccgtagacaatgcaactctcacacgattattcgcattccacttcctcctgccattcgtcgtcaccggtgcaaccattctacacttattattcttacacgagacagggtcgaacaaccccgccgggctaaattccgacgcagacaaaatttctttccacccatacttctcatataaagacctccttggcttcgtggcaatgctattagcgctcgcctctctgacactattttcccccaacttactaggtgacccggaaaactttaccccagcaaacccgctcgtaacacctccacatatccagccagaatgatacttcttatttgcctacgccatcctccgatccatcccaaataaactgggaggggttcttgcactactattcagcatcctcgtgctaatagttgtgcccattctacacacatcaaaacaacgaggactaactttccgcccagtgactcaattcctattctgagccctcgttgcagatatatttattttgacatgaatcggaggcatacccgtagaacacccgtttattattatcggccaaatcgcatccgtcctatactttgcactcttcctcgtccttgtcccaccagcgggatgggtggagaataaagcattgaaatgagccHM560214Telestescroaticus               ATGGCAAGCCTACGAAAAACCCACCCACTGATAAAAATCGCTAATGGCGCACTAGTCGACCTCCCAACACCATCTAATATCTCAGCAATATGAAACTTCGGCTCTCTCCTAGGACTATGTTTAATTACCCAAATCCTAACGGGATTATTCTTAGCCATACATTACACCTCTGACATCTCAACCGCATTCTCATCGGTAACCCACATCTGCCGAGACGTCAACTACGGCTGGCTCATCCGAAGCTTACATGCTAACGGAGCATCCTTTTTCTTCATCTGCCTCTATATGCATATCGCACGAGGCCTGTATTATGGATCATACCTTTATAAAGAGACCTGAAACATTGGCGTGGTCCTATTCCTTCTAGTTATAATAACAGCCTTCGTCGGCTACGTTCTTCCATGAGGACAAATATCCTTTTGAGGTGCCACCGTGATTACAAACCTCCTCTCAGCAGTCCCTTACATGGGAGACACCCTTGTTCAGTGAATCTGAGGCGGTTTCTCAGTAGACAATGCAACTCTCACACGATTCTTCGCATTCCACTTCCTCCTGCCGTTCGTTGTCGCTGGTGCAACCATTCTACACTTATTATTCTTACACGAGACAGGGTCGAACAACCCCGCCGGGCTAAATTCCGACGCAGACAAAATTTCTTTCCACCCATACTTCTCATATAAAGACCTCCTTGGCTTCGTGGCAATGCTATTAGCGCTCGCCTCTCTGACACTATTTTCCCCCAACTTACTAGGTGACCCGGAAAACTTTACCCCAGCAAACCCGCTCGTAACACCTCCACATATCCAGCCAGAATGATACTTCTTATTTGCCTACGCCATCCTCCGATCCATCCCAAATAAACTGGGAGGGGTTCTTGCACTACTATTCAGCATCCTCGTGCTAATAGTTGTGCCCATTCTACACACATCAAAACAACGAGGACTAACTTTCCGCCCAGTGACTCAATTCCTATTCTGAGCCCTCGTTGCAGATATATTTATTTTGACATGAATTGGAGGCATACCCGTAGAGCACCCGTATATTATTATTGGCCAAGTCGCATCCATCTTATACTTTGCACTCTTCCTCGTCCTTGTTCCGCTAGCAGGATGACTGGAAAATAAAGCATTAAAATGAGCCAY838932Telestescroaticus               atggcaagcctacgaaaaacccacccactgataaaaatcgctaatggcgcactagtcgacctcccaacaccatctaatatctcagcaatatgaaacttcggctctctcctaggactatgtttaattacccagatcctaacgggattattcttagccatacattacacctctgacatctcaaccgcattctcatcggtaacccacatctgccgagacgtcaactacggctggctcatccgaagcttacatgctaacggagcatcctttttcttcatctgcctctatatgcatatcgcgcgaggcctgtattatggatcatacctttataaagagacctgaaacattggcgtggtcctattccttctagttataataacggccttcgtcggctacgttcttccatgaggacaaatatccttttgaggtgccaccgtaattacaaatctcctctcagcagtcccttacataggagacacccttgttcaatgaatctggggcggtttctcagtagacaatgcaactctcacacgattcttcgcattccacttcctcctgccattcgttgtcgctggtgcaaccattctacacttattattcttncacgaaacagggtcgaacaaccccgccgggctaaattccgacgcagacaaaatctctttccacccatacttctcatataaagacctccttggcttcgtggcaatgctattagcgctcgcctctctcacgctgttttcccccaacttactaggtgacccggaaaactttaccccagcaaacccgctcgtaacacctccacatatccagccagaatgatacttcttatttgcctacgccatcctccgatccatcccaaataaactgggaggggttcttgcactactattcagcatcctcgtgctaatagttgtgccgattctacacacgtcaaaacaacgaggactaactttccgcccagtgactcaattcctattctgagccctcgttgcagatatatttattttgacatgaattggaggcatacccgtagaacacccatatattattattggccaagtcgcatccatcttatattttgcactcttcctcgtccttgttccgctagcaggatgactggaaaataaagcattaaaatgagccAY838928Telestesfontinalis              atggcaagcctacgaaaaacccacccactgataaaaatcgctaatggcgcactagtcgacctcccaacaccatctaatatctcagcaatatgaaacttcggctccctcctaggactatgtttaattacccagatcctaacaggattattcttagccatacattacacctctgacatctcaaccgcattctcatcggtaacccacatctgccgagacgtcaactacggctggctcatccgaagcttacatgctaacggagcatcctttttcttcatctgcctctatatgcatatcgcacgaggcctgtattatggatcatacctttataaagagacctgaaacattggcgtggtcctattccttctggttataataacggccttcgtcggctacgttcttccatgaggacaaatatccttttgaggtgctaccgtaattacaaacctcctctcagcagtcccttacatgggagacacccttgttcagtgaatctgaggcggtttctcagtagacaatgcaactctcacacgattcttcgcattccacttccttctgccattcattgtcgctggtgcaaccattctacacttattattcttacacgaaacagggtcgaacaaccccgccggactaaattccgacgcagacaaaatttctttccacccatacttctcatacaaagacctccttggcttcgtagcaatgttattagcgctcgcctctctaacactattttcccccaacttactaggtgacccggaaaactttaccccagcaaacccgctcgtaacacccccacacatccagccagaatgatacttcttatttgcctacgccatcctccgatccatcccaaataaactgggaggggttcttgcactactattcagcatcctcgtgctaatagttgtgccgattctacacacatcaaaacaacgaggactaaccttccgcccagtgactcaattcctattctgagccctcgttgcagatatatttattttgacatgaattgggggcatacccgtagaacacccatatattattattggccaagtcgcatccatcctatactttgcactcttcctcgtccttgttccgctagcaggatgactggaaaataaagcattaaaatgagccHM560215Telestesfontinalis              ATGGCAAGCCTACGAAAAACCCACCCACTGATAAAAATCGCTAATGGCGCACTAGTCGACCTCCCAACACCATCTAATATCTCAGCAATATGAAACTTCGGCTCCCTCCTAGGACTATGTTTAATTACCCAGATCCTAACAGGATTATTCTTAGCCATACATTACACCTCTGACATCTCAACCGCATTCTCATCGGTAACCCACATCTGCCGAGACGTCNACTACGGCTGGCTCATCCGAAGCTTACATGCTAACGGAGCATCCTTTTTCTTCATCTGCCTCTATATGCATATCGCACGAGGCCTGTATTATGGATCATACCTTTATAAAGAGACCTGAAACATTGGCGTGGTCCTATTCCTTCTGGTTATAATAACGGCCTTCGTCGGCTACGTTCTTCCATGAGGACAAATATCCTTTTGAGGTGCTACCGTAATTACAAACCTCCTCTCAGCAGTCCCTTACATGGGAGACACCCTTGTTCAGTGAATCTGAGGCGGTTTCTCAGTAGACAATGCAACTCTCACACGATTCTTCGCATTCCACTTCCTTCTGCCATTCGTTGTCGCTGGTGCAACCATTCTACACTTATTATTCTTACACGAAACAGGGTCGAACAACCCCGCCGGACTAAATTCCGACGCAGACAAAATTTCTTTCCACCCATACTTCTCATACAAAGACCTCCTTGGCTTCGTGGCAATGTTATTAGCGCTCGCCTCTCTAACACTATTTTCCCCCAACTTACTAGGTGACCCGGAAAACTTTACCCCAGCAAACCCGCTCGTAACACCCCCACACATCCAGCCAGAATGATACTTCTTATTTGCCTACGCCATCCTCCGATCCATCCCAAATAAACTGGGAGGGGTTCTTGCACTACTATTCAGCATCCTCGTGCTAATAGTTGTGCCGATTCTACACACATCAAAACAACGAGGACTAACCTTCCGCCCAGTGACTCAATTCCTATTCTGAGCCCTCGTTGCAGATATATTTATTTTGACATGAATTGGGGGCATACCCGTAGAACACCCATATATTATTATTGGCCAAGTCGCATCCATCCTATACTTTGCACTCTTCCTCGTCCTTGTTCCGCTAGCAGGATGACTGGAAAATAAAGCATTAAAATGAGCCHM560216Telestesmetohiensis             ATGGCAAGCCTACGAAAAACCCACCCACTAATAAAAATCGCTAATGACGCACTAGTCGACCTCCCAACACCATCTAACATCTCAGTAATGTGAAACTTCGGCTCTCTCCTGGGATTATGTTTAATTACCCAAATCCTGACAGGACTGTTTTTAGCCATACATTATACCTCTGATATCTCGACCGCATTCTCATCAGTAACCCACATCTGCCGGGACGTCAACTACGGCTGACTTATCCGAAGCCTACACGCCAACGGCGCATCCTTTTTCTTCATCTGCCTTTACATACATATCGCACGAGGTCTGTATTATGGGTCATACCTTTATAAAGAGACCTGAAATATTGGCGTAGTCCTATTTCTTTTAGTCATGATAACGGCCTTCGTCGGCTACGTTCTTCCATGAGGACAAATGTCCTTTTGAGGCGCCACCGTGATTACAAACCTCCTCTCGGCGGTCCCTTACATAGGGGATACCCTTGTGCAGTGAATCTGAGGCGGTTTCTCAGTAGATAATGCAACCCTCACACGATTCTTCGCATTCCATTTCCTCCTGCCATTTGTCGTCGCCGGCGCAACCATCCTACACTTATTATCCTTGCACGAAACGGGGTCAAACAACCCAGCCGGACTAAATTCCGACGCGGACAAAATTTCTTTCCACCCTTACTTCTCCTATAAAGACCTTCTTGGTTTCGTGGCGATATTACTAGCCCTTACCTCCCTAATATTGTTTTCCCCTAACCTACTTGGTGACCCGGAAAACTTtACCCCAGCAAACCCACTTGGGACACCTCcACATATCCAGCCAGAATGGTACTTCTTATTTGCCTACGCCATCCTCCGATCTATtCCAAATAAGCTAGGGGGGGTTCTTGCACTACTCTTCAGCATCCTGGtGCTAATAGtCGTGCCGATTCTACACACATCGAaACGACGAGGACTAACTTTCCGGCCGGTGACTCAATTCCTATTCTGAACCCTTGTGGCAGATATATTTATTTTGACGTGAATCGGCGGCATACCCGTAGAGCACCCATATATTATTATCGGCCAAATCGCATCCGTCCTATATTTTGCACTCTTCCTGATTCTTGTCCCACTGACAGGATTGGTGGAAAATAAGGCATTAAAATGAGCCHM560217Telestesmetohiensis             ATGGCAAGCCTACGAAAAACCCACCCACTAATAAAAATCGCTAATGATGCACTAGTCGACCTCCCAACACCATCTAACATCTCAGTAATGTGAAACTTCGGCTCTCTCCTGGGATTATGTTTAATTACCCAAATCCTGACAGGACTGTTTTTAGCCATACATTATACCTCTGATATCTCGACCGCATTCTCATCAGTAACCCACATCTGCCGGGACGTCAACTACGGCTGACTCATCCGAAGCCTACACGCTAACGGCGCATCCTTTTTCTTCATCTGCCTTTACATACATATCGCACGGGGTCTGTATTATGGGTCATACCTTTATAAAGAGACCTGAAATATTGGCGTAGTGCTATTTCTTTTAGTCATGATAACGGCCTTCGTCGGCTACGTTCTTCCATGAGGACAAATATCCTTTTGAGGCGCCACCGTGATTACAAACCTCCTCTCAGCGGTCCCTTACATAGGGGATACCCTTGTGCAATGAACCTGAGGCGGTTTTTCAGTAGATAATGCAACCCTCACACGATTCTTCGCATTCCATTTCCTCCTGCCATTTGTCGTCGCCGGCGCAACCATCCTACACTTATTATTCTTGCACGAAACGGGGTCAAACAACCCAGCCGGACTAAATTCCGACGCGGACAAAATTTCTTTCCACCCTTACTTCTCCTATAAAGACCTTCTTGGTTTCGTGGCGATATCACTAGCCCTTACCTCCCTAACATTGTTTTCCCCTAACCTACTTGGTGACCCGGAAAACTTTACCCCAGCAAACCCACTTGTGACACCTCCACATATCCAGCCAGAATGGTACTTCTTATTTGCCTACGCCATCCTCCGATCTATTCCAAATAAGCTAGGAGGGGTTCTTGCACTACTCTTCAGCATCCTAGTGCTAATAGTCGTACCGATTCTACACACATCGAAACAACGAGGACTAACTTTCCGGCCGGTGACTCAATTCCTATTCTGAACCCTTGTGGCAGATATATTTATTTTGACGTGAATCGGCGGCATACCCGTAGAACACCCATATATTATTATCGGCCAAATCGCATCCGTCCTGTATTTTGCACTCTTCCTGATTCTTGTCCCACTGGCAGGATTGGTGGAAAATAAGGCATTAAAATGAGCCAY509849Telestesmontenegrinus           atggcaagcctacgaaaaacccacccactaataaaaatcgctaatgacgcactagtcgacctaccaacaccatctaatatctcagcaatatgaaacttcggttctctgctcggattatgtctaattacccaaatcctaacaggattattcttagccatacattatacctccgacatctcaaccgcattctcgtcagtaactcacatctgccgagacgtcaattacggctgacttatccgaagcctacacgccaacggagcatcctttttcttcatctgcctttacatacatatcgcacgaggcctgtactatggatcatacctttataaggagacctgaaacattggcgtagtcctattccttctagttatgatgacagccttcgttggctacgttcttccatgaggacaaatatccttctggggtgctaccgtaattacgaatctcctttcaacgggcccttacatgggggatacccttgttcaatgaatctggggcggtttttcagtagacaatgcgactcttacacgattctttggattccacttcctcctgccattcgtcgtcaccggcgcaaccgttctacatttattgtttttacacgaaacaggctcaaacaacccagccggactaaactccgacgcagataaaatttctttccatccctacttctcatacaaagaccttcttggctttgtggcaatgttactagcacttacctctctaacattattctcccctaacctactaggtgacccggaaaactttacgccagcaaacccgctcgtaacgcctccacatatccagccagaatgatacttcttgtttgcctacgccatcctccgatctatccctaataaactatgaggggtccttgcactactgttcagcatccttgtattaatagtcgtgccaattttacacacgtcaaaacaacgaggactaactttccgccccgtgactcaattcctattctgaacccttgttgcagatatatttattttgacgtgaatcggaggcatacccgtagagcacccatatattgttatcggccaaatcgcatccgtcctatactttgcactcttccttattcttgtaccactagcaggttgggtggaaaataaagcattaaaatgagccAY509856Telestesmuticellus              atggctagcctacgaaaaacccacccactaataaaaatcgctaatgacgctctagtcgacctcccaacaccgtctaatatctcagcaatatgaaacttcggttctctcctaggattatgtttaattacccaaatcctgacaggattattcctagccatacattacacctctgacatctcaaccgcgttctcatcagtaactcacatctgccgagacgtcaattacggctgacttatccgaaacctgcatgccaatggcgcatcctttttcttcatctgcctttacatgcacattgcacgaggcctgtactatggatcatacctctataaggagacctgaaacattggcgtagtcctattcctcctagttatgataacagccttcgtcggctacgttcttccatggggacaaatatccttttgaggggcaaccgtaattacaaatctcctctcagcggtcccttatataggagatacgcttgttcaatgaatctgagggggtttctcagtagacaatgcaactctcacacggttcttcgcattccacttcctcctgccatttgtcgtcaccggcgcaaccattctacacttattatttttacacgaaacagggtcaaataacccggccggactaaattctgacgcggataaaatttctttccacccatacttctcatacaaagaccttcttggctttgtggcaatattattagcccttacctctctaacattattctcccccaacctactaggtgacccggaaaactttaccccggcaaaccccctcgtaacacccccacatatccagccagaatgatacttcttatttgcctacgccatcctccgatctattccaaataaactaggaggggttcttgcactactattcagcatcctcgtactaatagtcgtgccgattctacacacgtcgaaacaacgaggactaactttccgcccagtgactcaattcctattctgaaccctcgttgcagatatatttattttgacgtgaatcgggggcatacccgtagaacacccatatattattatgggccaaatcgcttccgtcctatattttgcactcttcctattccttgtcccactaccaggttgggtggaaaataaagcattaaaatgagccHM560218Telestesmuticellus              ATGGCAAGCCTACGAAAAACCCACCCACTGATAAAAATCGCTAATGACGCACTAGTCGACCTCCCAACACCATCTAATATCTCAGCAATATGAAACTTCGGTTCTCTCCTAGGATTATGTTTAATTACCCAAATCCTGACAGGATTATTCCTAGCCATACATTACACCTCTGACATCTCAACCGCGTTCTCATCAGTAACCCACATCTGCCGAGACGTCAATTACGGCTGACTTATCCGAAGCCTACATGCCAACGGCGCATCCTTTTTCTTCATCTGCCTTTACATGCATATTGCACGAGGCCTGTACTATGGATCATACCTTTATAAGGAGACCTGAAACATTGGTGTAGTCCTACTCCTTCTGGTTATGATAACAGCCTTCGTCGGCTACGTTCTTCCATGGGGACAGATATCCTTTTGAGGTGCAACCGTAATTACAAATCTGCTCTCAGCGGTCCCTTATATAGGAGATACGCTTGTTCAATGAATCTGAGGCGGTTTCTCAGTAGACAGTGCAACTCTCACACGATTCTTCGCATTCCACTTCCTTCTACCATTTGTCATCGCCGGCGCAACCATTCTGCACTTATTATTTTTACACGAAACGGGATCAAATAACCCGGCCGGACTAAATTCCGACGCAGATAAGATTTCTTTCCACCCATACTTCTCATACAAAGACCTTCTTGGCTTTGTGGCAATATTATTAGCCCTTACCTCGCTAACATTATTCTCCCCCAACCTACTCGGTGACCCGGAAAACTTTACCCCGGCGAACCCCCTCGTAACGCCGCCACATATCCAGCCAGAATGATACTTCTTATTTGCCTACGCCATCCTCCGATCTATTCCAAATAAACTAGGAGGTGTTCTTGCACTACTGTTCAGCATCCTGGTACTAATAGTCGTGCCCATTTTACACACATCGAAACAACGAGGACTAACTTTCCGCCCAGCGACTCAATTCCTATTCTGGACCCTCGTTGCGGATATATTTATTTTGACGTGAATCGGAGGCATACCCGTAGAGCACCCGTATATTATTATCGGCCAAATCGCATCCGTCCTATACTTTGCACTCTTCCTTATTCTTGTCCCCCTAGCAGGATGAGTGGAGAACAAAGCATTGAAATGAGCCHM560218Telestesmuticellus              ATGGCAAGCCTACGAAAAACCCACCCACTGATAAAAATCGCTAATGACGCACTAGTCGACCTCCCAACACCATCTAATATCTCAGCAATATGAAACTTCGGTTCTCTCCTAGGATTATGTTTAATTACCCAAATCCTGACAGGATTATTCCTAGCCATACATTACACCTCTGACATCTCAACCGCGTTCTCATCAGTAACCCACATCTGCCGAGACGTCAATTACGGCTGACTTATCCGAAGCCTACATGCCAACGGCGCATCCTTTTTCTTCATCTGCCTTTACATGCATATTGCACGAGGCCTGTACTATGGCTCATACCTTTATAAGGAGACCTGAAACATTGGTGTAGTCCTATTCCTTCTGGTTATGATAACAGCCTTCGTCGGCTACGTTCTTCCATGGGGACAGATATCCTTTTGAGGTGCAACCGTAATTACAAATCTGCTCTCAGCGGTCCCTTATATAGGAGATACGCTTGTTCAATGAATCTGAGGCGGTTTCTCAGTAGACAATGCAACTCTCACACGATTCTTCGCATTCCACTTCCTTCTACCATTTGTCATCGCCGGCGCAACCATTCTGCACTTATTATTTTTACACGAAACGGGATCAAATAACCCGGCCGGACTAAATTCCGACGCAGATAAGATTTCTTTCCACCCATACTTCTCATACAAAGACCTTCTTGGCTTTGTGGCAATATTATTAGCCCTTACCTCGCTAACATTATTCTCCCCCAACCTACTCGGTGACCCGGAAAACTTTACCCCGGCGAACCCCCTCGTAACGCCCCCACATATCCAGCCAGAATGATACTTCTTATTTGCCTACGCCATCCTCCGATCTATTCCAAATAAACTAGGAGGTGTTCTTGCACTACTGTTCAGCATCCTGGTACTAATAGTCGTGCCCATTTTACACACATCGAAACAACGAGGACTAACTTTCCGCCCAGCGACTCAATTCCTATTCTGGACCCTCGTTGCGGATATATTTATTTTGACGTGAATCGGGGGCATACCCGTAGAGCACCCGTATATTATTATCGGCCAAATCGCATCCGTCCTATACTTTGCACTCTTCCTTATTCTTGTCCCACTAGCAGGATGAGTGGAGAACAAAGCATTGAAATGAGCCAF090764Telestespleurobipunctatus       atggcaagcctacgaaaaacccacccactaataaagatcgctaacgacgcgctagtcgacctcccgacaccatctaatatctcagtaatatgaaacttcggttccctcctgggattatgtttaattacccaaatcctaacggggttattcttagccatacattatacctctgatatctcaaccgcattctcgtcagtcacccacatttgccgagacgtcaactacggctgacttatccggagcctacatgctaatggggcatcctttttcttcatctgtctttacatgcatatcgcacgaggactgtactacgggtcatacctttataaggagacctgaaacattggtgtggtattattcctcttagttatgatgacagccttcgttggctacgttctcccatgaggacaaatgtccttctgaggcgctaccgtaattacgaatctcctgtcagcggtcccttacataggggacacccttgtgcaatgaatctggggcggtttctccgtagacaatgcaactctcacacgattattcgcattccacttcctcctgccattcgtcgtcaccggcgcaaccattctgcatttattgtttttacacgaaacaggctcaaacaacccagccggattaaattccgacgcagacaaaatctctttccacccatacttctcatacaaagaccttcttggctttgtggcaatattgctagcccttacctccctaacattattctcccctaacctattgggtgacccggagaactttaccccagcaaacccgctcgtaacgcccccacatatccagccagagtgatacttcctgtttgcctacgccatcctccgctctatcccaaataaactaggaggggtccttgcactactattcagcatcctggtgctattggtcgtgcccattttacacacatcgaagcagcgaggactaactttccgcccagtgactcaattcctattctgaacccttgttgcagatatatttattttgacatgaatcgggggcatacccgtagaacacccatatattgttattggccaaatcgcatccgtcctatattttgcactcttcctagtccttgtcccactagcaggatgggtggaaaataaagcattaaaatgagccHM560220Telestespleurobipunctatus       ATGGCAAGCCTACGAAAAACCCACCCACTAATAAAGATCGCTAATGACGCGCTGGTCGACCTCCCAACACCATCTAATATCTCAGTAATATGAAACTTCGGTTCCCTCCTGGGATTATGTTTAATTACCCAAATCCTAACGGGATTATTCTTAGCCATACATTATACCTCTGATATCTCAACCGCATTCTCGTCAGTTACCCACATTTGCCGAGACGTCAACTACGGCTGACTTATCCGGAGCCTACATGCTAATGGGGCATCCTTTTTCTTCATCTGTCTTTACATGCATATCGCACGAGGCCTGTACTACGGGTCATACCTTTATAAGGAGACCTGAAACATTGGTGTGGTATTATTCCTCTTAGTTATGATGACAGCCTTCGTTGGCTACGTTCTCCCATGAGGACAAATATCCTTCTGAGGCGCTACCGTAATTACGAATCTCCTGTCAGCGGTCCCTTACATGGGGGACACCCTTGTTCAATGAATCTGGGGCGGTTTCTCCGTAGACAATGCAACTCTCACACGATTCTTCACATTCCACTTCCTCCTGCCATTCGTCGTCGCCGGCGCAACCATTCTGCATTTATTGTTTTTACACGAAACAGGGTCAAACAACCCGGCCGGGTTAAATTCCGACGCAGACAAAATCTCTTTCCACCCATACTTCTCATACAAAGACCTTCTTGGCTTCGTGGCAATGTTGCTAGCCCTCACCTCCCTAACATTATTCTCCCCTAACCTATTGGGTGACCCGGAGAACTTTACCCCAGCAAACCCGCTCGTGACGCCCCCACATATCCAGCCAGAGTGATACTTCCTGTTTGCCTACGCCATCCTCCGCTCTATCCCAAATAAACTAGGAGGGGTTCTTGCACTACTATTCAGCATCCTGGTGCTATTGGTCGTGCTGATTTTACACACATCGAAGCAGCGAGGACTAACTTTCCGCCCAGTGACTCAATTCCTATTCTGAACCCTTGTTGCAGATATATTTATTTTGACATGAATCGGGGGCATACCCGTAGAACACCCATATATTATTATTGGCCAAATCGCATCCGTCCTATATTTTGCACTCTTCCTCGTCCTTGCACCACTAGCGGGATGGGTGGAAAATAAAGCATTAAAATGAGCCHM560221Telestespolylepis               ATGGCAAGCCTACGAAAAACCCACCCGCTAATAAAAATCGCTAATGACGCACTAGTCGACCTCCCAACACCATCTAATATCTCAGCAATGTGGAACTTCGGTTCCCTCCTGGGATTATGTTTAATTACCCAAATCCTAACAGGaTTATTCTTAGCCATACATTATACCTCTGACATCTCAACCGCATTCTCATCAGTAACTCACATCTGCCGAGACGTCaATTACGGCTGACTTATCCGAAGCCTACATGCTAACGGAGCATCATTTTTCTTCATCTGCCTTTACATACATATCGCACGAGGCCTGTACTATGGGTCCTACCTTTATAAGGAGACCTGAAACATTGGTGTGGTCCTATTCCtTCTAGTTATAATGACGGCCTTCGTCGGCTACGTTCTTCCATGAGGACAAATATCCTTTTGAGGTGCTACCGTAATTACAAATCTCCTCTCAGCGGTCCCTTACATAGGAGATACCCTTGTTCAATGGATCTGAGGAGGTTTCTCAGTAGACAATGCAACCCTCACACGGTTCTTCGCATTCCACTTCCTCCTACCATTTGTCGTTGCCGGCGCAACCATTCTACACTTATTATTCTTACACGAAACCGGATCAAACAACCCGGCCGGACTAAATTCCGACGCAGATAAGATTTCTTTCCACCCGTACTTCTCATATAAAGACCTTCTTGGCTTTGTGGCAATGTTACTAGCCCTAACCTCTCTGACATTATTCTCCCCCAACCTTCTAGGTGACCCGGAAAACTTTACCCCGGCAAACCCGCTCGTAACACCTCCGCATATCCAGCCAGAATGATACTTCTTATTTGCCTACGCCATCCTCCGATCTATTCCAAATAAACTAGGAGGGGTTCTTGCACTACTATTCAGCATCCTGGTACTAATAGTCGTGCCAATTTTACACACATCTAAACAACGAGGACTAACTTTCCGCCCGGTGACTCAATTCCTATTCTGAACCCTTGTTGCAGATATATTTATTTTGACATGAATCGGGGGCATACCCGTAGAACACCCATATATTATTATCGGCCAAATCGCATCCGTCTTATACTTTGCACTCTTCCTCATTCTTGTCCCACTAGCAGGATGAGTGGAAAATAAAGCATTGAAATGAGCCHM560222Telestespolylepis               ATGGCAAGCCTACGAAAAACCCACCCGCTAATAAAAATCGCTAATGACGCACTAGTCGACCTCCCAACACCATCTAATATCTCAGCAATGTGGAACTTCGGTTCCCTCCTGGGATTATGTTTAATTACCCAAATCCTAACAGGATTATTCTTAGCCATACATTATACCTCTGACATCTCAACCGCATTCTCATCAGTAACTCACATCTGCCGAGACGTCAATTACGGCTGACTTATCCGAAGCCTACATGCTAACGGAGCATCATTTTTCTTCATCTGCCTTTACATACATATCGCACGAGGCCTGTACTATGGGTCCTACCTTTATAAGGAGACCTGAAACATTGGTGTGGTCCTATTCCTTCTAGTTATAATGACGGCCTTCGTCGGCTACGTTCTTCCATGAGGACAAATATCCTTTTGAGGTGCTACCGTAATTACAAATCTCCTCTCAGCGGTCCCTTACATAGGAGATACCCTTGTTCAATGGATCTGAGGAGGTTTCTCAGTAGACAATGCAACCCTCACACGGTTCTTCGCATTCCACTTCCTCCTACCATTTGTCGTTGCCGGCGCAACCATTCTACACTTATTATTCTTACACGAAACCGGATCAAACAACCCGGCCGGACTAAATTCCGACGCAGATAAGATTTCTTTCCACCCGTACTTCTCATATAAAGACCTTCTTGGCTTTGTGGCAATGTTACTAGCCCTAACCTCTCTGACATTATTCTCCCCCAACCTTCTAGGTGACCCGGAAAACTTTACCCCGGCAAACCCGCTCGTAACACCTCCGCATATCCAGCCAGAATGATACTTCTTATTTGCCTACGCCATCCTCCGATCTATTCCAAATAAACTAGGAGGGGTTCTTGCACTACTATTCAGCATCCTGGTACTAATAGTCGTGCCAATTTTACACACATCTAAACAACGAGGACTAACTTTCCGCCCGGTGACTCAATTCCTATTCTGAACCCTTGTTGCAGATATATTTATTTTGACATGAATCGGGGGCATACCCGTAGAACACCCATATATTATTATCGGCCAAATCGCATCCGTCTTATACTTTGCACTCTTCCTCATTCTTGTCCCACTAGCAGGATGAGTGGAAAATAAAGCATTGAAATGAGCCHM560223Telestessouffia                 ATGGCAAGCCTACGAAAAACCCACCCACTAATAAAAATCGCTAATGACGCACTAGTCGACCTCCCAACACCATCTAATATTTCAGTAATATGAAACTTCGGTTCTCTTCTAGGATTATGTTTAATTACCCAAATCCTAACAGGATTATTCTTAGCCATGCACTATACCTCTGACATCTCAACCGCATTCTCATCCGTGACCCACATCTGCCGAGACGTCAACTACGGCTGACTTATCCGGAGCCTACATGCTAACGGGGCATCCTTCTTCTTCATCTGCCTTTACATGCACATCGCACGAGGCCTGTATTATGGGTCATACCTTTATAAAGAGACCTGAAGCATTGGTGTGGTTCTATTCCTTCTGGTTATGATGACAGCCTTCGTCGGCTACGTTCTTCCATGAGGACAAATATCCTTTTGAGGTGCTACCGTAATTACGAACCTCCTCTCAGCAGTCCCTTACATGGGAGATACCCTTGTTCAGTGAATCTGAGGCGGTTTCTCCGTAGACAATGCAACTCTTACACGGTTCTTCGCATTCCACTTCCTCCTACCATTTGTCATCGCCGGCGCAACCATCCTGCACCTATTATTCTTACACGAAACGGGATCGAACAACCCAGCCGGACTAAATTCCGACGCGGACAAAATTTCTTTCCACCCATACTTCTCGTATAAGGACCTTCTTGGCTTTGTGGCAATGTTACTAGCCCTCACCTCTCTAACCTTATTTTCCCCTAACCTCTTAGGTGACCCGGAGAACTTCACCCCAGCAAACCCACTCGTAACGCCTCCACATATCCAGCCAGAGTGGTACTTCTTATTTGCCTACGCCATCCTCCGATCTATCCCAAATAAGCTGGGAGGGGTTCTTGCACTACTGTTCAGCATCCTGGTGCTAATAGTCGTGCCAATTTTACACACGTCCAAACAACGAGGACTAACTTTCCGCCCAGTGACTCAATTCCTATTCTGAACCCTTGTTGCGGATATATTTATTTTGACGTGAATCGGAGGCATACCCGTAGAACACCCATATATTATTATCGGCCAAATCGCATCCGTCCTATACTTTGCACTCTTCCTCGTTCTTGTCCCACTAGCGGGATGGGTGGAGAATAAAGCATTGAAATGAGCCHM530224Telestessouffia                 ATGGCAAGCCTACGAAAAACCCACCCACTAATAAAAATCGCTAATGACGCACTAGTCGACCTCCCAACACCATCTAATATTTCAGTAATATGAAACTTCGGTTCTCTTCTAGGATTATGTTTAATTACCCAAATCCTAACAGGATTATTCTTAGCCATGCACTATACCTCTGACATCTCAACCGCATTCTCATCCGTAACCCACATCTGCCGAGACGTCAACTACGGCTGACTTATCCGGAGCCTACATGCTAACGGGGCATCCTTCTTCTTCATCTGCCTTTACATGCACATCGCACGAGGCCTGTATTATGGGTCATACCTTTATAAAGAGACCTGAAGCATTGGTGTGGTTCTATTCCTTCTGGTTATGATGACAGCCTTCGTCGGCTACGTTCTTCCATGAGGACAAATATCCTTTTGAGGTGCTACCGTAATTACGAACCTCCTCTCAGCAGTCCCTTACATGGGAGATACCCTTGTTCAGTGAATCTGAGGCGGTTTCTCCGTAGACAATGCAACTCTTACACGGTTCTTCGCATTCCACTTCCTCCTACCATTTGTCATCGCCGGCGCAACCATCCTGCACCTATTATTCTTACACGAAACGGGATCGAACAACCCAGCCGGACTAAATTCCGACGCGGACAAAATTTCTTTCCACCCATACTTCTCGTATAAGGACCTTCTTGGCTTTGTGGCAATGTTACTAGCCCTCACCTCTCTAACCTTATTTTCCCCTAACCTCTTAGGTGACCCGGAGAACTTCACCCCAGCAAACCCACTCGTAACGCCTCCACATATCCAGCCAGAGTGGTACTTCTTATTTGCCTACGCCATCCTCCGATCTATCCCAAATAAGCTGGGAGGGGTTCTTGCACTACTGTTCAGCATCCTGGTGCTAATAGTCGTGCCAATTTTACACACGTCCAAACAACGAGGACTAACTTTCCGCCCAGTGACTCAATTCCTATTCTGAACCCTTGTTGCGGATATATTTATTTTGACGTGAATCGGAGGCATACCCGTAGAACACCCATATATTATTATCGGCCAAATCGCATCCGTCCTATACTTTGCACTCTTCCTCGTTCTTGTCCCACTAGCGGGATGGGTGGAGAATAAAGCATTGAAATGAGCCHM560225Telestessp                      ATGGCAAGCCTACGGAAAACCCACCCGCTAATAAAAATCGCTAATGACGCGCTAGTCGACCTCCCAACACCATCTAATATCTCAGCAATGTGGAACTTCGGTTCGCTCCTGGGATTATGTTTAATTACCCAGATCCTAACAGGATTATTCTTAGCCATACATTATACCTCTGACATCTCAACCGCATTCTCATCAGTAACTCACATCTGCCGAGACGTCAATTACGGCTGACTTATCCGAAGCCTGCATGCTAACGGAGCATCATTTTTCTTCATCTGCCTTTACATACATATCGCACGAGGACTGTACTATGGATCCTACCTTTATAAGGAGACCTGAAACATCGGTGTAGTACTGTTCCTTCTAGTTATGATGACGGCCTTCGTCGGCTACGTTCTTCCATGAGGACAAATATCCTTTTGAGGTGCTACCGTAATTACAAATCTCCTCTCAGCGGTCCCTTACATAGGGGATACCCTTGTTCAATGAATCTGAGGCGGTTTCTCAGTAGACAATGCAACTCTCACACGGTTCTTCGCGTTCCACTTCCTCCTACCATTTGTCGTTGCCGGCGCAACCATTCTACACTTATTGTTCTTACACGAAACCGGATCAAACAACCCGGCCGGACTAAATTCCGACGCAGATAAAATTTCTTTCCACCCGTACTTCTCATATAAAGACCTTCTTGGCTTTGTGGCAATGTTACTAGCCCTAACCTCTCTAACATTGTTCTCCCCCAACCTTCTGGGTGACCCGGAAAACTTTACCCCAGCAAACCCCCTTGTAACGCCTCCTCATATCCAGCCAGAGTGATACTTTTTGTTTGCCTACGCCATCCTCCGATCTATCCCAAATAAACTAGGAGGGGTTCTTGCACTACTATTCAGCATCCTGGTACTAATAGTTGTGCCGATTTTACACACATCTAAACAACGAGGACTAACTTTCCGGCCAGTGACTCAATTCCTATTCTGAACCCTTGTTGCAGATATATTTATTTTGACATGAATCGGGGGCATACCCGTAGAACACCCATATATTATTATCGGCCAAATCGCATCCGTCTTATATTTTGCACTCTTCCTCATTCTTGTCCCACTAGCAGGATGGGTGGAAAATAAAGCATTGAAATGAGCCHM560226Telestessp                      ATGGCAAGCCTACGGAAAACCCACCCGCTAATAAAAATCGCTAATGACGCGCTAGTCGACCTCCCAACACCATCTAATATCTCAGCAATGTGGAACTTCGGTTCGCTCCTGGGATTATGTTTAATTACCCAGATCCTAACAGGATTATTCTTAGCCATACATTATACCTCTGACATCTCAACCGCATTCTCATCAGTAACTCACATCTGCCGAGACGTCAATTACGGCTGACTTATCCGAAGCCTGCATGCTAACGGAGCATCATTTTTCTTCATCTGCCTTTACATACATATCGCACGAGGACTGTACTATGGATCCTACCTTTATAAGGAGACCTGAAACATCGGTGTAGTACTGTTCCTTCTAGTTATGATGACGGCCTTCGTCGGCTACGTTCTTCCATGAGGACAAATATCCTTTTGAGGTGCTACCGTAATTACAAATCTCCTCTCAGCGGTCCCTTACATAGGGGATACCCTTGTTCAATGAATCTGAGGCGGTTTCTCAGTAGACAATGCAACTCTCACACGGTTCTTCGCGTTCCACTTCCTCCTACCATTTGTCGTTGCCGGCGCAACCATTCTACACTTATTGTTCTTACACGAAACCGGATCAAACAACCCGGCCGGACTAAATTCCGACGCAGATAAAATTTCTTTCCACCCGTACTTCTCATATAAAGACCTTCTTGGCTTTGTGGCAATGTTACTAGCCCTAACCTCTCTAACATTGTTCTCCCCCAACCTTCTGGGTGACCCGGAAAACTTTACCCCAGCAAACCCCCTTGTAACGCCTCCTCATATCCAGCCAGAGTGATACTTTTTGTTTGCCTACGCCATCCTCCGATCTATCCCAAATAAACTAGGAGGGGTTCTTGCACTACTATTCAGCATCCTGGTACTAATAGTTGTGCCGATTTTACACACATCTAAACAACGAGGACTAACTTTCCGGCCAGTGACTCAATTCCTATTCTGAACCCTTGTTGCAGATATATTTATTTTGACATGAATCGGGGGCATACCCGTAGAACACCCATATATTATTATCGGCCAAATCGCATCCGTCTTATATTTTGCACTCTTCCTCATTCTTGTCCCACTAGCAGGATGGGTGGAAAATAAAGCATTGAAATGAGCCAY509825Telestesturskyi                 atggcaagcctacgaaaaacccacccactaataaagatcgctaatggcgcactagtcgacctccccacaccgtctaacatctcagcaatatgaaactttggttctctcctgggactatgtttaattacccaaatcctaacaggattgttcttagccatacattatacctctgacatctcaaccgcattctcatcagtaactcacatctgccgagacgttaattacggctgacttatccgaagcctacatgctaacggagcatcctttttcttcatctgcctttacatgcatatcgcacgaggcctatactacgggtcctacctttataaggagacctgaaacattggcgtagtcctattccttctagtcatgataacagccttcgtcggctacgttcttccatgaggacaaatatccttttgaggtgctaccgtaattacgaatctcctctcagcggtcccttacatgggagatacccttgttcaatgaatctgaggcggtttctcagtggacaatgcacctctcacacgattcttcgcattccacttcctcctgccgtttgtcgtagccggcgcgaccattctacacttattatttttacacgagactgggtcaaacaaccccgccgggctaaattccgacgcagataaaatttctttccacccctacttctcatataaagaccttcttgggtttgtggcaatgctactagcccttacctctctaacattgttctcccctaacctactgggtgacccggaaaactttacccccgctaacccactcgtaacgccgccacacattcagccggagtgatacttcttgtttgcctacgccatcctccgatctattccaaacaagctaggaggagttcttgcgctactattcagcatccttgtactgatagtcgtcccgattttacacacatcgaaacaacgcggactaactttccgcccagtgactcaattcctattctgagcccttgttgcagatatatttattttgacctgaatcgggggcatacccgtagaacacccatatattattatcggccaaatcgcatccgtcctatactttgcactcttcctcgtccttgtcccaccagcgggatgggtggagaataaagcattgaaatgagccHM560227Telestesturskyi                 ATGGCAAGCCTACGAAAAACCCACCCACTAATAAAGATCGCTAATGGCGCACTAGTCGACCTCCCCACACCGTCTAACATCTCAGCAATATGAAACTTCGGTTCTCTCCTGGGACTATGTTTAATTACCCAAATCCTAACAGGATTGTTCTTAGCCATACATTATACCTCTGACATCTCAACCGCATTCTCATCAGTAACTCACATCTGCCGAGACGTTAATTACGGCTGACTTATCCGAAGCCTACATGCTAACGGAGCATCCTTTTTCTTCATCTGCCTTTACATGCATATCGCACGAGGCCTATACTACGGGTCCTACCTTTATAAGGAGACCTGAAACATTGGCGTAGTCCTATTCCTTCTAGTCATGATAACAGCCTTCGTCGGCTACGTTCTTCCATGAGGACAAATATCCTTTTGAGGTGCTACCGTAATTACGAATCTCCTCTCAGCGGTCCCTTACATGGGAGATACCCTTGTTCAATGAATCTGAGGcGGTTTCTCAGTGGACAATGCAACACTCACACGATTCTTCGCATTCCACTTCCTCCTGCCGTTTGTCGTAGCCGGCGCGACCATTCTACACTTATTATTTTTACACGAGACGGGGTCAAACAACCCCGCCGGGCTAAATTCCGACGCAGATAAAATTTCTTTCCACCCCTACTTCTCATATAAAGACCTTCTTGGGTTTGTGGCAATGCTACTAGCCCTTACCTCTCTAACATTGTTCTCCCCTAACCTACTGGGTGACCCGGAAAACTTTACCCCCGCTAACCCACTCGTAACGCCGCCACACATTCAGCCGGAGTGATACTTCTTGTTTGCCTACGCCATCCTCCGATCTATTCCAAACAAGCTAGGAGGAGTTCTTGCGCTACTATTCAGCATCCTTGTACTGATAGTCGTCCCGATTTTACACACATCGAAACAACGCGGACTAACTTTCCGCCCAGTGACTCAATTCCTATTCTGAGCCCTTGTTGCAGATATATTTATTTTGACCTGAATCGGGGGCATACCCGTAGAACACCCATATATTATTATCGGCCAAATCGCGTCCGTCCTATATTTTGCACTCTTCCTGATTCTTTTCCCACTAGCAGGATGGGTGGAAAATAAAGCATTAAAATGAGCCHM560228Telestesukliva                  ATGGCAAGCCTACGAAAAACCCACCCACTAATAAAAATCGCTAATGGCGCACTAGCCGACCTCCCCACACCATCTAACATCTCAGCAATGTGAAACTTCGGTTCTCTCCTGGGACTATGTTTAATTACCCAAATCCTGACAGGATTGTTCTTAGCCATACATTACACCTCTGACATCTCGACCGCATTCTCATCAGTAACTCACATCTGCCGAGACGTCAATTACGGCTGGCTTATCCGGAGCCTACATGCTAACGGAGCATCCTTTTTCTTCATCTGCCTTTACATACATATCGCACGGGGCCTATACTATGGGTCCTACCTTTATAAGGAGACCTGAAACATTGGTGTGGTCCTATTCCTTCTAGTTGTGATAACAGCCTTCGTCGGCTACGTTCTTCCATGGGGACAAATGTCCTTTTGAGGTGCTACCGTAATTACAAATCTCCTCTCAGCGGTCCCTTACATGGGAGATACCCTTGTTCAGTGAATCTGAGGCGGTTTCTCAGTAGACAATGCAACGCTCACACGATTCTTCGCGTTCCATTTCCTCCTACCATTTGTCGTCGCCGGCGCAACCATTCTACACTTATTATTCTTACACGAGACGGGGTCAAATAACCCCGCCGGACTAAATTCCGACGCAGATAAAATTTCTTTCCACCCCTACTTCTCATATAAAGACCTTCTTGGGTTTGTGGCAATGTTACTAGCCCTTACCTCTCTAACATTATTCTCCCCTAACCTACTGGGTGACCCGGAAAACTTTACCCCCGCTAACCCGCTCGTAACGCCGCCACATATCCAGCCAGAATGATACTTCTTATTTGCCTACGCCATCCTCCGATCTATTCCGAATAAGCTAGGAGGGGTTCTTGCACTACTATTCAGCATCCTTGTACTAATAGTCGTGCCGATTTTACACACATCGAAACAACGAGGACTAACTTTCCGCCCGGTGACTCAATTCCTATTCTGAACCCTTGTTGCAGATATATTTATTTTGACCTGAATCGGCGGCATACCCGTAGAACACCCATATATTATTATCGGCCAAATCGCGTCCGTCCTATACTTTGCACTCTTCCTGATTCTTGTCCCACTAGCAGGATGGGTGGAAAATAAAGCATTAAAATGAGCCHM560229Telestesukliva                  ATGGCAAGCCTACGAAAAACCCACCCACTAATAAAAATCGCTAATGGCGCACTAGTCGACCTCCCCACACCATCTAACATCTCAGCAATGTGAAACTTCGGTTCTCTCCTGGGACTATGTTTAATTACCCAAATCCTGACAGGATTGTTCTTAGCCATACATTACACCTCTGACATCTCGACCGCATTCTCATCAGTAACTCACATCTGCCGAGACGTCAATTACGGCTGGCTTATCCGGAGCCTACATGCTAACGGAGCATCCTTTTTCTTCATCTGCCTTTACATACATATCGCACGGGGCCTATACTATGGGTCCTACCTTTATAAGGAGACCTGAAACATTGGTGTGGTCCTATTCCTTCTAGTTATGATAACAGCCTTCGTCGGCTACGTTCTTCCATGGGGACAAATGTCCTTTTGAGGTGCTACCGTAATTACAAATCTCCTCTCAGCGGTCCCTTACATGGGAGATACCCTTGTTCAGTGAATCTGAGGCGGTTTCTCAGTAgACAATGCAACGCTCACACGATTCTTCGCGTTCCATTTCCTCCTACCATTTGTCGTCGCCGGCGCAACCATTCTACACTTATTATTCTTACACGAGACGGGGTCAAATAACCCCGCCGGACTAAATTCCGACGCAGATAAAATTTCTTTCCACCCCTACTTCTCATATAAAGACCTTCTTGGGTTTGTGGCAATGTTACTAGCCCTTACCTCTCTAACATTATTCTCCCCTAACCTACTGGGTGACCCGGAAAACTTTACCCCCGCTAACCCGCTCGTAACGCCGCCACATATCCAGCCAGAATGATACTTCTTATTTGCCTACGCCATCCTCCGATCTATTCCGAATAAGCTAGGAGGGGTTCTTGCACTACTATTCAGCATCCTTGTACTAATAGTCGTGCCGATTTTACACACATCGAAACAACGAGGACTAACTTTCCGCCCGGTGACTCAATTCCTATTCTGAACCCTTGTTGCAGATATATTTATTTTGACCTGAATCGGCGGCATACCCGTAGAACACCCATATATTATTATCGGCCAAATCGCGTCCGTCCTATACTTTGCACTCTTCCTGATTCTTGTCCCACTAGCAGGATGGGTGGAAAATAAAGCATTAAAATGAGCCHM560230Tincatinca                      ATGGCAAGCCTACGAAAAACCCACCCCCTAATTAAAATTGCTAACGATGCACTAGTTGATTTACCAACACCCTCTAACATCTCAGTATGATGAAACTTCGGGTCCCTCCTTGGACTATGCTTAATTATCCAAATCTTAACAGGATTATTTTTAGCTATACATTACACCTCAGATATTTCAACCGCATTCTCGTCAGTAAACCACATTTGCCGTGATGTAAACTATGGCTGACTTATTCGTAACTTACACGCTAATGGGGCATCATTCTTCTTTATCTGCCTTTATATACATATCGCCCGAGGATTATATTACGGATCATACCTTTACAAAGAGACCTGAAATATTGGAGTAGTTCTTTTTCTATTAGTAATAATAACAGCCTTTGTTGGCTACGTCCTGCCATGAGGACAAATATCCTTTTGAGGCGCAACAGTAATTACTAACCTACTATCAGCAGTTCCCTACATAGGAGATGCTTTAGTTCAATGAATCTGAGGTGGCTTCTCAGTAGACAATGCAACACTTACGCGATTCTTCGCATTCCACTTCTTACTCCCATTCATTGTTGCCGCCGCCACCCTCCTACACCTGCTATTTTTACACGAAACAGGATCAAACAACCCAACAGGACTAAACTCCGACGCAGACAAAATCTCCTTCCACCCCTACTTTTCATATAAAGACCTTCTAGGGTTCGTAATTATATTATTAGCCCTCACATCACTAGCACTATTCTCTCCAAACTTATTAGGAGACCCAGAAAATTTTACCCCAGCAAACCCCTTAGTCACACCTCCACACATTCAGCCAGAATGATATTTCTTATTTGCCTACGCCATTTTACGATCAATCCCAAACAAGCTAGGAGGTGTTCTTGCACTATTATTCTCTATTTTAGTACTAATGGTGGTACCGATCTTACATACCTCAAAACAACGAGGACTTACATTCCGCCCAATCACTCAATTCTTATTCTGAACCTTGGTAGCGGATATAGTAATCTTAACATGAATCGGCGGTATACCTGTAGAACACCCATATATCATCATTGGTCAAATTGCATCAATTCTATACTTCGCACTTTTCCTTGTTCTCGCCCCCCTCGCAGGATGACTGGAAAATAAAGCATTGAAATGAGCTHM560231Tropidophoxinellushellenicus    ATGGCAAGCCTACGGAAAACCCACCCACTAATAAAAATCGCTAATGACGCACTAGTTGACCTTCCAACACCATCCAATATTTCAGCACTATGAAACTTTGGGTCCCTTCTAGGACTATGTTTAATTACCCAAATCTTAACAGGATTGTTCCTAGCTATACACTATACCTCTGACATCTCAACCGCATTTTCATCAGTAACACACATCTGCCGCGACGTCAACTACGGCTGACTAATTCGGAGCCTACATGCTAACGGAGCATCTTTCTTCTTCATCTGTCTTTATATACACATTGCACGAGGCCTATACTACGGATCATACCTTTACAAAGAAACTTGAAACATCGGCGTAGTGCTATTTCTTTTAGTTATAATAACAGCCTTCGTCGGCTATGTACTCCCATGAGGACAAATATCCTTTTGAGGCGCCACCGTAATTACAAATCTCCTCTCAGCAGTTCCCTACATAGGAGACACCCTTGTCCAGTGAATCTGGGGTGGCTTCTCAGTAGACAATGCAACCCTCACACGATTCTTCGCATTCCACTTCCTCCTGCCATTCGTCGTAACAGGCGCAACTGTATTCCACCTACTGTTTCTACACGAAACAGGGTCAAACAACCCGGCCGGACTAAACTCCGACGCGGACAAAATCTCCTTCCACCCATATTTTTCATATAAGGACCTCCTTGGCTTCGTAATTATACTACTAGCCCTCACATCTTTGGCATTATTTTCTCCCAACCTACTAGGTGACCCTGAAAATTTCACTCCAGCAAACCCACTTGTGACACCTCCACATATTCAGCCAGAGTGATATTTCCTATTTGCCTACGCTATTCTTCGATCCATTCCAAATAAACTAGGAGGTGTTCTTGCATTACTATTCAGTATCCTCGTGCTAATAGTTGTGCCTATTTTACACACTTCAAAACAACGAGGACTAACTTTCCGCCCTATGACTCAATTTCTGTTCTGGACCCTAGTTGCAGACATAATTATTCTAACATGAATTGGAGGCATACCTGTAGAACACCCATACATTATTATTGGCCAAATTGCATCTATCTTATACTTTGCGCTCTTCCTTATCCTTATCCCACTAGCAGGATtAATAGAAAACAAAGCATTGAAATGAGCCHM560232Tropidophoxinellushellenicus    ATGGCAAGCCTACGGAAAACCCACCCACTAATAAAAATCGCTAATGACGCACTAGTTGACCTTCCAACACCATCCAATATTTCAGCACTATGAAACTTTGGGTCCCTTCTAGGACTATGTTTAATTACCCAAATCTTAACAGGATTGTTCCTAGCTATACACTATACCTCTGACATCTCAACCGCATTTTCATCAGTAACACACATCTGCCGCGACGTCAACTACGGCTGACTAATTCGGAGCCTACATGCTAACGGAGCATCTTTCTTCTTCATCTGTCTTTATATACACATTGCACGAGGCCTATACTACGGATCATACCTTTACAAAGAAACTTGAAACATCGGCGTAGTGCTATTTCTTTTAGTTATAATAACAGCCTTCGTCGGCTATGTACTCCCATGAGGACAAATATCCTTTTGAGGCGCCACCGTAATTACAAATCTCCTCTCAGCAGTTCCCTACATAGGAGACACCCTTGTCCAGTGAATCTGAGGTGGCTTCTCAGTAGACAATGCAACCCTCACACGATTCTTCGCATTCCACTTCCTCCTGCCATTCGTCGTAACAGGCGCAACTGTATTCCACCTACTGTTTCTACACGAAACAGGGTCAAACAACCCGGCCGGACTAAACTCCGACGCGGACAAAATCTCCTTCCACCCATATTTTTCATATAAGGACCTCCTTGGCTTCGTAATTATACTACTAGCCCTCACATCTTTGGCATTATTTTCTCCCAACCTACTAGGTGACCCTGAAAATTTCACTCCAGCAAACCCACTTGTGACACCTCCACATATTCAGCCAGAGTGATATTTCCTATTTGCCTACGCTATTCTTCGATCCATTCCAAATAAACTAGGAGGTGTTCTTGCATTACTATTCAGTATCCTCGTGCTAATAGTTGTGCCTATTTTACACACTTCAAAACAACGAGGACTAACTTTCCGCCCTATGACTCAATTTCTGTTCTGGACCCTAGTTGCAGACATAGATATTCTAACATGAATTGGAGGCATACCTGTAGAACACCCATACATTATTATTGGCCAAATTGCATCTATCTTATACTTTGCGCTCTTCCTTATCCTTATCCCACTAGCAGGATTAATAGAAAACAAAGCATTGAAATGAGCCHM560233Tropidophoxinellusspartiaticus  ATGGCAAGCCTACGAAAAACCCACCCACTAATAAAAATCGCTAATGACGCACTAGTCGACCTTCCAACACCATCCAACATTTCAGCGCTATGAAACTTCGGGTCCCTTTTAGGACTGTGTTTAATTACCCAAATCTTAACAGGATTGTTCCTAGCTATACACTACACCTCTGACATCTCAACCGCATTTTCATCAGTAACACACATCTGCCGGGACGTCAACTACGGCTGACTAATTCGAAGTCTACATGCCAACGGAGCATCTTTCTTCTTCATCTGTCTTTATATACACATTGCACGAGGTCTATACTACGGATCATATCTTTATAAAGAGACTTGAAACATCGGCGTAGTACTATTTCTTTTAGTTATAATAACAGCCTTCGTCGGCTATGTACTCCCATGAGGACAAATATCCTTTTGAGGCGCCACCGTAATTACAAACCTCCTCTCAGCAGTTCCCTACATGGGAGACACCCTTGTCCAGTGAATCTGAGGTGGTTTCTCAGTAGACAACGCAACCCTCACACGGTTCTTCGCGTTCCACTTCCTCCTCCCATTTGTCGTAACGGGCGCAACTGTACTTCACCTACTATTCCTACACGAAACAGGGTCGAACAACCCAGCCGGACTAAACTCCGACGCGGACAAAATCTCCTTTCACCCCTATTTTTCGTACAAGGACCTCCTCGGCTTCGTAATTATACTACTAGCCCTCACATCTTTGGCATTATTTTCTCCCAACTTACTAGGTGATCCTGAAAATTTCACTCCAGCAAACCCACTTGTGACACCCCCACATATCCAACCAGAATGATACTTCCTATTTGCCTATGCCATTCTTCGATCCATTCCAAATAAGCTAGGAGGTGTTCTTGCATTACTATTCAGTATCCTCGTGCTAATAGTTGTGCCTATTTTACATACTTCAAAACAACGAGGATTAACTTTCCGCCCTATGACTCAATTCCTGTTCTGGACCCTAGTTGCAGACATGATTATCTTAACATGAATTGGGGGCATGCCTGTAGAACACCCATATATTGTTATTGGCCAAGTCGCATCTATCTTATACTTTGCACTCTTCCTTATCCTTATTCCACTAGCAGGGTTAATGGAGAACAAAGCATTGAAATGAGCCHM560234Tropidophoxinellusspartiaticus  ATGGCAAGCCTACGAAAAACCCACCCACTAATAAAAATCGCTAATGACGCACTAGTCGACCTTCCAACACCATCCAACATTTCAGCGCTATGAAACTTCGGGTCCCTTTTAGGACTGTGTTTAATTACCCAAATCTTAACAGGATTGTTCCTAGCTATACACTACACCTCTGACATCTCAACCGCATTTTCATCAGTAACACACATCTGCCGGGACGTCAACTACGGCTGACTAATTCGAAGTCTACATGCCAACGGAGCATCTTTCTTCTTCATCTGTCTTTATATACACATTGCACGAGGTCTATACTACGGATCATATCTTTATAAAGAGACTTGAAACATCGGCGTAGTACTATTTCTTTTAGTTATAATAACAGCCTTCGTCGGCTATGTACTCCCATGAGGACAAATATCCTTTTGAGGCGCCACCGTAATTACAAACCTCCTCTCAGCAGTTCCCTACATGGGAGACACCCTTGTCCAGTGAATCTGAGGTGGTTTCTCAGTAGACAACGCAACCCTCACACGGTTCTTCGCGTTCCACTTCCTCCTCCCATTTGTCGTAACGGGCGCAACTGTACTTCACCTACTATTCCTACACGAAACAGGGTCGAACAACCCAGCCGGACTAAACTCCGACGCGGACAAAATCTCCTTTCACCCCTATTTTTCGTACAAGGACCTCCTCGGCTTCGTAATTATACTACTAGCCCTCACATCTTTGGCATTATTTTCTCCCAACTTACTAGGTGATCCTGAAAATTTCACTCCAGCAAACCCACTTGTGACACCCCCACATATCCAACCAGAATGATACTTCCTATTTGCCTATGCCATTCTTCGATCCATTCCAAATAAGCTAGGAGGTGTTCTTGCATTACTATTCAGTATCCTCGTGCTAATAGTTGTGCCTATTTTACATACTTCAAAACAACGAGGATTAACTTTCCGCCCTATGACTCAATTCCTGTTCTGGACCCTAGTTGCAGACATGATTATCTTAACATGAATTGGGGGCATGCCTGTAGAACACCCATATATTGTTATTGGCCAAGTCGCATCTATCTTATACTTTGCACTCTTCCTTATCCTTATTCCACTAGCAGGGTTAATGGAGAACAAAGCATTGAAATGAGCCHM560235Vimbamelanops                  ATGGCAAGCCTACGAAAAACCCACCCACTAATAAAAATCGCTAACGGCGCACTAGTCGACCTTCCAACACCATCCAATATTTCAGCACTATGAAACTTCGGGTCCCTCCTAGGATTGTGTCTCATTACCCAAATCCTCACGGGATTATTTCTGGCCATGCATTACACCTCTGATATTTCCACCGCATTTTCGTCAGTAACCCACATTTGCCGAGACGTCAATTACGGCTGACTCATTCGAAATTTACACGCTAATGGAGCATCATTCTTCTTCATCTGTCTCTATATACATATTGCGCGGGGCCTATACTACGGGTCATATCTTTACAAAGAGACCTGGAATATTGGCGTAGTCCTATTTCTTCTAGTTATAATAACAGCCTTCGTCGGCTACGTACTTCCGTGAGGGCAGATGTCTTTTTGAGGTGCCACCGTAATTACGAACCTCCTCTCAGCAGTCCCTTATATAGGCGACACCCTTGTCCAATGAATCTGAGGCGGTTTCTCANNNNNNNNNNNNNNNNNNNNNNNNNNNNNNNNNNNNNNNNNNNNNNNNNNNNNNNNNNNNNNNNNNNNNNNNNNNNNNNNNNNNNNNNNNNNNNNNNNNNNNNNNNNNNNNNNNNNNNNNNNNNNNNNNNNNNNNNNNNNNNNNNNNNNNNNNNNNNNNNNNNNNNNNNNNNNNNNNNNNNNNNNNNNNNNNNNNNNNNNNNNNNNNNNNNNNNNNNNNNNNNNNNNNNNNNNNNNNNNNNNNNNNNNNNNNNNNNNNNNNNNNNNNNNNNNNNNNNNNNNNNNNNNNNNNNNNNNNNNNNNNNNNNNNNNNNNNNNNNNNNNNNNNNNNNNNNNNNNNNNNNNNNNNNNNNNNNNNNNNNNNNNNNNNNNNNNNNNNNNNNNNNNNNNNNNNNNNNNNNNNNNNNNNNNNNNNNNNNNNNNNNNNNNNNNNNNNNNNNNNNNNNNNNNNNNNNNNNNNNNNNNNNNNNNNNNNNNNNNNNNNNNNNNNNNNNNNNNNNNNNNNNNNNNNNNNNNNNNNNNNNNNNNNNNNNNNNNNNNNNNNNNNNNNNNNNNNNNNNNNNNNNNNNNNNNNNNNNNNNNNNNNNNNNNNNNNNNNNNNNNNNNNNNNNNNNNNNNNNNNNNNNNNNNNNNNHM560236Vimbamelanops                   ATGGCAAGCCTACGAAAAACCCACCCACTAATAAAAATCGCTAACGGCGCACTAGTCGACCTTCCAACACCATCCAATATTTCAGCACTATGAAACTTCGGGTCCCTCCTAGGATTGTGTCTCATTACCCAAATCCTCACGGGATTATTTCTGGCCATGCATTACACCTCTGATATTTCCACCGCATTTTCGTCAGTAACCCACATTTGCCGAGACGTCAATTACGGCTGACTCATTCGAAATTTACACGCTAATGGAGCATCATTCTTCTTCATCTGTCTCTATATACATATTGCGCGGGGCCTATACTACGGGTCATATCTTTACAAAGAGACCTGGAATATTGGCGTAGTCCTATTTCTTCTAGTTATAATAACAGCCTTCGTCGGCTACGTACTTCCGTGAGGGCAGATGTCTTTTTGAGGTGCCACCGTAATTACGAACCTCCTCTCAGCAGTCCCTTATATAGGCGACACCCTTGTCCAATGAATCTGAGGCGGTTTCTCANNNNNNNNNNNNNNNNNNNNNNNNNNNNNNNNNNNNNNNNNNNNNNNNNNNNNNNNNNNNNNNNNNNNNNNNNNNNNNNNNNNNNNNNNNNNNNNNNNNNNNNNNNNNNNNNNNNNNNNNNNNNNNNNNNNNNNNNNNNNNNNNNNNNNNNNNNNNNNNNNNNNNNNNNNNNNNNNNNNNNNNNNNNNNNNNNNNNNNNNNNNNNNNNNNNNNNNNNNNNNNNNNNNNNNNNNNNNNNNNNNNNNNNNNNNNNNNNNNNNNNNNNNNNNNNNNNNNNNNNNNNNNNNNNNNNNNNNNNNNNNNNNNNNNNNNNNNNNNNNNNNNNNNNNNNNNNNNNNNNNNNNNNNNNNNNNNNNNNNNNNNNNNNNNNNNNNNNNNNNNNNNNNNNNNNNNNNNNNNNNNNNNNNNNNNNNNNNNNNNNNNNNNNNNNNNNNNNNNNNNNNNNNNNNNNNNNNNNNNNNNNNNNNNNNNNNNNNNNNNNNNNNNNNNNNNNNNNNNNNNNNNNNNNNNNNNNNNNNNNNNNNNNNNNNNNNNNNNNNNNNNNNNNNNNNNNNNNNNNNNNNNNNNNNNNNNNNNNNNNNNNNNNNNNNNNNNNNNNNNNNNNNNNNNNNNNNNNNNNNNNNNNNNNNNNNNAY026410Vimbamirabilis                  atggcaagcctacgaaaaacccacccactaataaaaatcgctaacggcgcactagtcgaccttccaacaccatccaatatttcggcactatgaaacttcgggtccctcctgggattatgtctaattacccaaatcctcacgggattatttctggccatgcattacacctctgatatttccaccgcgttttcgtcagtaacccacatctgccgagacgttaattacggctgactcattcgaaatttacacgctaatggagcatcattcttcttcatctgcctctatatacatattgcgcgaggcctatactacggatcatatctttacaaggagacctgaaatattggcgtagtcctatttcttctagttataataacagccttcgtcggctacgtacttccgtgaggacagatgtctttttgaggtgccaccgtaattacgaacctcctctcagcagtcccttatataggcgacacccttgtccaatgaatctgaggcggtttttcagtagataacgcaactctcacacggttcttcgcattccacttcctcctaccattcgttgtcgccggcgcaaccctcttacacctactattcctacacgagacggggtcaaacaacccagccggattaaactccgacgcagataaaatttctttccacccatacttctcatataaagaccttcttggctttgtaattatattactagcccttacttctctagcattattttctcctaacctattaggtgacccagaaaattttactccagcaaacccactcgtgacacccccacatattcagccagaatgatatttcttatttgcttacgccattctccgatccattccaaataaactaggaggggtgcttgcattattattcagtattttagtgctaatagttgtgccaatcttacatacctcaaaacaacgaggactaactttccgtcctataacacaattcttattctgaaccttagttgcagacataatcattttgacatgaatcggaggcatacccgtagaacacccatatattgttattggccaaatcgcatccattttatattttgcactctttctcgttcttattccactagcaggatgaatagagaacaaagcattgaaatgagcc  HM560237Vimbavimba                      ATGGCAAGCCCACGAAAAACCCACCCACTAATAAAAATCGCTAACGGCGCACTAGTCGACCTTCCAACACCATCTAATATTTCGGCACTATGAAACTTCGGGTCCCTCCTAGGATTGTGTCTAATTACCCAAATCCTCACGGGATTATTTTTGGCCATACATTACACCTCTGATATTTCCACCGCATTTTCATCAGTAACCCACATCTGCCGAGACGTCAATTACGGCTGACTCATTCGAAACCTGCACGCTAATGGAGCATCATTCTTCTTCATCTGTCTCTATATACATATTGCGCGAGGCCTATACTACGGATCATATCTTTACAAAGAAACCTGAAATATTGGCGTAGTCCTATTTCTTCTAGTTATAATAACAGCCTTCGTCGGCTACGTGCTTCCGTGAGGACAGATGTCTTTTTGAGGTGCCACCGTAATTACAAACCTCCTCTCAGCAGTCCCTTATATAGGCGACACCCTTGTCCAATGAATCTGAGGCGGTTTCTCAGTAGATAACGCAACTCTCACACGGTTCTTCGCATTCCACTTCCTTCTACCATTCGTTGTCGCCGGCGCAACCCTCTTACACCTACTATTCCTACACGAGACGGGGTCAAACAACCCAGCCGGATTAAACTCCGACGCGGATAAAATTTCTTTCCACCCATACTTCTCATATAAAGACCTTCTTGGCTTTGTAATCATGTTACTAGCCCTTACTTCTCTAGCATTATTTTCTCCTAACCTATTAGGTGACCCAGAAAATTTTACTCCAGCAAACCCACTCGTGACACCCCCACATATTCAGCCAGAATGATATTTCTTATTTGCTTACGCCATTCTCCGATCCATTCCAAATAAACTAGGAGGGGTCCTTGCATTATTATTCAGTATTTTAGTGCTAATAGTTGTGCCAATCTTACATACCTCAAAACAACGAGGACTAACTTTCCGTCCTATAACACAATTCTTATTCTGAACCTTAGTTGCAGACATAATCATTTTGACATGAATCGGAGGCATACCCGTAGAACACCCATATATTATTATTGGCCAAATCGCATCCATTTTATATTTTGCACTCTTCCTCATTCTTATTCCACTAGCAGGATGAATAGAGAACAAAGCATTGAAATGAGCCAY026404Vimbavimba                      atggcaagcctacgaaaaacccacccactaataaaaatcgctaacggcgcactagtcgaccttccaacaccatctaatatttcggcactatgaaacttcgggtccctcctaggattgtgtctaattacccaaatcctcacgggattatttttggccatacattacacctctgatatttccaccgcattttcatcagtaacccacatctgccgagacgtcaattacggctgactcattcgaaacttgcacgctaatggagcatcattcttcttcatctgtctctatatacatattgcgcgaggcctatactacggatcatatctttacaaagaaacctgaaatattggcgtagtcctatttcttctagttataataacagccttcgtcggctacgtgcttccgtgaggacagatgtctttttgaggtgccaccgtaattacaaacctcctctcagcagtcccttatataggcgacacccttgtccaatgaatctgaggcggtttctcagtagataacgcaactctcacacggttcttcgcattccacttccttctaccattcgttgtcgccggcgcaaccctcttacacctactattcctacacgagacggggtcaaacaacccagccggattaaactccgacgcggataaaatttctttccacccatacttctcatataaagaccttcttggctttgtaatcatgttactagcccttacttctctagcattattttctcctaacctattaggtgacccagaaaattttactccagcaaacccactcgtgacacccccacatattcagccagaatgatatttcttatttgcttacgccattctccgatccattccaaataaactaggaggggtccttgcattattattcagtattttagtgctaatagttgtgccaatcttacatacctcaaaacaacgaggactaactttccgtcctataacacaattcttattctgaaccttagttgcagacataatcattttgacatgaatcggaggcatacccgtagaacacccatatattattattggccaaatcgcatccattttatattttgcactcttcctcattcttattccactagcaggatgaatagagaacaaagcattgaaatgagcc;End;2.- Cytochrome Oxidase I (COI) alignment used in mitochondrial phylogenetic analysis #NEXUS Begin data;Dimensions ntax=155 nchar=646;Format datatype=dna gap=-;Matrix HM560238Alburnoidesbipunctatus           GTGGGGACTGCCTTAAGCCTCCTTATTCGGGCCGAACTAAGCCAACCCGGGTCACTTTTAGGCGATGACCAAATTTATAATGTTATTGTTACTGCCCACGCCTTCGTAATAATTTTCTTTATAGTAATGCCAATTCTTATTGGCGGGTTCGGAAACTGACTCGTCCCACTAATGATTGGAGCACCTGATATAGCATTCCCACGAATAAATAATATAAGCTTCTGACTACTACCTCCATCATTTTTGCTACTATTAGCCTCTTCTGGTGTTGAGGCCGGGGCCGGAACTGGGTGAACGGTATACCCACCACTTGCAGGCAATCTTGCCCACGCAGGCGCATCAATTGACTTAACGATCTTTTCACTGCATCTAGCAGGCGTATCATCAATCTTGGGCGCAGTCAACTTTATTACCACAATTATTAATATGAAACCTCCGGCTATCTCCCAGTATCAAACACCCCTCTTTGTATGAGCCGTGCTAGTAACAGCTGTCCTTCTCCTATTATCATTACCAGTTTTAGCTGCCGGGATTACAATGCTTCTTACAGACCGTAATCTTAATACCACTTTCTTCGACCCGGCAGGAGGGGGAGATCCAATCTTATATCAACACTTATTCTGATTCTTTGGCCACCCAGAAGTCTNC_008659Alburnusalburnus                GTggggactgccctaagcctccttatccgagccgaactaagccagcctgggtcacttttaggtgatgatcaaatttataatgtcatcgttaccgcccacgcctttgtaataattttctttatagtcatgccaattcttattggggggtttggaaactgactcgtcccactaatgattggtgcacccgacatagcattcccacgaataaataacatgagtttctggctccttcccccatcattcctactgctattagcttcttctggagttgaggccggcgctgggacggggtgaacagtatacccacccctcgcaggcaatcttgcccatgcaggagcatcagtagatttaacaatcttctcactccatctagcaggtgtatcatcaattttaggtgcagttaacttcattaccacaattattaatatgaaacctccagccatctcccaatatcaaacacccctctttgtgtgagccgtactagtaacggctgtccttctcctcctctcactaccagttctagctgctggaattacaatgcttcttacggatcgtaatcttaataccacattcttcgatccggcagggggaggagacccaatcctatatcaacacttattctgattctttggccatccagaggtttHM560239Alburnusalburnus                 GTGGGGACTGCCCTAAGCCTCCTTATCCGAGCCGAACTAAGCCAGCCTGGGTCACTTTTAGGTGATGATCAAATTTATAATGTCATCGTTACCGCCCACGCCTTTGTAATAATTTTCTTTATAGTCATGCCAATTCTTATTGGAGGGTTTGGAAACTGACTCGTCCCACTAATGATTGGTGCACCCGACATAGCATTCCCACGAATAAATAACATGAGTTTCTGGCTCCTTCCCCCATCATTCCTACTGCTATTAGCTTCTTCTGGAGTTGAGGCCGGCGCTGGGACGGGGTGAACAGTATACCCACCCCTCGCAGGCAATCTTGCCCATGCAGGAGCATCAGTAGATTTAACAATCTTCTCACTCCATCTAGCAGGTGTATCATCAATTTTAGGTGCAGTTAACTTCATTACCACAATTATTAATATGAAACCTCCAGCCATCTCCCAATATCAAACACCCCTCTTTGTGTGAGCCGTACTAGTAACGGCTGTCCTTCTCCTCCTCTCACTACCAGTTCTAGCTGCTGGAATTACAATGCTTCTTACGGATCGTAATCTTAATACCACATTCTTCGATCCGGCAGGGGGAGGAGACCCAATCCTATATCAACACTTATTCTGATTCTTTGGCCACCCAGAAGTCTHM056240Alburnusalburnus                 GTGGGGACTGCCCTAAGCCTCCTTATCCGAGCCGAACTAAGCCAGCCTGGGTCACTTTTACGTGATGATCAAATTTATAATGTCATCGTTACCGCCCACGCCTTTGTAATAATTTTCTTTATAGTCATGCCAATTCTTATTGGAGGGTTTGGAAACTGACTCGTCCCACTAATGATTGGTGCACCCGACATAGCATTCCCACGAATAAATAACATGAGTTTCTGGCTCCTTCCCCCATCATTCCTACTGCTATTAGCTTCTTCTGGAGTTGAGGCCGGCGCTGGGACGGGGTGAACAGTATACCCACCCCTCGCAGGCAATCTTGCCCATGCAGGAGCATCAGTAGATTTAACAATCTTCTCACTCCATCTAGCAGGTGTATCATCAATTTTAGGTGCAGTTAACTTCATTACCACAATTATTAATATGAAACCTCCAGCCATCTCCCAATATCAAACACCCCTCTTTGTGTGAGCCGTACTAGTAACGGCTGTCCTTCTCCTCCTCTCACTACCAGTTCTAGCTGCTGGAATTACAATGCTTCTTACGGATCGTAATCTTAATACCACATTCTTCGATCCGGCAGGGGGAGGAGACCCAATCCTATATCAACACTTATTCTGATTCTTTGGCCACCCAGAAGTCTHM560241Alburnusalburnus                 GTGGGGACTGCCCTAAGCCTCCTTATCCGAGCCGAACTAAGCCAGCCTGGGTCACTTTTAGGTGATGATCAAATTTATAATGTCATCGTTACCGCCCACGCCTTTGTAATAATTTTCTTTATAGTCATGCCAATTCTTATTGGAGGGTTTGGAAACTGACTCGTCCCACTAATGATTGGTGCACCCGACATAGCATTCCCACGAATAAATAACATGAGTTTCTGGCTCCTTCCCCCATCATTCCTACTGCTATTAGCTTCTTCTGGAGTTGAGGCCGGCGCTGGGACGGGGTGAACAGTATACCCACCCCTCGCAGGCAATCTTGCCCATGCAGGAGCATCAGTAGATTTAACAATCTTCTCACTCCATCTAGCAGGTGTATCATCAATTTTAGGTGCAGTTAACTTCATTACCACAATTATTAATATGAAACCTCCAGCCATCTCCCAATATCAAACACCCCTCTTTGTGTGAGCCGTACTAGTAACGGCTGTCCTTCTCCTCCTCTCACTACCAGTTCTAGCTGCTGGAATTACAATGCTTCTTACGGATCGTAATCTTAATACCACATTCTTCGACCCGGCAGGGGGAGGAGACCCAATCCTATATCAACACTTATTCTGATTCTTTGGCCACCCAGAAGTCTHM560242Alburnusarborella                GTGGGGACTGCCCTAAGCCTCCTTATCCGAGCCGAACTAAGCCAGCCTGGGTCACTTTTAGGTGATGATCAAATTTATAATGTCATCGTTACCGCCCACGCCTTTGTAATAATTTTCTTTATAGTCATGCCAATTCTTATTGGAGGGTTTGGAAACTGACTCGTCCCACTAATGATCGGTGCGCCTGATATAGCATTCCCACGAATAAATAATATGAGTTTCTGGCTCCTTCCCCCATCATTCCTACTGTTATTAGCTTCTTCTGGCGTTGAGGCCGGAGCTGGAACGGGGTGAACAGTATACCCGCCCCTCGCAGGTAATCTTGCCCATGCAGGAGCATCAGTAGATTTAACAATCTTCTCACTTCATCTAGCAGGTGTGTCATCAATTTTAGGTGCAGTTAACTTCATTACCACAATTATTAATATGAAACCTCCAGCCATCTCCCAATATCAAACACCCCTCTTTGTGTGAGCCGTACTAGTAACGGCTGTCCTTCTCCTCCTCTCACTACCAGTTCTAGCTGCTGGAATTACAATGCTTCTTACAGACCGTAATCTTAATACCACATTCTTCGACCCGGCAGGAGGAGGAGACCCAATCTTATACCAACACTTATTCTGATTCTTTGGCCACCCAGAAGTCTHM560243Alburnusarborella                GTGGGGACTGCCCTAAGCCTCCTTATCCGAGCCGAACTAAGCCAGCCTGGGTCACTTTTAGGTGATGATCAAATTTATAATGTCATCGTTACCGCCCACGCCTTTGTAATAATTTTCTTTATAGTCATGCCAATTCTTATTGGAGGGTTTGGAAACTGACTCGTCCCACTAATGATCGGTGCGCCTGATATAGCATTCCCACGAATAAATAATATGAGTTTCTGGCTCCTTCCCCCATCATTCCTACTGTTATTAGCTTCTTCTGGCGTTGAGGCCGGAGCTGGAACGGGGTGAACAGTATACCCGCCCCTCGCAGGTAATCTTGCCCATGCAGGAGCATCAGTAGATTTAACAATCTTCTCACTTCATCTAGCAGGTGTGTCATCAATTTTAGGTGCAGTTAACTTCATTACCACAATTATTAATATGAAACCTCCAGCCATCTCCCAATATCAAACACCCCTCTTTGTGTGAGCCGTACTAGTAACGGCTGTCCTTCTCCTCCTCTCACTACCAGTTCTAGCTGCTGGAATTACAATGCTTCTTACAGACCGTAATCTTAATACCACATTCTTCGACCCGGCAGGAGGAGGAGACCCAATCTTATACCAACACTTATTCTGATTCTTTGGCCACCCAGAAGTCTHM560244Alburnusbelvica                  GTGGGGACTGCCCTAAGCCTCCTTATCCGAGCCGAGCTAAGCCNNNCTGGGNNACTTNNNNGNGATNATNNNNNNTNTNANNNNNNNNNTACCGNCCNNNNCNNNNNNATAATTTTCTTTATAGTCATGCCAATTCTTATTGGAGGGTTTGGAAACTGACTCGTCCCGCTAATGATTGGTGCGCCTGATATAGCATTCCCACGAATAAATAATATGAGTTTCTGACTCCTTCCCCCATCATTCCTACTGCTATTAGCTTCTTCTGGCGTTGAGGCCGGAGCCGGAACGGGATGAACGGTATACCCACCCCTCGCAGGTAATCTTGCCCATGCAGGCGCATCAGTAGATTTGACAATCTTCTCACTCCATCTAGCAGGTGTATCATCAATTTTAGGTGCAGTTAACTTCATTACCACAATTATTAATATGAAACCTCCAGCCATCTCCCAATATCAAACACCCCTCTTTGTATGAGCCGTACTAGTAACGGCTGTCCTTCTCCTCCTCTCACTACCAGTTCTAGCTGCTGGAATTACAATGCTTCTTACAGACCGTAATCTTAATACCACATTCTTCGACCCGGCAGGAGGAGGAGACCCAATCTTATACCAACACTTATTCTGATTCTTTGGCCACCCAGAAGTCTHM560246Alburnusescherichii              GTGGGGACTGCCCTAAGCCTCCTTATCCGAGCCGAACTAAGCCAGCCTGGGTCACTTTTAGGTGATGATCAAATTTATAATGTCATCGTTACCGCCCACGCCTTTGTAATAATTTTCTTTATAGTCATGCCAATTCTTATTGGGGGGTTTGGAAACTGACTCGTCCCACTAATGATTGGTGCACCCGATATAGCATTCCCACGAATAAATAACATGAGTTTCTGGCTCCTTCCCCCATCATTCCTACTGCTATTAGCTTCTTCTGGAGTTGAGGCCGGCGCTGGGACGGGATGAACAGTATACCCACCCCTCGCAGGCAATCTTGCCCATGCAGGAGCATCAGTAGATTTAACAATCTTCTCACTCCATCTAGCAGGTGTATCATCAATTTTAGGTGCAGTTAACTTCATTACCACAATTATTAATATGAAACCTCCAGCCATCTCCCAATATCAAACACCCCTCTTTGTGTGAGCCGTACTAGTAACGGCTGTCCTTCTCCTCCTCTCACTACCAGTTCTAGCTGCTGGAATTACAATGCTTCTTACAGATCGTAATCTTAATACCACATTCTTCGACCCGGCAGGGGGAGGAGACCCAATCCTATATCAACACTTATTCTGATTCTTTGGCCACCCAGAAGTCTHM560245Alburnussp1                      GTGGGGACTGCCCTAAGCCTCCTTATCCGAGCCGAACTAAGCCAGCCTGGGTCACTTTTAGGTGATGATCAAATTTATAATGTCATCGTTACCGCCCACGCCTTTGTAATAATTTTCTTTATAGTCATGCCTATTCTTATTGGAGGGTTTGGAAACTGACTCGTCCCACTAATGATTGGGGCACCCGATATAGCATTCCCACGAATAAATAATATGAGTTTCTGGCTCCTTCCCCCATCATTCCTACTGCTATTAGCTTCTTCTGGAGTTGAGGCCGGAGCTGGGACGGGATGAACAGTATACCCACCCCTCGCAGGTAATCTTGCCCATGCAGGAGCATCAGTAGATTTAACAATCTTCTCACTCCATCTAGCAGGTGTATCATCAATTTTAGGTGCAGTTAACTTCATTACCACAATTATTAATATGAAACCTCCAGCCATCTCCCAATATCAAACACCCCTCTTTGTGTGAGCCGTACTAGTAACGGCTGTCCTTCTCCTCCTCTCACTACCAGTTCTAGCTGCCGGAATTACAATGCTTCTTACAGACCGTAATCTTAACACCACATTCTTCGACCCGGCAGGGGGAGGAGACCCAATCCTATATCAACACTTATTCTGATTCTTTGGCCACCCAGAAGTCTHM560247Alburnusfilippii                 GTGGGGACTGCCCTAAGCCTCCTTATCCGGGCCGAACTAAGCCAGCCTGGGTCACTCTTAGGTGATGATCAAATTTATAATGTCATCGTTACCGCCCACGCCTTTGTAATAATTTTCTTTATAGTCATGCCAATTCTTATTGGAGGATTTGGAAACTGACTCGTCCCACTAATGATTGGTGCACCCGATATAGCATTCCCACGAATAAATAACATGAGTTTCTGACTCCTTCCGCCATCATTCCTACTGCTATTAGCTTCTTCTGGGGTTGAGGCCGGGGCTGGGACGGGATGAACAGTATACCCACCCCTCGCAGGCAATCTTGCCCACGCAGGAGCATCAGTAGATTTAACAATCTTCTCACTCCATCTAGCAGGTGTATCATCAATTTTAGGTGCAGTCAACTTCATTACCACCATTATTAATATGAAACCCCCAGCCATCTCCCAATATCAAACGCCTCTCTTTGTGTGAGCCGTACTAGTAACAGCTGTCCTTCTCCTCCTCTCACTACCAGTTCTGGCTGCTGGAATCACAATGCTTCTTACAGATCGTAATCTTAATACCACATTCTTCGACCCGGCAGGGGGAGGAGACCCAATCTTATATCAACACTTATTCTGATTCTTTGGCCACCCAGAAGTCTHM560248Alburnusfilippii                 GTGGGGACTGCCCTAAGCCTCCTTATCCGGGCCGAACTAAGCCAGCCTGGGTCACTCTTAGGTGATGATCAAATTTATAATGTCATCGTTACCGCCCACGCCTTTGTAATAATTTTCTTTATAGTCATGCCAATTCTTATTGGAGGATTTGGAAACTGACTCGTCCCACTAATGATTGGTGCACCCGATATAGCATTCCCACGAATAAATAACATGAGTTTCTGACTCCTTCCGCCATCATTCCTACTGCTATTAGCTTCTTCTGGGGTTGAGGCCGGGGCTGGGACGGGATGAACAGTATACCCACCCCTCGCAGGCAATCTTGCCCACGCAGGAGCATCAGTAGATTTAACAATCTTCTCACTCCATCTAGCAGGTGTATCATCAATTTTAGGTGCAGTCAACTTCATTACCACCATTATTAATATGAAACCCCCAGCCATCTCCCAATATCAAACGCCTCTCTTTGTGTGAGCCGTACTAGTAACAGCTGTCCTTCTCCTCCTCTCACTACCAGTTCTGGCTGCTGGAATCACAATGCTTCTTACAGATCGTAATCTTAATACCACATTCTTCGACCCGGCAGGGGGAGGGGACCCAATCTTATATCAACACTTATTCTGATTCTTTGGCCACCCAGAAGTCTHM560249Alburnuskotschyi                 GTGGGGACTGCCCTAAGCCTCCTTATTCGAGCCGAACTAAGCCAACCTGGGTCACTTTTAGGCGATGATCAAATCTATAATGTCATCGTTACCGCCCACGCCTTCGTAATAATTTTCTTTATAGTCATGCCAATTCTTATTGGAGGGTTTGGAAACTGACTCGTCCCACTAATGATTGGTGCACCCGATATAGCATTCCCACGAATAAATAACATGAGTTTCTGACTCCTTCCCCCATCATTCCTGCTACTACTGGCTTCTTCTGGAGTCGAGGCTGGGGCTGGAACGGGGTGAACAGTATATCCACCCCTCGCAGGCAATCTTGCCCACGCAGGAGCATCAGTAGATTTAACAATCTTCTCACTTCACCTGGCAGGTGTATCATCAATTTTAGGTGCAGTCAACTTCATCACCACAATTATCAACATGAAGCCCCCAGCCATTTCCCAGTATCAAACACCCCTCTTTGTGTGAGCCGTGCTAGTAACAGCCGTCCTGCTCCTCCTCTCACTACCAGTTCTAGCTGCTGGAATTACAATGCTTCTCACAGATCGTAATCTTAATACTACATTCTTCGACCCGGCAGGGGGAGGAGACCCGATCTTGTATCAACACTTATTCTGATTCTTTGGCCACCCAGAAGTCTHM560250Anaecyprishispanica              GTGGGGACTGCCTTAAGCCTCCTTATCCGAGCCGAACTGAGCCAACCTGGATCCCTTTTAGGTGATGACCAGATTTATAATGTTATCGTTACCGCCCACGCCTTTGTAATAATTTTCTTTATAGTCATGCCAATTCTTATCGGAGGGTTTGGGAACTGACTTGTTCCACTAATGATTGGTGCACCAGATATAGCATTCCCCCGAATAAATAACATGAGTTTCTGACTCCTTCCCCCCTCATTCCTACTCCTATTAGCTTCTTCCGGGGTTGAAGCCGGCGCTGGAACAGGTTGAACAGTATACCCACCCCTCGCAGGCAACCTTGCCCACGCAGGAGCATCAGTAGATTTGACAATCTTCTCACTGCATCTAGCAGGTGTATCATCAATTTTAGGCGCAGTCAACTTCATCACCACAATTATCAACATGAAACCCCCAGCCATCTCCCAATACCAAACACCCCTCTTTGTATGGGCCGTATTGGTAACAGCGGTCCTTCTCCTCCTCTCACTACCAGTTCTGGCTGCCGGAATTACAATGCTTCTCACAGATCGTAATCTTAACACCACATTCTTCGACCCGGCAGGGGGAGGTGACCCAATCTTATATCAACACTTATTCTGATTCTTTGGCCACCCAGAAGTCTHM560251Leuciscusaspius                  GTGGGGACTGCCCTAAGCCTCCTTATTCGGGCCGAACTAAGCCAACCCGGGTCACTTTTAGGCGATGACCAAATTTACAACGTTATCGTTACCGCCCACGCCTTCGTAATAATTTTCTTTATAGTAATGCCAATCCTTATTGGCGGGTTCGGAAACTGACTCGTCCCACTAATGATTGGCGCACCTGATATGGCATTCCCACGAATAAATAATATAAGCTTCTGACTTCTACCCCCATCATTCCTATTGCTACTAGCTTCTTCTGGTGTTGAAGCCGGGGCTGGAACAGGATGAACAGTGTACCCCCCACTCGCAGGCAATCTCGCCCACGCAGGAGCATCAGTGGACTTAACAATCTTCTCGCTTCACCTAGCAGGTGTATCATCAATTTTAGGCGCGGTCAACTTCATCACCACAATTATCAACATGAAACCCCCAGCCATCTCCCAGTATCAAACACCCCTCTTTGTATGAGCCGTGCTGGTAACAGCCGTCCTTCTCCTTCTATCTCTACCAGTTTTAGCTGCCGGAATTACAATGCTTCTTACAGATCGTAATCTCAACACTACATTCTTCGACCCAGCAGGGGGAGGTGACCCAATCTTATATCAACACTTATTTTGATTCTTTGGCCACCCAGAAGTCTHM560252Bliccabjoerkna                   GTGGGGACTGCCCTAAGCCTCCTTATTCGGGCCGAACTAAGCCAACCCGGGTCACTTTTAGGCGATGACCAGATTTACAATGTCATCGTTACCGCCCACGCCTTCGTAATAATTTTCTTTATAGTAATGCCAATTCTTATTGGAGGGTTCGGAAACTGACTCGTCCCACTAATAATCGGTGCGCCTGATATGGCATTCCCACGAATAAATAATATAAGCTTCTGACTTCTACCCCCATCGTTCCTACTACTATTAGCCTCTTCTGGTGTTGAGGCTGGTGCTGGGACAGGATGAACAGTATACCCGCCACTTGCAGGCAATCTCGCCCATGCAGGAGCATCAGTAGATTTAACAATCTTCTCCCTCCACCTAGCAGGTGTATCATCAATTTTAGGGGCAGTCAACTTCATTACCACAATCATTAACATGAAACCCCCAGCCATTTCCCAGTACCAAACACCCCTCTTTGTATGAGCCGTACTAGTAACAGCCGTCCTTCTCCTCCTATCATTACCAGTTTTAGCTGCCGGAATTACAATACTTCTTACAGACCGTAATCTTAATACCACATTCTTCGACCCAGCAGGGGGAGGAGACCCAATCTTATATCAACACTTATTCTGATTCTTTGGCCACCCAGAAGTCTHM560253Bliccabjoerkna                   NNNNNNNNNNNNNTAAGCCTCCTTATTCGGGCCGAACTAAGCCAACCCGGGTCACTTTTAGGCGATGACCAGATTTACAATGTCATCGTTACCGCCCACGCCTTCGTAATAATTTTCTTTATAGTAATGCCAATTCTTATTGGAGGGTTCGGAAACTGACTCGTCCCACTAATAATCGGTGCGCCTGATATGGCATTCCCACGAATAAATAATATAAGCTTCTGACTTCTACCCCCATCGTTCCTACTACTATTAGCCTCTTCTGGTGTTGAAGCTGGTGCTGGGACAGGATGAACAGTATACCCGCCACTTGCAGGCAATCTCGCCCATGCAGGAGCATCAGTAGATTTAACAATCTTCTCCCTCCACCTAGCAGGTGTATCATCAATTTTAGGGGCAGTCAACTTCATTACCACAATCATTAACATGAAACCCCCAGCCATTTCCCAGTACCAAACACCCCTCTTTGTATGAGCCGTACTAGTAACAGCCGTCCTTCTCCTCCTATCATTACCAGTTTTAGCTGCCGGAATTACAATACTTCTTACAGACCGTAATCTTAATACCACATTCTTCGACCCAGCAGGGGGAGGAGACCCAATCTTATATCAACACTTATTCTGATTCTTTGGCCACCCAGAAGTCTHM560254Chondrostomaangorense            NNNNNNNNNNNNNNNNNNNNNNNNNNNNNNNCCGAACTAAGCCAACCCGGGTCACTTTTAGGTGATGACCAAATTTATAATGTCATCGTCACCGCCCACGCCTTCGTAATAATTTTCTTTATAGTAATGCCAATTCTTATCGGGGGATTTGGAAACTGACTTGTCCCACTAATAATTGGTGCACCCGACATGGCATTTCCACGAATAAATAACATGAGCTTCTGACTTCTACCCCCCTCATTCCTCTTATTATTAGCCTCTTCTGGAGTTGAGGCCGGGGCCGGAACGGGGTGAACAGTATACCCGCCGCTTGCAGGCAATCTTGCCCACGCAGGTGCATCAGTAGATTTAACAATCTTCTCACTTCACCTGGCAGGTGTATCATCAATTTTAGGCGCAGTCAACTTCATTACCACAATTATTAATATGAAACCCCCAGCCATCTCCCAATATTAAANACCTCTNTTTGTATGAGCCGTGNTAGTANCNGCCGTGCTTCTCCTCCTATAACTACCAGTTCTAGCTGCCGGAATNNCTATGCTTCTTACAGATCGTAATCTTAATACCACCTTCNNCGNCCCGGNAGGAGGAGGAGACCCAATCCTATATCAACACTTANNNNGANNNNNNNNNNNNNNNGANNNNNHM560255Chondrostomaknerii               GTGGGGACTGCCCTAAGCCTCCTTATTCGGGCCGAACTAAGCCAACCCGGGTCACTTTTAGGTGATGACCAAATTTATAATGTCATCGTCACCGCCCACGCCTTCGTAATAATTTTCTTTATAGTAATGCCAATTCTTATTGGGGGATTCGGAAACTGACTTGTCCCACTAATAATTGGTGCACCCGACATGGCATTTCCACGAATAAATAACATGAGTTTCTGACTTCTACCACCATCATTCCTCCTATTATTAGCCTCTTCTGGCGTTGAGGCCGGGGCTGGAACAGGATGAACAGTATACCCGCCGCTTGCAGGCAATCTTGCCCACGCAGGTGCATCAGTAGATTTAACAATCTTCTCACTTCACCTAGCAGGTGTGTCATCAATTTTAGGAGCAGTCAACTTCATTACCACAATTATTAATATGAAACCCCCAGCCATCTCCCAATATCAAACACCTCTATTTGTATGAGCCGTGCTAGTAACAGCCGTGCTTCTCCTCCTATCATTACCAGTTCTAGCTGCCGGAATCACTATGCTTCTTACAGATCGTAATCTTAACACCACATTCTTCGACCCGGCAGGAGGAGGAGACCCAATCCTATACCAACACTTATTCTGATTCTTTGGCCACCCAGAAGTCTHM560256Chondrostomaknerii               GTGGGGACTGCCCTAAGCCTCCTTATTCGGGCCGAACTAAGCCAACCCGGGTCACTTTTAGGTGATGACCAAATTTATAATGTCATCGTCACCGCCCACGCCTTCGTAATAATTTTCTTTATAGTAATGCCAATTCTTATTGGGGGATTCGGAAACTGACTTGTCCCACTAATAATTGGTGCACCCGACATGGCATTTCCACGAATAAATAACATGAGTTTCTGACTTCTACCACCATCATTCCTCCTATTATTAGCCTCTTCTGGCGTTGAGGCCGGGGCTGGAACAGGATGAACAGTATACCCGCCGCTTGCAGGCAATCTTGCCCACGCAGGTGCATCAGTAGATTTAACAATCTTCTCACTTCACCTAGCAGGTGTGTCATCAATTTTAGGAGCAGTCAACTTCATTACCACAATTATTAATATGAAACCCCCAGCCATCTCCCAATATCAAACACCTCTATTTGTATGAGCCGTGCTAGTAACAGCCGTGCTTCTCCTCCTATCATTACCAGTTCTAGCTGCCGGAATCACTATGCTTCTTACAGATCGTAATCTTAACACCACATTCTTCGACCCGGCAGGAGGAGGAGACCCAATCCTATACCAACACTTATTCTGATTCTTTGGCCACCCAGAAGTCTHM560258Chondrostomaoxyrhynchum          GTGGGGACTGCCCTAAGCCTCCTAATTCGGGCCGAACTAAGCCAACCCGGGTCACTTTTAGGTGATGACCAAATTTATAATGTCATCGTCACCGCCCACGCCTTCGTAATAATTTTCTTTATAGTAATGCCAATTCTTATCGGGGGATTTGGAAACTGACTTGTCCCGCTAATGATTGGTGCACCCGACATGGCATTCCCACGAATAAACAACATGAGCTTCTGACTTCTACCCCCCTCATTCCTATTATTATTAGCCTCTTCTGGAGTTGAGGCCGGGGCCGGAACGGGGTGAACGGTATACCCCCCACTTGCAGGCAATCTTGCCCACGCGGGAGCATCAGTAGATTTAACAATCTTCTCACTCCACCTGGCAGGTGTGTCATCAATTTTAGGCGCAGTCAACTTCATTACCACAATTATTAATATGAAACCCCCAGCCATCTCCCAATATCAAACACCTCTCTTTGTGTGAGCCGTGCTAGTAACAGCCGTGCTTCTCCTCCTATCACTACCAGTTCTAGCTGCCGGAATCACTATGCTTCTTACAGATCGTAATCTTAACACCACATTCTTCGACCCGGCAGGAGGAGGAGACCCAATCCTATATCAACACTTATTCTGATTCTTTGGCCACCCAGAAGTCTHM560259Chondrostomaoxyrhynchum          GTGGGGACTGCCCTAAGCCTCCTAATTCGGGCCGAACTAAGCCAACCCGGGTCACTTTTAGGTGATGACCAAATTTATAATGTCATCGTCACCGCCCACGCCTTCGTAATAATTTTCTTTATAGTAATGCCAATTCTTATCGGGGGATTTGGAAACTGACTTGTCCCGCTAATGATTGGTGCACCCGACATGGCATTCCCACGAATAAACAACATGAGCTTCTGACTTCTACCCCCCTCATTCCTATTATTATTAGCCTCTTCTGGAGTTGAGGCCGGGGCCGGAACGGGGTGAACGGTATACCCCCCACTTGCAGGCAATCTTGCCCACGCGGGAGCATCAGTAGATTTAACAATCTTCTCACTCCACCTGGCAGGTGTGTCATCAATTTTAGGCGCAGTCAACTTCATTACCACAATTATTAATATGAAACCCCCAGCCATCTCCCAATATCAAACACCTCTCTTTGTGTGAGCCGTGCTAGTAACAGCCGTGCTTCTCCTCCTATCACTGCCAGTTCTAGCTGCCGGAATCACTATGCTTCTTACAGATCGTAATCTTAACACCACATTCTTCGACCCGGCAGGAGGAGGAGACCCAATCCTATATCAACACTTATTCTGATTCTTTGGCCACCCAGAAGTCTHM560257Chondrostomaphoxinus             GTGGGGACTGCCCTAAGCCTCCTTATTCGGGCCGAACTAAGCCAACCCGGGTCACTTTTAGGTGATGACCAAATTTATAATGTCATCGTCACCGCCCACGCCTTCGTAATAATTTTCTTTATAGTAATGCCAATTCTTATTGGGGGATTCGGAAACTGACTTGTCCCACTAATAATTGGTGCACCCGACATGGCATTTCCACGAATAAATAACATGAGTTTCTGACTTCTACCACCATCGTTCCTCCTATTATTAGCCTCTTCTGGCGTTGAGGCCGGGGCTGGAACAGGATGAACAGTATACCCGCCGCTTGCAGGCAATCTTGCCCACGCAGGTGCATCAGTAGATTTAACAATCTTCTCACTTCACCTAGCAGGTGTGTCATCAATTTTAGGGGCAGTCAACTTCATTACCACAATTATTAATATGAAACCCCCAGCCATCTCCCAATATCAAACACCTCTCTTTGTATGAGCCGTACTAGTAACAGCCGTGCTTCTCCTCCTATCATTGCCAGTTCTAGCTGCCGGAATCACTATGCTTCTTACAGATCGTAATCTTAATACCACATTCTTCGACCCAGCAGGAGGAGGAGACCCAATCCTATACCAACACTTATTCTGATTCTTTGGCCACCCAGAAGTCTHM560260Chondrostomavardarense           GTGGGGACTGCCCTAAGCCTCCTAATTCGGGCCGAACTAAGCCAACCCGGGTCACTTTTAGGTGATGACCAAATTTATAATGTCATCGTCACCGCCCACGCCTTCGTAATAATTTTCTTTATAGTAATGCCAATTCTTATTGGGGGATTTGGAAACTGACTTGTCCCATTAATAATTGGTGCGCCCGACATGGCATTTCCACGAATAAATAATATGAGCTTCTGACTTCTACCCCCCTCATTCCTCTTATTATTAGCCTCTTCTGGAGTTGAGGCCGGGGCTGGAACAGGATGAACGGTATACCCGCCGCTTGCAGGCAATCTTGCCCACGCAGGTGCATCAGTAGATTTAACAATCTTCTCACTCCACCTGGCAGGTGTATCATCAATTTTAGGCGCAGTCAACTTCATCACCACAATTATTAATATGAAACCCCCAGCCATCTCCCAATATCAAACACCTCTCTTTGTATGAGCCGTGTTAGTAACAGCCGTGCTTCTCCTCCTATCACTACCAGTTCTAGCTGCCGGAATCACTATGCTCCTTACAGATCGTAATCTTAACACCACATTCTTCGACCCGGCAGGAGGAGGAGACCCAATCCTATATCAACACTTATTCTGATTCTTTGGCCACCCAGAAGTCTHM560263Delminichthysadpersus            GTGGGGACTGCTTTAAGCCTCCTTATTCGAGCCGAACTAAGCCAACCGGGGTCACTTTTAGGCGATGACCAAATTTATAACGTTATTGTTACCGCCCACGCCTTCGTAATAATTTTCTTTATAGTAATGCCAATTCTTATCGGGGGTTTCGGAAACTGACTCGTCCCACTAATAATTGGTGCACCTGATATAGCGTTCCCACGAATAAATAACATGAGTTTTTGACTTCTGCCCCCGTCATTCCTGCTGCTCCTAGCCTCTTCCGGTGTTGAGGCTGGCGCTGGGACAGGGTGAACAGTCTATCCCCCCCTTGCTGGCAACCTTGCCCACGCAGGAGCATCGGTAGACTTAACCATCTTCTCACTTCACCTGGCAGGTGTGTCATCAATTTTAGGTGCAGTCAATTTCATCACCACAATTATTAATATGAAACCCCCAGCCATCTCCCAATATCAAACACCTCTATTTGTATGGTCCGTACTGGTAACAGCCGTCCTTCTCCTGCTATCGTTACCAGTTTTAGCTGCCGGAATTACAATACTTCTCACAGACCGTAATCTCAATACTACATTCTTCGACCCGTCAGGGGGAGGAGACCCAATTTTATATCAACACTTATTCTGATTCTTTGGCCACCCAGAAGTCTHM560264Delminichthysadpersus            GTGGGGACTGCTTTAAGCCTCCTTATTCGAGCCGAACTAAGCCAACCGGGGTCACTTTTAGGCGATGACCAAATTTATAACGTTATTGTTACCGCCCACGCCTTCGTAATAATTTTCTTTATAGTAATGCCAATTCTTATCGGGGGTTTCGGAAACTGACTCGTCCCACTAATAATTGGTGCACCTGATATAGCGTTCCCACGAATAAATAACATGAGTTTTTGACTTCTGCCCCCGTCATTCCTGCTGCTCCTAGCCTCTTCCGGTGTTGAGGCTGGCGCTGGGACAGGGTGAACAGTCTATCCCCCCCTTGCTGGCAACCTTGCCCACGCAGGAGCATCGGTAGACTTAACCATCTTCTCACTTCACCTGGCAGGTGTGTCATCAATTTTAGGTGCAGTCAATTTCATCACCACAATTATTAATATGAAACCCCCAGCCATCTCCCAATATCAAACACCTCTATTTGTATGGTCCGTACTGGTAACAGCCGTCCTTCTCCTGCTATCGTTACCAGTTTTAGCTGCCGGAATTACAATACTTCTCACAGACCGTAATCTCAATACTACATTCTTCGACCCGTCAGGGGGAGGAGACCCAATTTTATATCAACACTTATTCTGATTCTTTGGCCACCCAGAAGTCTHM560265Delminichthysghetaldii           GTGGGGACTGCTTTAAGCCTCCTTATTCGAGCCGAACTAAGCCAACCGGGGTCACTTTTAGGCGATGACCAAATTTATAACGTTATTGTTACCGCCCACGCCTTCGtAATAATTTTCTTTATAGTAATGCCAATTCTTATCGGAGGTTTCGGAAACTGACTCGTCCCGCTAATGATTGGTGCACCTGATATAGCGTTCCCGCGAATAAATAACATGAGTTTTTGACTTCTGCCTCCGTCATTCCTGCTGCTCCTAGCCTCTTCCGGTGTTGAGGCTGGCGCTGGAACAGGGTGAACAGTCTATCCCCCCCTTTCTGGCAACCTTGCCCACGCAGGAGCATCAGTAGACTTAACCATCTTCTCACTTCACCTGGCAGGTGTGTCATCAATTTTAGGTGCAGTCAATTTCATCACCACAATTATTAATATAAAACCCCCAGCCATCTCCCAATATCAAACACCTCTATTTGTGTGGTCCGTACTGGTAACGGCCGTCCTTCTCCTGCTATCGTTACCAGTTCTAGCTGCCGGAATTACAATACTTCTCACAGACCGTAATCTTAATACCACCTTCTTCGATCCGTCAGGAGGAGGAGACCCAATTTTATATCAACACTTATTCTGATTCTTTGGCCACCCAGAAGTCTHM560261Delminichthyskrbavensis          GTGGGGACTGCTTTAAGCCTCCTTATTCGAGCCGAACTAAGCCAACCGGGGTCACTTTTAGGCGATGACCAAATTTATAACGTTATCGTTACCGCCCACGCCTTCGTAATAATTTTCTTTATAGTAATGCCAATTCTTATCGGAGGTTTCGGAAACTGACTCGTCCCGCTAATAATTGGTGCACCTGATATAGCGTTCCCGCGAATAAATAACATGAGTTTTTGACTTCTGCCTCCGTCATTCCTACTGCTCCTAGCCTCTTCCGGTGTTGAGGCTGGTGCTGGGACAGGGTGAACAGTCTATCCCCCCCTTGCTGGCAACCTTGCTCACGCAGGAGCATCAGTAGACTTAACCATCTTCTCACTTCACCTAGCAGGTGTGTCATCCATTTTAGGTGCAGTCAATTTCATCACCACAATTATTAATATAAAACCCCCAGCCATCTCCCAGTATCAAACACCTCTATTTGTATGGTCCGTACTGGTAACAGCCGTCCTTCTCCTGCTATCGTTACCAGTTCTAGCTGCCGGAATTACAATACTTCTCACAGACCGTAATCTCAATACTACATTCTTCGACCCGTCAGGGGGAGGAGACCCAATTTTATATCAACACTTATTCTGATTCTTTGGCCACCCAGAAGTCTHM560262Delminichthyskrbavensis          GTGGGGACTGCTTTAAGCCTCCTTATTCGAGCCGAACTAAGCCAACCGGGGTCACTTTTAGGCGATGACCAAATTTATAACGTTATCGTTACCGCCCACGCCTTCGTAATAATTTTCTTTATAGTAATGCCAATTCTTATCGGAGGTTTCGGAAACTGACTCGTCCCGCTAATAATTGGTGCACCTGATATAGCGTTCCCGCGAATAAATAACATGAGTTTTTGACTTCTGCCTCCGTCATTCCTACTGCTCCTAGCCTCTTCCGGTGTTGAGGCTGGTGCTGGGACAGGGTGAACAGTCTATCCCCCCCTTGCTGGCAACCTTGCTCACGCAGGAGCATCAGTAGACTTAACCATCTTCTCACTTCACCTAGCAGGTGTGTCATCAATTTTAGGTGCAGTCAATTTCATCACCACAATTATTAATATAAAACCCCCAGCCATCTCCCAGTATCAAACACCTCTATTTGTATGGTCCGTACTGGTAACAGCCGTCCTTCTCCTGCTATCGTTACCAGTTCTAGCTGCCGGAATTACAATACTTCTCACAGACCGTAATCTCAATACTACATTCTTCGACCCGTCAGGGGGAGGAGACCCAATTTTATATCAACACTTATTCTGATTCTTTGGCCACCCAGAAGTCTHM560266Gobiogobio                       GTNGGGACTGCTTTAAGCCTCCTCATTCGAGCTGAGCTGAGCCAACCTGGCTCACTTCTAGGTGATGACCAAATTTATAATGTAATCGTCACTGCCCACGCCTTCGTAATAATTTTCTTTATAGTAATACCAATTCTCATTGGAGGGCTTGGAAACTGGCTTGTACCACTAATAATTGGGGCCCCAGACATGGCGATCCCACGAATAAACAACATAAGCTTCTGACTTCTACCCCCATCATTTCTTCTATTACTAGCCTCTTCAGGAGTCGAGGCTGGAGCGGGCACAGGGTGAACAGTTTATCCCCCACTATCAGGCAATCTTGCCCATGCAGGAGCCTCAGTAGACCTCACGAGTTTCTCACTACATTTAGCAGGTGTTTCATCAATTTTAGGGGCTATCAATTTTATTACCACCACTATTAACATGAAACCCCCAGCCATCTCACAGTATCAAACCCCCCTATTCGAATGGTCAGTACTGGGGACAGCGGTACTTCTACTCTTTTCACTTCCTGTCCTGGCTGCCGGAATTACAATGCTTCTTACAGACCGTAATCTTAACACTACATTCTTTGACCCGGCAGGGGGAGGAGACCCAATCCTTTACCAACACCTATTNNNNNNNNNNNNNNNNNNNNNNNNNNHM560267Iberochondrostomalemmingii       GTGGGGACTGCCCTGAGCCTCCTTATTCGAGCCGAACTAAGCCAGCCCGGGTCACTTTTAGGTGATGACCAAATTTATAATGTCATCGTCACTGCCCACGCCTTCGTAATAATTTTCTTTATAGTAATGCCAATTCTTATTGGCGGCTTCGGGAACTGACTTGTCCCACTAATGATTGGTGCACCCGACATGGCATTTCCGCGAATAAATAACATGAGCTTTTGACTTCTACCGCCATCATTCCTCCTATTATTGGCCTCTTCTGGTGTTGAAGCCGGGGCTGGCACAGGATGAACGGTATACCCGCCACTTGCAGGCAATCTTGCCCACGCGGGCGCATCAGTAGACTTAACAATCTTCTCACTTCATCTAGCAGGTGTATCGTCAATTTTAGGCGCAGTCAATTTCATTACCACAATTATTAACATGAAACCTCCAGCCATCTCCCAATATCAAACACCCCTCTTTGTGTGAGCTGTGCTAGTAACAGCCGTGCTACTCCTCCTATCACTACCAGTTCTAGCTGCCGGAATCACTATGCTTCTTACAGATCGTAATCTAAATACCACATTCTTCGATCCGGCGGGGGGAGGAGACCCAATCTTATATCAACACCTGTTCTGATTCTTTGGCCACCCAGAAGTCTHM560268Iberochondrostomaoretanum        GTGGGGACTGCCCTAAGCCTCCTTATTCGGGCCGAACTAAGCCAGCCCGGGTCACTTTTAGGTGATGACCAAATTTATAATGTCATCGTCACCGCCCACGCCTTCGTAATAATTTTCTTTATAGTAATGCCAATTCTTATTGGGGGCTTCGGGAACTGACTTGTCCCACTAATAATTGGTGCACCCGACATGGCATTTCCGCGAATAAATAACATAAGCTTTTGACTTCTACCGCCATCATTCCTCCTATTATTAGCCTCTTCTGGTGTTGAGGCCGGGGCTGGCACAGGATGAACGGTATACCCGCCACTTGCAGGCAATCTTGCCCACGCAGGTGCATCAGTAGACTTAACAATCTTCTCGCTCCATCTAGCAGGTGTATCATCAATTTTAGGCGCAGTCAATTTCATTACCACAATTATTAACATGAAACCTCCAGCCATCTCCCAATATCAAACACCCCTATTTGTATGAGCCGTGCTAGTAACAGCCGTGCTTCTCCTCCTATCACTACCAGTTCTGGCTGCCGGGATCACTATGCTTCTTACGGATCGTAATCTAAATACCACATTCTTCGACCCGGCGGGGGGAGGAGACCCAATCTTATATCAGCATCTGTTCTGATTCTTTGGCCACCCAGAAGTCTHM560269Ladigesocyprisghigii
[truncated: 891,494 more chars]
